# Celastrol alleviates comorbid obesity and depression by directly binding amygdala HnRNPA1 in a mouse model

**DOI:** 10.1002/ctm2.394

**Published:** 2021-06-06

**Authors:** Chunyan Zhu, Jun Yang, Yongping Zhu, Jiahao Li, Hongyu Chi, Congmin Tian, Yuqing Meng, Yanqing Liu, Jigang Wang, Na Lin

**Affiliations:** ^1^ Institute of Chinese Materia Medica China Academy of Chinese Medical Sciences Beijing China; ^2^ Shenzhen People's Hospital Shenzhen China; ^3^ Central People's Hospital of Zhanjiang Zhanjiang China

Dear Editor,

Obesity and depression often co‐occur and lack of effective treatment.^1^, ^2^ Our team revealed celastrol‐mediated curations on this comorbidity in mice bearing comorbid obesity and depression (COM). These effects may be associated with the direct binding and downregulation of heterogeneous nuclear ribonucleoprotein A1 (HnRNPA1) in the basolateral nucleus of the amygdala (BLA).

COM mice were constructed by double stresses from high‐fat diet and environment (Figure 1A). Compared with chow, COM mice were significantly obese (Figure 1B) and depressive (Figure 1C). Celastrol alleviated the above abnormalities in a dose‐dependent manner (Figure 1B and C). The TPH2‐positive 5‐HTergic projections, inhibited in depression conditions, were proven downregulated by COM and upregulated by celastrol in BLA (Figure 1D). The above data invite further explorations of the pharmacological procedure of celastrol in BLA.

**FIGURE 1:**
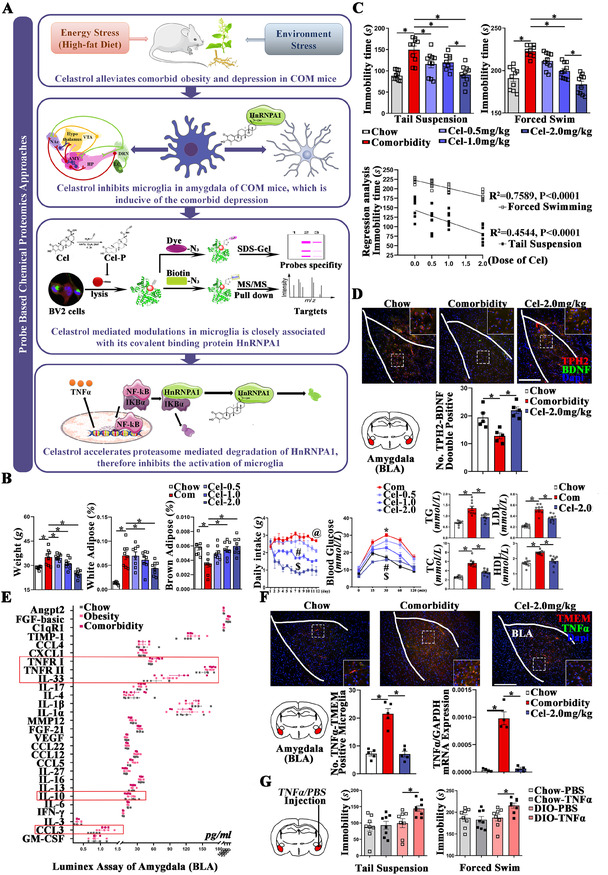
Graphical abstract and identification of amygdala as drug target of celastrol. (A) Graphical abstract. Schematic diagram of animal modeling and probe‐based chemical proteomics approach. (B) Obesity‐related symptoms quantified by weight, daily intake of food, fat rate of white/brown adipose, and quantification of TG/TC/LDL/HDL in serum. @ represents significant difference between Com and Cel‐0.5, # represents significant difference between Cel‐0.5 and Cel‐1.0, and $ represents significant difference between Cel‐1.0 and Cel‐2.0. (C) Depressive behaviors quantified by tail suspension and forced swimming tests, and correlation between dosage of celastrol and the anti‐depression effects. (D) 5‐HTergic neurons stained by TPH2 in BLA. (E) Luminex assay for inflammatory factors in BLA. (F) Quantification of TNF‐α protein by immunofluorescence staining and mRNA by RT‐PCR in BLA, bar 200 μm. (G) Depressive behaviors of mice underwent injection of purified mouse TNF‐α protein or PBS. Results are presented as mean ± SEM. *p* values are determined by one‐way ANOVA and Tukey's post tests, ^@,#,$,*^ *p* < 0.05. COM, comorbid obesity and depression; Cel, celastrol; BDNF, brain‐derived neurotrophic factor; TPH2, tryptophan hydroxylase 2; NF‐κB, nuclear factor‐κB; TNF‐α, tumor necrosis factor α; IKBα, NF‐κB inhibitor α; Cel‐P, probe‐labeled celastrol; HnRNPA1(A1), heterogeneous nuclear ribonucleoprotein A1; TG, triglyceride; TC, total cholesterol; LDL, low‐density lipoprotein cholesterol; HDL, high‐density lipoprotein cholesterol; BLA, basolateral amygdala; Chow, chow diet; DIO, diet induced obesity

Luminex assay was applied in BLA. Significant alternations were limited to M1/M2 markers of macrophage, such as TNFRI/II, IL‐33, CCL3, and IL‐10 (Figure 1E). Microglia is the macrophage in the brain. Shown by TNF‐α‐TMEM119 double staining in BLA, significant overexpression of TNF‐α in microglia was found in COM mice as compared with chow, which were alleviated by celastrol (Figure 1F). Besides, injection of purified TNF‐α protein in BLA induced behaviors of depression in diet‐induced obesity mice, but not in chow diet ones, indicating the overexpression of TNF‐α in BLA is strongly associated with comorbidity (Figure 1G).

Next, probe‐based chemical proteomics experiments were operated in BV2 cells to identify the direct binding target of celastrol^3^ (Figure 1A). Celastrol‐probe was constructed as described previously.^4^ In brief, alkyne‐linked celastrol‐probe was incubated with cell lysate. Then, biotin was added to enrich probe‐binding proteins by the biotin‐avidin system. First, 341 proteins were identified by mass spectrometry (Table S1). Second, protein–protein interaction network analysis was applied. HnRNP family, especially HnRNPA1, caught our attention (Figure 2A). As reported, HnRNPA1 mediates the proteasome‐dependent degradation of IKBα, the maximal activation of NFκB, and transcription of TNF‐α.^5^ Recently, GWAS found associations between HnRNPA1 and BMI‐adjusted waist circumference and waist–hip ratio, indicating role of HnRNPA1 in energy metabolism. Third, the direct binding relationship between celastrol and HnRNPA1 was certified by pull‐down assay, cellular thermal shift assay, and competitive binding test (Figure 2B–D), while binding affinity was measured by surface plasmon resonance test (Figure 2E).

**FIGURE 2:**
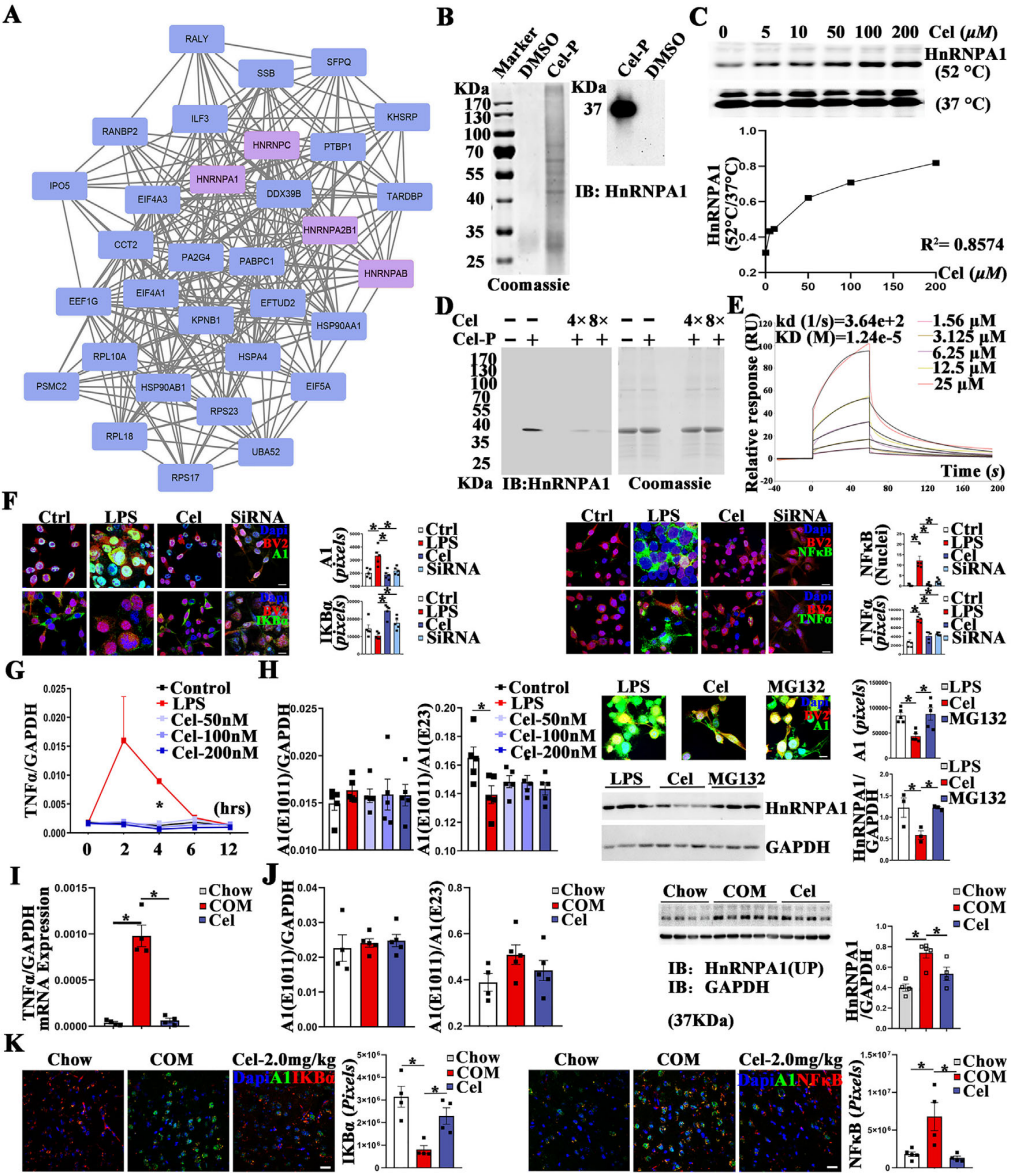
Identification of HnRNPA1 as potential drug target of celastrol. (A) PPI analysis of proteins covalently bind with celastrol. (B) Pull‐down analysis of HnRNPA1. (C) CETSA analysis of the binding between celastrol and HnRNPA1. (D) Competitive binding assay of celastrol/celastrol probe with purified HnRNPA1 protein. (E) SPR assay of gradient dosage of celastrol and purified HnRNPA1. (F) Immunofluorescence staining of HnRNPA1‐IKBα‐NFκB‐TNF‐α in BV2 cells, bar 10 μm. (G) Quantification of TNF‐α mRNA by RT‐PCR in BV2 cells. Quantification of HnRNPA1 mRNA by RT‐PCR, and HnRNPA1 protein by western blot in BV2 cells (H) and BLA (I). (K) Immunofluorescence staining of NFκB‐TNF‐α in the amygdala, bar 20 μm. Data are presented as mean ± SEM. *p* Values are determined by one‐way ANOVA and Tukey's post tests (*p < 0.05). PPI, protein–protein interaction network analysis; CETSA, cellular thermal shift assay; SPR, surface plasmon resonance tests

Then, we validated celastrol‐mediated modulation of HnRNPA1‐IKBα‐NFκB‐TNF‐α pathway in BV2 and BLA (Figure 2F, G, I, and K). By RT‐PCR and Western blot, celastrol‐mediated downregulation of HnRNPA1 in BV2 and amygdala were proven on the protein, but not mRNA level (Figure 2H and J). As shown by immunofluorescence staining and Western blot, the addition of proteasome inhibitor‐MG132 was sufficient to offset celastrol‐mediated degradation of HnRNPA1, indicating the dependence of proteasome (Figure 2H).

Furthermore, experiments were designed to validate the role HnRNPA1 assumed in the curation.

First, the protein expression level of HnRNPA1 was downregulated by adeno‐associated virus (AAV) encoding shRNA specific for HnRNPA1 in BLA of COM mice (A1‐sh‐AAV) (Figure 3A). As shown, effective inhibition of microglia and TNF‐α (Figure 3B), alleviation of obesity and depressive behaviors (Figure 3C), and reconstruction of neuropeptide Y (NPY) and TPH2 projections (Figure 3D) were achieved in A1‐sh‐AAV mice as compared to Com‐non ones. These results indicate that downregulation of HnRNPA1 is sufficient to alleviate obesity–depression comorbidity.

**FIGURE 3:**
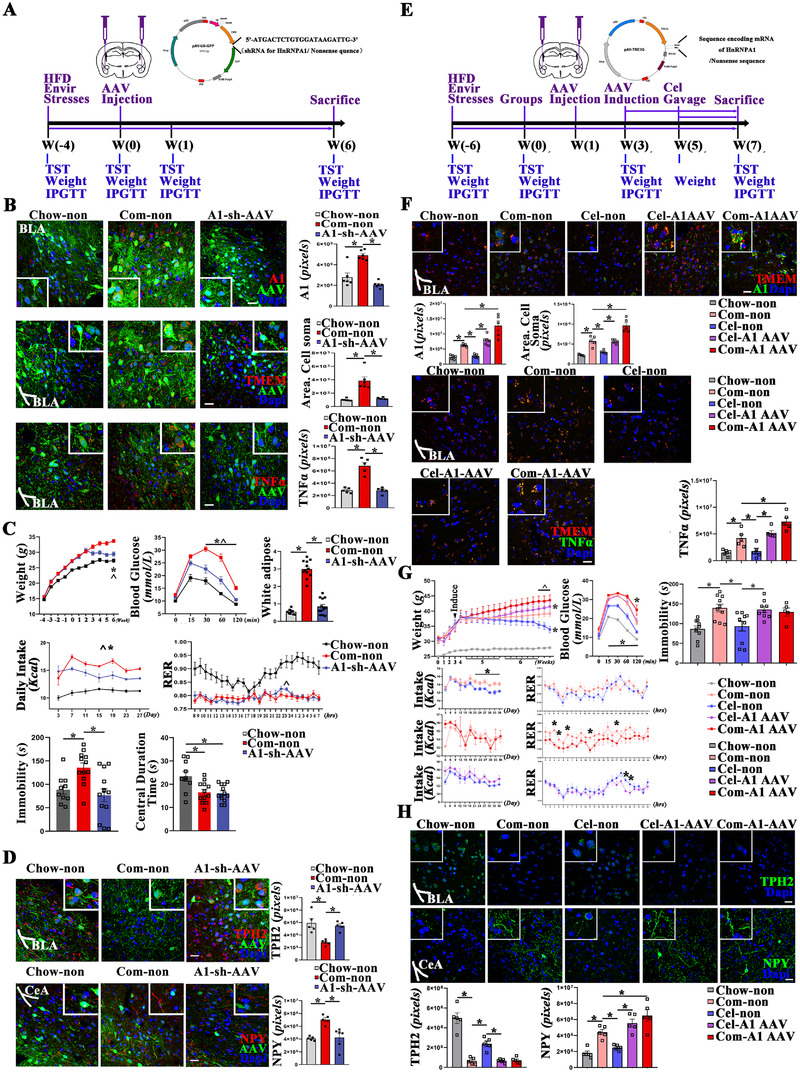
Validation of HnRNPA1 as important drug target of celastrol when dealing with comorbid obesity and depression. (A) Schematic diagram of forward experiments to validate curative effects by the downregulation of HnRNPA1. AAV was injected into BLA. In detail, AAV encoding nonsense shRNA‐GFP was injected into chow (Chow‐non) and comorbidity (Com‐non) mice; AAV encoding HnRNPA1‐shRNA‐GFP was injected into comorbidity mice (A1‐sh‐AAV). (B) Quantification of shRNA‐mediated regulation of HnRNPA1, microglia, and TNF‐α by immunofluorescence staining, bar 20 μm. (C) Quantification of shRNA‐mediated modulations of energy metabolism and depressive behaviors. (D) Quantification of shRNA‐mediated regulation of 5‐HTergic and NPY neurons in BLA, bar 20 μm. (E) Schematic diagram of reverse experiments to validate the offsets to celastrol by overexpressing HnRNPA1. In detail, AAV encoding nonsense sequence was injected into chow (Chow‐non), as well as comorbidity mice without (Com‐non)/with celastrol (Cel‐non); AAV encoding HnRNPA1 was injected into comorbidity mice without (Com‐A1‐AAV)/with celastrol (Cel‐A1‐AAV). (F) Quantifications of HnRNPA1, microglia, and TNF‐α by immunofluorescence staining, bar 20 μm. (G) Quantifications of energy metabolism and depressive behaviors. (H) Quantifications of 5‐HTergic and NPY neurons in BLA, bar 20 μm. Data are presented as mean ± SEM. *p* values are determined by one‐way ANOVA and Tukey's post tests (*p < 0.05). TST, tail suspension test; IPGTT, intraperitoneal glucose tolerance test; RER, respiratory exchange ratio

Second, HnRNPA1 was upregulated by HnRNPA1‐encoding AAV in BLA (Figure 3E). No significant difference of HnRNPA1 was detected between the Com‐non and Cel‐A1 AAV groups, indicating the overexpression of hnRNPA1 induced by AAV just offset the downregulation by celastrol. Activation of the microglia was found in Cel‐A1 AAV mice as compared with Cel‐non ones and in Com‐A1 AAV mice as compared with Com‐non (Figure 3F). Increase of weight, intolerance to glucose, and increase of immobility time in tail suspension test were observed in Cel‐A1 AAV mice as compared with Cel‐non ones (Figure 3G). In accordance with the morphological and behavioral changes, increases of TNF‐α expression and NPY positive neurons, as well as decrease in TPH2 positive neurons were found in Com‐A1‐AAV mice as compared with Chow‐non ones, and in Cel‐A1‐AAV mice as compared with Cel‐non ones (Figure 3H). These results indicate that HnRNPA1 is important in celastrol‐mediated curation of obesity‐depression comorbidity.

Metabolic cages were used to quantify energy intake and expenditure. In consistent with previous studies, celastrol may modulate energy intake rather than energy expenditure (Figures 1B and 3G). Besides, indicated by comparison between A1‐sh‐AAV and Com‐non, the downregulation of HnRNPA1 seems more likely to inhibit energy intake (Figure 3C). However, the upregulation of HnRNPA1 seems more likely to inhibit energy expenditure, indicated by comparisons between Com‐A1‐AAV and Com‐non, Cel‐A1‐AAV and Cel‐non (Figure 3G).

In general, this study demonstrates that by the direct binding with HnRNPA1, celastrol accelerates the degradation of HnRNPA1, which may explain its curation of comorbid obesity and depression in COM mice. These findings indicate that celastrol may carry therapeutic potential for this complicated comorbidity in clinical patients who are in despair. Besides, HnRNPA1 could be a potential and effective target to combat this comorbidity.

## CONFLICT OF INTEREST

The authors declare no conflict of interest.

## DATA AVAILABILITY STATEMENT

All data are presented in the letter or the supplementary materials.

## Supporting information

Supporting Information

Supporting Information

Supporting Information

Supporting Information

Supporting Information

Supporting Information
