# Supplementary material for: Celastrol alleviates comorbid obesity and depression by directly binding amygdala HnRNPA1 in a mouse model
Source: Clin Transl Med. 2021 Jun 6;11(6):e394. doi: 10.1002/ctm2.394 (PMC8181197; doi:10.1002/ctm2.394)
Supplement: Supplementary file 1 — Supporting Information [file CTM2-11-e394-s001.docx]

**2.1 Reagents and antibodies.** Rabbit anti-TPH2 primary antibody: Abcam ab184505, 1:400; Rabbit anti-NPY primary antibody: CST 11976, 1:400; Rabbit anti-TMEM119 primary antibody: Abcam ab209064, 1:400; Goat anti-TNFα primary antibody: R&D systems AF-410-NA, 1:40; Mouse anti-HnRNPA1 primary antibody: Abcam ab5832, 1:400. DyLight 488 donkey anti-goat IgG: EarthOx E032231-01 1:200; DyLight 594 donkey anti-rabbit IgG: EarthOx E032421-01 1:200; DyLight 488 goat anti-mouse IgG: EarthOx E032210-01 1:400; DyLight 594 goat anti-rabbit IgG: EarthOx E032420-01 1:400; DAPI: Solarbio, C0060; a two-step immunohistochemical staining Kit for mouse: ZSGB-BIO, PV-6002; BSA-02: EarthOx, Lot#: 812181; Triton X-100: Macklin, T824275-500ml, Lot#: C10410316, CAS: 9002-93-1; PBS: BOSTER, AR0030, Lot#: 15I01B30; Antifade Solution: Sigma, F6182-20ml; Permount TM Mounting Medium: ZSGB-BIO, ZLI-9516.

**2.2 The** **Obesity and Depression Comorbidity (COM) mice.** All Animal Experiments were supervised and approved by the Research Ethics Committee of China Academy of Chinese Medical Science, Beijing, China (permission number: 2017-006) and conducted by the trained researchers following the standards under NIH guidelines. All the results of behavioral experiments were analyzed quantitatively. The COM mice was constructed as follows: three-week-old male C57/BL6J mice were purchased from Beijing Vital River Laboratory Animal Technology Co., Ltd. and raised in SPF-grade animal house belonging to Institute of Chinese Materia Medica, China Academy of Chinese Medical Sciences. The mice, 5 in one cage, were kept in a 12-hour light and 12-hour dark interval, with free access to diet and water. Mice were randomly divided with random numbers generated by Excel 2013 software. Chow group were fed with control diet (D12450J) and the rest groups with high-fat diet (D12492) from Research Diets, Inc. The padding material of chow groups were changed twice a week, with other groups once every two weeks. Mice would be kept for a total of 16 weeks unless indicated.

**2.3 Orally administration of celastrol.** Celastrol (National Institutes for Food and Drug Control, 111946-201501, purity: 97.0%) was dissolved in dissolved in 0.5% carboxymethyl fiber vitamin sodium (CMC-Na) with ultrasonic dissolution for 2 hours (2mg in 10ml for 2mg/kg, and diluted with CMC-Na for other concentration groups), and used for gavage at 0.1ml/10g body weight. Mice in chow group and COM group would be gavaged with the same volume of CMC-Na. The time point of gavage was set at 12 noon. Food intake in the past 24 hours and body weight would be weighed just before gavage.

**2.4 Glucose Tolerance Tests.** Mice were fasted between 8:00 a.m. and 2:00 p.m., meanwhile free to water. Tail-tip blood was used to measure blood glucose with Accu-Chek Active test strips (Roche Diabetes Care GmbH) for 0 min at 2 p.m. For the next 15 minutes, mice were intraperitoneally injected with 20% glucose solution (dissolved with PBS) at 0.1 ml per 10 g body weight. Then the blood glucose was measured at 15 min, 30 min, 60 min and 120 min after intraperitoneal injection respectively.

**2.5 Forced Swimming Test.** The forced swimming tests were conducted 4hrs after the gavage on the testing days as described in the previous studies^1^. Before the test, the mice were acclimated to the test room for 1hr. In brief, the tests were conducted in a glass cylinder with 19cm in height and 14cm in diameter containing 1000ml water at 25 ±1 °C. In the 6-minute test, the duration time of the immobility and passive swimming would be measured in the last 4min.

**2.6 Tail Suspension Test.** The tail suspension tests were conducted 4hrs after the gavage on the testing days as described in the previous studies^2^. Before the test, the mice were acclimated to the test room for 1hr. In brief, the tests were performed in a suspension box (55 cm height, 60 cm width, 11.5 cm depth). In the separated compartments (15 cm width/mouse), four mice were tested at the same time. During the 6-minute test, the immobility time was recorded and analyzed in the last 4min.

**2.7 Open Field Test.** The open field tests were conducted 4hrs after the gavage on the testing days as described in the previous studies^3^. Before the test, the mice were acclimated to the test room for 1hr. Brieﬂy, a square black test cage 50 * 50 cm, with a wall 30 cm high and a border region 8 cm wide was used. At the beginning, each one was placed in the border region facing the wall. During the 4-minute test, the total duration time of mice staying in the central region would be measured.

**2.8 Mice execution and tissue preservation.** The mice were anesthetized with isoflurane and killed by cutting off the neck. Blood was collected and kept for 30 minutes at room temperature. Then the blood would be centrifuged with 1000g for 10 min at room temperature to obtain upper serum, which would be stored at -80℃ for subsequent testing. The mouse brain would be dissected with flat head tweezers, fixed with 4% paraformaldehyde, dehydrated with 8%, 15% and 30% sucrose solution sequentially, and frozen at -80℃ until frozen sections were made. In addition, the brains of mice were frozen in liquid nitrogen immediately after dissection and then transferred to -80℃ for storage. After the amygdala was extracted after frozen section (-23℃), the subsequent RNA (transzol up plus RNA kit, er501-01, transgene) or protein (minute, total protein extraction kit for animal packed cells and tissues, cat No. SD-001 / sn-002) extraction steps were immediately performed. Perirenal, mesenteric and epididymis white adipose tissue, and brown adipose tissue from the nape back would all be removed and weighed.

**2.9 Liver and kidney function and blood lipids test**. The blood lipid levels (triglyceride, total cholesterol, high density lipoprotein cholesterol, and low-density lipoprotein cholesterol), and liver (glutamic-pyruvic transaminase, glutamic-oxalacetic transaminease) and kidney function (creatinine) were detected in serum by AU680 automatic biochemical analysis system (Beckman Coulter, Indianapolis, IN). The specific operation method was carried out according to the instructions.

**2.10 Immuno-staining in brain tissue.** The coronal frozen sections of brain were conducted at 18μm per slice, antigen repaired with sodium citrate antigen repair solution, epitopes being blocked by BSAT (0.5% Triton X-100 in BSA), and stained with BSAT-dissolved primary antibodies overnight. Then, slices were washed with PBST (0.5% Triton X-100 in PBS) for 5 min, 3 times. For fluorescent staining, slices would be incubated with BSAT-dissolved fluorescent secondary antibodies for 30 min at room temperature away from light. For Immunohistochemical staining, slices would be incubated with BSAT-dissolved secondary antibody for 30 min at room temperature, followed by DAB staining for 5 min. Lastly, slices would be attached to glass slide, covered with Antifade Solution or Permount TM Mounting Medium followed by coverslip, and shot at 200× field of vision with sample information sealed and renumbered.

**2.11 RT-PCR.** The total RNA was extracted from the BV2 cells or the Amygdala tissue (TransZol Up Plus RNA Kit, ER501-01, Transgene), reverse transcripted (Transcript One-Step gDNA Removel and cDNA Synthesis SuperMix, AT311-03, Transgene) and then qualified by qRT-PCR using SYBR GREEN method (TransStart Green qPCR SuperMix UDG, AQ111, Transgene). Primers for SYBR GREEN method were designed as follows: for GAPDH, F-5'-TGTGTCCGTCGTGGATCTGA-3', R-5'-TTGCTGTTGAAGTCGCAGGAG-3';For TNFα, F-5'-CCACCACGCTCTTCTGTCT

AC-3', R-5'-AGGGTCTGGGCCATAGACT-3'; For HnRNPA1-E23, F-5'-AGACTG

TGTGGTAATGAGAG-3', R-5'- CTCTTAGGTTCCACAAC-3'; For HnRNPA1-E1011, F-5'- CATACAGCCAGGAAACAAAG -3', R-5'- CATGTCTTCTTTGTAGCA

GC-3'.The reaction procedure of qRT-PCR would be as: 95℃ 10min for predenaturing; 95℃ 5s for denaturing, 60℃ 1min for annealing and extending, and run for 40 cycles. Results would be analyzed by 2^-△CT^ method.

**2.12 Western Blot.** For western blotting analysis, amygdala tissues were homogenized in RIPA lysis buffer, which contained 50 mmol/L Tris-HCl, 1% Triton X-100, 0.1% sodium deoxycholate, 5 mmol/L EGTA, 5 mmol/L EDTA, 150 mmol/L NaCl, 40 mmol/L NaF, 2.175 mmol/L sodium orthovanadate, 0.1% SDS, 0.1% aprotinin, and 1 mmol/L phenylmethylsulfonyl fluoride, (pH = 7.2). Then, the tissue or cell homogenate was centrifuged at 12 000 g (4℃) for 15min, and the supernatant was collected as protein extracts. Next, a BCA protein assay kit (Thermo Fisher Scientific) was used to measure the protein concentration. After the total proteins were extracted and quantified, approximately 50 μg of total proteins were loaded in an SDS/PAGE gel and were transferred to polvinylidene fluoride membranes. Subsequently, 5% none-fat dry milk with TBS containing 0.1% Tween-20 was used to block the nonspecific protein binding sites. After that, the bands containing target proteins were incubated with primary antibodies overnight at 4℃. The following day, all bands were washed with TBS containing 0.1% Tween-20 three times for 10min and incubated with secondary antibodies conjugated to IRDye 800CW for 50 min. Finally, the bands containing target proteins were measured and quantified by the Fusion imaging system (Fusion-FX6.EDGE V.070, France). Protein expression levels were normalized to the GAPDH internal control.

**2.13 Luminex inflammatory factors assay**

The total protein was extracted from the Amygdala tissue. The BIO-RAD LXSAMSM-36 chip was used, which including GM-CSF, CXCL1/GRO, alpha/KC/CINC-1, TNF-alpha, TNF RI/TNFRSF1A, CCL2/JE/MCP-1, IL-1 beta/IL-1F2, CXCL2/GRO beta/MIP-2/CINC-3, VEGF, IL-2, IL-4, IL-5, IL-6, IL-10, IL-13, IL-17/IL-17A, IFN-gamma, IL-3 , IL-16, CCL5/RANTES, G-CSF, CCL12/MCP-5, IL-33, TIMP-1, CCL3/MIP-1 alpha, IL-1 alpha/IL-1F1, CCL20/MIP-3 alpha, CCL4/MIP-1 beta, IL-27, FGF-21, C1qR1/CD93, FGF basic/FGF2/bFGF, Angiopoietin-2, MMP-12, TNF RII/TNFRSF1B, CCL22/MDC, RAGE/AGER.

**2.14 BV2 cell culture.** In this study, the BV2-TdT cells (National Infrastructure of Cell Line Resource) were used for the Immuno-staining, qRT-PCR, chemical proteomics, pull-down experiment, and competitive binding assay. The cells grew in DMEM high glucose medium containing 10% FBS and 1% penicillin/streptomycin, with the temperature being 37 ℃, the CO_2_ concentration being 5%, and the humidity being saturated. Cells were plated on the glass covers (Fisher FIS 12-545-82) in 24-well plates (Corning, 3524) for Immuno-staining, in 6-well plates (Corning, 3516) for qRT-PCR, and directly in 100mm culture dishes (Corning, 430167) for other tests. In the experiments in 24-well and 6-well plates, 0/50/100/200 nM CEL (CEL dissolved in DMSO at 0/50/100/200 μM, then dissolved in DMEM) were given on the beginning of the 4hr-lipopolysaccharide (LPS, 1μg/ ml, L4516, sigma)-induced activation of BV2 cells. In 100mm culture dishes, cells were incubated only with LPS, but no CEL. After the inductions, cells plated on the glass covers were fixed for Immunofluorescence assay, cells plated in the 6-well plates were collected for the extraction of total RNA, and cells plated directly in the 10cm culture dishes were collected for the extraction of whole protein.

**2.15 Cell immunofluorescence assay.** After the inductions, cells plated on the glass covers were fixed with 4% paraformaldehyde (Solarbio, Cat#P1110) for 30min, epitopes being blocked by BSAT, stained with BSAT-dissolved primary antibodies overnight, and incubated with BSAT-dissolved fluorescent secondary antibodies for 60 min and DAPI for 5min at room temperature away from light. Lastly, glass covers containing cells would be attached to glass slide, and shot at 100× field of vision with Olympus FV1000 confocal microscope, while sample information were sealed and renumbered.

**2.16 Chemical proteomics.** This part was operated by following our previous protocol^4^. In short, a alkyne was added to the celastrol molecule to produce celasrol-probe. At the same time, BV2 cells after 4h’s LPS intervention were lysed and incubated with celastrol-probe for 4h at room temperature. Then, affinity tag-biotin was added to the probe, making proteins covalently binding with celastrol-probe can be enriched by the biotin-avidin system. The covalent binding proteins were identified with mass spectrometry (MS/MS).

**2.17 Protein-protein interaction network construction.** STRING 11.0^5^ was used to construct the interaction among direct targets of celastrol. Cytoscape 3.8.0^6^ and Mcode plug-in were used to analyse highly correlated gene groups.

**2.18 Pull-down assay.** Proteins binding with celastrol-probe were enriched by biotin affinity chromatography as described in previous studies^7^. SDS-PAGE and Westernblot were applied to identify proteins. In this experiment, 341 proteins were enriched by the celastrol-probe and identified by SDS-PAGE-MS/MS. HnRNPA1 was identified by the WB using anti-HnRNPA1 (mouse, dissolved in BSA at 1:1000, ab5832, abcam), and the secondary antibodies HRP goat anti-mouse (dissolved in BSA at 1:10000, E030110-01, Earthox).

**2.19 Competitive binding test.** In this part, same amount of purified mouse HnRNPA1 protein were incubated with celastol-probe with/without the pretreatment with 1μM, 4μM and 8μM celastrol. The proteins would be analyzed by SDS-PAGE and WB. HnRNPA1 was identified by the WB using anti-HnRNPA1 (mouse, dissolved in BSA at 1:1000, ab5832, abcam), and the secondary antibodies HRP goat anti-mouse (dissolved in BSA at 1:10000, E030110-01, Earthox).

**2.20 Cellular Thermal Shift Assay (CETSA).** Typically, BV2 cells (approximately 3.2 × 107) were collected, lysated and centrifuged at 15 000g for 20 min at 4 °C. Then, supernatants were divided equally into tubes and ncubated with celastrol (5-10-50-100-200μM) at RT for 30min. Then, tubes were incubated at 37/52°C for another 3min, centrifuged at 20 000g for 20 min at 4 °C. Supernatants were analyzed by Western blot.

HnRNPA1 was identified by anti-HnRNPA1 (1:1000, ab5832, abcam), and HRP goat anti-mouse (1:10000, E030110-01, Earthox). Tubulin was identified by anti-tubulin (1;5000, ab210797), HRP goat anti-rabbit (1:10000, E030110-02, Earthox).

**2.21 Surface plasmon resonance (SPR) detection.** To confirm the binding affinity of celastrol and HnRNPA1, SPR assay was applied. Proteins were immobilized on CM5 sensor chips by an amine-coupling procedure on flow cells channel 1~4, and flow cell 1 was set as a reference control. 0.4M 1-ethyl-3-(3-dimethyla-minopropyl carbodiimide (EDC) in H2O and 0.1M N-hydroxysuccinimide (NHS) in H2O at the ratio of 1:1 (v:v) were injected at a flow of 10µL/min for 420s for activating the carbonyl group. Purified HnRNPA1 protein was dissolved in sodium acetate acid buffer (pH 4.5) at a final concentration of 40 μg/mL with the injection time of 300s. Finally, all the channels were blocked with ethanolamine (1M) at a flow rate of 10uL/min for 420s. Sensor chips were primed twice with degassed physiological running buffer (pH 7.4), which were composed with 0.02M HEPES (Ph7.4), 2.7mM KCl, 137mM NaCl, 0.05% Surfactant P20 and 5%DMSO, and equilibrated at 30uL/min until the baseline remained stable. Celastrol was dissolved in DMSO, and diluted to a concentration series using HBS-EP (0.2 M HEPES pH7.4, 2.7mM KCl, 137mM NaCl, 0.05%P20, 3mM EDTA, 5%DMSO), and then injected. During the whole process of the experiment, the analysis was conducted at 25℃, defining an association time of 60s and a dissociation time of 120s at a flow of 30uL/min. The Biacore 8K evaluation software 2.0 (GE Healthcare) was used for calculating the related KD values.

**2.21 AAV virus.** HnRNPA1 shRNA or the nonsense shRNA after the CMV promoter (The shRNA encoding the HnRNPA1 siRNA, ATGACTCTGTGGATAAGATTGTGACAATCTTATCCACAGAGTCATTTTTTT, was inserted into the pAV-U6-GFP plasmid to construct AAV9 (Vigene Biosciences, Beijing); Sequence encoding mouse HnRNPA1 was inserted into the Tet-On systems to construct the AAV9, in which the overexpression of HnRNPA1 was induced by drinking distilled water containing 600 mg/L doxycycline and 40 g/L sucrose. The induction period lasted for 14 days, with drinking water changed every two days.

**2.22 Stereotactic Injections.** To inject purified TNFα protein to amygdala for consecutive 7 days, the micro drug delivery trocars were fixed at AP: -1.2 mm, R: ±2.8 mm, DV: -3.9 mm relative to bregma and dural surfaces one week before the real injections. Mice were randomly divided into 4 groups with random numbers generated by Excel 2013 software, and 10 in each group. Then, TNFα (1ng TNFα resolved in 2μl normal saline daily, recombinant mouse TNFα, ab157351) or equal volume normal saline was inserted slowly through the micro drug delivery trocars for the consecutive 7 days. The mice were anesthetized with isoflurane during the whole operation procedure. Penicillin was used to prevent infection before the surgical opening had been sutured. The mice were placed in cages alone after the operation, and not returned to the original cages until they were awake and recovered.

**2.23 Statistical analysis.** ImageJ was used to merge fluorescence images and to analyze the signal strength of fluorescence and histochemical images. GraphPad Prism 8.0.1 was used and all the data in this study were expressed as means ± SEM. Difference among multiple groups were analyzed by the one-way ANOVA and Tukey posttest, while differences between two groups were analyzed by the two-tailed t test. The correlations between fat and body weight, the expression of HnRNPA1 and TNFα in the amygdala of mice were analyzed by the linear regression and two-tailed correlation analysis. Significant differences were examined statistically as indicated (**P* < 0.05, ***P* < 0.01, ****P* < 0.001,).

1. Petit-Demouliere B, Chenu F, Bourin M. Forced swimming test in mice: a review of antidepressant activity. *Psychopharmacology (Berl).* 2005;177(3):245-255.

2. Stukalin Y, Lan A, Einat H. Revisiting the validity of the mouse tail suspension test: Systematic review and meta-analysis of the effects of prototypic antidepressants. *Neurosci Biobehav Rev.* 2020;112:39-47.

3. Zhu C, Xu Q, Mao Z, Lin N. The Chinese Medicine Wu-Tou Decoction Relieves Neuropathic Pain by Inhibiting Hippocampal Microglia Activation. *Sci Rep.* 2018;8(1):12292.

4. Wang J, Zhang CJ, Chia WN, et al. Haem-activated promiscuous targeting of artemisinin in Plasmodium falciparum. *Nature communications.* 2015;6:10111.

5. Szklarczyk D, Gable AL, Lyon D, et al. STRING v11: protein-protein association networks with increased coverage, supporting functional discovery in genome-wide experimental datasets. *Nucleic acids research.* 2019;47(D1):D607-d613.

6. Shannon P, Markiel A, Ozier O, et al. Cytoscape: a software environment for integrated models of biomolecular interaction networks. *Genome Res.* 2003;13(11):2498-2504.

7. Wang J, Zhang J, Shi Y, et al. Mechanistic Investigation of the Specific Anticancer Property of Artemisinin and Its Combination with Aminolevulinic Acid for Enhanced Anticolorectal Cancer Activity. *ACS Cent Sci.* 2017;3(7):743-750.
