# Supplementary material for: Celastrol alleviates comorbid obesity and depression by directly binding amygdala HnRNPA1 in a mouse model
Source: Clin Transl Med. 2021 Jun 6;11(6):e394. doi: 10.1002/ctm2.394 (PMC8181197; doi:10.1002/ctm2.394)
Supplement: Supplementary file 2 — Supporting Information [file CTM2-11-e394-s006.doc]

**Raw Pictures For Westernblot**

**Figure 2B**

**Pull-down by SDS-PAGE**

**
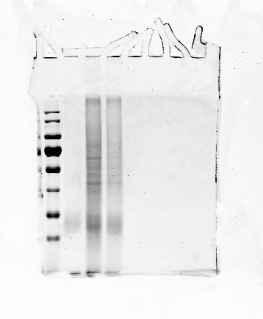
**

**Pull-down by Westernblot**


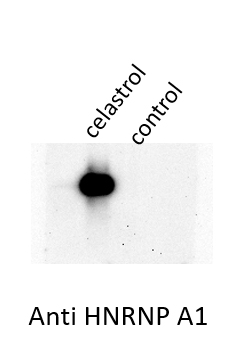

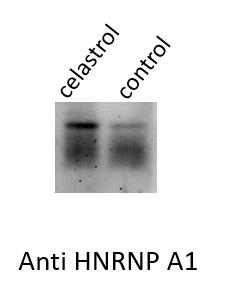


**Figure 2C**

**Repeat-1**


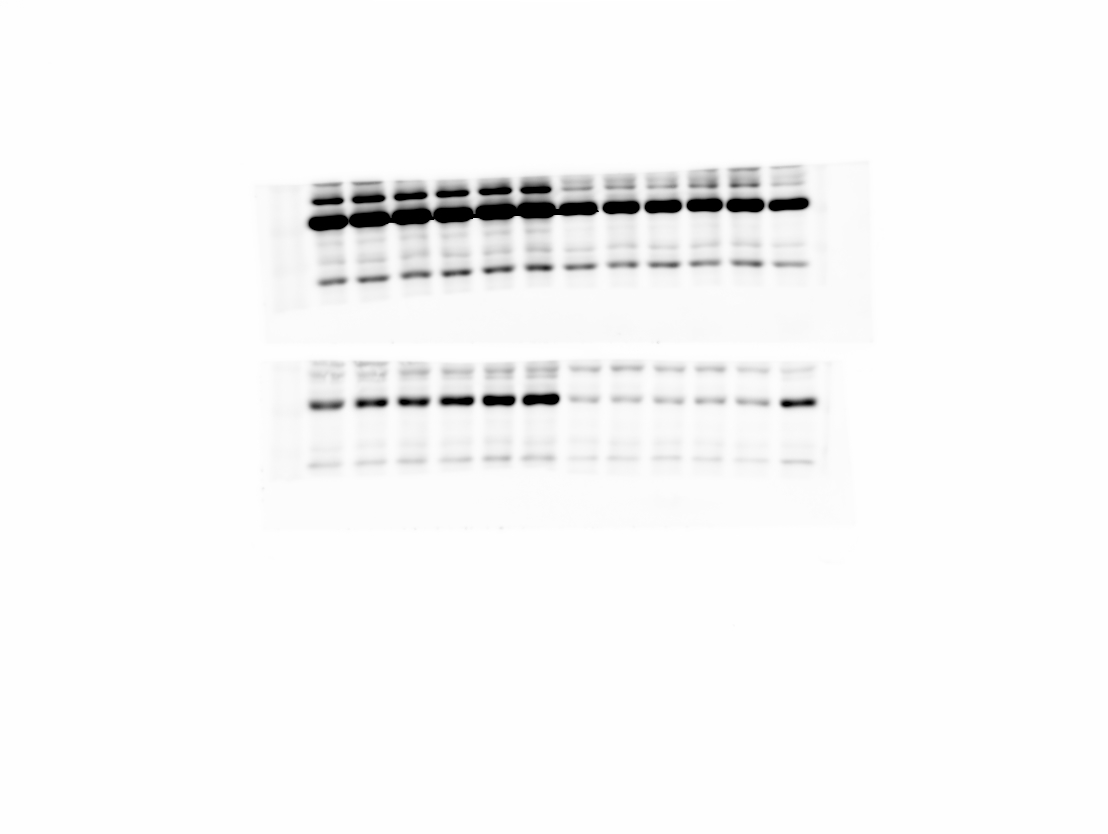

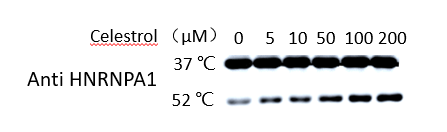


**Repeat-2**

**
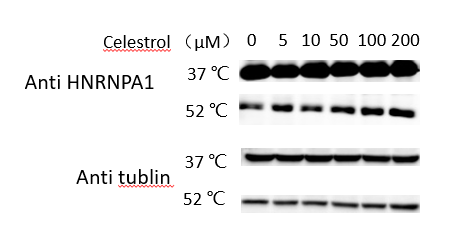
**

**Repeat-3**

**
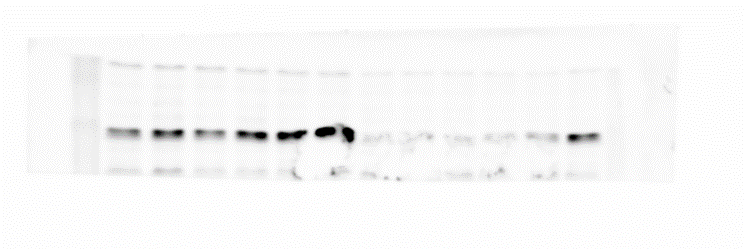

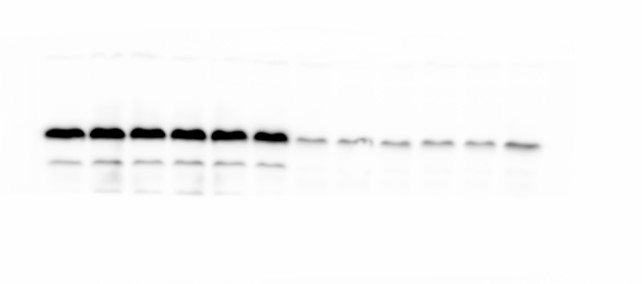
**

**
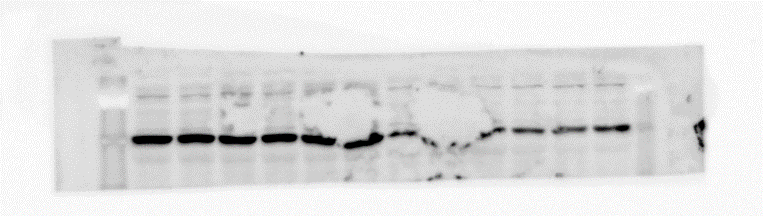

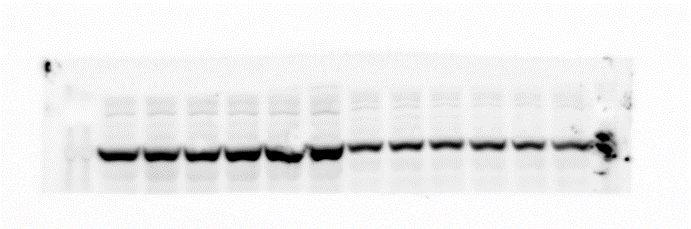
**

**
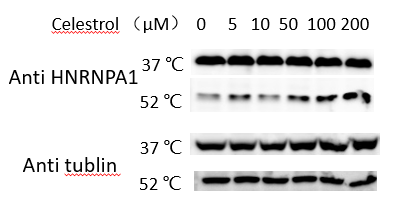
**

**Figure 2D**

**SDS-PAGE(Coomassie) Westernblot**


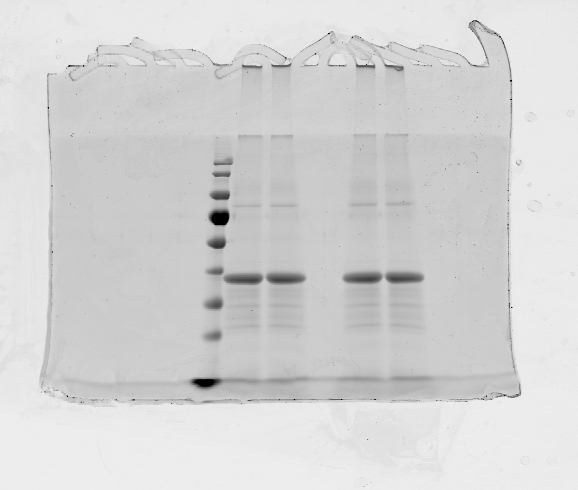

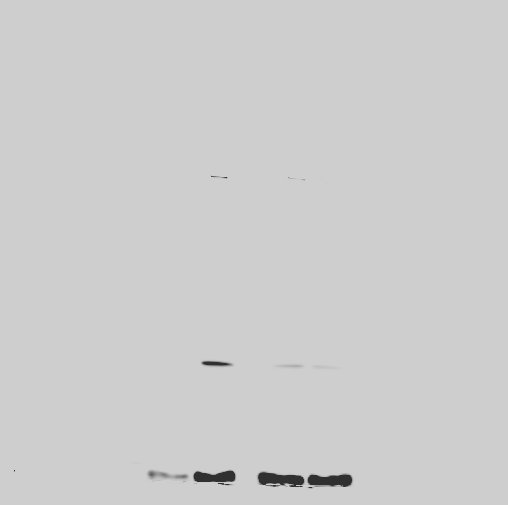


**Figure 2H**

**Repeat1 (LPS-3/Cel-3/MG132-3)**

**HnRNPA1 GAPDH**

**Repeat2 (Ctrl-2/LPS-2/Cel-2/MG132-2)**

**HnRNPA1 GAPDH**

**Repeat1 (LPS-3/Cel-3/MG132-3)**

**HnRNPA1 GAPDH**

**Figure 2J**

**Repeat1**

**HnRNPA1 GAPDH**

**
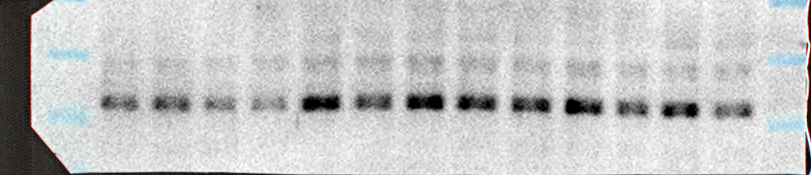

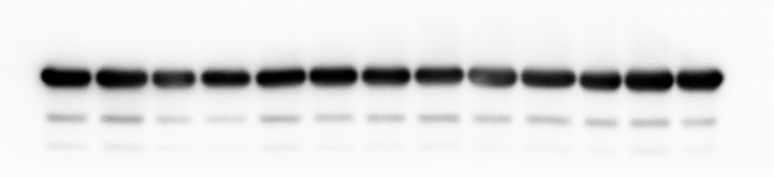
**

**Repeat2**

**HnRNPA1 GAPDH**


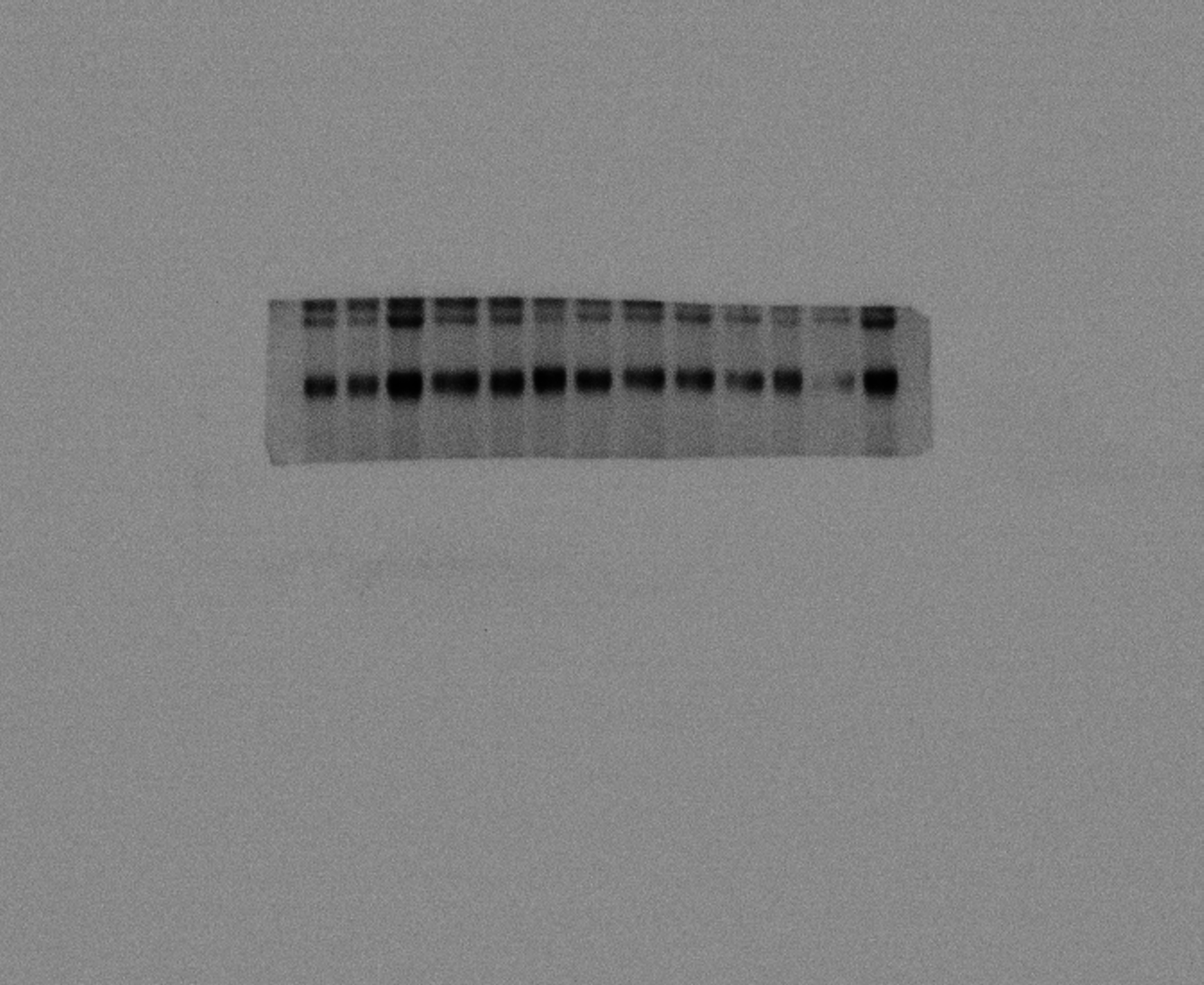

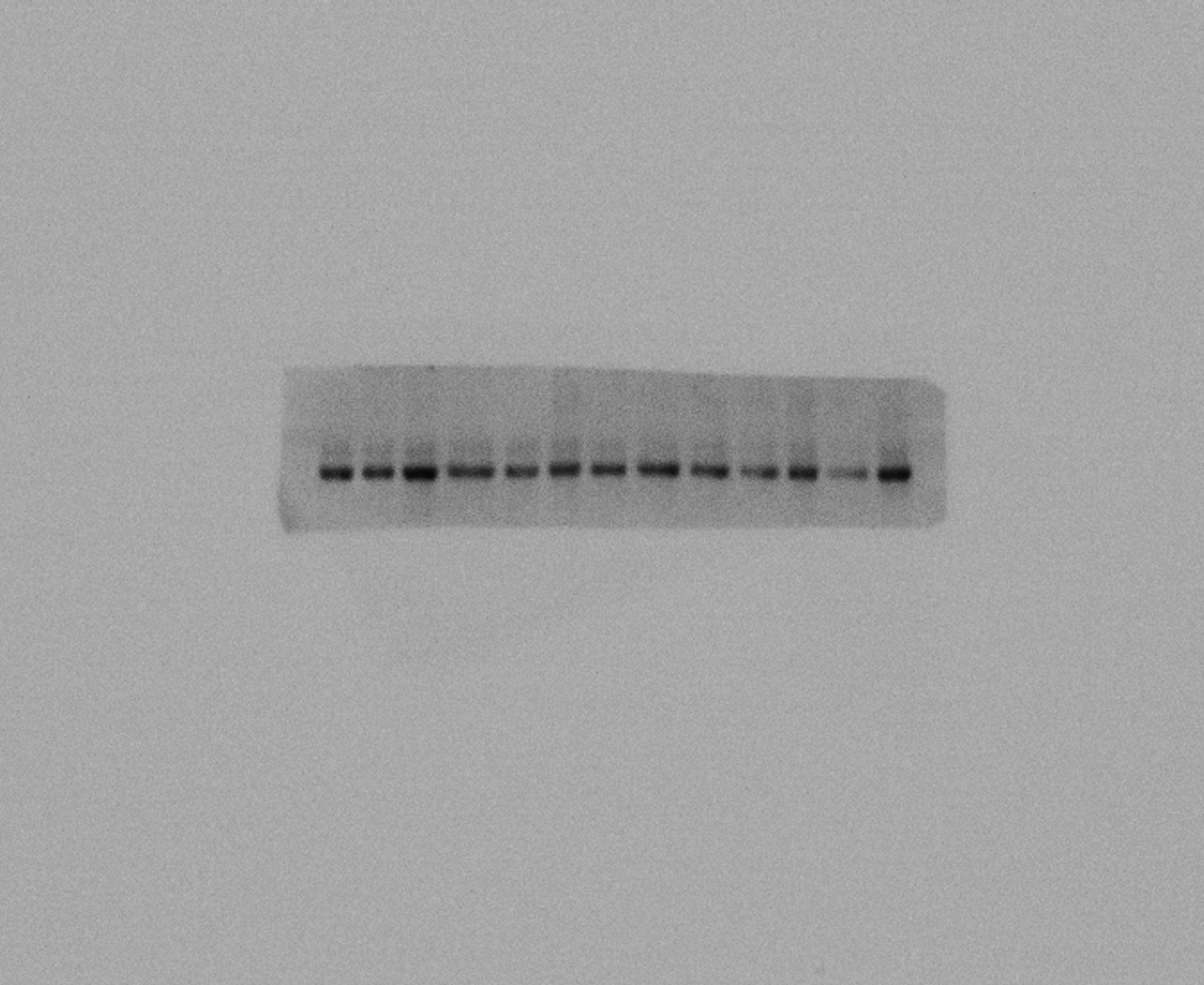


**Repeat3**

**HnRNPA1 GAPDH**

**
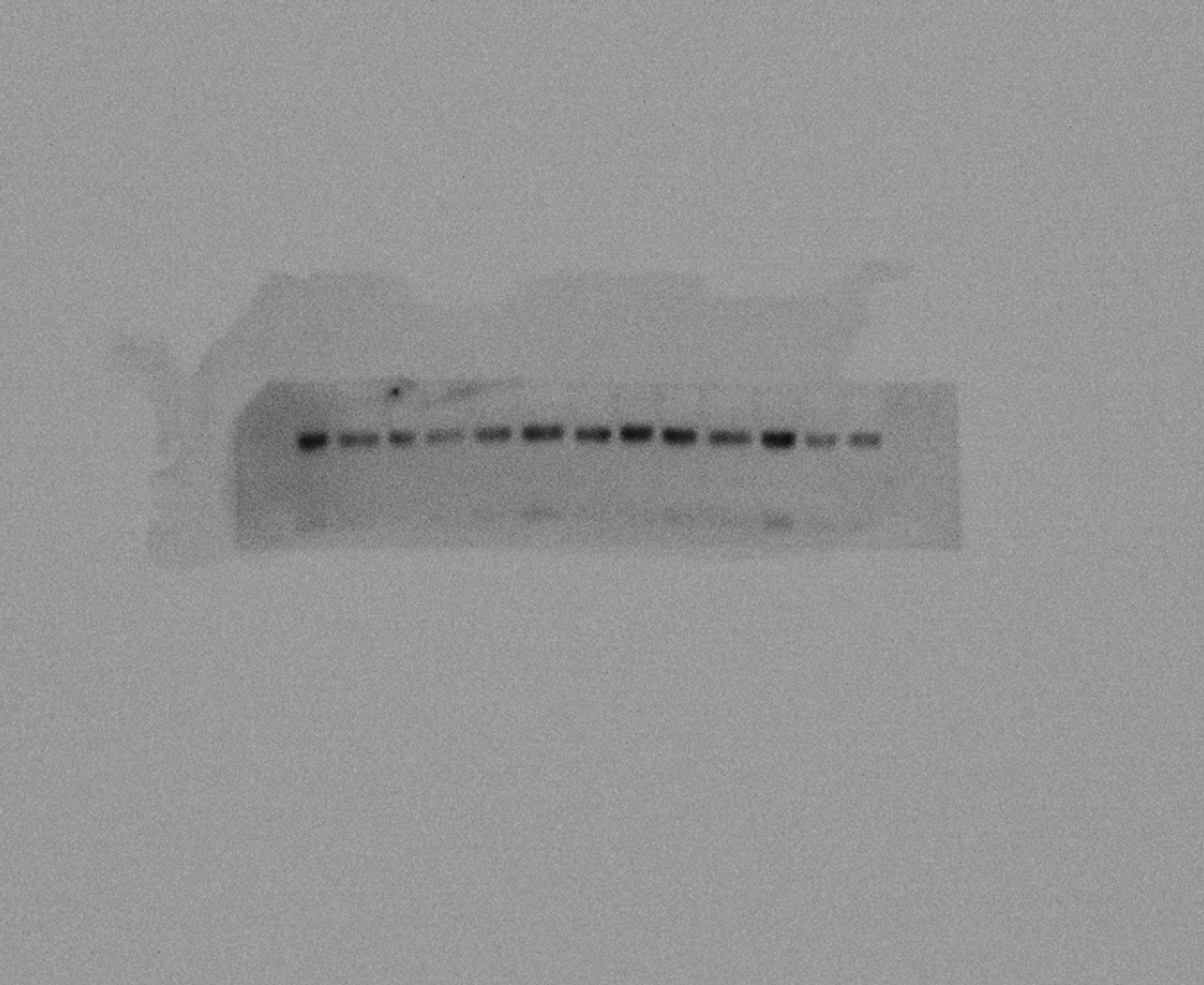

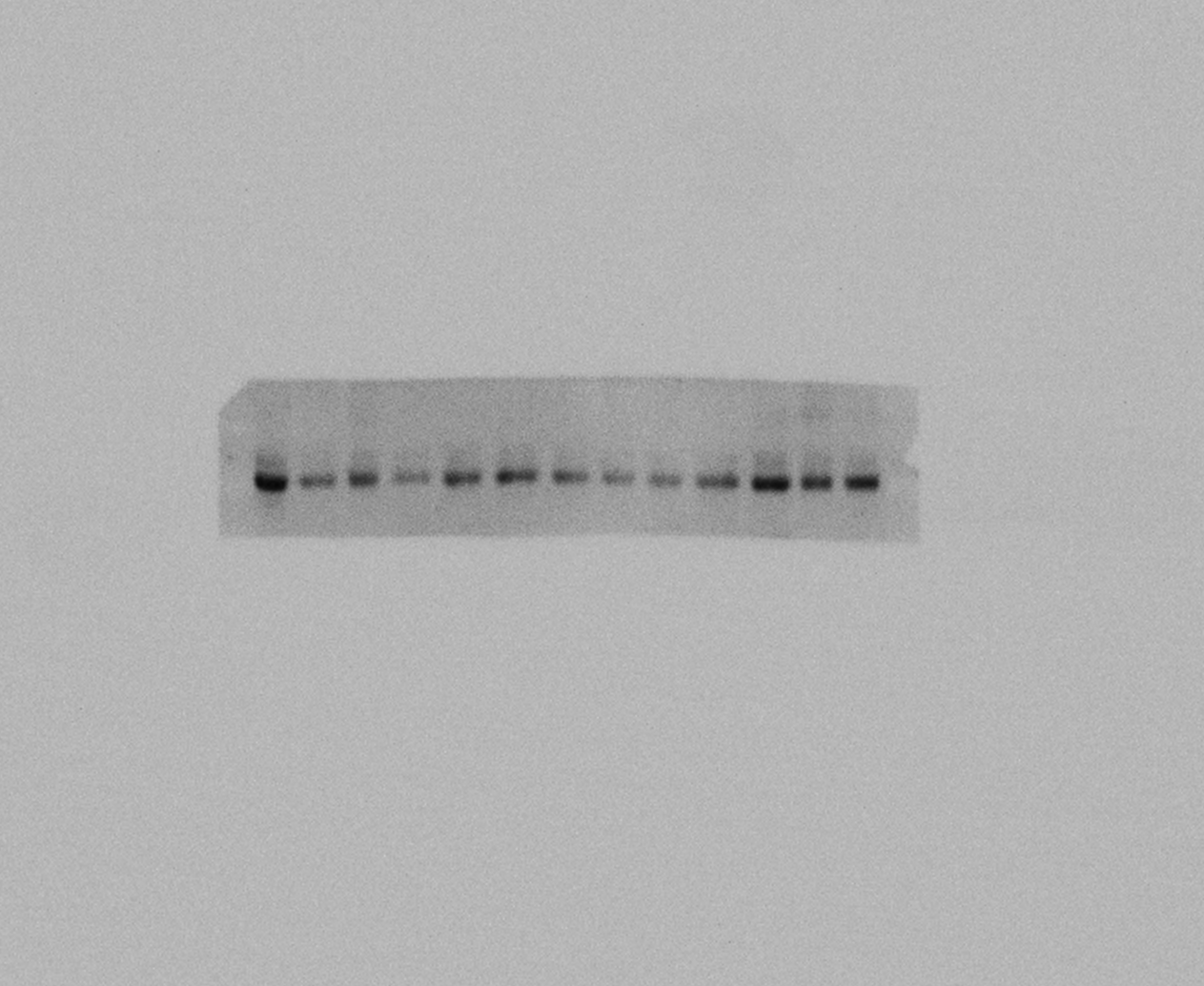
**

**Repeat4**

**HnRNPA1 GAPDH**

**
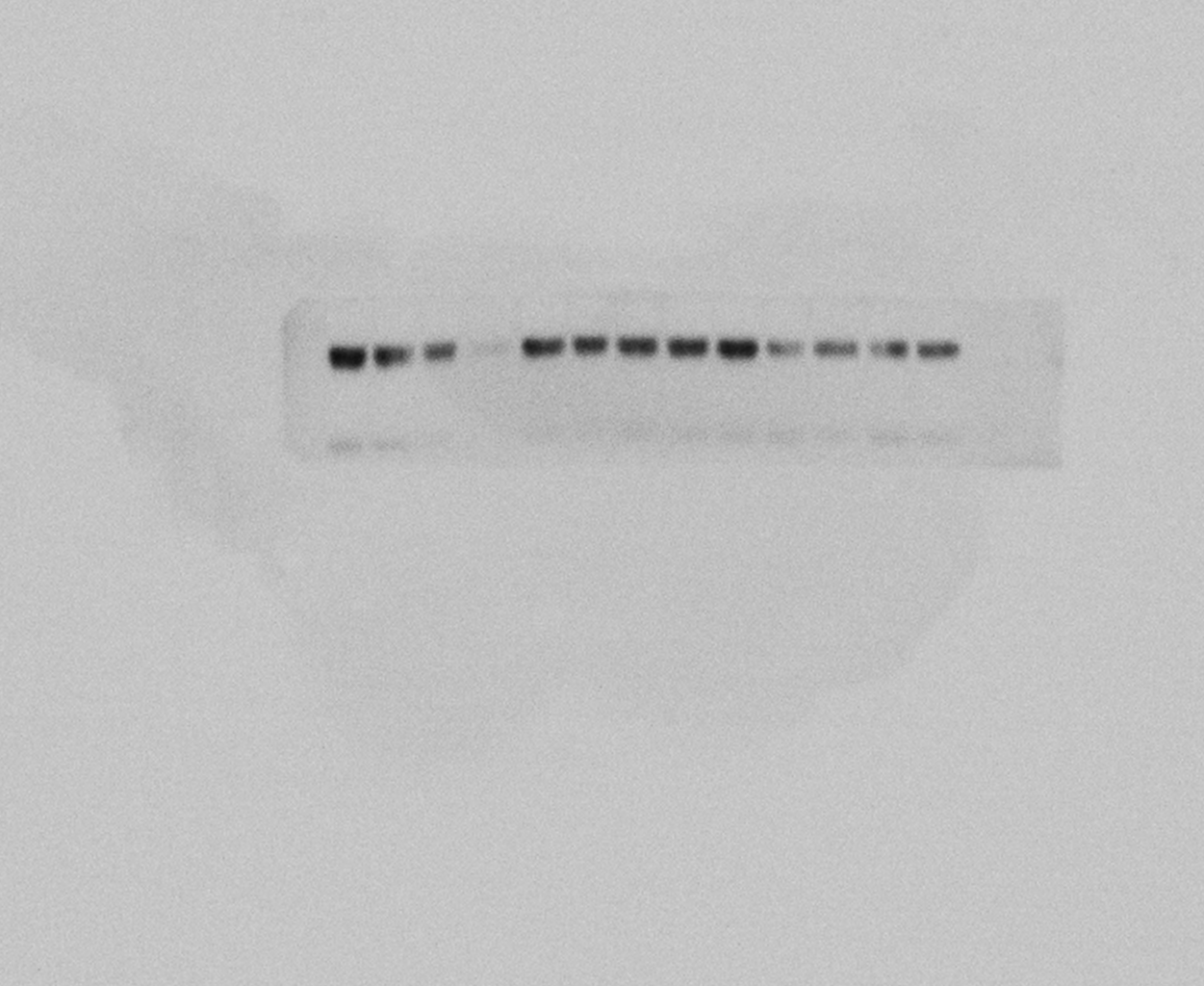

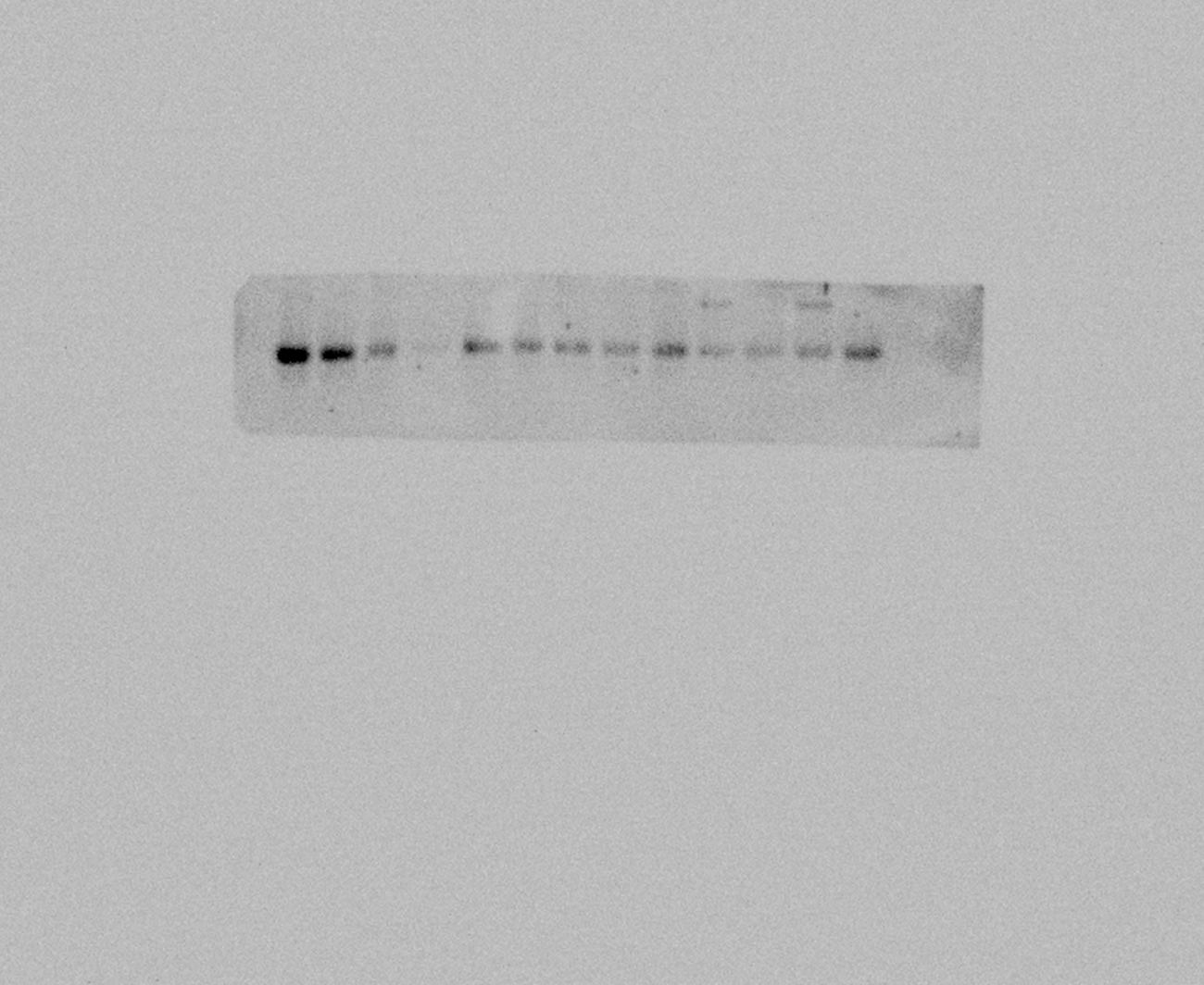
**
