# Supplementary material for: Celastrol alleviates comorbid obesity and depression by directly binding amygdala HnRNPA1 in a mouse model
Source: Clin Transl Med. 2021 Jun 6;11(6):e394. doi: 10.1002/ctm2.394 (PMC8181197; doi:10.1002/ctm2.394)

**Supplementary Figures**

**Legends:**

**Fig S1. CEL induce no damage on liver and kidney function.** Mice under diet-environment double pressures (COM) were daily treated with vehicle or celastrol (0.5, 1.0, 2.0 mg/kg) by gavage for 12 consecutive days (n=8 mice/group). A: Serum ALT; B: Serum AST; C: Serum Cr. Error bars are presented as mean ± SEM. *P* values are determined by one-way ANOVA and Tukey post tests (*P* values were all above 0.05)

**Fig S2. The CEL mediated regulation of HnRNPA1 protein expression in BLA of mice by Immunohistochemistry.** Mice under diet-environment double pressures (COM) were daily treated with vehicle or celastrol (2.0 mg/kg) by gavage for 12 consecutive days. HnRNPA1-positive cells were calculated. Results are presented as mean ± SEM. One-way ANOVA and Tukey post tests were used (**P* < 0.05).

**Fig S1**


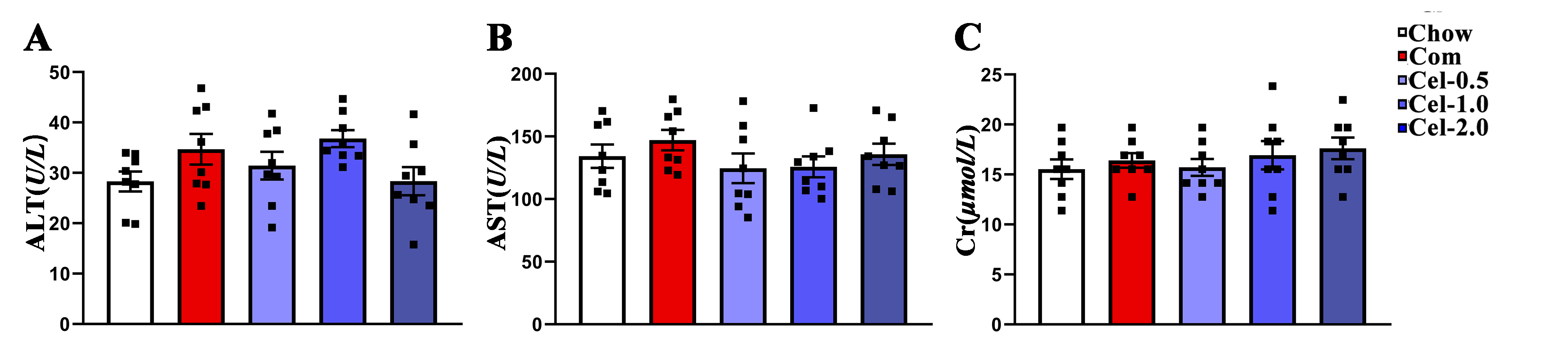


**Fig S2**


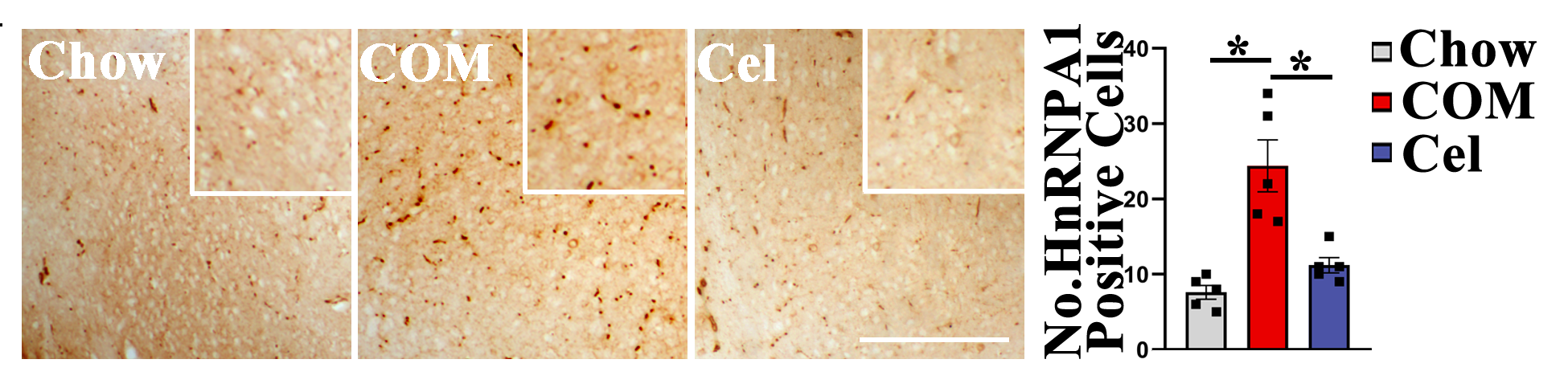

Supplement: Supplementary file 3 — Supporting Information [file CTM2-11-e394-s003.doc]
