# Supplementary material for: Celastrol alleviates comorbid obesity and depression by directly binding amygdala HnRNPA1 in a mouse model
Source: Clin Transl Med. 2021 Jun 6;11(6):e394. doi: 10.1002/ctm2.394 (PMC8181197; doi:10.1002/ctm2.394)
Supplement: Supplementary file 4 — Supporting Information [file CTM2-11-e394-s002.pdf]

Fig S1

ALT				
chow	HFD	0.5mg	1mg	2mg
28.22	36.14	29.71	31.14	29.43
27.79	30.16	19.15	36.02	30.31
19.86	27.67	23.42	33.5	41.63
30.41	27.91	38.45	44.67	25.66
32.26	23.43	41.79	34.45	35.68
20.11	42.37	37.79	42.31	15.78
33.98	43.09	31.98	33.4	23.54
33.75	46.8	29.27	38.9	24.72

AST				
chow	HFD	0.5mg	1mg	2mg
113.51	154.19	94.08	133.85	126.66
104.59	170.04	104.59	114.4	127.09
170.4	165.74	147.54	110.01	106.32
134.8	179.71	85.2	129.64	146.74
161.71	133.18	178.17	100.18	107.93
159.3	131.39	124.6	106.98	134.2
123.25	119.38	158.65	138.19	170.98
106.15	122.89	103.88	172.78	165.46

Cr				
chow	HFD	0.5mg	1mg	2mg
16.92	18.31	15.54	15.54	15.54
19.69	15.54	12.77	18.31	16.92
15.54	16.92	14.15	12.77	19.69
18.31	15.54	18.31	15.54	19.69
12.77	15.54	14.15	11.38	18.31
14.15	12.77	14.15	19.69	22.46
11.38	16.92	19.69	23.85	12.77
15.54	19.69	16.92	18.31	15.54

Fig S2

No. HnRNPA1		
Chow	Com	Cel
6	17	15
10	31	10
8	18	11
9	22	9
5	34	11
