# Supplementary material for: Celastrol alleviates comorbid obesity and depression by directly binding amygdala HnRNPA1 in a mouse model
Source: Clin Transl Med. 2021 Jun 6;11(6):e394. doi: 10.1002/ctm2.394 (PMC8181197; doi:10.1002/ctm2.394)
Supplement: Supplementary file 5 — Supporting Information [file CTM2-11-e394-s004.pdf]

Fig1. B

Weight (g)

Chow	Com	Cel-0.5	Cel-1.0	Cel-2.0
29	39	37	30.9	25.2
28.5	29.9	32.4	29.2	26.8
29	43	29.6	25.9	27.7
29.3	30.6	36.3	28.3	24.1
28.7	36.6	33.9	34.6	23.1
26.5	29	34.5	32.7	26.4
28.1	38	37.7	36.1	25.9
29.6	34.5	35.6	30.5	20.6

n=8 mice/ group

Daily Intake (g)

	Com			Cel-0.5			Cel-1.0			Cel-2.0		
	Mean	Sem	N	Mean	Sem	N	Mean	Sem	N	Mean	Sem	N
day1	2.35	0.15	8	2.45	0.02	8	2.35	0.05	8	2.65	0.15	8
day2	2.75	0.15	8	2.65	0.04	8	1.85	0.25	8	1.5	0.4	8
day3	2.8	0.3	8	2.65	0.05	8	2.1	0.03	8	1.45	0.15	8
day4	2.7	0.2	8	2.55	0.03	8	2.1	0.08	8	1.1	0.2	8
day5	2.8	0.1	8	2.7	0.07	8	2	0.12	8	1.25	0.05	8
day6	2.9	0.2	8	2.6	0.08	8	2.1	0.1	8	1.01	0.2	8
day7	3.1	0.1	8	2.65	0.35	8	1.7	0.04	8	0.9	0.1	8
day8	2.9	0.2	8	2.75	0.25	8	1.7	0.04	8	0.8	0.1	8
day9	2.89	0.2	8	2.55	0.05	8	1.8	0.13	8	0.9	0	8
day10	2.95	0.05	8	2.4	0.3	8	1.6	0.04	8	1	0	8
day11	2.76	0	8	2.25	0.15	8	1.5	0.03	8	1	0.3	8
day12	2.69	0.04	8	2.1	0.1	8	1.55	0.05	8	0.95	0.25	8

White adipose (%)

Chow	Com	Cel-0.5	Cel-1.0	Cel-2.0
0.0135	0.0842	0.0829	0.0538	0.0542
0.0145	0.0499	0.0672	0.0201	0.0423
0.0128	0.0433	0.0541	0.0687	0.0244
0.0167	0.0743	0.0345	0.066	0.0497
0.0127	0.068	0.0986	0.0784	0.0643
0.0146	0.113	0.0453	0.0755	0.0353
0.0077	0.0459	0.0852	0.0514	0.0274
0.0112	0.0839	0.0891	0.0771	0.0543

Brown adipose (%)

Chow	Com	Cel-0.5	Cel-1.0	Cel-2.0
0.0062	0.0049	0.0041	0.0053	0.005611
0.0061	0.006	0.0045	0.0065	0.004291
0.0063	0.0035	0.0068	0.0055	0.005126
0.0057	0.0033	0.0052	0.0069	0.006888
0.0044	0.0035	0.0036	0.0038	0.005065
0.0048	0.0019	0.0044	0.0052	0.006856
0.0081	0.0041	0.0049	0.0065	0.006293
0.0049	0.0013	0.006	0.0043	0.007816

Blood Glucose (mmol/L)

min (min)	Chow Mean	Sem	N	Com Mean	Sem	N	Cel-0.5 Mean	Sem	N	Cel-1.0 Mean	Sem	N	Cel-2.0 Mean	Sem	N
0	7.867	0.2511	9	10.87	0.36	9	11.43	0.44	9	9.444	0.9541	9	6.078	0.3624	9
15	19.68	1.157	9	25.98	1.4	9	23.14	1.366	9	21.62	1.544	9	14.57	0.7106	9
30	16.94	1.487	9	30.13	0.99	9	26.83	1.328	9	22.77	1.931	9	15.92	1.008	9
60	11.84	1.007	9	27.12	1.33	9	24.41	1.934	9	18.23	1.773	9	14.83	1.932	9
120	9.167	0.5612	9	17.19	1.51	9	16.3	1.799	9	10.21	0.8669	9	11.6	2.029	9

TG (mmol/L) Chow	Com	Cel-2.0	TC (mmol/L) Chow	Com	Cel-2.0	LDL (mmol/L) Chow	Com	Cel-2.0	HDL (mmol/L) Chow	Com	Cel-2.0
0.8	1.02	1.05	2.99	7.82	4.39	0.24	0.56	0.38	2.01	2.38	2.14
0.68	1.11	1.12	2.44	6.19	2.91	0.2	0.62	0.39	1.71	2.39	1.47
0.77	1.86	0.64	2.71	5.95	3.66	0.23	0.62	0.31	1.84	2.65	1.96
0.68	1.38	1.03	2.31	5.36	3.96	0.19	0.48	0.31	1.54	2.44	2.1
0.69	1.71	0.89	3.01	5.28	3.4	0.25	0.47	0.26	1.84	2.49	1.85
0.47	1.13	0.94	2.56	4.9	4.5	0.19	0.56	0.37	1.67	2.21	2.18
0.71	1.59	1.08	2.43	4.88	3.57	0.22	0.48	0.46	1.6	2.31	2.02
0.58	1.19	0.78	2.09	5.59	3.91	0.26	0.55	0.28	1.25	2.28	1.72
0.75	1.14	1.07	2.65	5.08	2.62	0.18	0.36	0.45	1.76	2.41	1.1

Fig1. C

Tail suspension test-Immunobility time (S)

Chow	Com	Cel-0.5	Cel-1.0	Cel-2.0
104	168	118	149	108
81	172	117	103	105
103	136	162	130	97
80	176	135	114	125
90	179	75	92	68
76	107	82	135	51
102	162	80	134	80
77	162	129	113	92
81	106	132	107	101
93	127	130	121	87

n=10 mice/ group

Forces swimming test-Immunobility time (S)

Chow	Com	Cel-0.5	Cel-1.0	Cel-2.0
183	223	219	198	193
177	218	208	192	196
201	219	213	210	180
204	227	220	213	171
208	230	223	191	192
193	230	221	189	173
199	211	210	210	199
172	217	206	203	190
197	224	195	190	169
176	230	198	199	174

n=10 mice/ group

Fig1. D

TPH2-BDNF doube

Chow	Com	Cel-2.0
21	14	26
17	11	23
16	13	20
25	10	19
18	16	22

n=5 mice/ group

Fig1.E

	Chow					Obesity					Comorbidity				
GM-CSF	1.18	0.81	0.65	0.65	0.99	0.81	0.65	1.18	0.81	0.81	0.81	0.81	0.81	0.99	0.81
CCL3	0.97	0.89	0.8	0.72	0.64	0.89	0.97	1.41	1.23	1.11	1.05	1.14	1.23	1.23	1.05
IL-3	0.31	0.31	0.24	0.31	0.44	0.44	0.19	0.19	0.31	0.44	0.37	0.31	0.31	0.31	0.31
IFN-γ	3.83	3.83	4	2.66	5.19	4.51	3.67	3.5	2.99	3.67	3.5	3.83	3.67	4.51	3.16
IL-6	11.88	12.8	12.49	12.49	17.36	13.1	13.71	11.58	11.88	11.27	11.88	12.49	13.1	14.32	11.27
IL-10	33.26	25.51	8.07	8.55	22.42	13.49	24.22	12.24	25	22.42	16.27	16.78	6.89	13.24	10.01
IL-13	24.05	24.05	19.25	14.48	22.45	19.25	20.85	20.85	28.85	19.25	22.45	24.05	17.66	20.85	25.65
IL-16	38.57	41.52	35.88	32.83	32.74	33.67	36.61	32.92	33.11	41.44	35.7	38.39	37.14	39.79	34.78
IL-27	9.62	10.88	10.88	5.85	8.36	13.38	15.86	5.85	5.85	5.85	10.88	8.36	2.7	5.85	8.36
CCL5	16.84	16.84	12.51	16.84	16.84	12.51	30.18	21.26	25.71	25.71	16.84	21.26	16.84	16.84	21.26
CCL12	30.64	29.02	23.95	24.82	25.25	24.39	29.22	32.22	26.53	36.07	28.61	29.22	29.42	28.61	26.95
CCL22	10.89	13.41	11.74	12.58	8.31	13.41	9.17	8.31	14.24	10.04	10.04	11.74	10.89	9.17	12.58
VEGF	10.3	13.47	10.3	8.8	7.68	9.55	6.94	6.94	8.05	11.42	9.55	11.79	11.42	10.67	9.17
FGF-21	34.99	34.99	30.06	29.45	39.92	33.14	38.69	36.22	34.37	30.06	33.76	35.61	32.53	30.06	31.3
MMP12	22.99	17.98	21.26	21.83	27.22	16.44	21.54	20.68	18.69	26.78	17.84	17.84	19.26	19.26	14.23
IL-1α	121.29	152.36	110.04	103.53	113.28	162.9	130.77	83.55	150.84	103.53	129.2	129.2	90.28	93.62	126.05
IL-1β	131.14	165.6	142.73	131.14	136.95	142.73	131.14	107.63	131.14	119.43	142.73	142.73	142.73	119.43	142.73
IL-4	29.76	61.91	7.83	13.57	5.66	22.12	5.66	8.94	14.76	17.18	18.4	5.66	10.08	45.59	42.91
IL-17	47.07	34.75	30.26	30.66	30.66	22.98	32.7	37.21	36.39	46.25	43.78	36.39	32.3	41.32	36.39
IL-33	54.91	88.57	48.37	41.94	88.57	64.86	89.14	102.34	78.34	95.44	116.21	123.17	61.52	158.15	74.95
TNFR II	159.97	171.21	158.71	144.67	138.84	169.97	158.71	156.18	164.37	185.91	167.49	173.68	169.97	154.28	163.74
TNFR I	55.42	57.02	54.96	51.21	51.64	54.4	64.4	54.57	51.9	55.9	75.64	70.37	53.19	68.13	57.49

CXCL1	36.61	43.76	34.15	32.9	41.41	31.64	44.92	46.07	41.41	47.21	40.23	44.92	39.63	35.39	37.83
CCL4	42.32	32.81	32.81	29.1	69.88	51.66	32.81	32.81	32.81	51.66	51.66	32.81	32.81	51.66	32.81
TIMP-1	123.14	143.65	117.89	128.34	101.86	143.65	123.14	128.34	128.34	158.56	123.14	128.34	130.92	123.14	128.34
C1qR1	683.91	613.09	521.68	446.58	461.67	451.61	546.51	481.74	436.49	837.98	666.88	652.25	539.07	654.69	561.37
FGF-basic	2366	3217	3024	2322	1688	2536	2138	1819	2173	2572	2893	2314	2293	3084	2697
Angiopoietin-2	539.4	787.15	593.16	483	423.58	693.92	453.69	423.58	483	566.59	593.16	553.08	566.59	483	511.55

n=5 mice/ group

Fig.1F

No.TNFα-TMEM119			TNFα/GAPDH mRNA expression in BLA		
Chow	Com	Cel-2.0	Chow	Com	Cel-2.0
4.67	15.33	6.33	0.00001	0.00082	0.00012
6.33	20.00	4.33	0.00007	0.00132	0.00008
8.33	25.67	6.00	0.00002	0.00083	0.00004
7.67	25.00	10.00	0.00005	0.00095	0.00001
8.00	22.00	8.40			

n=5 mice/ group

n=4 mice/ group

Fig.1G

Tail suspension test-Immunobility				Forced swimming test-			
Chow-PBS	Chow-TNFa	DIO-PBS	DIO-TNFa	Chow-PBS	Chow-TNFa	DIO-PBS	DIO-TNFa
131	87	101	147	180	177	144	230
86	46	128	128	149	165	189	235
13	134	99	117	210	203	171	220
124	123	127	182	202	213	201	216
88	55	35	171	206	159	197	210
105	114	140	139	199	204	211	199
90	70	47	155	187	162	205	186
67	117	120	116	163	180	182	227

n=8 mice/ group

n=8 mice/ group

Fig2.C

Celastrol (μM)	HnRNPA1 (52°C pixels)	HnRNPA1 (37°C pixels)	HnRNPA1 (52°C/ 37°C)
0	8157.468	26100.589	0.3125396
5	12127.64	28028.104	0.4326957
10	12545.64	28098.225	0.4464923
50	17066.589	27451.811	0.6216926
100	20526.054	28977.518	0.7083441
200	24033.125	29343.711	0.8190213

Fig. 2E

	1.56 µM		3.125 µM		6.25 µM		12.5 µM		25 µM		data		data		data		data		data
-37.80000305	-1.127498092	-37.70000076	-1.1127	-37.6	-0.9713	-37.5	-0.9566	-37.5	-0.8837	-37.8	0	-37.7	0	-37.6	0	-37.5	0	-37.5	0
-37.70000076	-1.109100053	-37.59999847	-1.0463	-37.5	-0.9792	-37.4	-0.959999	-37.4	-0.775699	-37.7	0	-37.6	0	-37.5	0	-37.4	0	-37.4	0
-37.60000229	-1.066599979	-37.5	-0.9557	-37.4	-0.942	-37.3	-0.8697	-37.3	-0.6625	-37.6	0	-37.5	0	-37.4	0	-37.3	0	-37.3	0
-37.5	-1.009400042	-37.39999771	-0.929099	-37.3	-0.9044	-37.2	-0.663	-37.2	-0.5791	-37.5	0	-37.4	0	-37.3	0	-37.2	0	-37.2	0
-37.40000153	-0.894299956	-37.29999924	-0.7549	-37.2	-0.8521	-37.1	-0.5963	-37.1	-0.4678	-37.4	0	-37.3	0	-37.2	0	-37.1	0	-37.1	0
-37.30000305	-0.923200707	-37.20000076	-0.7776	-37.1	-0.8312	-37	-0.6385	-37	-0.5283	-37.3	0	-37.2	0	-37.1	0	-37	0	-37	0
-37.20000076	-0.921000017	-37.09999847	-0.8336	-37	-0.8146	-36.9	-0.7352	-36.9	-0.6295	-37.2	0	-37.1	0	-37	0	-36.9	0	-36.9	0
-37.10000229	-0.988000043	-37	-0.9166	-36.9	-0.913	-36.8	-0.8393	-36.8	-0.7096	-37.1	0	-37	0	-36.9	0	-36.8	0	-36.8	0
-37	-1.112100006	-36.89999771	-0.9198	-36.8	-1.027	-36.7	-0.9313	-36.7	-0.778	-37	0	-36.9	0	-36.8	0	-36.7	0	-36.7	0
-36.90000153	-1.162500054	-36.79999924	-1.0125	-36.7	-1.0869	-36.6	-1.0163	-36.6	-0.889	-36.9	0	-36.8	0	-36.7	0	-36.6	0	-36.6	0
-36.80000305	-1.246600332	-36.70000076	-1.0961	-36.6	-1.1279	-36.5	-1.0934	-36.5	-0.9321	-36.8	0	-36.7	0	-36.6	0	-36.5	0	-36.5	0
-36.70000076	-1.288799944	-36.59999847	-1.1196	-36.5	-1.1524	-36.4	-1.0878	-36.4	-0.9943	-36.7	0	-36.6	0	-36.5	0	-36.4	0	-36.4	0
-36.60000229	-1.33059997	-36.5	-1.1786	-36.4	-1.1873	-36.3	-1.1127	-36.3	-0.9919	-36.6	0	-36.5	0	-36.4	0	-36.3	0	-36.3	0
-36.5	-1.343200044	-36.39999771	-1.2137	-36.3	-1.2043	-36.2	-1.1153	-36.2	-1.0118	-36.5	0	-36.4	0	-36.3	0	-36.2	0	-36.2	0
-36.40000153	-1.383800058	-36.29999924	-1.2368	-36.2	-1.2153	-36.1	-1.1718	-36.1	-1.0396	-36.4	0	-36.3	0	-36.2	0	-36.1	0	-36.1	0
-36.30000305	-1.357000346	-36.20000076	-1.2551	-36.1	-1.2621	-36	-1.197	-36	-1.0443	-36.3	0	-36.2	0	-36.1	0	-36	0	-36	0
-36.20000076	-1.360299997	-36.09999847	-1.2607	-36	-1.2608	-35.9	-1.2036	-35.9	-1.0548	-36.2	0	-36.1	0	-36	0	-35.9	0	-35.9	0
-36.10000229	-1.390399991	-36	-1.2742	-35.9	-1.2762	-35.8	-1.2219	-35.8	-1.0953	-36.1	0	-36	0	-35.9	0	-35.8	0	-35.8	0
-36	-1.406199975	-35.89999771	-1.3153	-35.8	-1.3099	-35.7	-1.2105	-35.7	-1.0873	-36	0	-35.9	0	-35.8	0	-35.7	0	-35.7	0
-35.90000153	-1.382899981	-35.79999924	-1.339	-35.7	-1.3075	-35.6	-1.1949	-35.6	-1.0953	-35.9	0	-35.8	0	-35.7	0	-35.6	0	-35.6	0
-35.80000305	-1.421899647	-35.70000076	-1.3196	-35.6	-1.2746	-35.5	-1.1805	-35.5	-1.0887	-35.8	0	-35.7	0	-35.6	0	-35.5	0	-35.5	0
-35.70000076	-1.419700003	-35.59999847	-1.3488	-35.5	-1.2595	-35.4	-1.1628	-35.4	-1.0692	-35.7	0	-35.6	0	-35.5	0	-35.4	0	-35.4	0
-35.60000229	-1.411699994	-35.5	-1.3414	-35.4	-1.2601	-35.3	-1.1566	-35.3	-1.0646	-35.6	0	-35.5	0	-35.4	0	-35.3	0	-35.3	0
-35.5	-1.423099962	-35.39999771	-1.3267	-35.3	-1.2614	-35.2	-1.1118	-35.2	-1.0666	-35.5	0	-35.4	0	-35.3	0	-35.2	0	-35.2	0
-35.40000153	-1.383600002	-35.29999924	-1.3069	-35.2	-1.2225	-35.1	-1.121599	-35.1	-1.036599	-35.4	0	-35.3	0	-35.2	0	-35.1	0	-35.1	0
-35.30000305	-1.369700159	-35.20000076	-1.3114	-35.1	-1.2344	-35	-1.1045	-35	-1.0297	-35.3	0	-35.2	0	-35.1	0	-35	0	-35	0
-35.20000076	-1.3509	-35.09999847	-1.308399	-35	-1.2259	-34.9	-1.0633	-34.9	-1.0007	-35.2	0	-35.1	0	-35	0	-34.9	0	-34.9	0
-35.10000229	-1.365499994	-35	-1.2889	-34.9	-1.1964	-34.8	-1.0553	-34.8	-0.9926	-35.1	0	-35	0	-34.9	0	-34.8	0	-34.8	0
-35	-1.324300002	-34.89999771	-1.2574	-34.8	-1.1676	-34.7	-1.0647	-34.7	-0.9865	-35	0	-34.9	0	-34.8	0	-34.7	0	-34.7	0
-34.90000153	-1.300599998	-34.79999924	-1.2511	-34.7	-1.1762	-34.6	-1.0379	-34.6	-0.9776	-34.9	0	-34.8	0	-34.7	0	-34.6	0	-34.6	0
-34.80000305	-1.292000116	-34.70000076	-1.2461	-34.6	-1.1628	-34.5	-1.0371	-34.5	-0.9735	-34.8	0	-34.7	0	-34.6	0	-34.5	0	-34.5	0
-34.70000076	-1.268500013	-34.59999847	-1.2323	-34.5	-1.1429	-34.4	-1.0289	-34.4	-0.945	-34.7	0	-34.6	0	-34.5	0	-34.4	0	-34.4	0
-34.60000229	-1.255699993	-34.5	-1.2495	-34.4	-1.1308	-34.3	-1.011	-34.3	-0.9531	-34.6	0	-34.5	0	-34.4	0	-34.3	0	-34.3	0
-34.5	-1.255100019	-34.39999771	-1.2191	-34.3	-1.119	-34.2	-1.0093	-34.2	-0.9207	-34.5	0	-34.4	0	-34.3	0	-34.2	0	-34.2	0
-34.40000153	-1.239699973	-34.29999924	-1.2029	-34.2	-1.1004	-34.1	-0.9992	-34.1	-0.9159	-34.4	0	-34.3	0	-34.2	0	-34.1	0	-34.1	0
-34.30000305	-1.218100105	-34.20000076	-1.167	-34.1	-1.0878	-34	-0.9991	-34	-0.9208	-34.3	0	-34.2	0	-34.1	0	-34	0	-34	0
-34.20000076	-1.199999984	-34.09999847	-1.1683	-34	-1.0819	-33.9	-0.9854	-33.9	-0.9151	-34.2	0	-34.1	0	-34	0	-33.9	0	-33.9	0
-34.10000229	-1.189000003	-34	-1.1681	-33.9	-1.0779	-33.8	-0.9736	-33.8	-0.9085	-34.1	0	-34	0	-33.9	0	-33.8	0	-33.8	0
-34	-1.183599971	-33.89999771	-1.158	-33.8	-1.0674	-33.7	-0.9634	-33.7	-0.8974	-34	0	-33.9	0	-33.8	0	-33.7	0	-33.7	0
-33.90000153	-1.17449999	-33.79999924	-1.1468	-33.7	-1.0574	-33.6	-0.9552	-33.6	-0.8941	-33.9	0	-33.8	0	-33.7	0	-33.6	0	-33.6	0
-33.80000305	-1.163600193	-33.70000076	-1.1364	-33.6	-1.043	-33.5	-0.9542	-33.5	-0.889	-33.8	0	-33.7	0	-33.6	0	-33.5	0	-33.5	0
-33.70000076	-1.151999996	-33.59999847	-1.1248	-33.5	-1.0355	-33.4	-0.9488	-33.4	-0.8799	-33.7	0	-33.6	0	-33.5	0	-33.4	0	-33.4	0
-33.60000229	-1.138099971	-33.5	-1.116	-33.4	-1.0284	-33.3	-0.9419	-33.3	-0.8708	-33.6	0	-33.5	0	-33.4	0	-33.3	0	-33.3	0
-33.5	-1.130699996	-33.39999771	-1.1045	-33.3	-1.0232	-33.2	-0.9337	-33.2	-0.8644	-33.5	0	-33.4	0	-33.3	0	-33.2	0	-33.2	0
-33.40000153	-1.120599972	-33.29999924	-1.0904	-33.2	-1.0119	-33.1	-0.9258	-33.1	-0.8566	-33.4	0	-33.3	0	-33.2	0	-33.1	0	-33.1	0
-33.30000305	-1.108000093	-33.20000076	-1.0779	-33.1	-1.0027	-33	-0.9187	-33	-0.8478	-33.3	0	-33.2	0	-33.1	0	-33	0	-33	0
-33.20000076	-1.097500025	-33.09999847	-1.0659	-33	-0.996	-32.9	-0.91	-32.9	-0.8372	-33.2	0	-33.1	0	-33	0	-32.9	0	-32.9	0
-33.10000229	-1.087500027	-33	-1.0574	-32.9	-0.9877	-32.8	-0.9023	-32.8	-0.829	-33.1	0	-33	0	-32.9	0	-32.8	0	-32.8	0
-33	-1.078500006	-32.89999771	-1.0446	-32.8	-0.9758	-32.7	-0.8924	-32.7	-0.8208	-33	0	-32.9	0	-32.8	0	-32.7	0	-32.7	0
-32.90000153	-1.067899994	-32.79999924	-1.0338	-32.7	-0.964	-32.6	-0.8773	-32.6	-0.8075	-32.9	0	-32.8	0	-32.7	0	-32.6	0	-32.6	0
-32.80000305	-1.057000204	-32.70000076	-1.0201	-32.6	-0.9524	-32.5	-0.8705	-32.5	-0.7984	-32.8	0	-32.7	0	-32.6	0	-32.5	0	-32.5	0
-32.70000076	-1.0493	-32.59999847	-1.0066	-32.5	-0.9415	-32.4	-0.8646	-32.4	-0.7896	-32.7	0	-32.6	0	-32.5	0	-32.4	0	-32.4	0
-32.60000229	-1.03679998	-32.5	-0.998	-32.4	-0.9307	-32.3	-0.8539	-32.3	-0.7785	-32.6	0	-32.5	0	-32.4	0	-32.3	0	-32.3	0
-32.5	-1.029499971	-32.39999771	-0.9912	-32.3	-0.9222	-32.2	-0.8482	-32.2	-0.7727	-32.5	0	-32.4	0	-32.3	0	-32.2	0	-32.2	0
-32.40000153	-1.021100006	-32.29999924	-0.9747	-32.2	-0.91	-32.1	-0.8399	-32.1	-0.7617	-32.4	0	-32.3	0	-32.2	0	-32.1	0	-32.1	0
-32.30000305	-1.008500305	-32.20000076	-0.9594	-32.1	-0.8992	-32	-0.8352	-32	-0.752	-32.3	0	-32.2	0	-32.1	0	-32	0	-32	0
-32.20000076	-1.002999975	-32.09999847	-0.9495	-32	-0.8906	-31.9	-0.8319	-31.9	-0.7426	-32.2	0	-32.1	0	-32	0	-31.9	0	-31.9	0
-32.10000229	-0.993099989	-32	-0.9438	-31.9	-0.8817	-31.8	-0.8203	-31.8	-0.7339	-32.1	0	-32	0	-31.9	0	-31.8	0	-31.8	0
-32	-0.981799977	-31.89999962	-0.9344	-31.8	-0.8726	-31.7	-0.8136	-31.7	-0.7272	-32	0	-31.9	0	-31.8	0	-31.7	0	-31.7	0
-31.90000153	-0.966699992	-31.79999924	-0.9223	-31.7	-0.8639	-31.6	-0.8064	-31.6	-0.7181	-31.9	0	-31.8	0	-31.7	0	-31.6	0	-31.6	0
-31.80000114	-0.95850002	-31.69999886	-0.9068	-31.6	-0.8588	-31.5	-0.7969	-31.5	-0.7101	-31.8	0	-31.7	0	-31.6	0	-31.5	0	-31.5	0
-31.70000076	-0.951700009	-31.59999847	-0.9008	-31.5	-0.8558	-31.4	-0.7996	-31.4	-0.7053	-31.7	0	-31.6	0	-31.5	0	-31.4	0	-31.4	0
-31.60000229	-0.942700031	-31.5	-0.8916	-31.4	-0.8446	-31.3	-0.7914	-31.3	-0.6974	-31.6	0	-31.5	0	-31.4	0	-31.3	0	-31.3	0
-31.50000191	-0.93820006	-31.39999962	-0.8855	-31.3	-0.8326	-31.2	-0.7919	-31.2	-0.7052	-31.5	0	-31.4	0	-31.3	0	-31.2	0	-31.2	0
-31.40000153	-0.93169998	-31.29999924	-0.8819	-31.2	-0.8285	-31.1	-0.7869	-31.1	-0.6987	-31.4	0	-31.3	0	-31.2	0	-31.1	0	-31.1	0
-31.30000114	-0.919399982	-31.19999886	-0.8783	-31.1	-0.8186	-31	-0.7794	-31	-0.6921	-31.3	0	-31.2	0	-31.1	0	-31	0	-31	0
-31.20000076	-0.912700001	-31.09999847	-0.8691	-31	-0.81	-30.9	-0.7694	-30.9	-0.6844	-31.2	0	-31.1	0	-31	0	-30.9	0	-30.9	0
-31.10000229	-0.908300082	-31	-0.8609	-30.9	-0.7985	-30.8	-0.7665	-30.8	-0.6826	-31.1	0	-31	0	-30.9	0	-30.8	0	-30.8	0
-31.00000191	-0.896300067	-30.89999962	-0.8535	-30.8	-0.7927	-30.7	-0.7646	-30.7	-0.677	-31	0	-30.9	0	-30.8	0	-30.7	0	-30.7	0
-30.90000153	-0.885499976	-30.79999924	-0.8514	-30.7	-0.7878	-30.6	-0.7574	-30.6	-0.6717	-30.9	0	-30.8	0	-30.7	0	-30.6	0	-30.6	0
-30.80000114	-0.8763	-30.69999886	-0.8467	-30.6	-0.7757	-30.5	-0.7504	-30.5	-0.6653	-30.8	0	-30.7	0	-30.6	0	-30.5	0	-30.5	0
-30.70000076	-0.865899976	-30.59999847	-0.8376	-30.5	-0.7645	-30.4	-0.7464	-30.4	-0.6659	-30.7	0	-30.6	0	-30.5	0	-30.4	0	-30.4	0
-30.60000229	-0.847600031	-30.5	-0.8278	-30.4	-0.759	-30.3	-0.7405	-30.3	-0.6643	-30.6	0	-30.5	0	-30.4	0	-30.3	0	-30.3	0
-30.50000191	-0.832500109	-30.39999962	-0.8226	-30.3	-0.7529	-30.2	-0.731	-30.2	-0.6555	-30.5	0	-30.4	0	-30.3	0	-30.2	0	-30.2	0
-30.40000153	-0.83159998	-30.29999924	-0.8171	-30.2	-0.7443	-30.1	-0.7222	-30.1	-0.6458	-30.4	0	-30.3	0	-30.2	0	-30.1	0	-30.1	0
-30.30000114	-0.829000029	-30.19999886	-0.8102	-30.1	-0.7347	-30	-0.7154	-30	-0.6391	-30.3	0	-30.2	0	-30.1	0	-30	0	-30	0
-30.20000076	-0.827199991	-30.09999847	-0.806	-30	-0.7339	-29.9	-0.7134	-29.9	-0.638	-30.2	0	-30.1	0	-30	0	-29.9	0	-29.9	0
-30.10000229	-0.816000127	-30	-0.7978	-29.9	-0.7347	-29.8	-0.7139	-29.8	-0.6241	-30.1	0	-30	0	-29.9	0	-29.8	0	-29.8	0
-30.00000191	-0.817500034	-29.89999962	-0.7916	-29.8	-0.7237	-29.7	-0.709	-29.7	-0.6173	-30	0	-29.9	0	-29.8	0	-29.7	0	-29.7	0
-29.90000153	-0.813000011	-29.79999924	-0.7844	-29.7	-0.72	-29.6	-0.7096	-29.6	-0.6115	-29.9	0	-29.8	0	-29.7	0	-29.6	0	-29.6	0
-29.80000114	-0.805600018	-29.69999886	-0.7795	-29.6	-0.7156	-29.5	-0.6975	-29.5	-0.6067	-29.8	0	-29.7	0	-29.6	0	-29.5	0	-29.5	0
-29.70000076	-0.799900023	-29.59999847	-0.7755	-29.5	-0.7085	-29.4	-0.6923	-29.4	-0.6038	-29.7	0	-29.6	0	-29.5	0	-29.4	0	-29.4	0
-29.60000229	-0.796200052	-29.5	-0.7718	-29.4	-0.7084	-29.3	-0.6927	-29.3	-0.5999	-29.6	0	-29.5	0	-29.4	0	-29.3	0	-29.3	0
-29.50000191	-0.791900081	-29.39999962	-0.7695	-29.3	-0.7069	-29.2	-0.685	-29.2	-0.5916	-29.5	0	-29.4	0	-29.3	0	-29.2	0	-29.2	0
-29.40000153	-0.788899971	-29.29999924	-0.7673	-29.2	-0.697	-29.1	-0.6776	-29.1	-0.5859	-29.4	0	-29.3	0	-29.2	0	-29.1	0	-29.1	0
-29.30000114	-0.784900024	-29.19999886	-0.7605	-29.1	-0.6881	-29	-0.6799	-29	-0.5841	-29.3	0	-29.2	0	-29.1	0	-29	0	-29	0
-29.20000076	-0.780499976	-29.09999847	-0.7546	-29	-0.6888	-28.9	-0.6773	-28.9	-0.5786	-29.2	0	-29.1	0	-29	0	-28.9	0	-28.9	0
-29.10000229	-0.774800065	-29	-0.7498	-28.9	-0.6876	-28.8	-0.6683	-28.8	-0.5729	-29.1	0	-29	0	-28.9	0	-28.8	0	-28.8	0
-29.00000191	-0.776500005	-28.89999962	-0.7409	-28.8	-0.6854	-28.7	-0.6628	-28.7	-0.5727	-29	0	-28.9	0	-28.8	0	-28.7	0	-28.7	0
-28.90000153	-0.773700027	-28.79999924	-0.735	-28.7	-0.6829	-28.6	-0.6572	-28.6	-0.5702	-28.9	0	-28.8	0	-28.7	0	-28.6	0	-28.6	0
-28.80000114	-0.766199986	-28.69999886	-0.7348	-28.6	-0.6764	-28.5	-0.6516	-28.5	-0.5614	-28.8	0	-28.7	0	-28.6	0	-28.5	0	-28.5	0
-28.70000076	-0.761000008	-28.59999847	-0.7346	-28.5	-0.6657	-28.4	-0.6483	-28.4	-0.5585	-28.7	0	-28.6	0	-28.5	0	-28.4	0	-28.4	0
-28.60000229	-0.754000024	-28.5	-0.7272	-28.4	-0.6593	-28.3	-0.6396	-28.3	-0.5501	-28.6	0	-28.5	0	-28.4	0	-28.3	0	-28.3	0
-28.50000191	-0.751000079	-28.39999962	-0.7181	-28.3	-0.6494	-28.2	-0.6359	-28.2	-0.543	-28.5	0	-28.4	0	-28.3	0	-28.2	0	-28.2	0
-28.40000153	-0.746800015	-28.29999924	-0.7094	-28.2	-0.6435	-28.1	-0.6314	-28.1	-0.5433	-28.4	0	-28.3	0	-28.2	0	-28.1	0	-28.1	0
-28.30000114	-0.738899986	-28.19999886	-0.7038	-28.1	-0.641	-28	-0.6244	-28	-0.5383	-28.3	0	-28.2	0	-28.1	0	-28	0	-28	0
-28.20000076	-0.738599982	-28.09999847	-0.701	-28	-0.6339	-27.9	-0.6235	-27.9	-0.5382	-28.2	0	-28.1	0	-28	0	-27.9	0	-27.9	0
-28.10000229	-0.736799995	-28	-0.6962	-27.9	-0.6285	-27.8	-0.624	-27.8	-0.5386	-28.1	0	-28	0	-27.9	0	-27.8	0	-27.8	0
-28.00000191	-0.732500064	-27.89999962	-0.6941	-27.8	-0.6279	-27.7	-0.6231	-27.7	-0.5381	-28	0	-27.9	0	-27.8	0	-27.7	0	-27.7	0
-27.90000153	-0.732900011	-27.79999924	-0.6944	-27.7	-0.6266	-27.6	-0.6158	-27.6	-0.5321	-27.9	0	-27.8	0	-27.7	0	-27.6	0	-27.6	0
-27.80000114	-0.731800021	-27.69999886	-0.6926	-27.6	-0.6199	-27.5	-0.6093	-27.5	-0.5277	-27.8	0	-27.7	0	-27.6	0	-27.5	0	-27.5	0
-27.70000076	-0.725799988	-27.59999847	-0.687	-27.5	-0.6169	-27.4	-0.6054	-27.4	-0.5245	-27.7	0	-27.6	0	-27.5	0	-27.4	0	-27.4	0

-27.60000229	-0.716100079	-27.5	-0.6782	-27.4	-0.6113	-27.3	-0.603	-27.3	-0.5208	-27.6	0	-27.5	0	-27.4	0	-27.3	0	-27.3	0
-27.50000191	-0.707400106	-27.39999962	-0.6708	-27.3	-0.6073	-27.2	-0.5972	-27.2	-0.5174	-27.5	0	-27.4	0	-27.3	0	-27.2	0	-27.2	0
-27.40000153	-0.702999982	-27.29999924	-0.6664	-27.2	-0.6004	-27.1	-0.5899	-27.1	-0.5123	-27.4	0	-27.3	0	-27.2	0	-27.1	0	-27.1	0
-27.30000114	-0.700099978	-27.19999886	-0.6579	-27.1	-0.5935	-27	-0.5823	-27	-0.5088	-27.3	0	-27.2	0	-27.1	0	-27	0	-27	0
-27.20000076	-0.701799993	-27.09999847	-0.6559	-27	-0.5892	-26.9	-0.578	-26.9	-0.5078	-27.2	0	-27.1	0	-27	0	-26.9	0	-26.9	0
-27.10000229	-0.701200048	-27	-0.6518	-26.9	-0.5884	-26.8	-0.5755	-26.8	-0.5027	-27.1	0	-27	0	-26.9	0	-26.8	0	-26.8	0
-27.00000191	-0.702200057	-26.89999962	-0.6451	-26.8	-0.5842	-26.7	-0.5744	-26.7	-0.5007	-27	0	-26.9	0	-26.8	0	-26.7	0	-26.7	0
-26.90000153	-0.699800013	-26.79999924	-0.6342	-26.7	-0.5835	-26.6	-0.5723	-26.6	-0.4989	-26.9	0	-26.8	0	-26.7	0	-26.6	0	-26.6	0
-26.80000114	-0.693700002	-26.69999886	-0.6298	-26.6	-0.5847	-26.5	-0.564	-26.5	-0.4939	-26.8	0	-26.7	0	-26.6	0	-26.5	0	-26.5	0
-26.70000076	-0.689099998	-26.59999847	-0.6248	-26.5	-0.5796	-26.4	-0.5579	-26.4	-0.4927	-26.7	0	-26.6	0	-26.5	0	-26.4	0	-26.4	0
-26.60000229	-0.687000037	-26.5	-0.6232	-26.4	-0.5806	-26.3	-0.5499	-26.3	-0.489	-26.6	0	-26.5	0	-26.4	0	-26.3	0	-26.3	0
-26.50000191	-0.684500086	-26.39999962	-0.6207	-26.3	-0.5733	-26.2	-0.5471	-26.2	-0.4906	-26.5	0	-26.4	0	-26.3	0	-26.2	0	-26.2	0
-26.40000153	-0.681199993	-26.29999924	-0.6162	-26.2	-0.5689	-26.1	-0.5406	-26.1	-0.4886	-26.4	0	-26.3	0	-26.2	0	-26.1	0	-26.1	0
-26.30000114	-0.678900025	-26.19999886	-0.6187	-26.1	-0.5608	-26	-0.5335	-26	-0.4837	-26.3	0	-26.2	0	-26.1	0	-26	0	-26	0
-26.20000076	-0.676100005	-26.09999847	-0.6112	-26	-0.5545	-25.9	-0.527	-25.9	-0.4813	-26.2	0	-26.1	0	-26	0	-25.9	0	-25.9	0
-26.10000229	-0.672500099	-26	-0.6044	-25.9	-0.5482	-25.8	-0.5195	-25.8	-0.4777	-26.1	0	-26	0	-25.9	0	-25.8	0	-25.8	0
-26.00000191	-0.665600107	-25.89999962	-0.5997	-25.8	-0.5454	-25.7	-0.5164	-25.7	-0.4731	-26	0	-25.9	0	-25.8	0	-25.7	0	-25.7	0
-25.90000153	-0.662200029	-25.79999924	-0.594	-25.7	-0.5395	-25.6	-0.5091	-25.6	-0.4663	-25.9	0	-25.8	0	-25.7	0	-25.6	0	-25.6	0
-25.80000114	-0.658300024	-25.69999886	-0.5903	-25.6	-0.5312	-25.5	-0.5007	-25.5	-0.459	-25.8	0	-25.7	0	-25.6	0	-25.5	0	-25.5	0
-25.70000076	-0.654800016	-25.59999847	-0.5846	-25.5	-0.5239	-25.4	-0.4951	-25.4	-0.4535	-25.7	0	-25.6	0	-25.5	0	-25.4	0	-25.4	0
-25.60000229	-0.64770012	-25.5	-0.578	-25.4	-0.517	-25.3	-0.4857	-25.3	-0.4465	-25.6	0	-25.5	0	-25.4	0	-25.3	0	-25.3	0
-25.50000191	-0.640500101	-25.39999962	-0.5727	-25.3	-0.511	-25.2	-0.483	-25.2	-0.4451	-25.5	0	-25.4	0	-25.3	0	-25.2	0	-25.2	0
-25.40000153	-0.631600003	-25.29999924	-0.5654	-25.2	-0.5095	-25.1	-0.4802	-25.1	-0.4445	-25.4	0	-25.3	0	-25.2	0	-25.1	0	-25.1	0
-25.30000114	-0.625199974	-25.19999886	-0.5655	-25.1	-0.5087	-25	-0.4756	-25	-0.4422	-25.3	0	-25.2	0	-25.1	0	-25	0	-25	0
-25.20000076	-0.622399973	-25.09999847	-0.5623	-25	-0.507	-24.9	-0.4688	-24.9	-0.4308	-25.2	0	-25.1	0	-25	0	-24.9	0	-24.9	0
-25.10000229	-0.621300068	-25	-0.5606	-24.9	-0.4971	-24.8	-0.4627	-24.8	-0.4253	-25.1	0	-25	0	-24.9	0	-24.8	0	-24.8	0
-25.00000191	-0.616400106	-24.89999962	-0.555	-24.8	-0.4885	-24.7	-0.4582	-24.7	-0.425	-25	0	-24.9	0	-24.8	0	-24.7	0	-24.7	0
-24.90000153	-0.612899978	-24.79999924	-0.5481	-24.7	-0.483	-24.6	-0.4558	-24.6	-0.4193	-24.9	0	-24.8	0	-24.7	0	-24.6	0	-24.6	0
-24.80000114	-0.606600019	-24.69999886	-0.5449	-24.6	-0.4751	-24.5	-0.4466	-24.5	-0.4107	-24.8	0	-24.7	0	-24.6	0	-24.5	0	-24.5	0
-24.70000076	-0.604199972	-24.59999847	-0.541	-24.5	-0.4685	-24.4	-0.44	-24.4	-0.4083	-24.7	0	-24.6	0	-24.5	0	-24.4	0	-24.4	0
-24.60000229	-0.599300132	-24.5	-0.5298	-24.4	-0.4644	-24.3	-0.4352	-24.3	-0.4048	-24.6	0	-24.5	0	-24.4	0	-24.3	0	-24.3	0
-24.50000191	-0.589200142	-24.39999962	-0.5274	-24.3	-0.4599	-24.2	-0.429	-24.2	-0.4005	-24.5	0	-24.4	0	-24.3	0	-24.2	0	-24.2	0
-24.40000153	-0.579199975	-24.29999924	-0.5241	-24.2	-0.4611	-24.1	-0.4239	-24.1	-0.3994	-24.4	0	-24.3	0	-24.2	0	-24.1	0	-24.1	0
-24.30000114	-0.57320002	-24.19999886	-0.521	-24.1	-0.4622	-24	-0.4242	-24	-0.3932	-24.3	0	-24.2	0	-24.1	0	-24	0	-24	0
-24.20000076	-0.569600002	-24.09999847	-0.5139	-24	-0.4609	-23.9	-0.4207	-23.9	-0.388	-24.2	0	-24.1	0	-24	0	-23.9	0	-23.9	0
-24.10000229	-0.56740008	-24	-0.5113	-23.9	-0.4559	-23.8	-0.416	-23.8	-0.3883	-24.1	0	-24	0	-23.9	0	-23.8	0	-23.8	0
-24.00000191	-0.565700042	-23.89999962	-0.504	-23.8	-0.4512	-23.7	-0.4091	-23.7	-0.3862	-24	0	-23.9	0	-23.8	0	-23.7	0	-23.7	0
-23.90000153	-0.558200013	-23.79999924	-0.4996	-23.7	-0.4486	-23.6	-0.4116	-23.6	-0.3836	-23.9	0	-23.8	0	-23.7	0	-23.6	0	-23.6	0
-23.80000114	-0.554500019	-23.69999886	-0.4944	-23.6	-0.4482	-23.5	-0.4067	-23.5	-0.3788	-23.8	0	-23.7	0	-23.6	0	-23.5	0	-23.5	0
-23.70000076	-0.550600001	-23.59999847	-0.4938	-23.5	-0.4411	-23.4	-0.4001	-23.4	-0.3725	-23.7	0	-23.6	0	-23.5	0	-23.4	0	-23.4	0
-23.60000229	-0.549000046	-23.5	-0.486	-23.4	-0.4336	-23.3	-0.3927	-23.3	-0.3644	-23.6	0	-23.5	0	-23.4	0	-23.3	0	-23.3	0
-23.50000191	-0.5395001	-23.39999962	-0.4787	-23.3	-0.4281	-23.2	-0.3892	-23.2	-0.3643	-23.5	0	-23.4	0	-23.3	0	-23.2	0	-23.2	0
-23.40000153	-0.532499975	-23.29999924	-0.4711	-23.2	-0.4256	-23.1	-0.3854	-23.1	-0.3607	-23.4	0	-23.3	0	-23.2	0	-23.1	0	-23.1	0
-23.30000114	-0.521099999	-23.19999886	-0.4698	-23.1	-0.4171	-23	-0.3776	-23	-0.3569	-23.3	0	-23.2	0	-23.1	0	-23	0	-23	0
-23.20000076	-0.517300001	-23.09999847	-0.4661	-23	-0.4095	-22.9	-0.3711	-22.9	-0.3525	-23.2	0	-23.1	0	-23	0	-22.9	0	-22.9	0
-23.10000229	-0.514900085	-23	-0.4601	-22.9	-0.4037	-22.8	-0.366	-22.8	-0.348	-23.1	0	-23	0	-22.9	0	-22.8	0	-22.8	0
-23.00000191	-0.512900067	-22.89999962	-0.4527	-22.8	-0.4016	-22.7	-0.3608	-22.7	-0.3437	-23	0	-22.9	0	-22.8	0	-22.7	0	-22.7	0
-22.90000153	-0.511899994	-22.79999924	-0.4494	-22.7	-0.398	-22.6	-0.3521	-22.6	-0.3361	-22.9	0	-22.8	0	-22.7	0	-22.6	0	-22.6	0
-22.80000114	-0.509399993	-22.69999886	-0.443	-22.6	-0.3859	-22.5	-0.3466	-22.5	-0.3345	-22.8	0	-22.7	0	-22.6	0	-22.5	0	-22.5	0
-22.70000076	-0.503999986	-22.59999847	-0.4371	-22.5	-0.384	-22.4	-0.3425	-22.4	-0.3335	-22.7	0	-22.6	0	-22.5	0	-22.4	0	-22.4	0
-22.60000229	-0.491500161	-22.5	-0.4351	-22.4	-0.3818	-22.3	-0.3283	-22.3	-0.3297	-22.6	0	-22.5	0	-22.4	0	-22.3	0	-22.3	0
-22.50000191	-0.486100119	-22.39999962	-0.4336	-22.3	-0.3769	-22.2	-0.3195	-22.2	-0.3249	-22.5	0	-22.4	0	-22.3	0	-22.2	0	-22.2	0
-22.40000153	-0.48280001	-22.29999924	-0.4289	-22.2	-0.3759	-22.1	-0.3088	-22.1	-0.3194	-22.4	0	-22.3	0	-22.2	0	-22.1	0	-22.1	0
-22.30000114	-0.466499989	-22.19999886	-0.4235	-22.1	-0.373	-22	-0.3038	-22	-0.3162	-22.3	0	-22.2	0	-22.1	0	-22	0	-22	0
-22.20000076	-0.461099999	-22.09999847	-0.4086	-22	-0.3631	-21.9	-0.3015	-21.9	-0.3122	-22.2	0	-22.1	0	-22	0	-21.9	0	-21.9	0
-22.10000229	-0.453900223	-22	-0.4008	-21.9	-0.3588	-21.8	-0.2934	-21.8	-0.3044	-22.1	0	-22	0	-21.9	0	-21.8	0	-21.8	0
-22.00000191	-0.45000007	-21.89999962	-0.3901	-21.8	-0.3441	-21.7	-0.2833	-21.7	-0.2942	-22	0	-21.9	0	-21.8	0	-21.7	0	-21.7	0
-21.90000153	-0.444899999	-21.79999924	-0.3715	-21.7	-0.3302	-21.6	-0.2769	-21.6	-0.2817	-21.9	0	-21.8	0	-21.7	0	-21.6	0	-21.6	0
-21.80000114	-0.437099997	-21.69999886	-0.3523	-21.6	-0.3281	-21.5	-0.2754	-21.5	-0.284	-21.8	0	-21.7	0	-21.6	0	-21.5	0	-21.5	0
-21.70000076	-0.438700009	-21.59999847	-0.3467	-21.5	-0.3379	-21.4	-0.2857	-21.4	-0.2944	-21.7	0	-21.6	0	-21.5	0	-21.4	0	-21.4	0
-21.60000229	-0.443200042	-21.5	-0.3559	-21.4	-0.3493	-21.3	-0.2866	-21.3	-0.3044	-21.6	0	-21.5	0	-21.4	0	-21.3	0	-21.3	0
-21.50000191	-0.45029982	-21.39999962	-0.3665	-21.3	-0.3567	-21.2	-0.3015	-21.2	-0.3145	-21.5	0	-21.4	0	-21.3	0	-21.2	0	-21.2	0
-21.40000153	-0.456999996	-21.29999924	-0.3786	-21.2	-0.3625	-21.1	-0.3176	-21.1	-0.3195	-21.4	0	-21.3	0	-21.2	0	-21.1	0	-21.1	0
-21.30000114	-0.464499987	-21.19999886	-0.3853	-21.1	-0.3652	-21	-0.3106	-21	-0.3144	-21.3	0	-21.2	0	-21.1	0	-21	0	-21	0
-21.20000076	-0.474499993	-21.09999847	-0.39	-21	-0.3614	-20.9	-0.3106	-20.9	-0.3066	-21.2	0	-21.1	0	-21	0	-20.9	0	-20.9	0
-21.10000229	-0.479499866	-21	-0.3788	-20.9	-0.3546	-20.8	-0.3114	-20.8	-0.2995	-21.1	0	-21	0	-20.9	0	-20.8	0	-20.8	0
-21.00000191	-0.475700067	-20.89999962	-0.3749	-20.8	-0.3518	-20.7	-0.3093	-20.7	-0.2949	-21	0	-20.9	0	-20.8	0	-20.7	0	-20.7	0
-20.90000153	-0.474299987	-20.79999924	-0.3745	-20.7	-0.3521	-20.6	-0.3065	-20.6	-0.2917	-20.9	0	-20.8	0	-20.7	0	-20.6	0	-20.6	0
-20.80000114	-0.474799987	-20.69999886	-0.3728	-20.6	-0.3469	-20.5	-0.3016	-20.5	-0.2882	-20.8	0	-20.7	0	-20.6	0	-20.5	0	-20.5	0
-20.70000076	-0.479800008	-20.59999847	-0.3688	-20.5	-0.3478	-20.4	-0.298	-20.4	-0.2826	-20.7	0	-20.6	0	-20.5	0	-20.4	0	-20.4	0
-20.60000229	-0.472699982	-20.5	-0.3658	-20.4	-0.3433	-20.3	-0.2947	-20.3	-0.2701	-20.6	0	-20.5	0	-20.4	0	-20.3	0	-20.3	0
-20.50000191	-0.467799969	-20.39999962	-0.3604	-20.3	-0.3388	-20.2	-0.2871	-20.2	-0.2588	-20.5	0	-20.4	0	-20.3	0	-20.2	0	-20.2	0
-20.40000153	-0.460999996	-20.29999924	-0.3578	-20.2	-0.3351	-20.1	-0.2825	-20.1	-0.2552	-20.4	0	-20.3	0	-20.2	0	-20.1	0	-20.1	0
-20.30000114	-0.45830001	-20.19999886	-0.3534	-20.1	-0.3303	-20	-0.286	-20	-0.2534	-20.3	0	-20.2	0	-20.1	0	-20	0	-20	0
-20.20000076	-0.454099985	-20.09999847	-0.3451	-20	-0.3307	-19.9	-0.2827	-19.9	-0.2474	-20.2	0	-20.1	0	-20	0	-19.9	0	-19.9	0
-20.10000229	-0.44400007	-20	-0.3401	-19.9	-0.3285	-19.8	-0.2791	-19.8	-0.2454	-20.1	0	-20	0	-19.9	0	-19.8	0	-19.8	0
-20.00000191	-0.44100001	-19.89999962	-0.3353	-19.8	-0.3327	-19.7	-0.2757	-19.7	-0.2415	-20	0	-19.9	0	-19.8	0	-19.7	0	-19.7	0
-19.90000153	-0.435699997	-19.79999924	-0.3413	-19.7	-0.3339	-19.6	-0.2809	-19.6	-0.2447	-19.9	0	-19.8	0	-19.7	0	-19.6	0	-19.6	0
-19.80000114	-0.434400002	-19.69999886	-0.3391	-19.6	-0.3356	-19.5	-0.2866	-19.5	-0.2498	-19.8	0	-19.7	0	-19.6	0	-19.5	0	-19.5	0
-19.70000076	-0.430799999	-19.59999847	-0.3386	-19.5	-0.3348	-19.4	-0.2782	-19.4	-0.2466	-19.7	0	-19.6	0	-19.5	0	-19.4	0	-19.4	0
-19.60000229	-0.43069994	-19.5	-0.3358	-19.4	-0.3291	-19.3	-0.2652	-19.3	-0.2339	-19.6	0	-19.5	0	-19.4	0	-19.3	0	-19.3	0
-19.50000191	-0.428099932	-19.39999962	-0.3315	-19.3	-0.3198	-19.2	-0.2609	-19.2	-0.226	-19.5	0	-19.4	0	-19.3	0	-19.2	0	-19.2	0
-19.40000153	-0.417199991	-19.29999924	-0.3194	-19.2	-0.3148	-19.1	-0.2601	-19.1	-0.2257	-19.4	0	-19.3	0	-19.2	0	-19.1	0	-19.1	0
-19.30000114	-0.408800012	-19.19999886	-0.3105	-19.1	-0.3105	-19	-0.2549	-19	-0.2174	-19.3	0	-19.2	0	-19.1	0	-19	0	-19	0
-19.20000076	-0.39980001	-19.09999847	-0.3038	-19	-0.3064	-18.9	-0.2495	-18.9	-0.2122	-19.2	0	-19.1	0	-19	0	-18.9	0	-18.9	0
-19.10000229	-0.395000032	-19	-0.2982	-18.9	-0.3053	-18.8	-0.2394	-18.8	-0.2139	-19.1	0	-19	0	-18.9	0	-18.8	0	-18.8	0
-19.00000191	-0.389500093	-18.89999962	-0.2927	-18.8	-0.2987	-18.7	-0.2311	-18.7	-0.2099	-19	0	-18.9	0	-18.8	0	-18.7	0	-18.7	0
-18.90000153	-0.386699994	-18.79999924	-0.2882	-18.7	-0.2968	-18.6	-0.232	-18.6	-0.2095	-18.9	0	-18.8	0	-18.7	0	-18.6	0	-18.6	0
-18.80000114	-0.375399999	-18.69999886	-0.2811	-18.6	-0.2989	-18.5	-0.2322	-18.5	-0.2066	-18.8	0	-18.7	0	-18.6	0	-18.5	0	-18.5	0
-18.70000076	-0.370600009	-18.59999847	-0.2847	-18.5	-0.2937	-18.4	-0.2287	-18.4	-0.1948	-18.7	0	-18.6	0	-18.5	0	-18.4	0	-18.4	0
-18.60000229	-0.369799976	-18.5	-0.2839	-18.4	-0.2862	-18.3	-0.2267	-18.3	-0.1887	-18.6	0	-18.5	0	-18.4	0	-18.3	0	-18.3	0
-18.50000191	-0.364300022	-18.39999962	-0.2787	-18.3	-0.2806	-18.2	-0.2242	-18.2	-0.1832	-18.5	0	-18.4	0	-18.3	0	-18.2	0	-18.2	0
-18.40000153	-0.355800001	-18.29999924	-0.2722	-18.2	-0.2753	-18.1	-0.218	-18.1	-0.1678	-18.4	0	-18.3	0	-18.2	0	-18.1	0	-18.1	0
-18.30000114	-0.345799996	-18.19999886	-0.267	-18.1	-0.2667	-18	-0.2156	-18	-0.1602	-18.3	0	-18.2	0	-18.1	0	-18	0	-18	0
-18.20000076	-0.342300008	-18.09999847	-0.2537	-18	-0.2597	-17.9	-0.2157	-17.9	-0.1588	-18.2	0	-18.1	0	-18	0	-17.9	0	-17.9	0
-18.10000229	-0.334800128	-18	-0.2478	-17.9	-0.2582	-17.8	-0.2128	-17.8	-0.1578	-18.1	0	-18	0	-17.9	0	-17.8	0	-17.8	0
-18.00000191	-0.328100038	-17.89999962	-0.2517	-17.8	-0.2544	-17.7	-0.2136	-17.7	-0.1553	-18	0	-17.9	0	-17.8	0	-17.7	0	-17.7	0
-17.90000153	-0.324999992	-17.79999924	-0.2514	-17.7	-0.2494	-17.6	-0.2195	-17.6	-0.1608	-17.9	0	-17.8	0	-17.7	0	-17.6	0	-17.6	0
-17.80000114	-0.327399988	-17.69999886	-0.2421	-17.6	-0.2492	-17.5	-0.2175	-17.5	-0.1588	-17.8	0	-17.7	0	-17.6	0	-17.5	0	-17.5	0
-17.70000076	-0.319699999	-17.59999847	-0.2452	-17.5	-0.2394	-17.4	-0.2124	-17.4	-0.1508	-17.7	0	-17.6	0	-17.5	0	-17.4	0	-17.4	0
-17.60000229	-0.320399953	-17.5	-0.2396	-17.4	-0.2298	-17.3	-0.2097	-17.3	-0.153	-17.6	0	-17.5	0	-17.4	0	-17.3	0	-17.3	0
-17.50000191	-0.315300146	-17.39999962	-0.2274	-17.3	-0.2273	-17.2	-0.2003	-17.2	-0.1459	-17.5	0	-17.4	0	-17.3	0	-17.2	0	-17.2	0
-17.40000153	-0.304899986	-17.29999924	-0.2279	-17.2	-0.2192	-17.1	-0.1878	-17.1	-0.1413	-17.4	0	-17.3	0	-17.2	0	-17.1	0	-17.1	0
-17.30000114	-0.303000011	-17.19999886	-0.2184	-17.1	-0.2107	-17	-0.1903	-17	-0.1449	-17.3	0	-17.2	0	-17.1	0	-17	0	-17	0
-17.20000076	-0.300399998	-17.09999847	-0.2087	-17	-0.2155	-16.9	-0.1854	-16.9	-0.1379	-17.2	0	-17.1	0	-17	0	-16.9	0	-16.9	0
-17.10000229	-0.289800225	-17	-0.2147	-16.9	-0.21	-16.8	-0.1743	-16.8	-0.1335	-17.1	0	-17	0	-16.9	0	-16.8	0	-16.8	0
-17.00000191	-0.293900003	-16.89999962	-0.2184	-16.8	-0.2087	-16.7	-0.1717	-16.7	-0.1353	-17	0	-16.9	0	-16.8	0	-16.7	0	-16.7	0
-16.90000153	-0.286600006	-16.79999924	-0.2149	-16.7	-0.2079	-16.6	-0.1726	-16.6	-0.1344	-16.9	0	-16.8	0	-16.7	0	-16.6	0	-16.6	0
-16.80000114	-0.277800001	-16.69999886	-0.2122	-16.6	-0.2091	-16.5	-0.1837	-16.5	-0.1475	-16.8	0	-16.7	0	-16.6	0	-16.5	0	-16.5	0
-16.70000076	-0.284100006	-16.59999847	-0.2064	-16.5	-0.2147	-16.4	-0.1807	-16.4	-0.1548	-16.7	0	-16.6	0	-16.5	0	-16.4	0	-16.4	0
-16.60000229	-0.284500081	-16.5	-0.2119	-16.4	-0.2122	-16.3	-0.185	-16.3	-0.1465	-16.6	0	-16.5	0	-16.4	0	-16.3	0	-16.3	0
-16.50000191	-0.29509992	-16.39999962	-0.2085	-16.3	-0.2079	-16.2	-0.1878	-16.2	-0.1365	-16.5	0	-16.4	0	-16.3	0	-16.2	0	-16.2	0
-16.40000153	-0.299199985	-16.29999924	-0.2026	-16.2	-0.2126	-16.1	-0.1846	-16.1	-0.1412	-16.4	0	-16.3	0	-16.2	0	-16.1	0	-16.1	0
-16.30000114	-0.297899989	-16.19999886	-0.1889	-16.1	-0.2075	-16	-0.1877	-16	-0.1472	-16.3	0	-16.2	0	-16.1	0	-16	0	-16	0
-16.20000076	-0.298100003	-16.09999847	-0.1903	-16	-0.213	-15.9	-0.1799	-15.9	-0.1513	-16.2	0	-16.1	0	-16	0	-15.9	0	-15.9	0

-16.10000229	-0.293100156	-16	-0.1931	-15.9	-0.2129	-15.8	-0.1806	-15.8	-0.1473	-16.1	0	-16	0	-15.9	0	-15.8	0	-15.8	0
-16.00000191	-0.278600172	-15.89999962	-0.1864	-15.8	-0.2207	-15.7	-0.1651	-15.7	-0.1422	-16	0	-15.9	0	-15.8	0	-15.7	0	-15.7	0
-15.90000153	-0.274099999	-15.79999924	-0.1804	-15.7	-0.2127	-15.6	-0.1526	-15.6	-0.1372	-15.9	0	-15.8	0	-15.7	0	-15.6	0	-15.6	0
-15.80000114	-0.266700006	-15.69999886	-0.1661	-15.6	-0.2077	-15.5	-0.1424	-15.5	-0.1299	-15.8	0	-15.7	0	-15.6	0	-15.5	0	-15.5	0
-15.70000076	-0.24780001	-15.59999847	-0.1522	-15.5	-0.1967	-15.4	-0.1357	-15.4	-0.1255	-15.7	0	-15.6	0	-15.5	0	-15.4	0	-15.4	0
-15.60000229	-0.236700285	-15.5	-0.1421	-15.4	-0.1903	-15.3	-0.1329	-15.3	-0.1195	-15.6	0	-15.5	0	-15.4	0	-15.3	0	-15.3	0
-15.50000191	-0.227800225	-15.39999962	-0.1306	-15.3	-0.1824	-15.2	-0.134	-15.2	-0.1151	-15.5	0	-15.4	0	-15.3	0	-15.2	0	-15.2	0
-15.40000153	-0.222900005	-15.29999924	-0.1256	-15.2	-0.1812	-15.1	-0.1379	-15.1	-0.1148	-15.4	0	-15.3	0	-15.2	0	-15.1	0	-15.1	0
-15.30000114	-0.2154	-15.19999886	-0.1228	-15.1	-0.1814	-15	-0.1379	-15	-0.1103	-15.3	0	-15.2	0	-15.1	0	-15	0	-15	0
-15.20000076	-0.213199999	-15.09999847	-0.1226	-15	-0.177	-14.9	-0.1397	-14.9	-0.1054	-15.2	0	-15.1	0	-15	0	-14.9	0	-14.9	0
-15.10000229	-0.215800012	-15	-0.1162	-14.9	-0.1761	-14.8	-0.1374	-14.8	-0.1054	-15.1	0	-15	0	-14.9	0	-14.8	0	-14.8	0
-15.00000191	-0.21410007	-14.89999962	-0.1148	-14.8	-0.1738	-14.7	-0.1356	-14.7	-0.1024	-15	0	-14.9	0	-14.8	0	-14.7	0	-14.7	0
-14.90000153	-0.209999999	-14.79999924	-0.1142	-14.7	-0.1667	-14.6	-0.1356	-14.6	-0.0952	-14.9	0	-14.8	0	-14.7	0	-14.6	0	-14.6	0
-14.80000114	-0.203000001	-14.69999886	-0.1073	-14.6	-0.1622	-14.5	-0.1274	-14.5	-0.0877	-14.8	0	-14.7	0	-14.6	0	-14.5	0	-14.5	0
-14.70000076	-0.196999995	-14.59999847	-0.1082	-14.5	-0.1568	-14.4	-0.1275	-14.4	-0.0846	-14.7	0	-14.6	0	-14.5	0	-14.4	0	-14.4	0
-14.60000229	-0.196300026	-14.5	-0.1058	-14.4	-0.1537	-14.3	-0.1291	-14.3	-0.0828	-14.6	0	-14.5	0	-14.4	0	-14.3	0	-14.3	0
-14.50000191	-0.190500093	-14.39999962	-0.1033	-14.3	-0.1525	-14.2	-0.1362	-14.2	-0.0848	-14.5	0	-14.4	0	-14.3	0	-14.2	0	-14.2	0
-14.40000153	-0.188900005	-14.29999924	-0.106	-14.2	-0.1526	-14.1	-0.1396	-14.1	-0.085	-14.4	0	-14.3	0	-14.2	0	-14.1	0	-14.1	0
-14.30000114	-0.187900003	-14.19999886	-0.1114	-14.1	-0.154	-14	-0.1353	-14	-0.08	-14.3	0	-14.2	0	-14.1	0	-14	0	-14	0
-14.20000076	-0.197300004	-14.09999847	-0.1171	-14	-0.1458	-13.9	-0.138	-13.9	-0.0757	-14.2	0	-14.1	0	-14	0	-13.9	0	-13.9	0
-14.10000229	-0.208699934	-14	-0.1206	-13.9	-0.1483	-13.8	-0.1264	-13.8	-0.074	-14.1	0	-14	0	-13.9	0	-13.8	0	-13.8	0
-14.00000191	-0.201200028	-13.89999962	-0.1191	-13.8	-0.147	-13.7	-0.1251	-13.7	-0.075	-14	0	-13.9	0	-13.8	0	-13.7	0	-13.7	0
-13.90000153	-0.198000001	-13.79999924	-0.1155	-13.7	-0.1472	-13.6	-0.126	-13.6	-0.0779	-13.9	0	-13.8	0	-13.7	0	-13.6	0	-13.6	0
-13.80000114	-0.197899993	-13.69999886	-0.1176	-13.6	-0.1434	-13.5	-0.1317	-13.5	-0.0778	-13.8	0	-13.7	0	-13.6	0	-13.5	0	-13.5	0
-13.70000076	-0.200600002	-13.59999847	-0.1158	-13.5	-0.1382	-13.4	-0.1275	-13.4	-0.0677	-13.7	0	-13.6	0	-13.5	0	-13.4	0	-13.4	0
-13.60000229	-0.192599978	-13.5	-0.1071	-13.4	-0.1286	-13.3	-0.1189	-13.3	-0.0596	-13.6	0	-13.5	0	-13.4	0	-13.3	0	-13.3	0
-13.50000191	-0.192200004	-13.39999962	-0.1023	-13.3	-0.1231	-13.2	-0.111	-13.2	-0.0494	-13.5	0	-13.4	0	-13.3	0	-13.2	0	-13.2	0
-13.40000153	-0.184899999	-13.29999924	-0.1058	-13.2	-0.1198	-13.1	-0.1071	-13.1	-0.0436	-13.4	0	-13.3	0	-13.2	0	-13.1	0	-13.1	0
-13.30000114	-0.165199995	-13.19999886	-0.1073	-13.1	-0.1182	-13	-0.0976	-13	-0.0352	-13.3	0	-13.2	0	-13.1	0	-13	0	-13	0
-13.20000076	-0.1443	-13.09999847	-0.1049	-13	-0.114	-12.9	-0.0891	-12.9	-0.0237	-13.2	0	-13.1	0	-13	0	-12.9	0	-12.9	0
-13.10000229	-0.132400157	-13	-0.097	-12.9	-0.1151	-12.8	-0.0786	-12.8	-0.0147	-13.1	0	-13	0	-12.9	0	-12.8	0	-12.8	0
-13.00000191	-0.118300218	-12.89999962	-0.0853	-12.8	-0.1186	-12.7	-0.0769	-12.7	-0.014	-13	0	-12.9	0	-12.8	0	-12.7	0	-12.7	0
-12.90000153	-0.104200004	-12.79999924	-0.0753	-12.7	-0.1227	-12.6	-0.0812	-12.6	-0.0126	-12.9	0	-12.8	0	-12.7	0	-12.6	0	-12.6	0
-12.80000114	-0.092899994	-12.69999886	-0.0743	-12.6	-0.119	-12.5	-0.0878	-12.5	-0.0186	-12.8	0	-12.7	0	-12.6	0	-12.5	0	-12.5	0
-12.70000076	-0.088299998	-12.59999847	-0.0705	-12.5	-0.1136	-12.4	-0.0861	-12.4	-0.015	-12.7	0	-12.6	0	-12.5	0	-12.4	0	-12.4	0
-12.60000229	-0.089899993	-12.5	-0.0807	-12.4	-0.1058	-12.3	-0.085	-12.3	-0.0097	-12.6	0	-12.5	0	-12.4	0	-12.3	0	-12.3	0
-12.50000191	-0.09699988	-12.39999962	-0.0824	-12.3	-0.0988	-12.2	-0.0862	-12.2	-0.0071	-12.5	0	-12.4	0	-12.3	0	-12.2	0	-12.2	0
-12.40000153	-0.098399994	-12.29999924	-0.0847	-12.2	-0.0939	-12.1	-0.0804	-12.1	-0.0066	-12.4	0	-12.3	0	-12.2	0	-12.1	0	-12.1	0
-12.30000114	-0.100500002	-12.19999886	-0.0842	-12.1	-0.0819	-12	-0.0715	-12	-0.001	-12.3	0	-12.2	0	-12.1	0	-12	0	-12	0
-12.20000076	-0.102500005	-12.09999847	-0.0767	-12	-0.073	-11.9	-0.0701	-11.9	0.0004	-12.2	0	-12.1	0	-12	0	-11.9	0	-11.9	0
-12.10000229	-0.093800036	-12	-0.0734	-11.9	-0.0744	-11.8	-0.0734	-11.8	-0.0015	-12.1	0	-12	0	-11.9	0	-11.8	0	-11.8	0
-12.00000191	-0.087800032	-11.89999962	-0.0737	-11.8	-0.0729	-11.7	-0.0718	-11.7	0.004	-12	0	-11.9	0	-11.8	0	-11.7	0	-11.7	0
-11.90000153	-0.090599996	-11.79999924	-0.0755	-11.7	-0.0679	-11.6	-0.0656	-11.6	0.005	-11.9	0	-11.8	0	-11.7	0	-11.6	0	-11.6	0
-11.80000114	-0.090500003	-11.69999886	-0.0696	-11.6	-0.0691	-11.5	-0.0543	-11.5	0.0109	-11.8	0	-11.7	0	-11.6	0	-11.5	0	-11.5	0
-11.70000076	-0.093599994	-11.59999847	-0.0662	-11.5	-0.0731	-11.4	-0.0543	-11.4	0.0047	-11.7	0	-11.6	0	-11.5	0	-11.4	0	-11.4	0
-11.60000229	-0.093699939	-11.5	-0.0652	-11.4	-0.077	-11.3	-0.0558	-11.3	-0.003	-11.6	0	-11.5	0	-11.4	0	-11.3	0	-11.3	0
-11.50000191	-0.090099977	-11.39999962	-0.0689	-11.3	-0.0863	-11.2	-0.0498	-11.2	-0.0065	-11.5	0	-11.4	0	-11.3	0	-11.2	0	-11.2	0
-11.40000153	-0.095500001	-11.29999924	-0.0732	-11.2	-0.0841	-11.1	-0.0457	-11.1	-0.0073	-11.4	0	-11.3	0	-11.2	0	-11.1	0	-11.1	0
-11.30000114	-0.090999994	-11.19999886	-0.0736	-11.1	-0.0817	-11	-0.0429	-11	-0.0086	-11.3	0	-11.2	0	-11.1	0	-11	0	-11	0
-11.20000076	-0.091699997	-11.09999847	-0.0711	-11	-0.0763	-10.9	-0.0363	-10.9	-0.0058	-11.2	0	-11.1	0	-11	0	-10.9	0	-10.9	0
-11.10000229	-0.087400059	-11	-0.0654	-10.9	-0.07	-10.8	-0.0302	-10.8	-0.0062	-11.1	0	-11	0	-10.9	0	-10.8	0	-10.8	0
-11.00000191	-0.083400089	-10.89999962	-0.0601	-10.8	-0.059	-10.7	-0.0212	-10.7	6.38E-08	-11	0	-10.9	0	-10.8	0	-10.7	0	-10.7	0
-10.90000153	-0.077400001	-10.79999924	-0.0529	-10.7	-0.0479	-10.6	-0.0091	-10.6	0.0035	-10.9	0	-10.8	0	-10.7	0	-10.6	0	-10.6	0
-10.80000114	-0.071700001	-10.69999886	-0.0483	-10.6	-0.0457	-10.5	-0.0011	-10.5	0.0099	-10.8	0	-10.7	0	-10.6	0	-10.5	0	-10.5	0
-10.70000076	-0.063999996	-10.59999847	-0.0476	-10.5	-0.0483	-10.4	0.0038	-10.4	0.0117	-10.7	0	-10.6	0	-10.5	0	-10.4	0	-10.4	0
-10.60000229	-0.065200064	-10.5	-0.0458	-10.4	-0.0441	-10.3	0.0071	-10.3	0.0161	-10.6	0	-10.5	0	-10.4	0	-10.3	0	-10.3	0
-10.50000191	-0.060000087	-10.39999962	-0.0429	-10.3	-0.0385	-10.2	0.0095	-10.2	0.0159	-10.5	0	-10.4	0	-10.3	0	-10.2	0	-10.2	0
-10.40000153	-0.058900002	-10.29999924	-0.0402	-10.2	-0.0367	-10.1	0.0175	-10.1	0.0191	-10.4	0	-10.3	0	-10.2	0	-10.1	0	-10.1	0
-10.30000114	-0.0583	-10.19999886	-0.0366	-10.1	-0.0298	-9.999998	0.0188	-9.999998	0.0227	-10.3	0	-10.2	0	-10.1	0	-10	0	-10	0
-10.20000076	-0.055300005	-10.09999847	-0.0312	-10	-0.0258	-9.899998	0.0166	-9.899998	0.0171	-10.2	0	-10.1	0	-10	0	-9.900002	0	-9.900002	0
-10.10000229	-0.04570009	-10	-0.0268	-9.900002	-0.0279	-9.799999	0.0129	-9.799999	0.0178	-10.1	0	-10	0	-9.900002	0	-9.800001	0	-9.800001	0
-10.00000191	-0.042700007	-9.899999619	-0.0284	-9.800001	-0.0329	-9.699999	0.0064	-9.699999	0.0118	-10	0	-9.900002	0	-9.800001	0	-9.700001	0	-9.700001	0
-9.900001526	-0.045999999	-9.799999237	-0.0321	-9.700001	-0.0328	-9.599998	0.0048	-9.599998	0.0103	-9.900002	0	-9.800001	0	-9.700001	0	-9.600002	0	-9.600002	0
-9.800001144	-0.0475	-9.699998856	-0.0375	-9.6	-0.033	-9.499998	-0.0039	-9.499998	0.0042	-9.800001	0	-9.700001	0	-9.600002	0	-9.500002	0	-9.500002	0
-9.700000763	-0.045000005	-9.599998474	-0.0391	-9.5	-0.0316	-9.399998	-0.0088	-9.399998	-0.0037	-9.700001	0	-9.600002	0	-9.500002	0	-9.400002	0	-9.400002	0
-9.600002289	-0.041999945	-9.5	-0.0448	-9.400002	-0.0341	-9.299999	-0.0181	-9.299999	-0.0115	-9.600002	0	-9.500002	0	-9.400002	0	-9.300001	0	-9.300001	0
-9.500001907	-0.04869987	-9.399999619	-0.042	-9.300001	-0.033	-9.199999	-0.0265	-9.199999	-0.0166	-9.500002	0	-9.400002	0	-9.300001	0	-9.200001	0	-9.200001	0
-9.400001526	-0.0533	-9.299999237	-0.0454	-9.200001	-0.0349	-9.099998	-0.0237	-9.099998	-0.0158	-9.400002	0	-9.300001	0	-9.200001	0	-9.100002	0	-9.100002	0
-9.300001144	-0.055000003	-9.199998856	-0.0484	-9.1	-0.0336	-8.999998	-0.0168	-8.999998	-0.0197	-9.300001	0	-9.200001	0	-9.100002	0	-9.000002	0	-9.000002	0
-9.200000763	-0.057500003	-9.099998474	-0.0467	-9	-0.0302	-8.899998	-0.0158	-8.899998	-0.0163	-9.200001	0	-9.100002	0	-9.000002	0	-8.900002	0	-8.900002	0
-9.100002289	-0.062700043	-9	-0.0504	-8.900002	-0.0265	-8.799999	-0.0162	-8.799999	-0.0163	-9.100002	0	-9.000002	0	-8.900002	0	-8.800001	0	-8.800001	0
-9.000001907	-0.062800064	-8.899999619	-0.0507	-8.800001	-0.0365	-8.699999	-0.0102	-8.699999	-0.0104	-9.000002	0	-8.900002	0	-8.800001	0	-8.700001	0	-8.700001	0
-8.900001526	-0.055299999	-8.799999237	-0.0517	-8.700001	-0.0432	-8.599998	-0.007	-8.599998	-0.0129	-8.900002	0	-8.800001	0	-8.700001	0	-8.600002	0	-8.600002	0
-8.800001144	-0.055900003	-8.699998856	-0.0483	-8.6	-0.0476	-8.499998	-0.0077	-8.499998	-0.0191	-8.800001	0	-8.700001	0	-8.600002	0	-8.500002	0	-8.500002	0
-8.700000763	-0.056699995	-8.599998474	-0.0498	-8.5	-0.0379	-8.399998	-0.0035	-8.399998	-0.0198	-8.700001	0	-8.600002	0	-8.500002	0	-8.400002	0	-8.400002	0
-8.600002289	-0.054200036	-8.5	-0.0553	-8.400002	-0.0404	-8.299999	-0.0024	-8.299999	-0.0139	-8.600002	0	-8.500002	0	-8.400002	0	-8.300001	0	-8.300001	0
-8.500001907	-0.055000048	-8.399999619	-0.0577	-8.300001	-0.0322	-8.199999	-0.0009	-8.199999	-0.0118	-8.500002	0	-8.400002	0	-8.300001	0	-8.200001	0	-8.200001	0
-8.400001526	-0.058000005	-8.299999237	-0.0491	-8.200001	-0.0274	-8.099998	-0.0056	-8.099998	-0.017	-8.400002	0	-8.300001	0	-8.200001	0	-8.100002	0	-8.100002	0
-8.300001144	-0.056199998	-8.199998856	-0.0396	-8.1	-0.0256	-7.999998	-0.0036	-7.999998	-0.0159	-8.300001	0	-8.200001	0	-8.100002	0	-8.000002	0	-8.000002	0
-8.200000763	-0.053899997	-8.099998474	-0.0418	-8	-0.0292	-7.899998	0.0049	-7.899998	-0.0143	-8.200001	0	-8.100002	0	-8.000002	0	-7.900002	0	-7.900002	0
-8.100002289	-0.052300054	-8	-0.0415	-7.900002	-0.0287	-7.799999	0.0067	-7.799999	-0.0178	-8.100002	0	-8.000002	0	-7.900002	0	-7.800001	0	-7.800001	0
-8.000001907	-0.0535001	-7.899999619	-0.0378	-7.800001	-0.0267	-7.699999	0.0049	-7.699999	-0.0214	-8.000002	0	-7.900002	0	-7.800001	0	-7.700001	0	-7.700001	0
-7.900001526	-0.049999998	-7.799999237	-0.0365	-7.700001	-0.0294	-7.599998	0.0072	-7.599998	-0.017	-7.900002	0	-7.800001	0	-7.700001	0	-7.600002	0	-7.600002	0
-7.800001144	-0.045900002	-7.699998856	-0.0401	-7.6	-0.0282	-7.499998	0.0114	-7.499998	-0.0109	-7.800001	0	-7.700001	0	-7.600002	0	-7.500002	0	-7.500002	0
-7.700000763	-0.047699998	-7.599998474	-0.0352	-7.5	-0.023	-7.399998	0.0114	-7.399998	-0.0112	-7.700001	0	-7.600002	0	-7.500002	0	-7.400002	0	-7.400002	0
-7.600002289	-0.049400155	-7.5	-0.0292	-7.400002	-0.0244	-7.299999	0.0127	-7.299999	-0.0152	-7.600002	0	-7.500002	0	-7.400002	0	-7.300001	0	-7.300001	0
-7.500001907	-0.039900182	-7.399999619	-0.0275	-7.300001	-0.0266	-7.199999	0.0146	-7.199999	-0.0192	-7.500002	0	-7.400002	0	-7.300001	0	-7.200001	0	-7.200001	0
-7.400001526	-0.034900002	-7.299999237	-0.0283	-7.200001	-0.0274	-7.099998	0.0178	-7.099998	-0.0176	-7.400002	0	-7.300001	0	-7.200001	0	-7.100002	0	-7.100002	0
-7.300001144	-0.038000002	-7.199998856	-0.0265	-7.1	-0.0257	-6.999998	0.0171	-6.999998	-0.0178	-7.300001	0	-7.200001	0	-7.100002	0	-7.000002	0	-7.000002	0
-7.200000763	-0.034900002	-7.099998474	-0.0225	-7	-0.0245	-6.899998	0.0152	-6.899998	-0.0168	-7.200001	0	-7.100002	0	-7.000002	0	-6.900002	0	-6.900002	0
-7.100002289	-0.031300115	-7	-0.0174	-6.900002	-0.0235	-6.799999	0.0137	-6.799999	-0.0203	-7.100002	0	-7.000002	0	-6.900002	0	-6.800001	0	-6.800001	0
-7.000001907	-0.033100071	-6.899999619	-0.0172	-6.800001	-0.0224	-6.699999	0.013	-6.699999	-0.0186	-7.000002	0	-6.900002	0	-6.800001	0	-6.700001	0	-6.700001	0
-6.900001526	-0.034199999	-6.799999237	-0.0164	-6.700001	-0.0191	-6.599998	0.0119	-6.599998	-0.0197	-6.900002	0	-6.800001	0	-6.700001	0	-6.600002	0	-6.600002	0
-6.800001144	-0.031799998	-6.699998856	-0.0124	-6.6	-0.02	-6.499998	0.0125	-6.499998	-0.0214	-6.800001	0	-6.700001	0	-6.600002	0	-6.500002	0	-6.500002	0
-6.700000763	-0.031299998	-6.599998474	-0.0117	-6.5	-0.0172	-6.399998	0.0143	-6.399998	-0.0192	-6.700001	0	-6.600002	0	-6.500002	0	-6.400002	0	-6.400002	0
-6.600002289	-0.031300029	-6.5	-0.0108	-6.400002	-0.0107	-6.299999	0.0205	-6.299999	-0.0141	-6.600002	0	-6.500002	0	-6.400002	0	-6.300001	0	-6.300001	0
-6.500001907	-0.031400054	-6.399999619	-0.0067	-6.300001	-0.0096	-6.199999	0.0176	-6.199999	-0.014	-6.500002	0	-6.400002	0	-6.300001	0	-6.200001	0	-6.200001	0
-6.400001526	-0.0313	-6.299999237	-0.0025	-6.200001	-0.0094	-6.099998	0.0181	-6.099998	-0.0111	-6.400002	0	-6.300001	0	-6.200001	0	-6.100002	0	-6.100002	0
-6.300001144	-0.024399999	-6.199998856	-0.0032	-6.1	-0.0043	-5.999998	0.0149	-5.999998	-0.0149	-6.300001	0	-6.200001	0	-6.100002	0	-6.000002	0	-6.000002	0
-6.200000763	-0.0241	-6.099998474	-0.0032	-6	-0.0024	-5.899998	0.0137	-5.899998	-0.0145	-6.200001	0	-6.100002	0	-6.000002	0	-5.900002	0	-5.900002	0
-6.100002289	-0.022100001	-6	-0.0057	-5.900002	-0.0037	-5.799999	0.0108	-5.799999	-0.0105	-6.100002	0	-6.000002	0	-5.900002	0	-5.800003	0	-5.800003	0
-6.000001907	-0.020800029	-5.899999619	-0.0079	-5.800001	-0.0055	-5.699999	0.0132	-5.699999	-0.0078	-6.000002	0	-5.900002	0	-5.800003	0	-5.700001	0	-5.700001	0
-5.900001526	-0.020800001	-5.799999237	-0.0076	-5.700001	-0.0034	-5.599998	0.0128	-5.599998	-0.0019	-5.900002	0	-5.800003	0	-5.700001	0	-5.600002	0	-5.600002	0
-5.800003052	-0.018300026	-5.700000763	-0.0069	-5.600002	0.004	-5.5	0.0117	-5.5	-0.0005	-5.800003	0	-5.700001	0	-5.600002	0	-5.5	0	-5.5	0
-5.700000763	-0.017800001	-5.599998474	-0.0023	-5.5	0.0045	-5.399998	0.008	-5.399998	-0.0044	-5.700001	0	-5.600002	0	-5.5	0	-5.400002	0	-5.400002	0
-5.600002289	-0.0152	-5.5	-0.0015	-5.400002	0.0016	-5.299999	0.0092	-5.299999	-0.0019	-5.600002	0	-5.5	0	-5.400002	0	-5.300003	0	-5.300003	0
-5.5	-0.0109	-5.399997711	-0.0015	-5.299999	1E-04	-5.199997	0.0079	-5.199997	0.0008	-5.5	0	-5.400002	0	-5.300003	0	-5.200001	0	-5.200001	0
-5.400001526	-0.0089	-5.299999237	-0.0001	-5.200001	0.0027	-5.099998	0.004	-5.099998	-0.0002	-5.400002	0	-5.300003	0	-5.200001	0	-5.100002	0	-5.100002	0
-5.300003052	-0.006100152	-5.200000763	-0.0006	-5.100002	0.0019	-5	0	-5	0	-5.300003	0	-5.200001	0	-5.100002	0	-5	0	-5	0
-5.200000763	-0.0016	-5.099998474	0.0013	-5	0	-4.899998	0.003	-4.899998	0.003	-5.200001	0	-5.100002	0	-5	0	-4.900002	0	-4.900002	0
-5.100002289	0.0013	-5	0	-4.900002	0.0028	-4.799999	0.009	-4.799999	0.0064	-5.100002	0	-5	0	-4.900002	0	-4.800003	0	-4.800003	0
-5	0.0044	-4.899997711	0.0036	-4.799999	0.0085	-4.699997	0.0148	-4.699997	0.0146	-5	0	-4.900002	0	-4.800003	0	-4.700001	0	-4.700001	0
-4.900001526	0.0069	-4.799999237	0.0097	-4.700001	0.0165	-4.599998	0.0211	-4.599998	0.0215	-4.900002	0	-4.800003	0	-4.700001	0	-4.600002	0	-4.600002	0
-4.800003052	0.010899699	-4.700000763	0.0146	-4.600002	0.0197	-4.5	0.0213	-4.5	0.0233	-4.800003	0	-4.700001	0	-4.600002	0	-4.5	0	-4.5	0
-4.700000763	0.0164	-4.599998474	0.0219	-4.5	0.0259	-4.399998	0.0229	-4.399998	0.0255	-4.700001	0	-4.600002	0	-4.5	0	-4.400002	0	-4.400002	0

-4.600002289	0.020300001	-4.5	0.0261	-4.400002	0.0302	-4.299999	0.0231	-4.299999	0.03	-4.600002	0	-4.5	0	-4.400002	0	-4.300003	0	-4.300003	0
-4.5	0.021100001	-4.399997711	0.0247	-4.299999	0.0309	-4.199997	0.0176	-4.199997	0.0259	-4.5	0	-4.400002	0	-4.300003	0	-4.200001	0	-4.200001	0
-4.400001526	0.022800001	-4.299999237	0.0255	-4.200001	0.0287	-4.099998	0.0149	-4.099998	0.0213	-4.400002	0	-4.300003	0	-4.200001	0	-4.100002	0	-4.100002	0
-4.300003052	0.024199828	-4.200000763	0.0222	-4.100002	0.0292	-4	0.0158	-4	0.0187	-4.300003	0	-4.200001	0	-4.100002	0	-4	0	-4	0
-4.200000763	0.024300001	-4.099998474	0.021	-4	0.0308	-3.899998	0.0174	-3.899998	0.0195	-4.200001	0	-4.100002	0	-4	0	-3.900002	0	-3.900002	0
-4.100002289	0.026000001	-4	0.0248	-3.900002	0.0313	-3.799999	0.0172	-3.799999	0.0175	-4.100002	0	-4	0	-3.900002	0	-3.800003	0	-3.800003	0
-4	0.030500001	-3.899997711	0.0245	-3.799999	0.0374	-3.699997	0.0181	-3.699997	0.0174	-4	0	-3.900002	0	-3.800003	0	-3.700001	0	-3.700001	0
-3.900001526	0.035000001	-3.799999237	0.0296	-3.700001	0.043	-3.599998	0.0179	-3.599998	0.0215	-3.900002	0	-3.800003	0	-3.700001	0	-3.600002	0	-3.600002	0
-3.800003052	0.035899842	-3.700000763	0.0361	-3.600002	0.0445	-3.5	0.0206	-3.5	0.0309	-3.800003	0	-3.700001	0	-3.600002	0	-3.5	0	-3.5	0
-3.700000763	0.0406	-3.599998474	0.0319	-3.5	0.0416	-3.399998	0.0298	-3.399998	0.0327	-3.700001	0	-3.600002	0	-3.5	0	-3.400002	0	-3.400002	0
-3.600002289	0.046999999	-3.5	0.0325	-3.400002	0.0452	-3.299999	0.0304	-3.299999	0.0353	-3.600002	0	-3.5	0	-3.400002	0	-3.300003	0	-3.300003	0
-3.5	0.051699998	-3.399997711	0.0452	-3.299999	0.0486	-3.199997	0.0357	-3.199997	0.034	-3.5	0	-3.400002	0	-3.300003	0	-3.200001	0	-3.200001	0
-3.400001526	0.058400003	-3.299999237	0.0474	-3.200001	0.0497	-3.099998	0.0358	-3.099998	0.034	-3.400002	0	-3.300003	0	-3.200001	0	-3.100002	0	-3.100002	0
-3.300003052	0.060299816	-3.200000763	0.0549	-3.100002	0.049	-3	0.0181	-3	0.0167	-3.300003	0	-3.200001	0	-3.100002	0	-3	0	-3	0
-3.200000763	0.064000001	-3.099998474	0.0656	-3	0.0399	-2.899998	0.004	-2.899998	0.0104	-3.200001	0	-3.100002	0	-3	0	-2.900002	0	-2.900002	0
-3.100002289	0.071600002	-3	0.0658	-2.900002	0.032	-2.799999	0.0089	-2.799999	0.008	-3.100002	0	-3	0	-2.900002	0	-2.800003	0	-2.800003	0
-3	0.066400001	-2.899997711	0.0608	-2.799999	0.0291	-2.699997	0.0114	-2.699997	0.0135	-3	0	-2.900002	0	-2.800003	0	-2.700001	0	-2.700001	0
-2.900001526	0.061799997	-2.799999237	0.0636	-2.700001	0.0344	-2.599998	0.013	-2.599998	0.0162	-2.900002	0	-2.800003	0	-2.700001	0	-2.600002	0	-2.600002	0
-2.800003052	0.066099966	-2.700000763	0.069	-2.600002	0.0359	-2.5	0.0114	-2.5	0.0137	-2.800003	0	-2.700001	0	-2.600002	0	-2.5	0	-2.5	0
-2.700000763	0.068700002	-2.599998474	0.074	-2.5	0.0367	-2.399998	0.0043	-2.399998	0.009	-2.700001	0	-2.600002	0	-2.5	0	-2.400002	0	-2.400002	0
-2.600002289	0.0698	-2.5	0.0744	-2.400002	0.0337	-2.299999	0.0027	-2.299999	0.0142	-2.600002	0	-2.5	0	-2.400002	0	-2.300003	0	-2.300003	0
-2.5	0.071	-2.399997711	0.0764	-2.299999	0.0323	-2.199997	0.0089	-2.199997	0.0223	-2.5	0	-2.400002	0	-2.300003	0	-2.200001	0	-2.200001	0
-2.400001526	0.0759	-2.299999237	0.0801	-2.200001	0.0387	-2.099998	0.0152	-2.099998	0.0291	-2.400002	0	-2.300003	0	-2.200001	0	-2.100002	0	-2.100002	0
-2.300003052	0.079699918	-2.200000763	0.0833	-2.100002	0.0407	-2	0.0241	-2	0.036	-2.300003	0	-2.200001	0	-2.100002	0	-2	0	-2	0
-2.200000763	0.085299999	-2.099998474	0.0865	-2	0.0484	-1.899998	0.0263	-1.899998	0.043	-2.200001	0	-2.100002	0	-2	0	-1.900002	0	-1.900002	0
-2.100002289	0.095000001	-2	0.087	-1.900002	0.061	-1.799999	0.0274	-1.799999	0.0423	-2.100002	0	-2	0	-1.900002	0	-1.800003	0	-1.800003	0
-2	0.097799998	-1.899997711	0.0938	-1.799999	0.0653	-1.699997	0.0303	-1.699997	0.0386	-2	0	-1.900002	0	-1.800003	0	-1.700001	0	-1.700001	0
-1.900001526	0.105299997	-1.799999237	0.1022	-1.700001	0.0625	-1.599998	0.025	-1.599998	0.0455	-1.900002	0	-1.800003	0	-1.700001	0	-1.600002	0	-1.600002	0
-1.800003052	0.114399849	-1.700000763	0.1193	-1.600002	0.0588	-1.5	0.0127	-1.5	0.0332	-1.800003	0	-1.700001	0	-1.600002	0	-1.5	0	-1.5	0
-1.700000763	0.121900003	-1.599998474	0.1311	-1.5	0.0443	-1.399998	0.0174	-1.399998	0.0282	-1.700001	0	-1.600002	0	-1.5	0	-1.400002	0	-1.400002	0
-1.600002289	0.131099999	-1.5	0.1267	-1.400002	0.0433	-1.299999	0.0186	-1.299999	0.0282	-1.600002	0	-1.5	0	-1.400002	0	-1.300003	0	-1.300003	0
-1.5	0.123300002	-1.399997711	0.1299	-1.299999	0.0433	-1.199997	0.0186	-1.199997	0.027	-1.5	0	-1.400002	0	-1.300003	0	-1.200001	0	-1.200001	0
-1.400001526	0.1298	-1.299999237	0.1482	-1.200001	0.0433	-1.099998	0.0186	-1.099998	0.0266	-1.400002	0	-1.300003	0	-1.200001	0	-1.100002	0	-1.100002	0
-1.300003052	0.1313	-1.200000763	0.1396	-1.100002	0.0443	-1	0.0186	-1	0.015	-1.300003	0	-1.200001	0	-1.100002	0	-1	0	-1	0
-1.200000763	0.1313	-1.099998474	0.1299	-1	0.0443	-0.899998	0.0186	-0.899998	-0.0092	-1.200001	0	-1.100002	0	-1	0	-0.900002	0	-0.900002	0
-1.100002289	0.1298	-1	0.1267	-0.900002	0.0644	-0.799999	0.0198	-0.799999	-0.0006	-1.100002	0	-1	0	-0.900002	0	-0.800003	0	-0.800003	0
-1	0.123300002	-0.899997711	0.1091	-0.799999	0.0641	-0.699997	0.0223	-0.699997	0.007999	-1	0	-0.900002	0	-0.800003	0	-0.700001	0	-0.700001	0
-0.900001526	0.122600002	-0.799999237	0.1091	-0.700001	0.0636	-0.599998	0.0246	-0.599998	0.015	-0.900002	0	-0.800003	0	-0.700001	0	-0.600002	0	-0.600002	0
-0.800003052	0.122600002	-0.700000763	0.1091	-0.600002	0.0505	-0.5	0.0246	-0.5	0.0266	-0.800003	0	-0.700001	0	-0.600002	0	-0.5	0	-0.5	0
-0.700000763	0.122600002	-0.599998474	0.1091	-0.5	0.0636	-0.399998	0.0283	-0.399998	0.027	-0.700001	0	-0.600002	0	-0.5	0	-0.400002	0	-0.400002	0
-0.600002289	0.122600002	-0.5	0.1091	-0.400002	0.0641	-0.299999	0.0297	-0.299999	0.0282	-0.600002	0	-0.5	0	-0.400002	0	-0.300003	0	-0.300003	0
-0.5	0.123300002	-0.399997711	0.1091	-0.299999	0.0644	-0.199997	0.0337	-0.199997	0.0282	-0.5	0	-0.400002	0	-0.300003	0	-0.200001	0	-0.200001	0
-0.400001526	0.1273	-0.299999237	0.1267	-0.200001	0.0683	-0.099998	0.0341	-0.099998	0.0315	-0.400002	0	-0.300003	0	-0.200001	0	-0.100002	0	-0.100002	0
-0.300003052	0.1298	-0.200000763	0.1299	-0.100002	0.0701	0	0.0364	0	0.0332	-0.300003	0	-0.200001	0	-0.100002	0	0	0	0	0
-0.200000763	0.1313	-0.099998474	0.1396	0	0.0727	0.100002	0.055599	0.100002	0.035698	-0.200001	0	-0.100002	0	0	0	0.099998	24.51	0.099998	44.43
-0.100002289	0.132700002	0	0.1482	0.099998	0.0746	0.200001	0.084998	0.200001	1.254422	-0.100002	0	0	0	0.099998	15.51	0.199997	24.60217	0.199997	44.61437
0	0.1328	0.100002289	0.1487	0.200001	0.0747	0.300003	2.493006	0.300003	12.79737	0	0	0.099998	7.893	0.199997	15.55609	0.299999	24.69422	0.299999	44.79853
0.099998474	0.133400003	0.200000763	0.1512	0.299999	0.0747	0.400002	12.64189	0.400002	22.77234	0.099998	4.321	0.199997	7.916045	0.299999	15.60211	0.399998	24.78612	0.399998	44.98247
0.199996948	0.133600001	0.299999237	5.282065	0.399998	6.530829	0.5	19.46801	0.5	28.93383	0.199997	4.332506	0.299999	7.93906	0.399998	15.64807	0.5	24.87789	0.5	45.16618
0.299999237	3.363062444	0.400001526	5.787715	0.5	11.33858	0.600002	22.29793	0.600002	33.27675	0.299999	4.343999	0.399998	7.962044	0.5	15.69397	0.599998	24.96953	0.599998	45.34967
0.399997711	3.40148196	0.5	5.832541	0.599998	11.41587	0.700001	22.59223	0.700001	36.48404	0.399998	4.35548	0.5	7.984999	0.599998	15.7398	0.699997	25.06103	0.699997	45.53294
0.5	3.429914495	0.600002289	5.859787	0.700001	11.70258	0.800003	22.85464	0.800003	39.32582	0.5	4.366949	0.599998	8.007922	0.699997	15.78557	0.799999	25.1524	0.799999	45.71598
0.599998474	3.445849775	0.700000763	6.063269	0.799999	11.95429	0.900002	22.94233	0.900002	41.17753	0.599998	4.378405	0.699997	8.030815	0.799999	15.83127	0.899998	25.24363	0.899998	45.8988
0.699996948	3.445849775	0.799999237	6.111809	0.899998	12.18903	1	23.09785	1	42.81351	0.699997	4.389848	0.799999	8.053678	0.899998	15.8769	1	25.33472	1	46.0814
0.799999237	3.445849775	0.900001526	6.142207	1	12.34625	1.100002	23.31548	1.100002	43.74649	0.799999	4.40128	0.899998	8.07651	1	15.92248	1.099998	25.42568	1.099998	46.26376
0.899997711	3.445849775	1	6.152409	1.099998	12.40557	1.200001	23.5547	1.200001	43.79145	0.899998	4.412699	1	8.099312	1.099998	15.96799	1.199997	25.5165	1.199997	46.4459
1	3.445849775	1.100002289	6.210356	1.200001	12.53496	1.300003	23.61511	1.300003	44.282	1	4.424106	1.099998	8.122083	1.199997	16.01343	1.299999	25.60719	1.299999	46.62781
1.099998474	3.464746147	1.200000763	6.327445	1.299999	12.57252	1.400002	23.62099	1.400002	44.28815	1.099998	4.435501	1.199997	8.144824	1.299999	16.05881	1.399998	25.69774	1.399998	46.80949
1.199996948	3.480053369	1.299999237	6.338421	1.399998	12.59637	1.5	23.69697	1.5	44.34759	1.199997	4.446883	1.299999	8.167535	1.399998	16.10412	1.5	25.78815	1.5	46.99095
1.299999237	3.498971439	1.400001526	6.365831	1.5	12.60631	1.600002	23.71789	1.600002	44.35655	1.299999	4.458253	1.399998	8.190215	1.5	16.14938	1.599998	25.87843	1.599998	47.17217
1.399997711	3.512512375	1.5	6.36839	1.599998	12.6429	1.700001	23.76234	1.700001	44.41859	1.399998	4.46961	1.5	8.212866	1.599998	16.19456	1.699997	25.96856	1.699997	47.35316
1.5	3.515766045	1.600002289	6.436719	1.700001	12.79366	1.800003	23.82902	1.800003	44.46014	1.5	4.480956	1.599998	8.235486	1.699997	16.23968	1.799999	26.05857	1.799999	47.53392
1.599998474	3.539234138	1.700000763	6.451821	1.799999	12.9151	1.900002	23.82902	1.900002	44.46427	1.599998	4.492288	1.699997	8.258075	1.799999	16.28474	1.899998	26.14843	1.899998	47.71444
1.699996948	3.548775902	1.799999237	6.436719	1.899998	13.017	2	24.16235	2	44.95664	1.699997	4.503609	1.799999	8.280636	1.899998	16.32973	2	26.23816	2	47.89473
1.799999237	3.572200865	1.900001526	6.581938	2	13.04856	2.100002	24.48605	2.100002	45.4803	1.799999	4.514918	1.899998	8.303165	2	16.37466	2.099998	26.32774	2.099998	48.07479
1.899997711	3.587852089	2	6.679554	2.099998	13.29934	2.200001	24.62471	2.200001	45.83401	1.899998	4.526214	2	8.325664	2.099998	16.41952	2.199997	26.41719	2.199997	48.2546
2	3.58786091	2.100002289	6.700685	2.200001	13.47666	2.300003	24.94393	2.300003	46.2299	2	4.537498	2.099998	8.348133	2.199997	16.46432	2.299999	26.50651	2.299999	48.43419
2.099998474	3.600249403	2.200000763	6.847474	2.299999	13.59126	2.400002	24.99989	2.400002	46.61623	2.099998	4.54877	2.199997	8.370572	2.299999	16.50906	2.399998	26.59568	2.399998	48.61354
2.199996948	3.637326619	2.299999237	7.103462	2.399998	13.73127	2.5	25.27675	2.5	47.08415	2.199997	4.560029	2.299999	8.392981	2.399998	16.55372	2.5	26.68471	2.5	48.79265
2.299999237	3.649853795	2.400001526	7.156058	2.5	14.02972	2.600002	25.36035	2.600002	47.56154	2.299999	4.571277	2.399998	8.415359	2.5	16.59833	2.599998	26.77361	2.599998	48.97152
2.399997711	3.705231702	2.5	7.179862	2.599998	14.09474	2.700001	25.80155	2.700001	47.97513	2.399998	4.582512	2.5	8.437709	2.599998	16.64287	2.699997	26.86236	2.699997	49.15014
2.5	3.86131794	2.600002289	7.211748	2.700001	14.27317	2.800003	26.01834	2.800003	48.3382	2.5	4.593735	2.599998	8.460027	2.699997	16.68735	2.799999	26.95098	2.799999	49.32853
2.599998474	3.832950416	2.700000763	7.336018	2.799999	14.36423	2.900002	26.1327	2.900002	48.60831	2.599998	4.604946	2.699997	8.482315	2.799999	16.73176	2.899998	27.03946	2.899998	49.50668
2.699996948	3.967307711	2.799999237	7.368971	2.899998	14.3623	3	26.24043	3	48.81345	2.699997	4.616144	2.799999	8.504574	2.899998	16.77611	3	27.1278	3	49.68459
2.799999237	4.051866909	2.900001526	7.37886	3	14.3607	3.100002	26.4116	3.100002	49.13431	2.799999	4.627331	2.899998	8.526803	3	16.82039	3.099998	27.216	3.099998	49.86224
2.899997711	3.986486446	3	7.364832	3.099998	14.36895	3.200001	26.6077	3.200001	49.40733	2.899998	4.638505	3	8.549002	3.099998	16.86461	3.199997	27.30405	3.199997	50.03966
3	3.894487117	3.100002289	7.370456	3.200001	14.5333	3.300003	26.85426	3.300003	49.75853	3	4.649668	3.099998	8.57117	3.199997	16.90876	3.299999	27.39197	3.299999	50.21683
3.099998474	3.804573037	3.200000763	7.395128	3.299999	14.77428	3.400002	27.05099	3.400002	50.13312	3.099998	4.660817	3.199997	8.593309	3.299999	16.95285	3.399998	27.47975	3.399998	50.39376
3.199996948	3.827874009	3.299999237	7.507582	3.399998	14.89468	3.5	27.16458	3.5	50.48156	3.199997	4.671955	3.299999	8.615418	3.399998	16.99688	3.5	27.56739	3.5	50.57044
3.299999237	3.946031215	3.400001526	7.567564	3.5	15.04076	3.600002	27.29638	3.600002	50.72573	3.299999	4.683081	3.399998	8.637497	3.5	17.04084	3.599998	27.65488	3.599998	50.74687
3.399997711	3.984505195	3.5	7.66063	3.599998	15.14963	3.700001	27.44292	3.700001	51.02548	3.399998	4.694195	3.5	8.659547	3.599998	17.08473	3.699997	27.74224	3.699997	50.92305
3.5	4.044586623	3.600002289	7.660619	3.700001	15.26333	3.800003	27.56548	3.800003	51.32759	3.5	4.705297	3.599998	8.681566	3.699997	17.12857	3.799999	27.82946	3.799999	51.09898
3.599998474	4.048516951	3.700000763	7.720126	3.799999	15.36387	3.900002	27.73351	3.900002	51.61665	3.599998	4.716386	3.699997	8.703555	3.799999	17.17233	3.899998	27.91653	3.899998	51.27466
3.699996948	4.089779602	3.799999237	7.773673	3.899998	15.48486	4	27.92066	4	51.86734	3.699997	4.727464	3.799999	8.725515	3.899998	17.21604	4	28.00346	4	51.45009
3.799999237	4.104558194	3.900001526	7.828091	4	15.58733	4.100002	28.04428	4.100002	52.11105	3.799999	4.73853	3.899998	8.747445	4	17.25968	4.099998	28.09025	4.099998	51.62527
3.899997711	4.151984398	4	7.899923	4.099998	15.68841	4.200001	28.21406	4.200001	52.42731	3.899998	4.749583	4	8.769346	4.099998	17.30325	4.199997	28.1769	4.199997	51.80019
4	4.182432709	4.100002289	7.925244	4.200001	15.81495	4.300003	28.34166	4.300003	52.67754	4	4.760625	4.099998	8.791216	4.199997	17.34676	4.299999	28.2634	4.299999	51.97487
4.099998474	4.176033272	4.200000763	7.995998	4.299999	15.88335	4.400002	28.51793	4.400002	52.95871	4.099998	4.771654	4.199997	8.813056	4.299999	17.39021	4.399998	28.34977	4.399998	52.14928
4.199996948	4.239309996	4.299999237	8.030743	4.399998	15.99539	4.5	28.66842	4.5	53.20815	4.199997	4.782671	4.299999	8.834868	4.399998	17.43359	4.5	28.43599	4.5	52.32344
4.299999237	4.238914221	4.400001526	8.099554	4.5	16.09166	4.600002	28.79326	4.600002	53.44424	4.299999	4.793677	4.399998	8.856649	4.5	17.4769	4.599998	28.52207	4.599998	52.49734
4.399997711	4.281941705	4.5	8.141945	4.599998	16.18269	4.700001	28.92582	4.700001	53.70242	4.399998	4.80467	4.5	8.878402	4.599998	17.52016	4.699997	28.608	4.699997	52.67099
4.5	4.29653013	4.600002289	8.185827	4.700001	16.27386	4.800003	29.06328	4.800003	53.93528	4.5	4.815652	4.599998	8.900123	4.699997	17.56334	4.799999	28.6938	4.799999	52.84438
4.599998474	4.322632011	4.700000763	8.257002	4.799999	16.37819	4.900002	29.21076	4.900002	54.19153	4.599998	4.826621	4.699997	8.921816	4.799999	17.60647	4.899998	28.77945	4.899998	53.0175
4.699996948	4.367892569	4.799999237	8.279917	4.899998	16.49392	5	29.343	5	54.43522	4.699997	4.837578	4.799999	8.943479	4.899998	17.64953	5	28.86496	5	53.19037
4.799999237	4.362936175	4.900001526	8.339067	5	16.58596	5.100002	29.48749	5.100002	54.67097	4.799999	4.848524	4.899998	8.965112	5	17.69252	5.099998	28.95032	5.099998	53.36298
4.899997711	4.412562334	5	8.388987	5.099998	16.68477	5.200001	29.61368	5.200001	54.90586	4.899998	4.859457	5	8.986717	5.099998	17.73545	5.199997	29.03554	5.199997	53.53532
5	4.444595839	5.100002289	8.443639	5.200001	16.78001	5.300003	29.73993	5.300003	55.13638	5	4.870379	5.099998	9.008291	5.199997	17.77832	5.299999	29.12062	5.299999	53.7074
5.099998474	4.466779824	5.200000763	8.491704	5.299999	16.85552	5.400002	29.87185	5.400002	55.35358	5.099998	4.881289	5.199997	9.029836	5.299999	17.82112	5.399998	29.20555	5.399998	53.87921
5.199996948	4.488734325	5.299999237	8.545806	5.399998	16.95259	5.5	30.01854	5.5	55.58881	5.199997	4.892186	5.299999	9.051352	5.399998	17.86386	5.5	29.29034	5.5	54.05077
5.299999237	4.522869805	5.400001526	8.583784	5.5	17.06099	5.600002	30.12993	5.600002	55.82864	5.299999	4.903073	5.399998	9.072837	5.5	17.90653	5.599998	29.37498	5.599998	54.22205
5.399997711	4.528693363	5.5	8.633899	5.599998	17.14563	5.700001	30.26491	5.700001	56.04599	5.399998	4.913946	5.5	9.094294	5.599998	17.94914	5.699997	29.45948	5.699997	54.39306
5.5	4.563428061	5.600002289	8.680625	5.700001	17.241	5.800003	30.39248	5.800003	56.26043	5.5	4.924809	5.599998	9.115721	5.699997	17.99169	5.799999	29.54383	5.799999	54.56382
5.599998474	4.573852893	5.700000763	8.696993	5.799999	17.32619	5.900002	30.52581	5.900002	56.48907	5.599998	4.935659	5.699997	9.137119	5.799999	18.03417	5.899998	29.62804	5.899998	54.73429
5.699996948	4.597968323	5.799999237	8.743896	5.899998	17.41199	6	30.64922	6	56.69919	5.699997	4.946497	5.799999	9.158488	5.899998	18.07659	6	29.71211	6	54.90451
5.799999237	4.599716744	5.900001526	8.787175	6	17.50288	6.100002	30.76339	6.100002	56.89769	5.799999	4.957324	5.899998	9.179826	6	18.11894	6.099998	29.79602	6.099998	55.07444
5.899997711	4.631104196	6	8.82931	6.099998	17.58813	6.200001	30.87665	6.200001	57.09765	5.899998	4.968138	6	9.201137	6.099998	18.16123	6.199997	29.8798	6.199997	55.24411
6	4.636102282	6.100002289	8.858491	6.200001	17.66542	6.300003	30.98535	6.300003	57.3091	6	4.978941	6.099998	9.222417	6.199997	18.20345	6.299999	29.96343	6.299999	55.41351
6.099998474	4.633950489	6.200000763	8.90408	6.299999	17.74703	6.400002	31.0705	6.400002	57.49902	6.099998	4.989732	6.199997	9.243668	6.299999	18.24561	6.399998	30.04691	6.399998	55.58263
6.199996948	4.645018179	6.299999237	8.941928	6.399998	17.8077	6.5	31.16592	6.5	57.6773	6.199997	5.000511	6.299999	9.26489	6.399998	18.28771	6.5	30.13024	6.5	55.75148
6.299999237	4.680706302	6.400001526	8.968886	6.5	17.88524	6.600002	31.2652	6.600002	57.86447	6.299999	5.011279	6.399998	9.286083	6.5	18.32974	6.599998	30.21343	6.599998	55.92004
6.399997711	4.696971239	6.5	8.989145	6.599998	17.95832	6.700001	31.35618	6.700001	58.04477	6.399998	5.022034	6.5	9.307247	6.599998	18.3717	6.699997	30.29648	6.699997	56.08834
6.5	4.711106964	6.600002289	9.008025	6.700001	18.03071	6.800003	31.43824	6.800003	58.21537	6.5	5.032779	6.599998	9.328381	6.699997	18.41361	6.799999	30.37938	6.799999	56.25636
6.599998474	4.724724501	6.700000763	9.054515	6.799999	18.11085	6.900002	31.5218	6.900002	58.39774	6.599998	5.04351	6.699997	9.349486	6.799999	18.45545	6.899998	30.46213	6.899998	56.42409
6.699996948	4.743389054	6.799999237	9.091408	6.899998	18.18938	7	31.59976	7	58.5672	6.699997	5.05423	6.799999	9.370562	6.899998	18.49722	7	30.54473	7	56.59155
6.799999237	4.762619006	6.900001526	9.121715	7	18.25281	7.100002	31.71128	7.100002	58.74988	6.799999	5.064939	6.899998	9.391609	7	18.53893	7.099998	30.62719	7.099998	56.75873

6.899997711	4.774389223	7	9.144874	7.099998	18.34888	7.200001	31.81168	7.200001	58.91797	6.899998	5.075636	7	9.412627	7.099998	18.58058	7.199997	30.7095	7.199997	56.92562
7	4.771839076	7.100002289	9.200112	7.200001	18.41564	7.300003	31.90913	7.300003	59.07894	7	5.086321	7.099998	9.433616	7.199997	18.62216	7.299999	30.79166	7.299999	57.09224
7.099998474	4.798841414	7.200000763	9.237009	7.299999	18.48166	7.400002	31.99531	7.400002	59.23419	7.099998	5.096994	7.199997	9.454575	7.299999	18.66368	7.399998	30.87368	7.399998	57.25857
7.199996948	4.807992829	7.299999237	9.268458	7.399998	18.55606	7.5	32.08946	7.5	59.40505	7.199997	5.107656	7.299999	9.475506	7.399998	18.70513	7.5	30.95555	7.5	57.42462
7.299999237	4.815520326	7.400001526	9.298046	7.5	18.62502	7.600002	32.17337	7.600002	59.58473	7.299999	5.118306	7.399998	9.496407	7.5	18.74653	7.599998	31.03727	7.599998	57.59038
7.399997711	4.826685435	7.5	9.341687	7.599998	18.68597	7.700001	32.25514	7.700001	59.74165	7.399998	5.128944	7.5	9.517281	7.599998	18.78785	7.699997	31.11884	7.699997	57.75585
7.5	4.8485099	7.600002289	9.377358	7.700001	18.74178	7.800003	32.33448	7.800003	59.91515	7.5	5.139571	7.599998	9.538124	7.699997	18.82911	7.799999	31.20026	7.799999	57.92104
7.599998474	4.862442328	7.700000763	9.395657	7.799999	18.80571	7.900002	32.42958	7.900002	60.08441	7.599998	5.150185	7.699997	9.558938	7.799999	18.87031	7.899998	31.28154	7.899998	58.08594
7.699996948	4.873129667	7.799999237	9.427879	7.899998	18.866	8	32.50563	8	60.23564	7.699997	5.160788	7.799999	9.579725	7.899998	18.91145	8	31.36267	8	58.25055
7.799999237	4.887074852	7.900001526	9.45659	8	18.92196	8.100002	32.58251	8.100002	60.37664	7.799999	5.17138	7.899998	9.600481	8	18.95252	8.099998	31.44365	8.099998	58.41487
7.899997711	4.911214595	8	9.482147	8.099998	18.97605	8.200001	32.67473	8.200001	60.54714	7.899998	5.18196	8	9.62121	8.099998	18.99352	8.199997	31.52448	8.199997	58.57889
8	4.925896015	8.100002289	9.496151	8.200001	19.03625	8.300003	32.76125	8.300003	60.69578	8	5.192528	8.099998	9.641909	8.199997	19.03446	8.299999	31.60516	8.299999	58.74263
8.099998474	4.938501191	8.200000763	9.531669	8.299999	19.09452	8.400002	32.84533	8.400002	60.84556	8.099998	5.203085	8.199997	9.662579	8.299999	19.07534	8.399998	31.68569	8.399998	58.90607
8.199996948	4.961570814	8.299999237	9.558623	8.399998	19.16087	8.5	32.93212	8.5	61.00031	8.199997	5.213629	8.299999	9.683221	8.399998	19.11616	8.5	31.76608	8.5	59.06922
8.299999237	4.97008289	8.400001526	9.57482	8.5	19.21921	8.600002	33.01871	8.600002	61.15148	8.299999	5.224163	8.399998	9.703834	8.5	19.15691	8.599998	31.84631	8.599998	59.23207
8.399997711	4.98171379	8.5	9.59385	8.599998	19.28254	8.700001	33.10156	8.700001	61.31003	8.399998	5.234685	8.5	9.724418	8.599998	19.1976	8.699997	31.9264	8.699997	59.39462
8.5	4.989526779	8.600002289	9.613645	8.700001	19.35007	8.800003	33.18275	8.800003	61.45781	8.5	5.245195	8.599998	9.744973	8.699997	19.23822	8.799999	32.00633	8.799999	59.55689
8.599998474	4.999577156	8.700000763	9.640828	8.799999	19.40396	8.900002	33.26128	8.900002	61.59982	8.599998	5.255693	8.699997	9.765499	8.799999	19.27878	8.899998	32.08612	8.899998	59.71884
8.699996948	5.01443074	8.799999237	9.666901	8.899998	19.45541	9	33.32809	9	61.74039	8.699997	5.26618	8.799999	9.785998	8.899998	19.31927	9	32.16576	9	59.88051
8.799999237	5.028581617	8.900001526	9.688656	9	19.5103	9.100002	33.40536	9.100002	61.86775	8.799999	5.276656	8.899998	9.806467	9	19.35971	9.099998	32.24524	9.099998	60.04187
8.899997711	5.040044998	9	9.713016	9.099998	19.5575	9.200001	33.47966	9.200001	62.01375	8.899998	5.28712	9	9.826908	9.099998	19.40007	9.199997	32.32457	9.199997	60.20292
9	5.06016396	9.100002289	9.739528	9.200001	19.60988	9.300003	33.5544	9.300003	62.15648	9	5.297572	9.099998	9.847319	9.199997	19.44038	9.299999	32.40376	9.299999	60.36368
9.099998474	5.076572136	9.200000763	9.764789	9.299999	19.65926	9.400002	33.62339	9.400002	62.29181	9.099998	5.308013	9.199997	9.867703	9.299999	19.48062	9.399998	32.4828	9.399998	60.52413
9.199996948	5.087812936	9.299999237	9.789158	9.399998	19.71339	9.5	33.69553	9.5	62.43035	9.199997	5.318442	9.299999	9.888058	9.399998	19.52079	9.5	32.56168	9.5	60.68428
9.299999237	5.097698278	9.400001526	9.81623	9.5	19.75669	9.600002	33.76459	9.600002	62.57496	9.299999	5.32886	9.399998	9.908384	9.5	19.56091	9.599998	32.64041	9.599998	60.84412
9.399997711	5.115969958	9.5	9.846072	9.599998	19.8105	9.700001	33.84084	9.700001	62.71661	9.399998	5.339267	9.5	9.928682	9.599998	19.60096	9.699997	32.71899	9.699997	61.00365
9.5	5.127116112	9.600002289	9.86756	9.700001	19.86424	9.800003	33.91141	9.800003	62.84676	9.5	5.349662	9.599998	9.948951	9.699997	19.64094	9.799999	32.79743	9.799999	61.16289
9.599998474	5.13862141	9.700000763	9.893227	9.799999	19.91298	9.900002	33.98513	9.900002	62.97704	9.599998	5.360045	9.699997	9.969191	9.799999	19.68086	9.899998	32.87571	9.899998	61.3218
9.699996948	5.153595511	9.799999237	9.914925	9.899998	19.96824	10	34.05967	10	63.11153	9.699997	5.370417	9.799999	9.989404	9.899998	19.72072	10	32.95384	10	61.48042
9.799999237	5.164244427	9.900001526	9.940536	10	20.02086	10.1	34.12961	10.1	63.24483	9.799999	5.380777	9.899998	10.00959	10	19.76052	10.1	33.03181	10.1	61.63871
9.899997711	5.174489303	10	9.96559	10.1	20.06798	10.2	34.19383	10.2	63.37452	9.899998	5.391126	10	10.02974	10.1	19.80025	10.2	33.10964	10.2	61.7967
10	5.186012635	10.10000229	9.984183	10.2	20.12331	10.3	34.27063	10.3	63.51547	10	5.401464	10.1	10.04987	10.2	19.83991	10.3	33.18731	10.3	61.95437
10.09999847	5.197818783	10.20000076	10.01235	10.3	20.18238	10.4	34.34501	10.4	63.65849	10.1	5.41179	10.2	10.06997	10.3	19.87952	10.4	33.26483	10.4	62.11173
10.19999695	5.210199294	10.29999924	10.03987	10.4	20.23215	10.5	34.41123	10.5	63.78783	10.2	5.422104	10.3	10.09004	10.4	19.91906	10.5	33.34221	10.5	62.26878
10.29999924	5.224653357	10.40000153	10.06083	10.5	20.27175	10.6	34.48289	10.6	63.91824	10.3	5.432408	10.4	10.11008	10.5	19.95853	10.6	33.41942	10.6	62.42551
10.39999771	5.23257679	10.5	10.08241	10.6	20.31546	10.7	34.55103	10.7	64.04736	10.4	5.4427	10.5	10.13009	10.6	19.99795	10.7	33.49649	10.7	62.58192
10.5	5.243157158	10.60000229	10.10436	10.7	20.36462	10.8	34.62727	10.8	64.18645	10.5	5.45298	10.6	10.15008	10.7	20.03729	10.8	33.5734	10.8	62.73802
10.59999847	5.253074095	10.70000076	10.12426	10.8	20.41979	10.9	34.69975	10.9	64.31931	10.6	5.463249	10.7	10.17004	10.8	20.07658	10.9	33.65016	10.9	62.89379
10.69999695	5.265667715	10.79999924	10.15262	10.9	20.46977	11	34.77174	11	64.4518	10.7	5.473507	10.8	10.18997	10.9	20.1158	11	33.72677	11	63.04925
10.79999924	5.287139851	10.90000153	10.175	11	20.52623	11.1	34.84606	11.1	64.57547	10.8	5.483753	10.9	10.20987	11	20.15496	11.1	33.80322	11.1	63.20438
10.89999771	5.302615935	11	10.2025	11.1	20.58332	11.2	34.91245	11.2	64.70151	10.9	5.493988	11	10.22974	11.1	20.19406	11.2	33.87952	11.2	63.35919
11	5.317128595	11.10000229	10.22971	11.2	20.63028	11.3	34.9829	11.3	64.83004	11	5.504212	11.1	10.24958	11.2	20.23309	11.3	33.95567	11.3	63.51368
11.09999847	5.33585925	11.20000076	10.25349	11.3	20.67896	11.4	35.05589	11.4	64.9576	11.1	5.514424	11.2	10.2694	11.3	20.27206	11.4	34.03167	11.4	63.66785
11.19999695	5.349004646	11.29999924	10.27871	11.4	20.73378	11.5	35.12589	11.5	65.08251	11.2	5.524625	11.3	10.28919	11.4	20.31096	11.5	34.10751	11.5	63.82169
11.29999924	5.366832971	11.40000153	10.30448	11.5	20.78483	11.6	35.19458	11.6	65.20591	11.3	5.534815	11.4	10.30895	11.5	20.34981	11.6	34.1832	11.6	63.9752
11.39999771	5.3806982	11.5	10.32581	11.6	20.83386	11.7	35.26128	11.7	65.32562	11.4	5.544993	11.5	10.32868	11.6	20.38859	11.7	34.25873	11.7	64.12838
11.5	5.393878505	11.60000229	10.35089	11.7	20.88587	11.8	35.33051	11.8	65.45094	11.5	5.555161	11.6	10.34838	11.7	20.4273	11.8	34.33412	11.8	64.28124
11.59999847	5.404586976	11.70000076	10.37419	11.8	20.93658	11.9	35.40212	11.9	65.58412	11.6	5.565316	11.7	10.36806	11.8	20.46595	11.9	34.40934	11.9	64.43377
11.69999695	5.415491401	11.79999924	10.39602	11.9	20.98981	12	35.46814	12	65.71363	11.7	5.575461	11.8	10.38771	11.9	20.50454	12	34.48442	12	64.58597
11.79999924	5.428390823	11.90000153	10.42262	12	21.0422	12.1	35.53811	12.1	65.83233	11.8	5.585594	11.9	10.40733	12	20.54307	12.1	34.55933	12.1	64.73783
11.89999771	5.446819396	12	10.44867	12.1	21.08901	12.2	35.60495	12.2	65.95208	11.9	5.595716	12	10.42692	12.1	20.58153	12.2	34.6341	12.2	64.88936
12	5.461419108	12.10000229	10.47368	12.2	21.13489	12.3	35.67279	12.3	66.07564	12	5.605827	12.1	10.44648	12.2	20.61993	12.3	34.70871	12.3	65.04057
12.09999847	5.479408961	12.20000076	10.49561	12.3	21.18175	12.4	35.73143	12.4	66.1966	12.1	5.615927	12.2	10.46602	12.3	20.65827	12.4	34.78317	12.4	65.19143
12.19999695	5.493063666	12.29999924	10.52181	12.4	21.22449	12.5	35.79792	12.5	66.31931	12.2	5.626015	12.3	10.48553	12.4	20.69654	12.5	34.85747	12.5	65.34196
12.29999924	5.508439498	12.40000153	10.54256	12.5	21.2674	12.6	35.86346	12.6	66.45097	12.3	5.636092	12.4	10.50501	12.5	20.73475	12.6	34.93162	12.6	65.49215
12.39999771	5.520273779	12.5	10.56569	12.6	21.31227	12.7	35.93122	12.7	66.57715	12.4	5.646158	12.5	10.52446	12.6	20.7729	12.7	35.00561	12.7	65.642
12.5	5.526244696	12.60000229	10.58738	12.7	21.35906	12.8	35.99281	12.8	66.69863	12.5	5.656213	12.6	10.54388	12.7	20.81098	12.8	35.07945	12.8	65.79152
12.59999847	5.535602735	12.70000076	10.61125	12.8	21.40744	12.9	36.05577	12.9	66.82313	12.6	5.666256	12.7	10.56328	12.8	20.849	12.9	35.15313	12.9	65.94069
12.69999695	5.552733739	12.79999924	10.63107	12.9	21.45269	13	36.12082	13	66.94445	12.7	5.676289	12.8	10.58265	12.9	20.88696	13	35.22665	13	66.08953
12.79999924	5.560399266	12.90000153	10.6525	13	21.49246	13.1	36.18825	13.1	67.06753	12.8	5.68631	12.9	10.60199	13	20.92486	13.1	35.30003	13.1	66.23802
12.89999771	5.574614268	13	10.67634	13.1	21.53665	13.2	36.25052	13.2	67.19013	12.9	5.69632	13	10.6213	13.1	20.96269	13.2	35.37324	13.2	66.38617
13	5.58524205	13.10000229	10.69879	13.2	21.58324	13.3	36.3158	13.3	67.30446	13	5.706319	13.1	10.64059	13.2	21.00046	13.3	35.4463	13.3	66.53397
13.09999847	5.601205204	13.20000076	10.71859	13.3	21.62974	13.4	36.38194	13.4	67.41628	13.1	5.716307	13.2	10.65985	13.3	21.03816	13.4	35.51921	13.4	66.68143
13.19999695	5.619927673	13.29999924	10.73561	13.4	21.67541	13.5	36.44464	13.5	67.53379	13.2	5.726283	13.3	10.67908	13.4	21.07581	13.5	35.59196	13.5	66.82855
13.29999924	5.627132884	13.40000153	10.75847	13.5	21.72224	13.6	36.51494	13.6	67.65191	13.3	5.736249	13.4	10.69828	13.5	21.11339	13.6	35.66455	13.6	66.97531
13.39999771	5.637864972	13.5	10.78593	13.6	21.7739	13.7	36.58041	13.7	67.76841	13.4	5.746203	13.5	10.71746	13.6	21.1509	13.7	35.73699	13.7	67.12172
13.5	5.653588454	13.60000229	10.81191	13.7	21.81445	13.8	36.65208	13.8	67.8956	13.5	5.756147	13.6	10.7366	13.7	21.18836	13.8	35.80927	13.8	67.26779
13.59999847	5.668690392	13.70000076	10.83806	13.8	21.85952	13.9	36.71701	13.9	68.0191	13.6	5.766079	13.7	10.75572	13.8	21.22575	13.9	35.8814	13.9	67.41351
13.69999695	5.67952488	13.79999924	10.86218	13.9	21.90649	14	36.77567	14	68.1386	13.7	5.776	13.8	10.77482	13.9	21.26308	14	35.95337	14	67.55888
13.79999924	5.694426528	13.90000153	10.89136	14	21.95108	14.1	36.83552	14.1	68.25592	13.8	5.78591	13.9	10.79388	14	21.30035	14.1	36.02518	14.1	67.70389
13.89999771	5.707250043	14	10.91155	14.1	21.99469	14.2	36.89424	14.2	68.37233	13.9	5.795809	14	10.81292	14.1	21.33755	14.2	36.09684	14.2	67.84855
14	5.719064946	14.10000229	10.93401	14.2	22.04021	14.3	36.95294	14.3	68.48735	14	5.805697	14.1	10.83193	14.2	21.37469	14.3	36.16834	14.3	67.99285
14.09999847	5.729664694	14.20000076	10.95835	14.3	22.08313	14.4	37.01554	14.4	68.59859	14.1	5.815574	14.2	10.85091	14.3	21.41177	14.4	36.23968	14.4	68.1368
14.19999695	5.739566781	14.29999924	10.98172	14.4	22.12647	14.5	37.07936	14.5	68.70223	14.2	5.825439	14.3	10.86987	14.4	21.44879	14.5	36.31087	14.5	68.28039
14.29999924	5.751298775	14.40000153	11.00347	14.5	22.16855	14.6	37.14667	14.6	68.8157	14.3	5.835295	14.4	10.88879	14.5	21.48574	14.6	36.3819	14.6	68.42363
14.39999771	5.764450299	14.5	11.02533	14.6	22.21227	14.7	37.21081	14.7	68.922	14.4	5.845138	14.5	10.9077	14.6	21.52263	14.7	36.45277	14.7	68.5665
14.5	5.771444129	14.60000229	11.05018	14.7	22.2561	14.8	37.27426	14.8	69.02531	14.5	5.854971	14.6	10.92657	14.7	21.55946	14.8	36.52349	14.8	68.70902
14.59999847	5.786722278	14.70000076	11.07198	14.8	22.29824	14.9	37.34042	14.9	69.13528	14.6	5.864793	14.7	10.94541	14.8	21.59623	14.9	36.59405	14.9	68.85117
14.69999695	5.797193261	14.79999924	11.09174	14.9	22.33912	15	37.40179	15	69.24141	14.7	5.874604	14.8	10.96423	14.9	21.63293	15	36.66446	15	68.99297
14.79999924	5.806872586	14.90000153	11.119	15	22.37762	15.1	37.46024	15.1	69.35259	14.8	5.884404	14.9	10.98302	15	21.66957	15.1	36.7347	15.1	69.13439
14.89999771	5.82128963	15	11.14439	15.1	22.42057	15.2	37.51758	15.2	69.4652	14.9	5.894193	15	11.00179	15.1	21.70615	15.2	36.80479	15.2	69.27546
15	5.833162766	15.10000229	11.16733	15.2	22.45779	15.3	37.57854	15.3	69.57796	15	5.903971	15.1	11.02053	15.2	21.74267	15.3	36.87472	15.3	69.41616
15.09999847	5.847033593	15.20000076	11.1868	15.3	22.49609	15.4	37.63834	15.4	69.68949	15.1	5.913738	15.2	11.03923	15.3	21.77912	15.4	36.94449	15.4	69.55649
15.19999695	5.856284679	15.29999924	11.20781	15.4	22.53541	15.5	37.69854	15.5	69.80475	15.2	5.923494	15.3	11.05792	15.4	21.81551	15.5	37.01411	15.5	69.69646
15.29999924	5.866707809	15.40000153	11.2255	15.5	22.57775	15.6	37.75887	15.6	69.9168	15.3	5.933239	15.4	11.07657	15.5	21.85184	15.6	37.08357	15.6	69.83606
15.39999771	5.876646343	15.5	11.24623	15.6	22.61917	15.7	37.82264	15.7	70.03188	15.4	5.942973	15.5	11.0952	15.6	21.88811	15.7	37.15287	15.7	69.97529
15.5	5.886453906	15.60000229	11.26879	15.7	22.66061	15.8	37.88499	15.8	70.14239	15.5	5.952697	15.6	11.1138	15.7	21.92431	15.8	37.22201	15.8	70.11415
15.59999847	5.900222549	15.70000076	11.29017	15.8	22.69682	15.9	37.94478	15.9	70.24788	15.6	5.962409	15.7	11.13238	15.8	21.96046	15.9	37.291	15.9	70.25264
15.69999695	5.914143975	15.79999924	11.31027	15.9	22.73443	16	38.00269	16	70.34747	15.7	5.972111	15.8	11.15092	15.9	21.99654	16	37.35982	16	70.39076
15.79999924	5.924925496	15.90000153	11.32897	16	22.77041	16.1	38.06451	16.1	70.45671	15.8	5.981802	15.9	11.16944	16	22.03255	16.1	37.42849	16.1	70.5285
15.89999771	5.937109334	16	11.34562	16.1	22.81632	16.2	38.12013	16.2	70.55981	15.9	5.991482	16	11.18794	16.1	22.06851	16.2	37.497	16.2	70.66586
16	5.945401089	16.10000229	11.37113	16.2	22.85591	16.3	38.18004	16.3	70.66602	16	6.001151	16.1	11.2064	16.2	22.1044	16.3	37.56536	16.3	70.80286
16.09999847	5.961835251	16.20000076	11.39522	16.3	22.89996	16.4	38.24391	16.4	70.77579	16.1	6.010809	16.2	11.22484	16.3	22.14024	16.4	37.63355	16.4	70.93948
16.19999695	5.972031453	16.29999924	11.41533	16.4	22.94464	16.5	38.30603	16.5	70.8864	16.2	6.020456	16.3	11.24325	16.4	22.17601	16.5	37.70159	16.5	71.07572
16.29999924	5.986997709	16.40000153	11.44131	16.5	22.98485	16.6	38.36557	16.6	70.98911	16.3	6.030093	16.4	11.26164	16.5	22.21171	16.6	37.76947	16.6	71.21158
16.39999771	6.00328661	16.5	11.46497	16.6	23.02329	16.7	38.42189	16.7	71.09102	16.4	6.039718	16.5	11.28	16.6	22.24736	16.7	37.83719	16.7	71.34706
16.5	6.017199219	16.60000229	11.48638	16.7	23.06212	16.8	38.48029	16.8	71.19647	16.5	6.049333	16.6	11.29833	16.7	22.28294	16.8	37.90475	16.8	71.48217
16.59999847	6.023988594	16.70000076	11.50846	16.8	23.0999	16.9	38.54049	16.9	71.30009	16.6	6.058937	16.7	11.31663	16.8	22.31847	16.9	37.97215	16.9	71.61689
16.69999695	6.031402819	16.79999924	11.53126	16.9	23.13378	17	38.59775	17	71.40482	16.7	6.06853	16.8	11.33491	16.9	22.35393	17	38.0394	17	71.75123
16.79999924	6.043945405	16.90000153	11.54774	17	23.17672	17.1	38.65598	17.1	71.50424	16.8	6.078113	16.9	11.35316	17	22.38933	17.1	38.10648	17.1	71.88519
16.89999771	6.05352406	17	11.56991	17.1	23.21583	17.2	38.70859	17.2	71.60864	16.9	6.087685	17	11.37139	17.1	22.42466	17.2	38.17341	17.2	72.01876
17	6.069920674	17.10000229	11.59102	17.2	23.25182	17.3	38.76223	17.3	71.71164	17	6.097246	17.1	11.38958	17.2	22.45994	17.3	38.24018	17.3	72.15195
17.09999847	6.086826071	17.20000076	11.61284	17.3	23.28962	17.4	38.82055	17.4	71.81661	17.1	6.106796	17.2	11.40775	17.3	22.49515	17.4	38.30679	17.4	72.28475
17.19999695	6.099091871	17.29999924	11.6313	17.4	23.33218	17.5	38.87615	17.5	71.91687	17.2	6.116335	17.3	11.4259	17.4	22.5303	17.5	38.37324	17.5	72.41716
17.29999924	6.109768265	17.40000153	11.65028	17.5	23.36783	17.6	38.93447	17.6	72.0158	17.3	6.125864	17.4	11.44402	17.5	22.56539	17.6	38.43953	17.6	72.54919
17.39999771	6.121766248	17.5	11.6675	17.6	23.40678	17.7	38.99786	17.7	72.11482	17.4	6.135382	17.5	11.46211	17.6	22.60042	17.7	38.50566	17.7	72.68082
17.5	6.133329854	17.60000229	11.68429	17.7	23.44515	17.8	39.05891	17.8	72.21624	17.5	6.14489	17.6	11.48017	17.7	22.63539	17.8	38.57164	17.8	72.81207
17.59999847	6.141204204	17.70000076	11.70117	17.8	23.47701	17.9	39.11221	17.9	72.31466	17.6	6.154386	17.7	11.49821	17.8	22.67029	17.9	38.63745	17.9	72.94293
17.69999695	6.151278769	17.79999924	11.71287	17.9	23.50874	18	39.16839	18	72.41476	17.7	6.163872	17.8	11.51622	17.9	22.70514	18	38.70311	18	73.0734
17.79999924	6.162950689	17.90000153	11.7266	18	23.54892	18.1	39.22391	18.1	72.51281	17.8	6.173348	17.9	11.5342	18	22.73992	18.1	38.76861	18.1	73.20347
17.89999771	6.170594038	18	11.74718	18.1	23.58692	18.2	39.27997	18.2	72.62142	17.9	6.182812	18	11.55216	18.1	22.77464	18.2	38.83395	18.2	73.33315
18	6.182781258	18.10000229	11.76405	18.2	23.62441	18.3	39.33764	18.3	72.72719	18	6.192266	18.1	11.57009	18.2	22.8093	18.3	38.89913	18.3	73.46243
18.09999847	6.196712456	18.20000076	11.7849	18.3	23.66431	18.4	39.38838	18.4	72.82823	18.1	6.201709	18.2	11.588	18.3	22.8439	18.4	38.96414	18.4	73.59132
18.19999695	6.208854298	18.29999924	11.80692	18.4	23.69958	18.5	39.44507	18.5	72.92658	18.2	6.211142	18.3	11.60588	18.4	22.87844	18.5	39.02901	18.5	73.71982
18.29999924	6.224750988	18.40000153	11.82272	18.5	23.73052	18.6	39.50749	18.6	73.03258	18.3	6.220564	18.4	11.62373	18.5	22.91291	18.6	39.09371	18.6	73.84791

18.39999771	6.234136022	18.5	11.841	18.6	23.76851	18.7	39.57271	18.7	73.13651	18.4	6.229975	18.5	11.64156	18.6	22.94733	18.7	39.15825	18.7	73.97561
18.5	6.245921093	18.60000229	11.86511	18.7	23.80393	18.8	39.62537	18.8	73.23866	18.5	6.239376	18.6	11.65935	18.7	22.98168	18.8	39.22263	18.8	74.10291
18.59999847	6.263878183	18.70000076	11.89158	18.8	23.83877	18.9	39.68275	18.9	73.34417	18.6	6.248766	18.7	11.67713	18.8	23.01597	18.9	39.28685	18.9	74.2298
18.69999695	6.281109319	18.79999924	11.91148	18.9	23.87251	19	39.73963	19	73.44448	18.7	6.258146	18.8	11.69487	18.9	23.0502	19	39.35092	19	74.3563
18.79999924	6.289819417	18.90000153	11.92929	19	23.91069	19.1	39.79227	19.1	73.54279	18.8	6.267515	18.9	11.71259	19	23.08437	19.1	39.41482	19.1	74.4824
18.89999771	6.304359211	19	11.94932	19.1	23.94779	19.2	39.84624	19.2	73.64427	18.9	6.276873	19	11.73029	19.1	23.11848	19.2	39.47856	19.2	74.60809
19	6.313272417	19.10000229	11.9655	19.2	23.98547	19.3	39.89637	19.3	73.7495	19	6.286221	19.1	11.74796	19.2	23.15253	19.3	39.54215	19.3	74.73338
19.09999847	6.322917848	19.20000076	11.9859	19.3	24.02129	19.4	39.94758	19.4	73.85746	19.1	6.295558	19.2	11.7656	19.3	23.18651	19.4	39.60558	19.4	74.85826
19.19999695	6.33645041	19.29999924	12.00456	19.4	24.05541	19.5	40.0048	19.5	73.95478	19.2	6.304885	19.3	11.78321	19.4	23.22044	19.5	39.66884	19.5	74.98275
19.29999924	6.346732287	19.40000153	12.02367	19.5	24.09507	19.6	40.05934	19.6	74.05035	19.3	6.314201	19.4	11.8008	19.5	23.2543	19.6	39.73195	19.6	75.10682
19.39999771	6.359815158	19.5	12.04326	19.6	24.13287	19.7	40.11428	19.7	74.14744	19.4	6.323507	19.5	11.81837	19.6	23.28811	19.7	39.79489	19.7	75.23049
19.5	6.373385117	19.60000229	12.06073	19.7	24.16801	19.8	40.16813	19.8	74.24776	19.5	6.332802	19.6	11.8359	19.7	23.32185	19.8	39.85768	19.8	75.35375
19.59999847	6.383856367	19.70000076	12.08108	19.8	24.20047	19.9	40.22354	19.9	74.34283	19.6	6.342087	19.7	11.85341	19.8	23.35553	19.9	39.92031	19.9	75.4766
19.69999695	6.400443547	19.79999924	12.10056	19.9	24.23083	20	40.27288	20	74.43603	19.7	6.351361	19.8	11.8709	19.9	23.38915	20	39.98278	20	75.59904
19.79999924	6.413793265	19.90000153	12.11594	20	24.26048	20.1	40.32796	20.1	74.53695	19.8	6.360625	19.9	11.88836	20	23.42271	20.1	40.04509	20.1	75.72107
19.89999771	6.424268947	20	12.13035	20.1	24.29119	20.2	40.38207	20.2	74.63542	19.9	6.369878	20	11.90579	20.1	23.45621	20.2	40.10723	20.2	75.84269
20	6.436412209	20.10000229	12.15302	20.2	24.32226	20.3	40.44169	20.3	74.73576	20	6.37912	20.1	11.9232	20.2	23.48965	20.3	40.16922	20.3	75.9639
20.09999847	6.449325191	20.20000076	12.17334	20.3	24.35961	20.4	40.49359	20.4	74.83066	20.1	6.388352	20.2	11.94058	20.3	23.52303	20.4	40.23105	20.4	76.0847
20.19999695	6.463542675	20.29999924	12.19207	20.4	24.38791	20.5	40.54721	20.5	74.92561	20.2	6.397574	20.3	11.95793	20.4	23.55635	20.5	40.29272	20.5	76.20508
20.29999924	6.480249183	20.40000153	12.20492	20.5	24.41987	20.6	40.59824	20.6	75.01932	20.3	6.406786	20.4	11.97526	20.5	23.58961	20.6	40.35423	20.6	76.32505
20.39999771	6.492265733	20.5	12.2222	20.6	24.45091	20.7	40.65092	20.7	75.10859	20.4	6.415986	20.5	11.99257	20.6	23.6228	20.7	40.41558	20.7	76.4446
20.5	6.503847782	20.60000229	12.23885	20.7	24.4822	20.8	40.70312	20.8	75.20607	20.5	6.425177	20.6	12.00984	20.7	23.65594	20.8	40.47677	20.8	76.56374
20.59999847	6.515725721	20.70000076	12.25596	20.8	24.51762	20.9	40.76106	20.9	75.30402	20.6	6.434357	20.7	12.02709	20.8	23.68902	20.9	40.5378	20.9	76.68246
20.69999695	6.526603611	20.79999924	12.27569	20.9	24.55346	21	40.81772	21	75.39867	20.7	6.443526	20.8	12.04432	20.9	23.72203	21	40.59868	21	76.80077
20.79999924	6.543414315	20.90000153	12.29753	21	24.59214	21.1	40.87007	21.1	75.49248	20.8	6.452686	20.9	12.06152	21	23.75499	21.1	40.65939	21.1	76.91865
20.89999771	6.560363996	21	12.32009	21.1	24.62738	21.2	40.91984	21.2	75.58655	20.9	6.461835	21	12.07869	21.1	23.78788	21.2	40.71994	21.2	77.03612
21	6.577561987	21.10000229	12.34128	21.2	24.65646	21.3	40.96804	21.3	75.68256	21	6.470973	21.1	12.09584	21.2	23.82072	21.3	40.78033	21.3	77.15317
21.09999847	6.591718282	21.20000076	12.35746	21.3	24.69079	21.4	41.01655	21.4	75.77739	21.1	6.480101	21.2	12.11296	21.3	23.85349	21.4	40.84056	21.4	77.2698
21.19999695	6.605304683	21.29999924	12.37919	21.4	24.71832	21.5	41.06511	21.5	75.86966	21.2	6.489219	21.3	12.13006	21.4	23.88621	21.5	40.90064	21.5	77.38601
21.29999924	6.623098422	21.40000153	12.40075	21.5	24.74593	21.6	41.11414	21.6	75.96047	21.3	6.498327	21.4	12.14713	21.5	23.91886	21.6	40.96055	21.6	77.50179
21.39999771	6.635517081	21.5	12.42169	21.6	24.77118	21.7	41.16294	21.7	76.04964	21.4	6.507423	21.5	12.16418	21.6	23.95146	21.7	41.0203	21.7	77.61716
21.5	6.650981893	21.60000229	12.43671	21.7	24.7988	21.8	41.21252	21.8	76.13521	21.5	6.51651	21.6	12.1812	21.7	23.98399	21.8	41.0799	21.8	77.7321
21.59999847	6.665514456	21.70000076	12.45322	21.8	24.82419	21.9	41.27003	21.9	76.22565	21.6	6.525587	21.7	12.19819	21.8	24.01646	21.9	41.13933	21.9	77.84662
21.69999695	6.672061395	21.79999924	12.47052	21.9	24.85715	22	41.32092	22	76.32108	21.7	6.534653	21.8	12.21516	21.9	24.04888	22	41.19861	22	77.96071
21.79999924	6.679980029	21.90000153	12.49538	22	24.88632	22.1	41.36671	22.1	76.40993	21.8	6.543709	21.9	12.2321	22	24.08123	22.1	41.25772	22.1	78.07438
21.89999771	6.691214595	22	12.51775	22.1	24.90857	22.2	41.41937	22.2	76.49917	21.9	6.552754	22	12.24902	22.1	24.11353	22.2	41.31668	22.2	78.18762
22	6.70621391	22.10000229	12.5294	22.2	24.93085	22.3	41.47769	22.3	76.59864	22	6.561789	22.1	12.26591	22.2	24.14576	22.3	41.37548	22.3	78.30044
22.09999847	6.715010364	22.20000076	12.54645	22.3	24.96545	22.4	41.5282	22.4	76.69319	22.1	6.570814	22.2	12.28278	22.3	24.17794	22.4	41.43411	22.4	78.41283
22.19999695	6.723557216	22.29999924	12.56999	22.4	24.99893	22.5	41.57126	22.5	76.78913	22.2	6.579829	22.3	12.29962	22.4	24.21005	22.5	41.49259	22.5	78.5248
22.29999924	6.738165889	22.40000153	12.58985	22.5	25.02551	22.6	41.61915	22.6	76.88487	22.3	6.588834	22.4	12.31644	22.5	24.24211	22.6	41.55091	22.6	78.63633
22.39999771	6.749593217	22.5	12.60668	22.6	25.05726	22.7	41.66762	22.7	76.97856	22.4	6.597828	22.5	12.33323	22.6	24.2741	22.7	41.60907	22.7	78.74744
22.5	6.761550731	22.60000229	12.62719	22.7	25.08486	22.8	41.71695	22.8	77.06919	22.5	6.606812	22.6	12.34999	22.7	24.30604	22.8	41.66707	22.8	78.85812
22.59999847	6.773552275	22.70000076	12.64788	22.8	25.115	22.9	41.76121	22.9	77.15618	22.6	6.615785	22.7	12.36673	22.8	24.33791	22.9	41.72491	22.9	78.96836
22.69999695	6.785038825	22.79999924	12.66425	22.9	25.14072	23	41.80699	23	77.24363	22.7	6.624749	22.8	12.38345	22.9	24.36973	23	41.78259	23	79.07818
22.79999924	6.795957417	22.90000153	12.6785	23	25.16986	23.1	41.85904	23.1	77.33311	22.8	6.633702	22.9	12.40014	23	24.40149	23.1	41.84011	23.1	79.18757
22.89999771	6.797790425	23	12.69636	23.1	25.19509	23.2	41.90729	23.2	77.42291	22.9	6.642645	23	12.4168	23.1	24.43319	23.2	41.89747	23.2	79.29652
23	6.807268218	23.10000229	12.71349	23.2	25.22342	23.3	41.9517	23.3	77.51597	23	6.651578	23.1	12.43344	23.2	24.46483	23.3	41.95467	23.3	79.40505
23.09999847	6.814133039	23.20000076	12.73134	23.3	25.25368	23.4	42.00176	23.4	77.61661	23.1	6.660501	23.2	12.45005	23.3	24.49641	23.4	42.01172	23.4	79.51314
23.19999695	6.819330303	23.29999924	12.74557	23.4	25.2807	23.5	42.04853	23.5	77.71574	23.2	6.669413	23.3	12.46664	23.4	24.52792	23.5	42.0686	23.5	79.6208
23.29999924	6.827304975	23.40000153	12.7726	23.5	25.30607	23.6	42.0945	23.6	77.81461	23.3	6.678316	23.4	12.4832	23.5	24.55939	23.6	42.12533	23.6	79.72803
23.39999771	6.842113004	23.5	12.79435	23.6	25.33466	23.7	42.13962	23.7	77.90456	23.4	6.687208	23.5	12.49974	23.6	24.59079	23.7	42.18189	23.7	79.83482
23.5	6.853386511	23.60000229	12.81358	23.7	25.35977	23.8	42.19176	23.8	77.99639	23.5	6.696091	23.6	12.51625	23.7	24.62213	23.8	42.2383	23.8	79.94118
23.59999847	6.863077152	23.70000076	12.83079	23.8	25.38677	23.9	42.24202	23.9	78.08863	23.6	6.704962	23.7	12.53274	23.8	24.65341	23.9	42.29455	23.9	80.0471
23.69999695	6.870200293	23.79999924	12.84652	23.9	25.41663	24	42.28902	24	78.17044	23.7	6.713824	23.8	12.54921	23.9	24.68464	24	42.35064	24	80.15259
23.79999924	6.884650119	23.90000153	12.8673	24	25.44372	24.1	42.33441	24.1	78.25609	23.8	6.722676	23.9	12.56564	24	24.7158	24.1	42.40657	24.1	80.25764
23.89999771	6.896862373	24	12.88351	24.1	25.47125	24.2	42.38353	24.2	78.34788	23.9	6.731518	24	12.58206	24.1	24.74691	24.2	42.46234	24.2	80.36226
24	6.907953248	24.10000229	12.89311	24.2	25.50075	24.3	42.43628	24.3	78.43753	24	6.740349	24.1	12.59844	24.2	24.77796	24.3	42.51796	24.3	80.46644
24.09999847	6.914347408	24.20000076	12.90633	24.3	25.52826	24.4	42.48847	24.4	78.52603	24.1	6.749171	24.2	12.61481	24.3	24.80894	24.4	42.57341	24.4	80.57019
24.19999695	6.923862026	24.29999924	12.92263	24.4	25.55797	24.5	42.53744	24.5	78.6138	24.2	6.757982	24.3	12.63114	24.4	24.83987	24.5	42.62871	24.5	80.6735
24.29999924	6.934719564	24.40000153	12.94166	24.5	25.58375	24.6	42.58556	24.6	78.70727	24.3	6.766784	24.4	12.64746	24.5	24.87074	24.6	42.68385	24.6	80.77637
24.39999771	6.945046807	24.5	12.95763	24.6	25.61532	24.7	42.63378	24.7	78.79625	24.4	6.775575	24.5	12.66374	24.6	24.90156	24.7	42.73883	24.7	80.8788
24.5	6.956381631	24.60000229	12.97529	24.7	25.6407	24.8	42.68196	24.8	78.8827	24.5	6.784356	24.6	12.68001	24.7	24.93231	24.8	42.79365	24.8	80.98079
24.59999847	6.97031639	24.70000076	12.99364	24.8	25.66653	24.9	42.73194	24.9	78.97253	24.6	6.793127	24.7	12.69624	24.8	24.963	24.9	42.84831	24.9	81.08235
24.69999695	6.982805106	24.79999924	13.00932	24.9	25.69438	25	42.77671	25	79.05873	24.7	6.801888	24.8	12.71246	24.9	24.99364	25	42.90282	25	81.18347
24.79999924	6.994985048	24.90000153	13.02562	25	25.72006	25.1	42.82472	25.1	79.14962	24.8	6.81064	24.9	12.72865	25	25.02422	25.1	42.95716	25.1	81.28414
24.89999771	7.011970345	25	13.04194	25.1	25.75051	25.2	42.86991	25.2	79.2286	24.9	6.819381	25	12.74481	25.1	25.05474	25.2	43.01135	25.2	81.38438
25	7.025469726	25.10000229	13.05954	25.2	25.77288	25.3	42.91715	25.3	79.31197	25	6.828112	25.1	12.76095	25.2	25.0852	25.3	43.06538	25.3	81.48418
25.09999847	7.042332546	25.20000076	13.07516	25.3	25.79562	25.4	42.96566	25.4	79.39757	25.1	6.836833	25.2	12.77706	25.3	25.1156	25.4	43.11926	25.4	81.58354
25.19999695	7.049548442	25.29999924	13.09002	25.4	25.82077	25.5	43.01221	25.5	79.48513	25.2	6.845544	25.3	12.79315	25.4	25.14594	25.5	43.17297	25.5	81.68246
25.29999924	7.058925479	25.40000153	13.10801	25.5	25.84752	25.6	43.05981	25.6	79.57137	25.3	6.854245	25.4	12.80922	25.5	25.17623	25.6	43.22653	25.6	81.78094
25.39999771	7.070596946	25.5	13.1269	25.6	25.87029	25.7	43.10856	25.7	79.65624	25.4	6.862936	25.5	12.82526	25.6	25.20645	25.7	43.27993	25.7	81.87897
25.5	7.081183519	25.60000229	13.14167	25.7	25.89669	25.8	43.15881	25.8	79.74801	25.5	6.871618	25.6	12.84127	25.7	25.23662	25.8	43.33317	25.8	81.97657
25.59999847	7.092721781	25.70000076	13.15877	25.8	25.92604	25.9	43.20254	25.9	79.83907	25.6	6.880289	25.7	12.85727	25.8	25.26673	25.9	43.38626	25.9	82.07373
25.69999695	7.104510471	25.79999924	13.17884	25.9	25.95393	26	43.24462	26	79.91442	25.7	6.88895	25.8	12.87323	25.9	25.29679	26	43.43918	26	82.17044
25.79999924	7.114322946	25.90000153	13.19586	26	25.97798	26.1	43.29077	26.1	79.99525	25.8	6.897602	25.9	12.88917	26	25.32678	26.1	43.49195	26.1	82.26671
25.89999771	7.125143908	26	13.21149	26.1	26.00358	26.2	43.33806	26.2	80.07399	25.9	6.906243	26	12.90509	26.1	25.35672	26.2	43.54456	26.2	82.36255
26	7.135666992	26.10000229	13.22611	26.2	26.02929	26.3	43.38435	26.3	80.15311	26	6.914875	26.1	12.92098	26.2	25.3866	26.3	43.59702	26.3	82.45793
26.09999847	7.141918519	26.20000076	13.24051	26.3	26.05902	26.4	43.43184	26.4	80.23843	26.1	6.923497	26.2	12.93685	26.3	25.41642	26.4	43.64932	26.4	82.55289
26.19999695	7.15229425	26.29999924	13.2586	26.4	26.08689	26.5	43.47644	26.5	80.3214	26.2	6.932109	26.3	12.9527	26.4	25.44618	26.5	43.70146	26.5	82.64739
26.29999542	7.164856872	26.39999771	13.28042	26.5	26.1104	26.6	43.52057	26.6	80.40817	26.3	6.940711	26.4	12.96852	26.5	25.47588	26.6	43.75345	26.6	82.74146
26.40000153	7.178304742	26.50000381	13.2997	26.6	26.13415	26.7	43.56624	26.7	80.48752	26.4	6.949303	26.5	12.98431	26.6	25.50553	26.7	43.80527	26.7	82.83508
26.5	7.189021548	26.60000229	13.31656	26.7	26.15741	26.8	43.6145	26.8	80.56852	26.5	6.957885	26.6	13.00008	26.7	25.53512	26.8	43.85694	26.8	82.92826
26.59999847	7.20092336	26.70000076	13.3328	26.8	26.18151	26.9	43.6653	26.9	80.65476	26.6	6.966458	26.7	13.01583	26.8	25.56465	26.9	43.90846	26.9	83.02101
26.69999695	7.21221651	26.79999924	13.34824	26.9	26.20877	27	43.71353	27	80.73466	26.7	6.97502	26.8	13.03155	26.9	25.59413	27	43.95982	27	83.1133
26.79999542	7.219780771	26.89999771	13.36444	27	26.23399	27.1	43.76809	27.1	80.81661	26.8	6.983573	26.9	13.04725	27	25.62354	27.1	44.01102	27.1	83.20516
26.90000153	7.229275929	27.00000381	13.37949	27.1	26.26	27.2	43.81801	27.2	80.90147	26.9	6.992116	27	13.06293	27.1	25.6529	27.2	44.06206	27.2	83.29657
27	7.239086363	27.10000229	13.40271	27.2	26.28746	27.3	43.86564	27.3	80.98717	27	7.000649	27.1	13.07857	27.2	25.6822	27.3	44.11295	27.3	83.38754
27.09999847	7.250289905	27.20000076	13.41649	27.3	26.31611	27.4	43.91674	27.4	81.07055	27.1	7.009172	27.2	13.0942	27.3	25.71145	27.4	44.16369	27.4	83.47807
27.19999695	7.261085232	27.29999924	13.43436	27.4	26.34386	27.5	43.96332	27.5	81.15275	27.2	7.017685	27.3	13.1098	27.4	25.74064	27.5	44.21427	27.5	83.56816
27.29999542	7.270164569	27.39999771	13.45027	27.5	26.37301	27.6	44.00449	27.6	81.23574	27.3	7.026189	27.4	13.12538	27.5	25.76976	27.6	44.26469	27.6	83.6578
27.40000153	7.280568429	27.50000381	13.46046	27.6	26.39978	27.7	44.04695	27.7	81.32063	27.4	7.034683	27.5	13.14093	27.6	25.79884	27.7	44.31495	27.7	83.747
27.5	7.290589473	27.60000229	13.4714	27.7	26.42905	27.8	44.08687	27.8	81.40459	27.5	7.043167	27.6	13.15646	27.7	25.82785	27.8	44.36506	27.8	83.83576
27.59999847	7.297995609	27.70000076	13.48502	27.8	26.45157	27.9	44.12491	27.9	81.4852	27.6	7.051642	27.7	13.17196	27.8	25.85681	27.9	44.41502	27.9	83.92409
27.69999695	7.302188743	27.79999924	13.49551	27.9	26.47596	28	44.16853	28	81.56438	27.7	7.060106	27.8	13.18745	27.9	25.88571	28	44.46482	28	84.01197
27.79999542	7.309968932	27.89999771	13.50413	28	26.50024	28.1	44.21257	28.1	81.64585	27.8	7.068561	27.9	13.2029	28	25.91455	28.1	44.51446	28.1	84.0994
27.90000153	7.312980579	28.00000381	13.51231	28.1	26.52572	28.2	44.25962	28.2	81.73028	27.9	7.077007	28	13.21834	28.1	25.94334	28.2	44.56395	28.2	84.1864
28	7.321882346	28.10000229	13.52559	28.2	26.55458	28.3	44.29868	28.3	81.81019	28	7.085442	28.1	13.23374	28.2	25.97207	28.3	44.61328	28.3	84.27295
28.09999847	7.331653944	28.20000076	13.54105	28.3	26.57822	28.4	44.3385	28.4	81.89136	28.1	7.093867	28.2	13.24913	28.3	26.00074	28.4	44.66247	28.4	84.35907
28.19999695	7.336864445	28.29999924	13.54926	28.4	26.60223	28.5	44.37818	28.5	81.96616	28.2	7.102283	28.3	13.26449	28.4	26.02936	28.5	44.71149	28.5	84.44474
28.29999542	7.33973974	28.39999771	13.55936	28.5	26.62803	28.6	44.4274	28.6	82.04649	28.3	7.11069	28.4	13.27983	28.5	26.05792	28.6	44.76036	28.6	84.52997
28.40000153	7.345301623	28.50000381	13.57579	28.6	26.65557	28.7	44.47224	28.7	82.13178	28.4	7.119087	28.5	13.29514	28.6	26.08642	28.7	44.80908	28.7	84.61477
28.5	7.357203567	28.60000229	13.59306	28.7	26.68127	28.8	44.52185	28.8	82.21199	28.5	7.127474	28.6	13.31043	28.7	26.11487	28.8	44.85764	28.8	84.69912
28.59999847	7.369303014	28.70000076	13.60799	28.8	26.70637	28.9	44.56909	28.9	82.28963	28.6	7.135851	28.7	13.32569	28.8	26.14326	28.9	44.90605	28.9	84.78304
28.69999695	7.37714806	28.79999924	13.62521	28.9	26.72885	29	44.61722	29	82.37389	28.7	7.144218	28.8	13.34094	28.9	26.17159	29	44.9543	29	84.86651
28.79999542	7.387939081	28.89999771	13.64438	29	26.75384	29.1	44.66266	29.1	82.45394	28.8	7.152576	28.9	13.35616	29	26.19987	29.1	45.0024	29.1	84.94954
28.90000153	7.398158229	29.00000381	13.66227	29.1	26.77408	29.2	44.7047	29.2	82.52935	28.9	7.160925	29	13.37135	29.1	26.22809	29.2	45.05035	29.2	85.03214
29	7.411967363	29.10000229	13.6763	29.2	26.79893	29.3	44.74589	29.3	82.61164	29	7.169264	29.1	13.38652	29.2	26.25625	29.3	45.09814	29.3	85.1143
29.09999847	7.420288796	29.20000076	13.69207	29.3	26.8228	29.4	44.78997	29.4	82.69246	29.1	7.177592	29.2	13.40167	29.3	26.28435	29.4	45.14578	29.4	85.19602
29.19999695	7.429447451	29.29999924	13.70762	29.4	26.84937	29.5	44.83478	29.5	82.77189	29.2	7.185912	29.3	13.41679	29.4	26.31241	29.5	45.19327	29.5	85.27731
29.29999542	7.438771807	29.39999771	13.72643	29.5	26.88079	29.6	44.88311	29.6	82.85723	29.3	7.194222	29.4	13.43189	29.5	26.3404	29.6	45.2406	29.6	85.35815
29.40000153	7.447145644	29.50000381	13.73823	29.6	26.91118	29.7	44.92441	29.7	82.93824	29.4	7.202522	29.5	13.44697	29.6	26.36834	29.7	45.28778	29.7	85.43856
29.5	7.456560491	29.60000229	13.75454	29.7	26.93847	29.8	44.96887	29.8	83.01803	29.5	7.210813	29.6	13.46202	29.7	26.39622	29.8	45.33481	29.8	85.51853
29.59999847	7.46884921	29.70000076	13.76719	29.8	26.96248	29.9	45.01272	29.9	83.10065	29.6	7.219094	29.7	13.47705	29.8	26.42405	29.9	45.38168	29.9	85.59808
29.69999695	7.47911028	29.79999924	13.78219	29.9	26.99127	30	45.05343	30	83.1781	29.7	7.227365	29.8	13.49205	29.9	26.45182	30	45.4284	30	85.67718
29.79999542	7.486016509	29.89999771	13.79641	30	27.01179	30.1	45.09495	30.1	83.26008	29.8	7.235627	29.9	13.50703	30	26.47953	30.1	45.47497	30.1	85.75584

29.90000153	7.492203117	30.00000381	13.80982	30.1	27.0326	30.2	45.14172	30.2	83.34652	29.9	7.24388	30	13.52199	30.1	26.50719	30.2	45.52139	30.2	85.83407
30	7.497796377	30.10000229	13.82118	30.2	27.05318	30.3	45.18728	30.3	83.43058	30	7.252123	30.1	13.53693	30.2	26.53479	30.3	45.56766	30.3	85.91187
30.09999847	7.503211442	30.20000076	13.83463	30.3	27.07507	30.4	45.2345	30.4	83.51814	30.1	7.260356	30.2	13.55184	30.3	26.56234	30.4	45.61377	30.4	85.98924
30.19999695	7.513352961	30.29999924	13.84998	30.4	27.10102	30.5	45.27657	30.5	83.59842	30.2	7.26858	30.3	13.56672	30.4	26.58983	30.5	45.65973	30.5	86.06617
30.29999542	7.522529042	30.39999771	13.86714	30.5	27.12118	30.6	45.31851	30.6	83.67438	30.3	7.276794	30.4	13.58159	30.5	26.61727	30.6	45.70555	30.6	86.14267
30.40000153	7.534655383	30.50000381	13.8803	30.6	27.14428	30.7	45.36281	30.7	83.75409	30.4	7.284999	30.5	13.59643	30.6	26.64464	30.7	45.7512	30.7	86.21873
30.5	7.539404756	30.60000229	13.89196	30.7	27.16329	30.8	45.41111	30.8	83.83314	30.5	7.293195	30.6	13.61125	30.7	26.67197	30.8	45.79671	30.8	86.29437
30.59999847	7.545330597	30.70000076	13.90521	30.8	27.18572	30.9	45.45783	30.9	83.91356	30.6	7.30138	30.7	13.62604	30.8	26.69924	30.9	45.84207	30.9	86.36958
30.69999695	7.549429372	30.79999924	13.9177	30.9	27.2069	31	45.49957	31	83.99306	30.7	7.309557	30.8	13.64081	30.9	26.72645	31	45.88728	31	86.44435
30.79999542	7.55667011	30.89999771	13.93207	31	27.23429	31.1	45.54084	31.1	84.07335	30.8	7.317724	30.9	13.65556	31	26.75361	31.1	45.93233	31.1	86.5187
30.90000153	7.561449691	31.00000381	13.94789	31.1	27.25633	31.2	45.57688	31.2	84.15136	30.9	7.325881	31	13.67029	31.1	26.78071	31.2	45.97724	31.2	86.59261
31	7.566226895	31.10000229	13.9608	31.2	27.27557	31.3	45.61774	31.3	84.23219	31	7.334029	31.1	13.68499	31.2	26.80776	31.3	46.02199	31.3	86.6661
31.09999847	7.570770004	31.20000076	13.96748	31.3	27.30043	31.4	45.66376	31.4	84.3095	31.1	7.342168	31.2	13.69967	31.3	26.83475	31.4	46.0666	31.4	86.73917
31.19999695	7.572808731	31.29999924	13.98187	31.4	27.32345	31.5	45.7056	31.5	84.38618	31.2	7.350297	31.3	13.71432	31.4	26.86169	31.5	46.11105	31.5	86.8118
31.29999542	7.579184685	31.39999771	14.00012	31.5	27.34437	31.6	45.74398	31.6	84.46153	31.3	7.358416	31.4	13.72896	31.5	26.88857	31.6	46.15536	31.6	86.88401
31.40000153	7.588096002	31.50000381	14.01472	31.6	27.36402	31.7	45.78615	31.7	84.54198	31.4	7.366527	31.5	13.74356	31.6	26.91539	31.7	46.19951	31.7	86.95579
31.5	7.592032434	31.60000229	14.02676	31.7	27.38701	31.8	45.82638	31.8	84.62195	31.5	7.374628	31.6	13.75815	31.7	26.94217	31.8	46.24352	31.8	87.02715
31.59999847	7.597984137	31.70000076	14.04288	31.8	27.40781	31.9	45.85748	31.9	84.69265	31.6	7.382719	31.7	13.77271	31.8	26.96888	31.9	46.28737	31.9	87.09809
31.69999695	7.609906259	31.79999924	14.05391	31.9	27.42377	32	45.89625	32	84.76294	31.7	7.390801	31.8	13.78725	31.9	26.99554	32	46.33108	32	87.1686
31.79999542	7.616595463	31.89999771	14.06294	32	27.4474	32.1	45.93962	32.1	84.84175	31.8	7.398874	31.9	13.80177	32	27.02215	32.1	46.37464	32.1	87.23869
31.90000153	7.617610214	32.00000381	14.07651	32.1	27.47634	32.2	45.98213	32.2	84.92221	31.9	7.406938	32	13.81626	32.1	27.0487	32.2	46.41805	32.2	87.30836
32	7.623856253	32.10000229	14.09208	32.2	27.50079	32.3	46.02468	32.3	84.99667	32	7.414992	32.1	13.83074	32.2	27.0752	32.3	46.46131	32.3	87.37761
32.09999847	7.633238692	32.20000076	14.10656	32.3	27.52628	32.4	46.06418	32.4	85.06932	32.1	7.423036	32.2	13.84518	32.3	27.10164	32.4	46.50443	32.4	87.44644
32.19999695	7.642314861	32.29999924	14.11577	32.4	27.54921	32.5	46.10512	32.5	85.14437	32.2	7.431072	32.3	13.85961	32.4	27.12803	32.5	46.54739	32.5	87.51485
32.29999542	7.648449216	32.39999771	14.12742	32.5	27.57164	32.6	46.14789	32.6	85.22244	32.3	7.439097	32.4	13.87401	32.5	27.15437	32.6	46.59021	32.6	87.58284
32.40000153	7.655168466	32.50000381	14.13858	32.6	27.59503	32.7	46.19098	32.7	85.29558	32.4	7.447115	32.5	13.88839	32.6	27.18064	32.7	46.63288	32.7	87.65041
32.5	7.659039502	32.60000229	14.15079	32.7	27.61785	32.8	46.23456	32.8	85.37228	32.5	7.455122	32.6	13.90275	32.7	27.20687	32.8	46.6754	32.8	87.71757
32.59999847	7.666402687	32.70000076	14.16329	32.8	27.64567	32.9	46.2737	32.9	85.44748	32.6	7.46312	32.7	13.91708	32.8	27.23304	32.9	46.71778	32.9	87.78432
32.69999695	7.671045665	32.79999924	14.17796	32.9	27.67046	33	46.31886	33	85.52257	32.7	7.471108	32.8	13.93139	32.9	27.25916	33	46.76001	33	87.85065
32.79999542	7.679377613	32.89999771	14.19233	33	27.69427	33.1	46.35887	33.1	85.59592	32.8	7.479088	32.9	13.94568	33	27.28522	33.1	46.80209	33.1	87.91656
32.90000153	7.688473218	33.00000381	14.20798	33.1	27.71776	33.2	46.39205	33.2	85.66935	32.9	7.487058	33	13.95995	33.1	27.31123	33.2	46.84402	33.2	87.98206
33	7.698314797	33.10000229	14.22212	33.2	27.74033	33.3	46.42824	33.3	85.74178	33	7.495019	33.1	13.97419	33.2	27.33718	33.3	46.88581	33.3	88.04715
33.09999847	7.704108463	33.20000076	14.23293	33.3	27.76066	33.4	46.46297	33.4	85.81005	33.1	7.502971	33.2	13.98841	33.3	27.36308	33.4	46.92745	33.4	88.11184
33.19999695	7.707542859	33.29999924	14.24718	33.4	27.77806	33.5	46.50111	33.5	85.88224	33.2	7.510913	33.3	14.00261	33.4	27.38893	33.5	46.96895	33.5	88.17611
33.29999542	7.712249075	33.39999771	14.25479	33.5	27.79986	33.6	46.53794	33.6	85.94839	33.3	7.518846	33.4	14.01678	33.5	27.41472	33.6	47.0103	33.6	88.23997
33.40000153	7.715896389	33.50000381	14.26876	33.6	27.81911	33.7	46.57558	33.7	86.01913	33.4	7.526771	33.5	14.03094	33.6	27.44046	33.7	47.0515	33.7	88.30343
33.5	7.722898407	33.60000229	14.2849	33.7	27.84264	33.8	46.61394	33.8	86.08357	33.5	7.534685	33.6	14.04507	33.7	27.46614	33.8	47.09256	33.8	88.36648
33.59999847	7.731948922	33.70000076	14.29934	33.8	27.86245	33.9	46.65348	33.9	86.14787	33.6	7.542591	33.7	14.05917	33.8	27.49177	33.9	47.13348	33.9	88.42913
33.69999695	7.739889726	33.79999924	14.31111	33.9	27.88448	34	46.69908	34	86.21855	33.7	7.550487	33.8	14.07326	33.9	27.51735	34	47.17425	34	88.49137
33.79999542	7.748349909	33.89999771	14.32284	34	27.90946	34.1	46.73828	34.1	86.28969	33.8	7.558374	33.9	14.08732	34	27.54287	34.1	47.21487	34.1	88.5532
33.90000153	7.753710219	34.00000381	14.34163	34.1	27.93035	34.2	46.77931	34.2	86.36328	33.9	7.566252	34	14.10136	34.1	27.56834	34.2	47.25535	34.2	88.61464
34	7.765025848	34.10000229	14.35749	34.2	27.95754	34.3	46.81763	34.3	86.4344	34	7.574121	34.1	14.11538	34.2	27.59376	34.3	47.29569	34.3	88.67567
34.09999847	7.767614238	34.20000076	14.37266	34.3	27.98232	34.4	46.85852	34.4	86.50821	34.1	7.58198	34.2	14.12938	34.3	27.61912	34.4	47.33588	34.4	88.73631
34.19999695	7.77586447	34.29999924	14.38379	34.4	28.00728	34.5	46.89824	34.5	86.57856	34.2	7.589831	34.3	14.14335	34.4	27.64443	34.5	47.37593	34.5	88.79654
34.29999542	7.783845032	34.39999771	14.40142	34.5	28.02805	34.6	46.94091	34.6	86.65032	34.3	7.597672	34.4	14.1573	34.5	27.66968	34.6	47.41583	34.6	88.85638
34.40000153	7.795725424	34.50000381	14.41512	34.6	28.04976	34.7	46.97953	34.7	86.7229	34.4	7.605504	34.5	14.17123	34.6	27.69489	34.7	47.45559	34.7	88.91582
34.5	7.803481323	34.60000229	14.42746	34.7	28.07286	34.8	47.01871	34.8	86.79472	34.5	7.613327	34.6	14.18513	34.7	27.72004	34.8	47.49521	34.8	88.97487
34.59999847	7.808696196	34.70000076	14.43519	34.8	28.09418	34.9	47.05419	34.9	86.86597	34.6	7.621141	34.7	14.19902	34.8	27.74513	34.9	47.53469	34.9	89.03353
34.69999695	7.817129409	34.79999924	14.44793	34.9	28.11561	35	47.09198	35	86.94119	34.7	7.628946	34.8	14.21288	34.9	27.77018	35	47.57402	35	89.09179
34.79999542	7.822863783	34.89999771	14.45638	35	28.14162	35.1	47.12692	35.1	87.01251	34.8	7.636741	34.9	14.22672	35	27.79517	35.1	47.61321	35.1	89.14966
34.90000153	7.828695934	35.00000381	14.46744	35.1	28.16597	35.2	47.16345	35.2	87.08806	34.9	7.644528	35	14.24054	35.1	27.82011	35.2	47.65226	35.2	89.20714
35	7.836728899	35.10000229	14.47842	35.2	28.1912	35.3	47.19773	35.3	87.16341	35	7.652306	35.1	14.25433	35.2	27.84499	35.3	47.69116	35.3	89.26423
35.09999847	7.839184818	35.20000076	14.48967	35.3	28.21379	35.4	47.23536	35.4	87.23734	35.1	7.660074	35.2	14.2681	35.3	27.86982	35.4	47.72993	35.4	89.32093
35.19999695	7.8456459	35.29999924	14.49843	35.4	28.23508	35.5	47.27553	35.5	87.31264	35.2	7.667833	35.3	14.28185	35.4	27.8946	35.5	47.76855	35.5	89.37725
35.29999542	7.847842899	35.39999771	14.5101	35.5	28.25694	35.6	47.31557	35.6	87.38602	35.3	7.675583	35.4	14.29558	35.5	27.91933	35.6	47.80703	35.6	89.43318
35.40000153	7.85085901	35.50000381	14.52241	35.6	28.27618	35.7	47.34981	35.7	87.45268	35.4	7.683325	35.5	14.30929	35.6	27.944	35.7	47.84537	35.7	89.48873
35.5	7.857089026	35.60000229	14.53603	35.7	28.29771	35.8	47.38479	35.8	87.52447	35.5	7.691057	35.6	14.32297	35.7	27.96862	35.8	47.88357	35.8	89.54389
35.59999847	7.862919065	35.70000076	14.54527	35.8	28.32071	35.9	47.42357	35.9	87.59939	35.6	7.69878	35.7	14.33664	35.8	27.99319	35.9	47.92163	35.9	89.59868
35.69999695	7.867942741	35.79999924	14.55989	35.9	28.34498	36	47.46222	36	87.66849	35.7	7.706494	35.8	14.35028	35.9	28.01771	36	47.95955	36	89.65308
35.79999542	7.874029621	35.89999771	14.575	36	28.36466	36.1	47.50271	36.1	87.74265	35.8	7.714199	35.9	14.36389	36	28.04217	36.1	47.99733	36.1	89.70711
35.90000153	7.88386484	36.00000381	14.5922	36.1	28.38767	36.2	47.54339	36.2	87.81051	35.9	7.721895	36	14.37749	36.1	28.06658	36.2	48.03497	36.2	89.76075
36	7.892260504	36.10000229	14.6043	36.2	28.41008	36.3	47.57852	36.3	87.87642	36	7.729582	36.1	14.39107	36.2	28.09094	36.3	48.07247	36.3	89.81403
36.09999847	7.89982811	36.20000076	14.61704	36.3	28.42843	36.4	47.61426	36.4	87.94525	36.1	7.73726	36.2	14.40462	36.3	28.11525	36.4	48.10984	36.4	89.86693
36.19999695	7.906010851	36.29999924	14.62681	36.4	28.45116	36.5	47.65022	36.5	88.01025	36.2	7.744929	36.3	14.41815	36.4	28.13951	36.5	48.14706	36.5	89.91945
36.29999542	7.9138936	36.39999771	14.63555	36.5	28.47195	36.6	47.68987	36.6	88.08262	36.3	7.752589	36.4	14.43166	36.5	28.16371	36.6	48.18414	36.6	89.97161
36.40000153	7.921295485	36.50000381	14.64526	36.6	28.49742	36.7	47.72757	36.7	88.15457	36.4	7.760241	36.5	14.44515	36.6	28.18786	36.7	48.22109	36.7	90.02339
36.5	7.924466708	36.60000229	14.65959	36.7	28.51626	36.8	47.76667	36.8	88.22567	36.5	7.767883	36.6	14.45861	36.7	28.21196	36.8	48.25789	36.8	90.0748
36.59999847	7.935088835	36.70000076	14.67407	36.8	28.5399	36.9	47.80333	36.9	88.29461	36.6	7.775516	36.7	14.47205	36.8	28.236	36.9	48.29456	36.9	90.12585
36.69999695	7.942458648	36.79999924	14.6871	36.9	28.56025	37	47.83821	37	88.36531	36.7	7.78314	36.8	14.48548	36.9	28.26	37	48.33109	37	90.17653
36.79999542	7.9546702	36.89999771	14.70123	37	28.58183	37.1	47.87638	37.1	88.43861	36.8	7.790755	36.9	14.49888	37	28.28395	37.1	48.36749	37.1	90.22685
36.90000153	7.959732581	37.00000381	14.71128	37.1	28.60096	37.2	47.91124	37.2	88.50808	36.9	7.798362	37	14.51226	37.1	28.30784	37.2	48.40374	37.2	90.2768
37	7.969548499	37.10000229	14.7223	37.2	28.62189	37.3	47.95141	37.3	88.5814	37	7.805959	37.1	14.52561	37.2	28.33168	37.3	48.43986	37.3	90.3264
37.09999847	7.975650528	37.20000076	14.73419	37.3	28.64968	37.4	47.99032	37.4	88.65514	37.1	7.813548	37.2	14.53895	37.3	28.35547	37.4	48.47585	37.4	90.37563
37.19999695	7.98196772	37.29999924	14.756	37.4	28.6705	37.5	48.02746	37.5	88.72473	37.2	7.821127	37.3	14.55226	37.4	28.37921	37.5	48.51169	37.5	90.42451
37.29999542	7.991924272	37.39999771	14.76938	37.5	28.69071	37.6	48.06605	37.6	88.79582	37.3	7.828698	37.4	14.56556	37.5	28.40289	37.6	48.5474	37.6	90.47302
37.40000153	7.999728577	37.50000381	14.78174	37.6	28.7134	37.7	48.10488	37.7	88.87167	37.4	7.836261	37.5	14.57883	37.6	28.42653	37.7	48.58298	37.7	90.52118
37.5	8.007928647	37.60000229	14.79279	37.7	28.73723	37.8	48.14193	37.8	88.94288	37.5	7.843814	37.6	14.59207	37.7	28.45011	37.8	48.61841	37.8	90.56899
37.59999847	8.015437147	37.70000076	14.80583	37.8	28.7572	37.9	48.176	37.9	89.01158	37.6	7.851358	37.7	14.6053	37.8	28.47364	37.9	48.65372	37.9	90.61645
37.69999695	8.024833623	37.79999924	14.81841	37.9	28.77669	38	48.21358	38	89.08335	37.7	7.858893	37.8	14.61851	37.9	28.49712	38	48.68889	38	90.66355
37.79999542	8.02740677	37.89999771	14.83233	38	28.79979	38.1	48.24785	38.1	89.15381	37.8	7.866419	37.9	14.63169	38	28.52055	38.1	48.72392	38.1	90.7103
37.90000153	8.036204583	38.00000381	14.84587	38.1	28.82192	38.2	48.28034	38.2	89.22123	37.9	7.873937	38	14.64486	38.1	28.54393	38.2	48.75882	38.2	90.75671
38	8.047857086	38.10000229	14.85862	38.2	28.83958	38.3	48.31587	38.3	89.28956	38	7.881446	38.1	14.658	38.2	28.56726	38.3	48.79359	38.3	90.80277
38.09999847	8.056470004	38.20000076	14.86999	38.3	28.85832	38.4	48.35032	38.4	89.35468	38.1	7.888946	38.2	14.67112	38.3	28.59054	38.4	48.82822	38.4	90.84849
38.19999695	8.066037656	38.29999924	14.87889	38.4	28.87564	38.5	48.3924	38.5	89.42286	38.2	7.896437	38.3	14.68422	38.4	28.61376	38.5	48.86272	38.5	90.89386
38.29999542	8.071305378	38.39999771	14.8901	38.5	28.89409	38.6	48.42804	38.6	89.49083	38.3	7.90392	38.4	14.6973	38.5	28.63694	38.6	48.89708	38.6	90.93889
38.40000153	8.077910502	38.50000381	14.90181	38.6	28.91467	38.7	48.46576	38.7	89.55776	38.4	7.911394	38.5	14.71035	38.6	28.66006	38.7	48.93131	38.7	90.98358
38.5	8.085134879	38.60000229	14.91366	38.7	28.93608	38.8	48.50455	38.8	89.6288	38.5	7.918859	38.6	14.72339	38.7	28.68314	38.8	48.96541	38.8	91.02793
38.59999847	8.094342308	38.70000076	14.92809	38.8	28.95559	38.9	48.54375	38.9	89.69499	38.6	7.926315	38.7	14.7364	38.8	28.70616	38.9	48.99938	38.9	91.07194
38.69999695	8.099935757	38.79999924	14.94261	38.9	28.97451	39	48.58008	39	89.75833	38.7	7.933762	38.8	14.7494	38.9	28.72914	39	49.03321	39	91.11562
38.79999542	8.108547215	38.89999771	14.95695	39	28.99281	39.1	48.61145	39.1	89.82179	38.8	7.9412	38.9	14.76237	39	28.75206	39.1	49.06691	39.1	91.15897
38.90000153	8.115869516	39.00000381	14.96822	39.1	29.00736	39.2	48.64322	39.2	89.88965	38.9	7.94863	39	14.77532	39.1	28.77493	39.2	49.10048	39.2	91.20198
39	8.120319862	39.10000229	14.97808	39.2	29.0266	39.3	48.68119	39.3	89.95833	39	7.956051	39.1	14.78825	39.2	28.79775	39.3	49.13392	39.3	91.24466
39.09999847	8.122902942	39.20000076	14.98817	39.3	29.04742	39.4	48.71726	39.4	90.02586	39.1	7.963464	39.2	14.80116	39.3	28.82053	39.4	49.16723	39.4	91.28702
39.19999695	8.131236099	39.29999924	15.00102	39.4	29.06575	39.5	48.7536	39.5	90.09244	39.2	7.970867	39.3	14.81404	39.4	28.84325	39.5	49.2004	39.5	91.32904
39.29999542	8.141847571	39.39999771	15.0113	39.5	29.08415	39.6	48.78963	39.6	90.15709	39.3	7.978262	39.4	14.82691	39.5	28.86592	39.6	49.23345	39.6	91.37074
39.40000153	8.147889347	39.50000381	15.02656	39.6	29.1036	39.7	48.83067	39.7	90.22641	39.4	7.985649	39.5	14.83976	39.6	28.88854	39.7	49.26636	39.7	91.41212
39.5	8.155344906	39.60000229	15.03478	39.7	29.12597	39.8	48.86784	39.8	90.29319	39.5	7.993026	39.6	14.85258	39.7	28.91111	39.8	49.29915	39.8	91.45317
39.59999847	8.160967438	39.70000076	15.047	39.8	29.1474	39.9	48.90342	39.9	90.36249	39.6	8.000395	39.7	14.86538	39.8	28.93364	39.9	49.33181	39.9	91.49391
39.69999695	8.169487409	39.79999924	15.06029	39.9	29.16945	40	48.93773	40	90.43374	39.7	8.007755	39.8	14.87816	39.9	28.95611	40	49.36434	40	91.53432
39.79999542	8.177116212	39.89999771	15.07501	40	29.18758	40.1	48.97429	40.1	90.50193	39.8	8.015106	39.9	14.89093	40	28.97853	40.1	49.39673	40.1	91.57442
39.90000153	8.186997789	40.00000381	15.08548	40.1	29.20476	40.2	49.01716	40.2	90.57079	39.9	8.02245	40	14.90367	40.1	29.0009	40.2	49.429	40.2	91.6142
40	8.194394146	40.10000229	15.09384	40.2	29.2235	40.3	49.05672	40.3	90.6374	40	8.029784	40.1	14.91639	40.2	29.02323	40.3	49.46114	40.3	91.65366
40.09999847	8.201395064	40.20000076	15.10772	40.3	29.24188	40.4	49.09039	40.4	90.70393	40.1	8.037109	40.2	14.92908	40.3	29.0455	40.4	49.49316	40.4	91.69282
40.19999695	8.213331226	40.29999924	15.11777	40.4	29.26042	40.5	49.12569	40.5	90.76794	40.2	8.044426	40.3	14.94176	40.4	29.06773	40.5	49.52504	40.5	91.73166
40.29999542	8.220285965	40.39999771	15.1303	40.5	29.27963	40.6	49.16031	40.6	90.83793	40.3	8.051734	40.4	14.95442	40.5	29.0899	40.6	49.5568	40.6	91.77019
40.40000153	8.226651179	40.50000381	15.14014	40.6	29.30028	40.7	49.19806	40.7	90.9078	40.4	8.059034	40.5	14.96706	40.6	29.11203	40.7	49.58843	40.7	91.80842
40.5	8.232180115	40.60000229	15.1548	40.7	29.31905	40.8	49.23378	40.8	90.97647	40.5	8.066325	40.6	14.97967	40.7	29.1341	40.8	49.61993	40.8	91.84634
40.59999847	8.240711953	40.70000076	15.16756	40.8	29.33628	40.9	49.27157	40.9	91.041	40.6	8.073608	40.7	14.99227	40.8	29.15613	40.9	49.65131	40.9	91.88396
40.69999695	8.25066329	40.79999924	15.18398	40.9	29.35846	41	49.30928	41	91.10858	40.7	8.080881	40.8	15.00484	40.9	29.17811	41	49.68256	41	91.92127
40.79999542	8.26025974	40.89999771	15.19711	41	29.3752	41.1	49.34423	41.1	91.17143	40.8	8.088147	40.9	15.01739	41	29.20004	41.1	49.71368	41.1	91.95828
40.90000153	8.268151836	41.00000381	15.20774	41.1	29.38976	41.2	49.37701	41.2	91.23592	40.9	8.095404	41	15.02993	41.1	29.22192	41.2	49.74468	41.2	91.99499
41	8.275180807	41.10000229	15.21858	41.2	29.4054	41.3	49.41077	41.3	91.29457	41	8.102652	41.1	15.04244	41.2	29.24375	41.3	49.77555	41.3	92.03141
41.09999847	8.281563512	41.20000076	15.22904	41.3	29.42531	41.4	49.44502	41.4	91.35858	41.1	8.109891	41.2	15.05493	41.3	29.26553	41.4	49.8063	41.4	92.06753
41.19999695	8.291460001	41.29999924	15.24141	41.4	29.44338	41.5	49.48271	41.5	91.42993	41.2	8.117122	41.3	15.0674	41.4	29.28726	41.5	49.83692	41.5	92.10336
41.29999542	8.29856841	41.39999771	15.25307	41.5	29.46359	41.6	49.51307	41.6	91.49596	41.3	8.124344	41.4	15.07985	41.5	29.30895	41.6	49.86741	41.6	92.13889

41.40000153	8.308201653	41.50000381	15.26312	41.6	29.48196	41.7	49.54855	41.7	91.5565	41.4	8.131559	41.5	15.09228	41.6	29.33058	41.7	49.89779	41.7	92.17413
41.5	8.318215222	41.60000229	15.27202	41.7	29.50258	41.8	49.58647	41.8	91.6176	41.5	8.138764	41.6	15.10469	41.7	29.35217	41.8	49.92804	41.8	92.20909
41.59999847	8.324411101	41.70000076	15.28341	41.8	29.52008	41.9	49.61452	41.9	91.6751	41.6	8.145961	41.7	15.11708	41.8	29.37371	41.9	49.95816	41.9	92.24375
41.69999695	8.334896752	41.79999924	15.29714	41.9	29.53182	42	49.64718	42	91.73851	41.7	8.153149	41.8	15.12945	41.9	29.3952	42	49.98817	42	92.27813
41.79999542	8.343293246	41.89999771	15.3028	42	29.54938	42.1	49.68094	42.1	91.80273	41.8	8.160329	41.9	15.1418	42	29.41664	42.1	50.01804	42.1	92.31223
41.90000153	8.344366421	42.00000381	15.3109	42.1	29.5629	42.2	49.71715	42.2	91.86795	41.9	8.167501	42	15.15413	42.1	29.43803	42.2	50.0478	42.2	92.34604
42	8.350223144	42.10000229	15.31843	42.2	29.58665	42.3	49.75529	42.3	91.93597	42	8.174664	42.1	15.16643	42.2	29.45938	42.3	50.07743	42.3	92.37958
42.09999847	8.353340483	42.20000076	15.33101	42.3	29.60479	42.4	49.79489	42.4	92.00411	42.1	8.181818	42.2	15.17872	42.3	29.48068	42.4	50.10695	42.4	92.41283
42.19999695	8.362779882	42.29999924	15.34371	42.4	29.62292	42.5	49.83316	42.5	92.0736	42.2	8.188964	42.3	15.19099	42.4	29.50193	42.5	50.13634	42.5	92.44581
42.29999542	8.37392162	42.39999771	15.35648	42.5	29.6449	42.6	49.86658	42.6	92.13608	42.3	8.196102	42.4	15.20323	42.5	29.52313	42.6	50.1656	42.6	92.47851
42.40000153	8.381813097	42.50000381	15.37614	42.6	29.66164	42.7	49.89683	42.7	92.19765	42.4	8.203231	42.5	15.21546	42.6	29.54428	42.7	50.19475	42.7	92.51094
42.5	8.391345951	42.60000229	15.3884	42.7	29.67758	42.8	49.93042	42.8	92.25946	42.5	8.210352	42.6	15.22767	42.7	29.56538	42.8	50.22378	42.8	92.5431
42.59999847	8.398074918	42.70000076	15.40277	42.8	29.69766	42.9	49.96599	42.9	92.31735	42.6	8.217464	42.7	15.23985	42.8	29.58644	42.9	50.25268	42.9	92.57498
42.69999695	8.402790739	42.79999924	15.41542	42.9	29.71588	43	50.00308	43	92.38018	42.7	8.224568	42.8	15.25202	42.9	29.60745	43	50.28147	43	92.6066
42.79999542	8.409995835	42.89999771	15.42729	43	29.73685	43.1	50.03945	43.1	92.44352	42.8	8.231663	42.9	15.26416	43	29.62841	43.1	50.31013	43.1	92.63795
42.90000153	8.413856815	43.00000381	15.43945	43.1	29.75605	43.2	50.07491	43.2	92.51009	42.9	8.238751	43	15.27629	43.1	29.64933	43.2	50.33868	43.2	92.66903
43	8.423218812	43.10000229	15.45523	43.2	29.77751	43.3	50.11276	43.3	92.57304	43	8.245829	43.1	15.28839	43.2	29.67019	43.3	50.36711	43.3	92.69985
43.09999847	8.428790623	43.20000076	15.47053	43.3	29.79621	43.4	50.14483	43.4	92.63433	43.1	8.252899	43.2	15.30048	43.3	29.69101	43.4	50.39542	43.4	92.73041
43.19999695	8.438539659	43.29999924	15.48423	43.4	29.8098	43.5	50.17605	43.5	92.69949	43.2	8.259961	43.3	15.31254	43.4	29.71179	43.5	50.4236	43.5	92.76071
43.29999542	8.446412447	43.39999771	15.48869	43.5	29.82662	43.6	50.20727	43.6	92.76408	43.3	8.267014	43.4	15.32459	43.5	29.73251	43.6	50.45167	43.6	92.79075
43.40000153	8.451034722	43.50000381	15.49517	43.6	29.84779	43.7	50.24134	43.7	92.82706	43.4	8.27406	43.5	15.33661	43.6	29.75319	43.7	50.47963	43.7	92.82053
43.5	8.455487228	43.60000229	15.50479	43.7	29.86598	43.8	50.272	43.8	92.88692	43.5	8.281097	43.6	15.34862	43.7	29.77382	43.8	50.50746	43.8	92.85005
43.59999847	8.461346165	43.70000076	15.51623	43.8	29.87934	43.9	50.30764	43.9	92.9489	43.6	8.288125	43.7	15.3606	43.8	29.7944	43.9	50.53518	43.9	92.87933
43.69999695	8.471012337	43.79999924	15.5265	43.9	29.89681	44	50.3437	44	93.01395	43.7	8.295145	43.8	15.37257	43.9	29.81494	44	50.56278	44	92.90835
43.79999542	8.476992872	43.89999771	15.53721	44	29.91293	44.1	50.37725	44.1	93.0793	43.8	8.302157	43.9	15.38451	44	29.83542	44.1	50.59026	44.1	92.93712
43.90000153	8.483293538	44.00000381	15.5505	44.1	29.93451	44.2	50.4097	44.2	93.14002	43.9	8.30916	44	15.39644	44.1	29.85587	44.2	50.61763	44.2	92.96564
44	8.486148117	44.10000229	15.56326	44.2	29.95426	44.3	50.4433	44.3	93.20512	44	8.316155	44.1	15.40834	44.2	29.87626	44.3	50.64488	44.3	92.99391
44.09999847	8.491841597	44.20000076	15.57366	44.3	29.9773	44.4	50.47485	44.4	93.26823	44.1	8.323142	44.2	15.42023	44.3	29.89661	44.4	50.67202	44.4	93.02194
44.19999695	8.500081079	44.29999924	15.58678	44.4	29.99876	44.5	50.50619	44.5	93.32862	44.2	8.330121	44.3	15.4321	44.4	29.91691	44.5	50.69904	44.5	93.04973
44.29999542	8.505920591	44.39999771	15.59882	44.5	30.01678	44.6	50.53196	44.6	93.38422	44.3	8.337091	44.4	15.44394	44.5	29.93717	44.6	50.72594	44.6	93.07727
44.40000153	8.5128859	44.50000381	15.61135	44.6	30.03413	44.7	50.56704	44.7	93.44727	44.4	8.344053	44.5	15.45577	44.6	29.95737	44.7	50.75273	44.7	93.10457
44.5	8.518474813	44.60000229	15.6212	44.7	30.05364	44.8	50.59709	44.8	93.50899	44.5	8.351007	44.6	15.46758	44.7	29.97753	44.8	50.77941	44.8	93.13163
44.59999847	8.518254606	44.70000076	15.63328	44.8	30.07321	44.9	50.6291	44.9	93.56935	44.6	8.357952	44.7	15.47936	44.8	29.99765	44.9	50.80597	44.9	93.15846
44.69999695	8.527748642	44.79999924	15.64381	44.9	30.09333	45	50.66035	45	93.63154	44.7	8.364889	44.8	15.49113	44.9	30.01772	45	50.83242	45	93.18505
44.79999542	8.534984037	44.89999771	15.65465	45	30.11136	45.1	50.70213	45.1	93.70018	44.8	8.371818	44.9	15.50288	45	30.03774	45.1	50.85875	45.1	93.21141
44.90000153	8.541132036	45.00000381	15.666	45.1	30.13283	45.2	50.74081	45.2	93.76501	44.9	8.378739	45	15.51461	45.1	30.05772	45.2	50.88497	45.2	93.23753
45	8.547110474	45.10000229	15.68108	45.2	30.15253	45.3	50.77832	45.3	93.8275	45	8.385652	45.1	15.52631	45.2	30.07765	45.3	50.91108	45.3	93.26343
45.09999847	8.55554755	45.20000076	15.69207	45.3	30.17215	45.4	50.80642	45.4	93.88735	45.1	8.392556	45.2	15.538	45.3	30.09753	45.4	50.93707	45.4	93.28909
45.19999695	8.561453821	45.29999924	15.70597	45.4	30.18957	45.5	50.84022	45.5	93.95152	45.2	8.399452	45.3	15.54967	45.4	30.11737	45.5	50.96296	45.5	93.31453
45.29999542	8.568340664	45.39999771	15.71398	45.5	30.2103	45.6	50.87308	45.6	94.01713	45.3	8.40634	45.4	15.56132	45.5	30.13716	45.6	50.98873	45.6	93.33974
45.40000153	8.577195389	45.50000381	15.72562	45.6	30.23117	45.7	50.90551	45.7	94.08143	45.4	8.41322	45.5	15.57295	45.6	30.15691	45.7	51.01439	45.7	93.36473
45.5	8.586869297	45.60000229	15.7357	45.7	30.25066	45.8	50.93604	45.8	94.14337	45.5	8.420091	45.6	15.58456	45.7	30.17661	45.8	51.03994	45.8	93.38949
45.59999847	8.595479746	45.70000076	15.74581	45.8	30.26774	45.9	50.9666	45.9	94.20362	45.6	8.426954	45.7	15.59615	45.8	30.19626	45.9	51.06537	45.9	93.41404
45.69999695	8.599246343	45.79999924	15.75278	45.9	30.28375	46	51.00383	46	94.26799	45.7	8.433809	45.8	15.60772	45.9	30.21587	46	51.0907	46	93.43836
45.79999542	8.604550556	45.89999771	15.75798	46	30.30448	46.1	51.04207	46.1	94.33419	45.8	8.440656	45.9	15.61928	46	30.23544	46.1	51.11592	46.1	93.46247
45.90000153	8.609443894	46.00000381	15.77086	46.1	30.32786	46.2	51.07089	46.2	94.39524	45.9	8.447496	46	15.63081	46.1	30.25495	46.2	51.14102	46.2	93.48635
46	8.617019957	46.10000229	15.78673	46.2	30.34936	46.3	51.10125	46.3	94.45376	46	8.454326	46.1	15.64232	46.2	30.27443	46.3	51.16602	46.3	93.51003
46.09999847	8.628446187	46.20000076	15.7986	46.3	30.36788	46.4	51.13621	46.4	94.51792	46.1	8.461149	46.2	15.65382	46.3	30.29385	46.4	51.19091	46.4	93.53349
46.19999695	8.634376256	46.29999924	15.80658	46.4	30.38618	46.5	51.1714	46.5	94.58213	46.2	8.467963	46.3	15.66529	46.4	30.31324	46.5	51.21569	46.5	93.55674
46.29999542	8.640556667	46.39999771	15.817	46.5	30.40189	46.6	51.20762	46.6	94.64745	46.3	8.474769	46.4	15.67675	46.5	30.33257	46.6	51.24036	46.6	93.57978
46.40000153	8.650187444	46.50000381	15.82805	46.6	30.42108	46.7	51.24265	46.7	94.71161	46.4	8.481568	46.5	15.68819	46.6	30.35186	46.7	51.26492	46.7	93.60261
46.5	8.657672236	46.60000229	15.83886	46.7	30.44443	46.8	51.27471	46.8	94.77392	46.5	8.488358	46.6	15.6996	46.7	30.37111	46.8	51.28937	46.8	93.62524
46.59999847	8.665076115	46.70000076	15.85013	46.8	30.46281	46.9	51.30428	46.9	94.83643	46.6	8.49514	46.7	15.711	46.8	30.39031	46.9	51.31372	46.9	93.64766
46.69999695	8.672571744	46.79999924	15.85951	46.9	30.48451	47	51.33501	47	94.90213	46.7	8.501914	46.8	15.72238	46.9	30.40947	47	51.33796	47	93.66988
46.79999542	8.675703888	46.89999771	15.86831	47	30.50287	47.1	51.37001	47.1	94.96149	46.8	8.508679	46.9	15.73374	47	30.42858	47.1	51.36209	47.1	93.69189
46.90000153	8.677306931	47.00000381	15.8785	47.1	30.52303	47.2	51.40315	47.2	95.02165	46.9	8.515438	47	15.74508	47.1	30.44765	47.2	51.38611	47.2	93.7137
47	8.684032659	47.10000229	15.89191	47.2	30.53896	47.3	51.43778	47.3	95.08439	47	8.522187	47.1	15.7564	47.2	30.46667	47.3	51.41003	47.3	93.73531
47.09999847	8.690432269	47.20000076	15.90306	47.3	30.55923	47.4	51.47184	47.4	95.14509	47.1	8.528929	47.2	15.76771	47.3	30.48565	47.4	51.43385	47.4	93.75673
47.19999695	8.6969257	47.29999924	15.916	47.4	30.57647	47.5	51.50945	47.5	95.20736	47.2	8.535662	47.3	15.77899	47.4	30.50458	47.5	51.45755	47.5	93.77795
47.29999542	8.702227629	47.39999771	15.92617	47.5	30.59702	47.6	51.54039	47.6	95.2673	47.3	8.542388	47.4	15.79025	47.5	30.52347	47.6	51.48115	47.6	93.79898
47.40000153	8.707869243	47.50000381	15.93857	47.6	30.61617	47.7	51.57888	47.7	95.3275	47.4	8.549106	47.5	15.8015	47.6	30.54231	47.7	51.50465	47.7	93.81981
47.5	8.715684947	47.60000229	15.95051	47.7	30.63432	47.8	51.61345	47.8	95.38909	47.5	8.555815	47.6	15.81273	47.7	30.56111	47.8	51.52804	47.8	93.84045
47.59999847	8.723730812	47.70000076	15.9634	47.8	30.65156	47.9	51.6505	47.9	95.44536	47.6	8.562517	47.7	15.82393	47.8	30.57986	47.9	51.55133	47.9	93.8609
47.69999695	8.730389531	47.79999924	15.97264	47.9	30.6698	48	51.6869	48	95.50359	47.7	8.56921	47.8	15.83512	47.9	30.59858	48	51.57452	48	93.88116
47.79999542	8.734752837	47.89999771	15.98126	48	30.6906	48.1	51.72396	48.1	95.56513	47.8	8.575896	47.9	15.84629	48	30.61724	48.1	51.59759	48.1	93.90123
47.90000153	8.738743982	48.00000381	15.99135	48.1	30.71592	48.2	51.75569	48.2	95.62404	47.9	8.582574	48	15.85744	48.1	30.63587	48.2	51.62057	48.2	93.92112
48	8.747530855	48.10000229	16.01098	48.2	30.73558	48.3	51.78426	48.3	95.68888	48	8.589243	48.1	15.86858	48.2	30.65444	48.3	51.64344	48.3	93.94082
48.09999847	8.757930375	48.20000076	16.02116	48.3	30.75186	48.4	51.81929	48.4	95.75488	48.1	8.595905	48.2	15.87969	48.3	30.67298	48.4	51.66622	48.4	93.96034
48.19999695	8.763971068	48.29999924	16.03004	48.4	30.77029	48.5	51.85225	48.5	95.82094	48.2	8.602558	48.3	15.89078	48.4	30.69147	48.5	51.68889	48.5	93.97968
48.29999542	8.76822472	48.39999771	16.04196	48.5	30.7875	48.6	51.88168	48.6	95.88374	48.3	8.609204	48.4	15.90186	48.5	30.70992	48.6	51.71145	48.6	93.99884
48.40000153	8.774409466	48.50000381	16.05547	48.6	30.80547	48.7	51.9127	48.7	95.94331	48.4	8.615842	48.5	15.91292	48.6	30.72832	48.7	51.73392	48.7	94.01782
48.5	8.779287582	48.60000229	16.06197	48.7	30.8267	48.8	51.94782	48.8	96.00121	48.5	8.622472	48.6	15.92395	48.7	30.74668	48.8	51.75628	48.8	94.03662
48.59999847	8.784180975	48.70000076	16.07385	48.8	30.84617	48.9	51.97977	48.9	96.06147	48.6	8.629093	48.7	15.93497	48.8	30.76499	48.9	51.77854	48.9	94.05525
48.69999695	8.790969793	48.79999924	16.07982	48.9	30.86787	49	52.00608	49	96.11909	48.7	8.635707	48.8	15.94597	48.9	30.78327	49	51.80071	49	94.0737
48.79999542	8.797929965	48.89999771	16.08938	49	30.88539	49.1	52.0361	49.1	96.17855	48.8	8.642313	48.9	15.95696	49	30.8015	49.1	51.82277	49.1	94.09198
48.90000153	8.805810162	49.00000381	16.09908	49.1	30.90286	49.2	52.0687	49.2	96.24071	48.9	8.648912	49	15.96792	49.1	30.81968	49.2	51.84473	49.2	94.11009
49	8.809135886	49.10000229	16.11136	49.2	30.92128	49.3	52.09965	49.3	96.29847	49	8.655502	49.1	15.97887	49.2	30.83782	49.3	51.86659	49.3	94.12802
49.09999847	8.814929237	49.20000076	16.11897	49.3	30.94102	49.4	52.12885	49.4	96.35952	49.1	8.662084	49.2	15.98979	49.3	30.85592	49.4	51.88835	49.4	94.14579
49.19999695	8.820522546	49.29999924	16.12822	49.4	30.95879	49.5	52.16249	49.5	96.42156	49.2	8.668659	49.3	16.0007	49.4	30.87398	49.5	51.91002	49.5	94.16339
49.29999542	8.829285717	49.39999771	16.13532	49.5	30.97917	49.6	52.19956	49.6	96.48079	49.3	8.675225	49.4	16.01159	49.5	30.89199	49.6	51.93158	49.6	94.18083
49.40000153	8.838105023	49.50000381	16.14704	49.6	31.00101	49.7	52.23626	49.7	96.54406	49.4	8.681784	49.5	16.02246	49.6	30.90996	49.7	51.95305	49.7	94.1981
49.5	8.845856491	49.60000229	16.16124	49.7	31.01984	49.8	52.26832	49.8	96.60438	49.5	8.688335	49.6	16.03331	49.7	30.92789	49.8	51.97442	49.8	94.21521
49.59999847	8.857082818	49.70000076	16.17357	49.8	31.0364	49.9	52.30105	49.9	96.66806	49.6	8.694878	49.7	16.04415	49.8	30.94577	49.9	51.99569	49.9	94.23215
49.69999695	8.861329957	49.79999924	16.18549	49.9	31.05728	50	52.33292	50	96.73131	49.7	8.701413	49.8	16.05496	49.9	30.96361	50	52.01686	50	94.24893
49.79999542	8.868321128	49.89999771	16.1998	50	31.07409	50.1	52.36694	50.1	96.79489	49.8	8.70794	49.9	16.06576	50	30.98141	50.1	52.03794	50.1	94.26556
49.90000153	8.878547805	50.00000381	16.21386	50.1	31.09271	50.2	52.40167	50.2	96.85453	49.9	8.71446	50	16.07654	50.1	30.99917	50.2	52.05891	50.2	94.28203
50	8.886562677	50.10000229	16.22673	50.2	31.10873	50.3	52.43266	50.3	96.91792	50	8.720972	50.1	16.0873	50.2	31.01688	50.3	52.0798	50.3	94.29834
50.09999847	8.898271798	50.20000076	16.23735	50.3	31.1227	50.4	52.46422	50.4	96.97845	50.1	8.727476	50.2	16.09804	50.3	31.03455	50.4	52.10059	50.4	94.31449
50.19999695	8.904511257	50.29999924	16.25065	50.4	31.13737	50.5	52.49327	50.5	97.03749	50.2	8.733972	50.3	16.10876	50.4	31.05218	50.5	52.12128	50.5	94.33049
50.29999542	8.90616272	50.39999771	16.2608	50.5	31.15076	50.6	52.52974	50.6	97.09649	50.3	8.74046	50.4	16.11947	50.5	31.06977	50.6	52.14187	50.6	94.34634
50.40000153	8.911347254	50.50000381	16.2679	50.6	31.17095	50.7	52.56509	50.7	97.15632	50.4	8.746941	50.5	16.13016	50.6	31.08731	50.7	52.16237	50.7	94.36203
50.5	8.917260204	50.60000229	16.28262	50.7	31.18916	50.8	52.59754	50.8	97.21295	50.5	8.753414	50.6	16.14083	50.7	31.10481	50.8	52.18278	50.8	94.37758
50.59999847	8.925053168	50.70000076	16.29774	50.8	31.20954	50.9	52.62537	50.9	97.27435	50.6	8.759879	50.7	16.15148	50.8	31.12227	50.9	52.20309	50.9	94.39298
50.69999695	8.935040891	50.79999924	16.30861	50.9	31.22592	51	52.65235	51	97.33489	50.7	8.766336	50.8	16.16211	50.9	31.13969	51	52.22331	51	94.40822
50.79999542	8.940340783	50.89999771	16.31867	51	31.23963	51.1	52.68434	51.1	97.39881	50.8	8.772785	50.9	16.17272	51	31.15706	51.1	52.24343	51.1	94.42333
50.90000153	8.947029734	51.00000381	16.3275	51.1	31.25813	51.2	52.71199	51.2	97.45735	50.9	8.779228	51	16.18332	51.1	31.1744	51.2	52.26346	51.2	94.43828
51	8.950687795	51.10000229	16.33815	51.2	31.27448	51.3	52.73994	51.3	97.52129	51	8.785662	51.1	16.1939	51.2	31.19169	51.3	52.2834	51.3	94.45309
51.09999847	8.959903427	51.20000076	16.34649	51.3	31.29242	51.4	52.76288	51.4	97.583	51.1	8.792088	51.2	16.20446	51.3	31.20894	51.4	52.30324	51.4	94.46777
51.19999695	8.965668733	51.29999924	16.35685	51.4	31.3083	51.5	52.79404	51.5	97.643	51.2	8.798506	51.3	16.215	51.4	31.22615	51.5	52.323	51.5	94.48229
51.29999542	8.973029629	51.39999771	16.36942	51.5	31.3281	51.6	52.82514	51.6	97.70317	51.3	8.804917	51.4	16.22553	51.5	31.24331	51.6	52.34265	51.6	94.49668
51.40000153	8.979057443	51.50000381	16.38215	51.6	31.34727	51.7	52.85374	51.7	97.76128	51.4	8.811321	51.5	16.23603	51.6	31.26044	51.7	52.36222	51.7	94.51093
51.5	8.98317655	51.60000229	16.39578	51.7	31.36263	51.8	52.88236	51.8	97.82067	51.5	8.817716	51.6	16.24652	51.7	31.27752	51.8	52.3817	51.8	94.52504
51.59999847	8.988733248	51.70000076	16.40783	51.8	31.37757	51.9	52.91183	51.9	97.87798	51.6	8.824104	51.7	16.25699	51.8	31.29456	51.9	52.40109	51.9	94.53902
51.69999695	8.993381329	51.79999924	16.41978	51.9	31.39096	52	52.94403	52	97.93508	51.7	8.830484	51.8	16.26744	51.9	31.31156	52	52.42038	52	94.55285
51.79999542	9.000001532	51.89999771	16.43028	52	31.40583	52.1	52.96876	52.1	97.98744	51.8	8.836856	51.9	16.27788	52	31.32852	52.1	52.43958	52.1	94.56656
51.90000153	9.003918482	52.00000381	16.4437	52.1	31.41386	52.2	52.99655	52.2	98.04544	51.9	8.843222	52	16.28829	52.1	31.34544	52.2	52.4587	52.2	94.58013
52	9.011462122	52.10000229	16.44958	52.2	31.4257	52.3	53.02583	52.3	98.09954	52	8.849579	52.1	16.29869	52.2	31.36231	52.3	52.47772	52.3	94.59357
52.09999847	9.015653549	52.20000076	16.46159	52.3	31.44216	52.4	53.05561	52.4	98.15578	52.1	8.855928	52.2	16.30907	52.3	31.37915	52.4	52.49666	52.4	94.60688
52.19999695	9.017324399	52.29999924	16.4756	52.4	31.45898	52.5	53.08334	52.5	98.2087	52.2	8.86227	52.3	16.31943	52.4	31.39595	52.5	52.5155	52.5	94.62006
52.29999542	9.024237102	52.39999771	16.48575	52.5	31.46986	52.6	53.11014	52.6	98.26323	52.3	8.868604	52.4	16.32978	52.5	31.4127	52.6	52.53426	52.6	94.6331
52.40000153	9.029462513	52.50000381	16.49445	52.6	31.48163	52.7	53.13838	52.7	98.31707	52.4	8.874931	52.5	16.34011	52.6	31.42941	52.7	52.55292	52.7	94.64603
52.5	9.029311985	52.60000229	16.50265	52.7	31.49638	52.8	53.17073	52.8	98.37337	52.5	8.88125	52.6	16.35042	52.7	31.44608	52.8	52.5715	52.8	94.65882
52.59999847	9.033581807	52.70000076	16.51772	52.8	31.51146	52.9	53.20198	52.9	98.43529	52.6	8.887561	52.7	16.36071	52.8	31.46271	52.9	52.59	52.9	94.67149
52.69999695	9.041710504	52.79999924	16.53114	52.9	31.53012	53	53.22926	53	98.49713	52.7	8.893865	52.8	16.37098	52.9	31.47931	53	52.6084	53	94.68404
52.79999542	9.050895993	52.89999771	16.54159	53	31.54334	53.1	53.25373	53.1	98.55474	52.8	8.900161	52.9	16.38124	53	31.49586	53.1	52.62672	53.1	94.69646

52.90000153	9.058707469	53.00000381	16.55249	53.1	31.56175	53.2	53.28544	53.2	98.61547	52.9	8.90645	53	16.39148	53.1	31.51237	53.2	52.64495	53.2	94.70876
53	9.064055585	53.10000229	16.56078	53.2	31.58156	53.3	53.32109	53.3	98.67262	53	8.912731	53.1	16.4017	53.2	31.52883	53.3	52.66309	53.3	94.72094
53.09999847	9.067944717	53.20000076	16.57213	53.3	31.60026	53.4	53.35203	53.4	98.72938	53.1	8.919004	53.2	16.4119	53.3	31.54526	53.4	52.68115	53.4	94.73301
53.19999695	9.077068938	53.29999924	16.58189	53.4	31.6201	53.5	53.37968	53.5	98.79073	53.2	8.92527	53.3	16.42209	53.4	31.56165	53.5	52.69912	53.5	94.74495
53.29999542	9.085419201	53.39999771	16.59305	53.5	31.63754	53.6	53.40641	53.6	98.84666	53.3	8.931529	53.4	16.43226	53.5	31.578	53.6	52.71701	53.6	94.75677
53.40000153	9.096811389	53.50000381	16.60669	53.6	31.65203	53.7	53.43334	53.7	98.90204	53.4	8.93778	53.5	16.44241	53.6	31.59431	53.7	52.7348	53.7	94.76848
53.5	9.105872293	53.60000229	16.61481	53.7	31.66842	53.8	53.46039	53.8	98.95708	53.5	8.944023	53.6	16.45254	53.7	31.61058	53.8	52.75252	53.8	94.78007
53.59999847	9.110680819	53.70000076	16.62311	53.8	31.68736	53.9	53.48824	53.9	99.0144	53.6	8.950259	53.7	16.46266	53.8	31.62681	53.9	52.77015	53.9	94.79155
53.69999695	9.112669812	53.79999924	16.6335	53.9	31.70728	54	53.51426	54	99.07182	53.7	8.956487	53.8	16.47276	53.9	31.64299	54	52.7877	54	94.80291
53.79999542	9.119498484	53.89999771	16.64688	54	31.72127	54.1	53.54959	54.1	99.13375	53.8	8.962708	53.9	16.48284	54	31.65914	54.1	52.80516	54.1	94.81417
53.90000153	9.127189643	54.00000381	16.66056	54.1	31.74122	54.2	53.57791	54.2	99.19169	53.9	8.968921	54	16.49291	54.1	31.67525	54.2	52.82254	54.2	94.82531
54	9.134987446	54.10000229	16.67547	54.2	31.75704	54.3	53.60677	54.3	99.24901	54	8.975127	54.1	16.50295	54.2	31.69132	54.3	52.83983	54.3	94.83634
54.09999847	9.143594285	54.20000076	16.68251	54.3	31.77048	54.4	53.63178	54.4	99.3042	54.1	8.981325	54.2	16.51298	54.3	31.70735	54.4	52.85704	54.4	94.84726
54.19999695	9.150814558	54.29999924	16.6955	54.4	31.78398	54.5	53.65739	54.5	99.35511	54.2	8.987516	54.3	16.52299	54.4	31.72334	54.5	52.87417	54.5	94.85807
54.29999542	9.156802383	54.39999771	16.70504	54.5	31.79651	54.6	53.68519	54.6	99.40766	54.3	8.993699	54.4	16.53299	54.5	31.73929	54.6	52.89122	54.6	94.86877
54.40000153	9.161739578	54.50000381	16.71034	54.6	31.81495	54.7	53.71213	54.7	99.46145	54.4	8.999875	54.5	16.54297	54.6	31.7552	54.7	52.90818	54.7	94.87937
54.5	9.162375965	54.60000229	16.72306	54.7	31.82819	54.8	53.74233	54.8	99.51682	54.5	9.006044	54.6	16.55293	54.7	31.77107	54.8	52.92507	54.8	94.88986
54.59999847	9.169589622	54.70000076	16.73323	54.8	31.8446	54.9	53.77066	54.9	99.57021	54.6	9.012205	54.7	16.56287	54.8	31.7869	54.9	52.94187	54.9	94.90025
54.69999695	9.176237762	54.79999924	16.74382	54.9	31.86207	55	53.80278	55	99.62034	54.7	9.018358	54.8	16.57279	54.9	31.8027	55	52.95859	55	94.91054
54.79999542	9.186146941	54.89999771	16.75605	55	31.87673	55.1	53.84109	55.1	99.67887	54.8	9.024504	54.9	16.5827	55	31.81845	55.1	52.97523	55.1	94.92072
54.90000153	9.195145599	55.00000381	16.76677	55.1	31.89534	55.2	53.8725	55.2	99.73459	54.9	9.030643	55	16.59259	55.1	31.83417	55.2	52.99179	55.2	94.9308
55	9.199782096	55.10000229	16.78012	55.2	31.9113	55.3	53.90233	55.3	99.78624	55	9.036774	55.1	16.60247	55.2	31.84984	55.3	53.00826	55.3	94.94078
55.09999847	9.209221248	55.20000076	16.79278	55.3	31.92673	55.4	53.93156	55.4	99.8354	55.1	9.042898	55.2	16.61233	55.3	31.86548	55.4	53.02466	55.4	94.95066
55.19999695	9.214512457	55.29999924	16.80308	55.4	31.93969	55.5	53.96135	55.5	99.8865	55.2	9.049014	55.3	16.62217	55.4	31.88108	55.5	53.04098	55.5	94.96044
55.29999542	9.219012374	55.39999771	16.80739	55.5	31.95954	55.6	53.98942	55.6	99.93544	55.3	9.055123	55.4	16.63199	55.5	31.89664	55.6	53.05722	55.6	94.97013
55.40000153	9.215809094	55.50000381	16.82024	55.6	31.97336	55.7	54.01456	55.7	99.98477	55.4	9.061225	55.5	16.6418	55.6	31.91216	55.7	53.07338	55.7	94.97971
55.5	9.216580726	55.60000229	16.82911	55.7	31.98764	55.8	54.03892	55.8	100.0381	55.5	9.06732	55.6	16.65158	55.7	31.92764	55.8	53.08946	55.8	94.9892
55.59999847	9.21891602	55.70000076	16.84027	55.8	32.00091	55.9	54.0708	55.9	100.0967	55.6	9.073406	55.7	16.66136	55.8	31.94308	55.9	53.10547	55.9	94.9986
55.69999695	9.22033517	55.79999924	16.84781	55.9	32.01721	56	54.10281	56	100.1504	55.7	9.079486	55.8	16.67111	55.9	31.95849	56	53.12139	56	95.0079
55.79999542	9.226789722	55.89999771	16.86026	56	32.03094	56.1	54.13063	56.1	100.2026	55.8	9.085558	55.9	16.68085	56	31.97386	56.1	53.13724	56.1	95.0171
55.90000153	9.232065805	56.00000381	16.86944	56.1	32.04613	56.2	54.1529	56.2	100.2547	55.9	9.091623	56	16.69057	56.1	31.98918	56.2	53.15301	56.2	95.02622
56	9.234093378	56.10000229	16.87494	56.2	32.0561	56.3	54.17876	56.3	100.3112	56	9.097681	56.1	16.70028	56.2	32.00447	56.3	53.1687	56.3	95.03524
56.09999847	9.237161027	56.20000076	16.87737	56.3	32.07049	56.4	54.20493	56.4	100.3648	56.1	9.103731	56.2	16.70996	56.3	32.01973	56.4	53.18432	56.4	95.04417
56.19999695	9.237808192	56.29999924	16.88384	56.4	32.08601	56.5	54.2293	56.5	100.4148	56.2	9.109774	56.3	16.71963	56.4	32.03494	56.5	53.19986	56.5	95.05302
56.29999542	9.24585609	56.39999771	16.89027	56.5	32.09981	56.6	54.25816	56.6	100.4639	56.3	9.115809	56.4	16.72929	56.5	32.05012	56.6	53.21532	56.6	95.06177
56.40000153	9.254267645	56.50000381	16.89926	56.6	32.11504	56.7	54.28448	56.7	100.5156	56.4	9.121838	56.5	16.73892	56.6	32.06525	56.7	53.23071	56.7	95.07043
56.5	9.25723964	56.60000229	16.90992	56.7	32.12674	56.8	54.31119	56.8	100.5649	56.5	9.127859	56.6	16.74855	56.7	32.08035	56.8	53.24602	56.8	95.07901
56.59999847	9.262444488	56.70000076	16.91927	56.8	32.13411	56.9	54.33482	56.9	100.6104	56.6	9.133872	56.7	16.75815	56.8	32.09542	56.9	53.26126	56.9	95.0875
56.69999695	9.267894745	56.79999924	16.92644	56.9	32.1438	57	54.36562	57	100.6571	56.7	9.139878	56.8	16.76774	56.9	32.11044	57	53.27642	57	95.09591
56.79999542	9.274196573	56.89999771	16.92763	57	32.15891	57.1	54.39241	57.1	100.7061	56.8	9.145877	56.9	16.77731	57	32.12543	57.1	53.2915	57.1	95.10423
56.90000153	9.276265214	57.00000381	16.93704	57.1	32.17589	57.2	54.41742	57.2	100.7628	56.9	9.15187	57	16.78686	57.1	32.14038	57.2	53.30651	57.2	95.11246
57	9.283219792	57.10000229	16.94703	57.2	32.19692	57.3	54.44124	57.3	100.8122	57	9.157854	57.1	16.7964	57.2	32.15529	57.3	53.32145	57.3	95.12061
57.09999847	9.286615183	57.20000076	16.9565	57.3	32.21365	57.4	54.46995	57.4	100.8636	57.1	9.163831	57.2	16.80592	57.3	32.17016	57.4	53.33632	57.4	95.12868
57.19999695	9.298022344	57.29999924	16.96409	57.4	32.23372	57.5	54.49554	57.5	100.9098	57.2	9.169801	57.3	16.81542	57.4	32.185	57.5	53.35111	57.5	95.13667
57.29999542	9.297420126	57.39999771	16.97656	57.5	32.25565	57.6	54.52224	57.6	100.9674	57.3	9.175764	57.4	16.82491	57.5	32.1998	57.6	53.36583	57.6	95.14458
57.40000153	9.304202595	57.50000381	16.98753	57.6	32.27446	57.7	54.55268	57.7	101.017	57.4	9.18172	57.5	16.83438	57.6	32.21456	57.7	53.38047	57.7	95.1524
57.5	9.312552926	57.60000229	16.99776	57.7	32.28099	57.8	54.5901	57.8	101.0778	57.5	9.187668	57.6	16.84383	57.7	32.22928	57.8	53.39504	57.8	95.16015
57.59999847	9.321283604	57.70000076	17.00954	57.8	32.29962	57.9	54.62026	57.9	101.1325	57.6	9.193609	57.7	16.85327	57.8	32.24397	57.9	53.40954	57.9	95.16782
57.69999695	9.325690738	57.79999924	17.02265	57.9	32.30781	58	54.64734	58	101.1856	57.7	9.199543	57.8	16.86269	57.9	32.25862	58	53.42397	58	95.17541
57.79999542	9.334836504	57.89999771	17.02802	58	32.31458	58.1	54.67765	58.1	101.2463	57.8	9.20547	57.9	16.87209	58	32.27324	58.1	53.43833	58.1	95.18292
57.90000153	9.336415915	58.00000381	17.03835	58.1	32.32673	58.2	54.69622	58.2	101.2837	57.9	9.21139	58	16.88148	58.1	32.28781	58.2	53.45261	58.2	95.19036
58	9.342989138	58.10000229	17.0497	58.2	32.33834	58.3	54.72018	58.3	101.3344	58	9.217302	58.1	16.89085	58.2	32.30235	58.3	53.46682	58.3	95.19772
58.09999847	9.352630481	58.20000076	17.05098	58.3	32.35018	58.4	54.74959	58.4	101.3956	58.1	9.223207	58.2	16.90021	58.3	32.31686	58.4	53.48097	58.4	95.205
58.19999695	9.344198607	58.29999924	17.05959	58.4	32.36499	58.5	54.77705	58.5	101.4576	58.2	9.229105	58.3	16.90955	58.4	32.33132	58.5	53.49504	58.5	95.21222
58.29999542	9.345778867	58.39999771	17.07105	58.5	32.38683	58.6	54.7913	58.6	101.5038	58.3	9.234996	58.4	16.91887	58.5	32.34575	58.6	53.50904	58.6	95.21935
58.40000153	9.353585011	58.50000381	17.0798	58.6	32.39029	58.7	54.81119	58.7	101.5525	58.4	9.24088	58.5	16.92818	58.6	32.36015	58.7	53.52297	58.7	95.22642
58.5	9.355284341	58.60000229	17.08578	58.7	32.40196	58.8	54.82147	58.8	101.6055	58.5	9.246757	58.6	16.93747	58.7	32.3745	58.8	53.53683	58.8	95.23341
58.59999847	9.355284341	58.70000076	17.08739	58.8	32.40479	58.9	54.8466	58.9	101.6528	58.6	9.252626	58.7	16.94674	58.8	32.38882	58.9	53.55063	58.9	95.24033
58.69999695	9.357895874	58.79999924	17.09777	58.9	32.41411	59	54.8466	59	101.6106	58.7	9.258488	58.8	16.956	58.9	32.40311	59	53.56435	59	95.24718
58.79999542	9.358561273	58.89999771	17.11635	59	32.41411	59.1	54.82707	59.1	101.6081	58.8	9.264343	58.9	16.96524	59	32.41735	59.1	53.57801	59.1	95.25396
58.90000153	9.36380364	59.00000381	17.11635	59.1	32.41088	59.2	54.82468	59.2	101.6055	58.9	9.270192	59	16.97446	59.1	32.43157	59.2	53.59159	59.2	95.26067
59	9.36380364	59.10000229	17.10328	59.2	32.41031	59.3	54.82707	59.3	101.5576	59	9.276033	59.1	16.98367	59.2	32.44574	59.3	53.60511	59.3	95.26732
59.09999847	9.36380364	59.20000076	17.10098	59.3	32.41088	59.4	54.82707	59.4	101.5564	59.1	9.281867	59.2	16.99287	59.3	32.45988	59.4	53.61856	59.4	95.27389
59.19999695	9.36408257	59.29999924	17.10328	59.4	32.41088	59.5	54.82468	59.5	101.5525	59.2	9.287693	59.3	17.00204	59.4	32.47398	59.5	53.63194	59.5	95.2804
59.29999542	9.365728632	59.39999771	17.10328	59.5	32.41031	59.6	54.82147	59.6	101.5156	59.3	9.293513	59.4	17.0112	59.5	32.48805	59.6	53.64526	59.6	95.28684
59.40000153	9.365728632	59.50000381	17.10098	59.6	32.40924	59.7	54.81871	59.7	101.5147	59.4	9.299326	59.5	17.02035	59.6	32.50208	59.7	53.65851	59.7	95.29321
59.5	9.36408257	59.60000229	17.09939	59.7	32.40899	59.8	54.81822	59.8	101.5102	59.5	9.305131	59.6	17.02948	59.7	32.51608	59.8	53.67169	59.8	95.29952
59.59999847	9.36380364	59.70000076	17.09777	59.8	32.40895	59.9	54.81119	59.9	101.5061	59.6	9.31093	59.7	17.03859	59.8	32.53004	59.9	53.68481	59.9	95.30576
59.69999695	9.36373203	59.79999924	17.09648	59.9	32.40479	60	54.80757	60	101.5038	59.7	9.316721	59.8	17.04768	59.9	32.54396	60	53.69785	60	95.31194
59.79999542	9.363466857	59.89999771	17.09605	60	32.40196	60.1	54.80757	60.1	101.5038	59.8	9.322505	59.9	17.05676	60	32.55785	60.1	29.18785	60.1	50.88194
59.90000153	9.358561273	60.00000381	17.08739	60.1	32.40196	60.2	54.80757	60.2	101.1945	59.9	9.328283	60	17.06583	60.1	17.04785	60.2	29.10883	60.2	50.70453
60	9.357895874	60.10000229	17.08739	60.2	32.40196	60.3	53.0007	60.3	98.5442	60	9.334053	60.1	9.172829	60.2	17.01565	60.3	29.03021	60.3	50.52892
60.09999847	9.357895874	60.20000076	17.08739	60.3	31.9789	60.4	42.6901	60.4	86.2643	60.1	5.013053	60.2	9.158837	60.3	16.98352	60.4	28.95197	60.4	50.35506
60.19999695	9.357895874	60.29999924	16.4921	60.4	23.014	60.5	36.4171	60.5	78.546	60.2	5.007312	60.3	9.14487	60.4	16.95147	60.5	28.87413	60.5	50.18292
60.29999542	9.277678829	60.39999771	10.9469	60.5	20.5518	60.6	32.3142	60.6	72.5875	60.3	5.001577	60.4	9.130927	60.5	16.91951	60.6	28.79667	60.6	50.01247
60.40000153	6.378199946	60.50000381	10.9469	60.6	20.5518	60.7	31.88158	60.7	69.0086	60.4	4.995849	60.5	9.117009	60.6	16.88763	60.7	28.7196	60.7	49.84366
60.5	6.378199946	60.60000229	10.9469	60.7	20.5518	60.8	31.5953	60.8	66.4923	60.5	4.990129	60.6	9.103115	60.7	16.85583	60.8	28.64291	60.8	49.67645
60.59999847	6.378199946	60.70000076	10.9469	60.8	20.5518	60.9	31.5953	60.9	64.4714	60.6	4.984415	60.7	9.089246	60.8	16.82411	60.9	28.56658	60.9	49.5108
60.69999695	6.378199946	60.79999924	10.9469	60.9	20.5518	61	31.5953	61	62.6898	60.7	4.978708	60.8	9.075401	60.9	16.79247	61	28.49063	61	49.34671
60.79999542	6.378199946	60.89999771	10.9469	61	20.5518	61.1	31.5953	61.1	61.3828	60.8	4.973009	60.9	9.061579	61	16.76091	61.1	28.41506	61.1	49.18411
60.90000153	6.378199946	61.00000381	10.9469	61.1	20.5518	61.2	31.5953	61.2	60.4032	60.9	4.967315	61	9.047783	61.1	16.72943	61.2	28.33984	61.2	49.023
61	6.378199946	61.10000229	10.9469	61.2	20.5518	61.3	31.5448	61.3	59.5335	61	4.961629	61.1	9.03401	61.2	16.69803	61.3	28.26499	61.3	48.86333
61.09999847	6.378199946	61.20000076	10.9469	61.3	20.5518	61.4	31.5448	61.4	58.8811	61.1	4.95595	61.2	9.020262	61.3	16.66671	61.4	28.19049	61.4	48.70506
61.19999695	6.378199946	61.29999924	10.9469	61.4	20.5518	61.5	31.5448	61.5	58.6648	61.2	4.950278	61.3	9.006538	61.4	16.63547	61.5	28.11635	61.5	48.54819
61.29999542	6.378199946	61.39999771	10.9469	61.5	20.5518	61.6	31.5	61.6	58.3051	61.3	4.944613	61.4	8.992837	61.5	16.6043	61.6	28.04256	61.6	48.39268
61.40000153	6.378199946	61.50000381	10.9469	61.6	20.5518	61.7	31.5	61.7	58.00288	61.4	4.938954	61.5	8.979161	61.6	16.57322	61.7	27.96912	61.7	48.2385
61.5	6.378199946	61.60000229	10.9469	61.7	20.5169	61.8	31.46622	61.8	57.81092	61.5	4.933303	61.6	8.965509	61.7	16.54221	61.8	27.89603	61.8	48.08564
61.59999847	6.378199946	61.70000076	10.9469	61.8	20.1015	61.9	31.20628	61.9	57.23188	61.6	4.927658	61.7	8.95188	61.8	16.51128	61.9	27.82327	61.9	47.93404
61.69999695	6.378199946	61.79999924	10.836	61.9	19.9646	62	30.70118	62	56.13828	61.7	4.922021	61.8	8.938276	61.9	16.48043	62	27.75086	62	47.78372
61.79999542	6.241276037	61.89999771	10.81	62	19.7868	62.1	30.12672	62.1	55.37992	61.8	4.91639	61.9	8.924694	62	16.44965	62.1	27.67879	62.1	47.63463
61.90000153	6.163684119	62.00000381	10.58375	62.1	19.4712	62.2	29.71379	62.2	54.59269	61.9	4.910765	62	8.911137	62.1	16.41895	62.2	27.60705	62.2	47.48675
62	5.974776774	62.10000229	10.5174	62.2	19.1674	62.3	29.34188	62.3	53.92098	62	4.905148	62.1	8.897603	62.2	16.38833	62.3	27.53564	62.3	47.34007
62.09999847	5.760723206	62.20000076	10.3056	62.3	18.8967	62.4	28.9787	62.4	53.1201	62.1	4.899538	62.2	8.884093	62.3	16.35778	62.4	27.46455	62.4	47.19455
62.19999695	5.708414199	62.29999924	9.9926	62.4	18.5775	62.5	28.89549	62.5	52.74279	62.2	4.893934	62.3	8.870607	62.4	16.32731	62.5	27.39379	62.5	47.05019
62.29999542	5.647217469	62.39999771	9.914186	62.5	18.5655	62.6	28.70431	62.6	52.11051	62.3	4.888337	62.4	8.857143	62.5	16.29692	62.6	27.32336	62.6	46.90696
62.40000153	5.520998717	62.50000381	9.914186	62.6	18.5659	62.7	28.39159	62.7	51.41189	62.4	4.882747	62.5	8.843704	62.6	16.2666	62.7	27.25324	62.7	46.76484
62.5	5.488292933	62.60000229	9.8689	62.7	18.3396	62.8	27.9917	62.8	50.9118	62.5	4.877164	62.6	8.830288	62.7	16.23635	62.8	27.18344	62.8	46.62381
62.59999847	5.419507004	62.70000076	9.7741	62.8	18.1254	62.9	27.8125	62.9	50.6363	62.6	4.871587	62.7	8.816895	62.8	16.20618	62.9	27.11395	62.9	46.48384
62.69999695	5.399906586	62.79999924	9.6498	62.9	18.0747	63	27.576	63	50.3749	62.7	4.866018	62.8	8.803526	62.9	16.17609	63	27.04478	63	46.34494
62.79999542	5.455505827	62.89999771	9.6259	63	17.8677	63.1	27.3508	63.1	49.8402	62.8	4.860455	62.9	8.790178	63	16.14606	63.1	26.97591	63.1	46.20708
62.90000153	5.473597676	63.00000381	9.439497	63.1	17.768	63.2	27.1872	63.2	49.3552	62.9	4.854898	63	8.776855	63.1	16.11612	63.2	26.90736	63.2	46.07024
63	5.397098537	63.10000229	9.4008	63.2	17.6947	63.3	26.9305	63.3	48.9306	63	4.849349	63.1	8.763555	63.2	16.08624	63.3	26.8391	63.3	45.9344
63.09999847	5.337701477	63.20000076	9.3778	63.3	17.5344	63.4	26.7595	63.4	48.4309	63.1	4.843806	63.2	8.750278	63.3	16.05644	63.4	26.77115	63.4	45.79954
63.19999695	5.324400731	63.29999924	9.3048	63.4	17.3308	63.5	26.5593	63.5	48.1052	63.2	4.83827	63.3	8.737025	63.4	16.02672	63.5	26.70349	63.5	45.66567
63.29999542	5.237103204	63.39999771	9.1849	63.5	17.2206	63.6	26.3685	63.6	47.764	63.3	4.832741	63.4	8.723793	63.5	15.99706	63.6	26.63614	63.6	45.53275
63.40000153	5.175901342	63.50000381	9.186499	63.6	17.0785	63.7	26.2697	63.7	47.4781	63.4	4.827218	63.5	8.710585	63.6	15.96748	63.7	26.56907	63.7	45.40078
63.5	5.242599728	63.60000229	9.1042	63.7	17.0376	63.8	26.1522	63.8	47.1356	63.5	4.821702	63.6	8.6974	63.7	15.93797	63.8	26.5023	63.8	45.26973
63.59999847	5.205100325	63.70000076	9.1322	63.8	16.9779	63.9	26.0154	63.9	46.8115	63.6	4.816193	63.7	8.684238	63.8	15.90853	63.9	26.43582	63.9	45.13959
63.69999695	5.223198712	63.79999924	9.1192	63.9	16.8744	64	25.8876	64	46.5064	63.7	4.81069	63.8	8.671098	63.9	15.87917	64	26.36962	64	45.01036
63.79999542	5.222798435	63.89999771	9.0412	64	16.772	64.1	25.7055	64.1	46.1748	63.8	4.805194	63.9	8.65798	64	15.84987	64.1	26.30371	64.1	44.88201
63.90000153	5.165800286	64	8.9908	64.10001	16.6781	64.2	25.5352	64.2	45.9007	63.9	4.799705	64	8.644886	64.1	15.82065	64.2	26.23809	64.2	44.75455
64	5.145100394	64.1000061	8.9453	64.2	16.5912	64.3	25.3683	64.3	45.6044	64	4.794222	64.1	8.631815	64.2	15.7915	64.3	26.17274	64.3	44.62794
64.09999847	5.123799154	64.19999695	8.8872	64.3	16.4784	64.4	25.2438	64.4	45.3471	64.1	4.788746	64.2	8.618766	64.3	15.76242	64.4	26.10767	64.4	44.50217
64.19999695	5.089000033	64.30000305	8.8043	64.39999	16.4041	64.5	25.0904	64.5	45.0917	64.2	4.783277	64.3	8.60574	64.4	15.73341	64.5	26.04288	64.5	44.37724
64.29999542	5.034601137	64.3999939	8.7424	64.5	16.3445	64.6	24.9454	64.6	44.8116	64.3	4.777814	64.4	8.592735	64.5	15.70447	64.6	25.97836	64.6	44.25314

64.40000153	5.017400849	64.5	8.6998	64.60001	16.2544	64.7	24.8049	64.7	44.5597	64.4	4.772358	64.5	8.579754	64.6	15.6756	64.7	25.91411	64.7	44.12986
64.5	5.018000048	64.6000061	8.6699	64.7	16.1841	64.8	24.6486	64.8	44.295	64.5	4.766908	64.6	8.566795	64.7	15.6468	64.8	25.85013	64.8	44.00738
64.59999847	4.980798915	64.69999695	8.6254	64.8	16.087	64.9	24.5306	64.9	44.0498	64.6	4.761466	64.7	8.553858	64.8	15.61807	64.9	25.78642	64.9	43.88567
64.69999695	4.983099994	64.80000305	8.5794	64.89999	16.0157	65	24.4063	65	43.801	64.7	4.756029	64.8	8.540944	64.9	15.5894	65	25.72298	65	43.76476
64.79999542	4.951400189	64.8999939	8.5367	65	15.9331	65.1	24.2839	65.1	43.5634	64.8	4.7506	64.9	8.52805	65	15.56081	65.1	25.6598	65.1	43.64461
64.90000153	4.921400723	65	8.4857	65.10001	15.8788	65.2	24.1572	65.2	43.3187	64.9	4.745176	65	8.51518	65.1	15.53229	65.2	25.59688	65.2	43.52523
65	4.906299949	65.1000061	8.45	65.2	15.806	65.3	24.0583	65.3	43.0812	65	4.73976	65.1	8.502333	65.2	15.50383	65.3	25.53422	65.3	43.40659
65.09999847	4.875698667	65.19999695	8.4113	65.3	15.724	65.4	23.9465	65.4	42.8506	65.1	4.73435	65.2	8.489507	65.3	15.47544	65.4	25.47182	65.4	43.28868
65.19999695	4.858300001	65.30000305	8.37	65.39999	15.665	65.5	23.8287	65.5	42.6275	65.2	4.728946	65.3	8.476703	65.4	15.44712	65.5	25.40967	65.5	43.17152
65.29999542	4.843699006	65.3999939	8.3366	65.5	15.5961	65.6	23.7088	65.6	42.3943	65.3	4.723549	65.4	8.463921	65.5	15.41887	65.6	25.34778	65.6	43.05507
65.40000153	4.828800813	65.5	8.288	65.60001	15.5201	65.7	23.5827	65.7	42.1781	65.4	4.718158	65.5	8.451161	65.6	15.39069	65.7	25.28614	65.7	42.93933
65.5	4.806600015	65.6000061	8.2448	65.7	15.4671	65.8	23.4724	65.8	41.9749	65.5	4.712774	65.6	8.438423	65.7	15.36257	65.8	25.22476	65.8	42.82429
65.59999847	4.800699959	65.69999695	8.2118	65.8	15.4081	65.9	23.3728	65.9	41.7653	65.6	4.707397	65.7	8.425708	65.8	15.33452	65.9	25.16361	65.9	42.70993
65.69999695	4.764499997	65.80000305	8.187	65.89999	15.3514	66	23.2578	66	41.5449	65.7	4.702026	65.8	8.413014	65.9	15.30654	66	25.10272	66	42.59627
65.79999542	4.749498876	65.8999939	8.1824	66	15.2647	66.1	23.1525	66.1	41.3465	65.8	4.696662	65.9	8.40034	66	15.27862	66.1	25.04207	66.1	42.48328
65.90000153	4.737400004	66	8.1333	66.10001	15.1991	66.2	23.0377	66.2	41.1476	65.9	4.691303	66	8.38769	66.1	15.25077	66.2	24.98166	66.2	42.37096
66	4.705000018	66.1000061	8.109	66.2	15.1346	66.3	22.928	66.3	40.9608	66	4.685952	66.1	8.375061	66.2	15.22299	66.3	24.9215	66.3	42.2593
66.09999847	4.684698914	66.19999695	8.0645	66.3	15.0702	66.4	22.8359	66.4	40.7727	66.1	4.680607	66.2	8.362454	66.3	15.19527	66.4	24.86157	66.4	42.14828
66.19999695	4.653399997	66.30000305	8.0361	66.39999	15.0104	66.5	22.7343	66.5	40.586	66.2	4.675268	66.3	8.349868	66.4	15.16762	66.5	24.80188	66.5	42.03791
66.29999542	4.640699114	66.3999939	8.012	66.5	14.9551	66.6	22.645	66.6	40.423	66.3	4.669936	66.4	8.337303	66.5	15.14004	66.6	24.74243	66.6	41.92817
66.40000153	4.631900888	66.5	7.9833	66.60001	14.8952	66.7	22.5565	66.7	40.2524	66.4	4.66461	66.5	8.32476	66.6	15.11251	66.7	24.68322	66.7	41.81906
66.5	4.616800054	66.6000061	7.9641	66.7	14.8405	66.8	22.4642	66.8	40.0808	66.5	4.659291	66.6	8.312239	66.7	15.08506	66.8	24.62423	66.8	41.71057
66.59999847	4.612098469	66.69999695	7.9318	66.8	14.785	66.9	22.3874	66.9	39.9173	66.6	4.653978	66.7	8.299739	66.8	15.05767	66.9	24.56548	66.9	41.60269
66.69999695	4.599200007	66.80000305	7.9096	66.89999	14.7234	67	22.341	67	39.7634	66.7	4.648671	66.8	8.287261	66.9	15.03034	67	24.50696	67	41.49541
66.79999542	4.575999049	66.8999939	7.8902	67	14.6769	67.1	22.2504	67.1	39.5933	66.8	4.643371	66.9	8.274803	67	15.00308	67.1	24.44866	67.1	41.38874
66.90000153	4.565901563	67	7.8784	67.10001	14.6114	67.2	22.176	67.2	39.4373	66.9	4.638077	67	8.262367	67.1	14.97588	67.2	24.39059	67.2	41.28266
67	4.563500011	67.1000061	7.844	67.2	14.5541	67.3	22.0958	67.3	39.2645	67	4.63279	67.1	8.249952	67.2	14.94875	67.3	24.33275	67.3	41.17716
67.09999847	4.545599883	67.19999695	7.8217	67.3	14.4896	67.4	22.0229	67.4	39.0965	67.1	4.627509	67.2	8.237559	67.3	14.92168	67.4	24.27513	67.4	41.07223
67.19999695	4.537499971	67.30000305	7.799	67.39999	14.4422	67.5	21.9496	67.5	38.9401	67.2	4.622234	67.3	8.225186	67.4	14.89467	67.5	24.21773	67.5	40.96788
67.29999542	4.521099461	67.3999939	7.7804	67.5	14.3921	67.6	21.8745	67.6	38.7843	67.3	4.616966	67.4	8.212834	67.5	14.86772	67.6	24.16055	67.6	40.8641
67.40000153	4.512600881	67.5	7.7692	67.60001	14.3401	67.7	21.7879	67.7	38.6239	67.4	4.611704	67.5	8.200504	67.6	14.84084	67.7	24.1036	67.7	40.76087
67.5	4.500399998	67.6000061	7.744	67.7	14.2853	67.8	21.7083	67.8	38.472	67.5	4.606449	67.6	8.188195	67.7	14.81403	67.8	24.04686	67.8	40.6582
67.59999847	4.492499405	67.69999695	7.7105	67.8	14.2351	67.9	21.6251	67.9	38.3232	67.6	4.601199	67.7	8.175907	67.8	14.78727	67.9	23.99033	67.9	40.55607
67.69999695	4.471000023	67.80000305	7.6858	67.89999	14.1823	68	21.5397	68	38.1757	67.7	4.595957	67.8	8.163639	67.9	14.76058	68	23.93402	68	40.45448
67.79999542	4.465099173	67.8999939	7.6513	68	14.1242	68.1	21.4541	68.1	38.0254	67.8	4.59072	67.9	8.151392	68	14.73395	68.1	23.87792	68.1	40.35343
67.90000153	4.447200431	68	7.6181	68.10001	14.0683	68.2	21.3736	68.2	37.8797	67.9	4.58549	68	8.139166	68.1	14.70738	68.2	23.82204	68.2	40.25291
68	4.427800052	68.1000061	7.586	68.2	14.0173	68.3	21.293	68.3	37.7322	68	4.580266	68.1	8.126961	68.2	14.68087	68.3	23.76636	68.3	40.15291
68.09999847	4.41269966	68.19999695	7.5585	68.3	13.9654	68.4	21.2241	68.4	37.5891	68.1	4.575048	68.2	8.114777	68.3	14.65443	68.4	23.71089	68.4	40.05342
68.19999695	4.399900056	68.30000305	7.5367	68.39999	13.9144	68.5	21.1564	68.5	37.4518	68.2	4.569837	68.3	8.102614	68.4	14.62804	68.5	23.65563	68.5	39.95445
68.29999542	4.380199535	68.3999939	7.5174	68.5	13.8736	68.6	21.0851	68.6	37.323	68.3	4.564632	68.4	8.09047	68.5	14.60172	68.6	23.60058	68.6	39.85599
68.40000153	4.366200804	68.5	7.4988	68.60001	13.8232	68.7	21.014	68.7	37.2121	68.4	4.559433	68.5	8.078348	68.6	14.57546	68.7	23.54573	68.7	39.75803
68.5	4.355799973	68.6000061	7.4814	68.7	13.7748	68.8	20.9435	68.8	37.0906	68.5	4.55424	68.6	8.066246	68.7	14.54926	68.8	23.49108	68.8	39.66057
68.59999847	4.338199338	68.69999695	7.4606	68.8	13.7294	68.9	20.8724	68.9	36.9709	68.6	4.549054	68.7	8.054165	68.8	14.52312	68.9	23.43664	68.9	39.56359
68.69999695	4.318999966	68.80000305	7.4422	68.89999	13.6814	69	20.807	69	36.8507	68.7	4.543874	68.8	8.042105	68.9	14.49703	69	23.3824	69	39.46711
68.79999542	4.302299205	68.8999939	7.4186	69	13.6466	69.1	20.7356	69.1	36.7333	68.8	4.5387	68.9	8.030064	69	14.47101	69.1	23.32835	69.1	39.3711
68.90000153	4.287300821	69	7.3983	69.10001	13.6085	69.2	20.6611	69.2	36.6143	68.9	4.533532	69	8.018044	69.1	14.44505	69.2	23.27451	69.2	39.27557
69	4.275799988	69.1000061	7.3818	69.2	13.5648	69.3	20.5883	69.3	36.4889	69	4.528371	69.1	8.006044	69.2	14.41915	69.3	23.22086	69.3	39.18052
69.09999847	4.259899137	69.19999695	7.3596	69.3	13.5182	69.4	20.5235	69.4	36.3643	69.1	4.523215	69.2	7.994065	69.3	14.39331	69.4	23.16741	69.4	39.08592
69.19999695	4.243599955	69.30000305	7.3327	69.39999	13.4739	69.5	20.4604	69.5	36.2476	69.2	4.518066	69.3	7.982106	69.4	14.36753	69.5	23.11415	69.5	38.9918
69.29999542	4.229199556	69.3999939	7.3055	69.5	13.4289	69.6	20.3909	69.6	36.1286	69.3	4.512924	69.4	7.970167	69.5	14.34181	69.6	23.06109	69.6	38.89813
69.40000153	4.212700466	69.5	7.2814	69.60001	13.3907	69.7	20.3282	69.7	36.0112	69.4	4.507787	69.5	7.958248	69.6	14.31614	69.7	23.00822	69.7	38.80492
69.5	4.197099981	69.6000061	7.261	69.7	13.3555	69.8	20.2613	69.8	35.8962	69.5	4.502656	69.6	7.94635	69.7	14.29054	69.8	22.95554	69.8	38.71216
69.59999847	4.188699098	69.69999695	7.2388	69.8	13.3097	69.9	20.2047	69.9	35.7808	69.6	4.497532	69.7	7.934472	69.8	14.26499	69.9	22.90304	69.9	38.61983
69.69999695	4.17999997	69.80000305	7.2152	69.89999	13.268	70	20.1517	70	35.6664	69.7	4.492414	69.8	7.922614	69.9	14.2395	70	22.85074	70	38.52795
69.79999542	4.165399446	69.8999939	7.1959	70	13.2308	70.1	20.0855	70.1	35.5462	69.8	4.487302	69.9	7.910775	70	14.21407	70.1	22.79862	70.1	38.43651
69.90000153	4.152700717	70	7.1752	70.10001	13.1895	70.2	20.0152	70.2	35.4224	69.9	4.482196	70	7.898956	70.1	14.1887	70.2	22.7467	70.2	38.3455
70	4.143499959	70.1000061	7.152	70.2	13.1481	70.3	19.954	70.3	35.3021	70	4.477096	70.1	7.887158	70.2	14.16339	70.3	22.69495	70.3	38.25492
70.09999847	4.130399614	70.19999695	7.1269	70.3	13.1195	70.4	19.8956	70.4	35.1874	70.1	4.472003	70.2	7.87538	70.3	14.13813	70.4	22.64339	70.4	38.16476
70.19999695	4.113800022	70.30000305	7.1064	70.39999	13.0824	70.5	19.8392	70.5	35.0728	70.2	4.466915	70.3	7.863621	70.4	14.11293	70.5	22.59201	70.5	38.07502
70.29999542	4.100699465	70.3999939	7.088	70.5	13.0488	70.6	19.7913	70.6	34.9572	70.3	4.461834	70.4	7.851882	70.5	14.08779	70.6	22.54082	70.6	37.9857
70.40000153	4.088500743	70.5	7.0677	70.60001	13.0112	70.7	19.735	70.7	34.8426	70.4	4.456758	70.5	7.840163	70.6	14.06271	70.7	22.4898	70.7	37.89679
70.5	4.076800054	70.6000061	7.0447	70.7	12.9733	70.8	19.67	70.8	34.7316	70.5	4.451689	70.6	7.828464	70.7	14.03768	70.8	22.43897	70.8	37.80829
70.59999847	4.066299369	70.69999695	7.0206	70.8	12.9296	70.9	19.6119	70.9	34.6278	70.6	4.446626	70.7	7.816784	70.8	14.01271	70.9	22.3883	70.9	37.72019
70.69999695	4.05209999	70.80000305	6.9999	70.89999	12.8893	71	19.5495	71	34.5227	70.7	4.441569	70.8	7.805124	70.9	13.98779	71	22.33783	71	37.6325
70.79999542	4.034699664	70.8999939	6.9821	71	12.8523	71.1	19.4965	71.1	34.4205	70.8	4.436518	70.9	7.793483	71	13.96294	71.1	22.28752	71.1	37.54521
70.90000153	4.019500541	71	6.9615	71.10001	12.8226	71.2	19.4387	71.2	34.3198	70.9	4.431473	71	7.781862	71.1	13.93813	71.2	22.23739	71.2	37.45831
71	4.00750005	71.1000061	6.9453	71.2	12.7898	71.3	19.3842	71.3	34.2102	71	4.426434	71.1	7.770261	71.2	13.91339	71.3	22.18744	71.3	37.3718
71.09999847	4.000799147	71.19999695	6.9343	71.3	12.7558	71.4	19.3238	71.4	34.1027	71.1	4.421401	71.2	7.758679	71.3	13.8887	71.4	22.13766	71.4	37.28567
71.19999695	3.986000007	71.30000305	6.9217	71.39999	12.7162	71.5	19.2671	71.5	34.0011	71.2	4.416375	71.3	7.747117	71.4	13.86406	71.5	22.08805	71.5	37.19993
71.29999542	3.974799324	71.3999939	6.9023	71.5	12.6788	71.6	19.2135	71.6	33.8945	71.3	4.411354	71.4	7.735573	71.5	13.83948	71.6	22.03861	71.6	37.11458
71.40000153	3.962500492	71.5	6.883	71.60001	12.644	71.7	19.1591	71.7	33.7918	71.4	4.406339	71.5	7.72405	71.6	13.81496	71.7	21.98934	71.7	37.0296
71.5	3.953500021	71.6000061	6.8668	71.7	12.6092	71.8	19.1101	71.8	33.6964	71.5	4.40133	71.6	7.712546	71.7	13.79049	71.8	21.94024	71.8	36.94499
71.59999847	3.942699513	71.69999695	6.8503	71.8	12.5774	71.9	19.0524	71.9	33.5954	71.6	4.396328	71.7	7.701061	71.8	13.76608	71.9	21.89131	71.9	36.86075
71.69999695	3.92999996	71.80000305	6.8352	71.89999	12.5404	72	18.9984	72	33.4937	71.7	4.391331	71.8	7.689595	71.9	13.74172	72	21.84255	72	36.77689
71.79999542	3.925299166	71.8999939	6.8167	72	12.5016	72.1	18.947	72.1	33.3928	71.8	4.386341	71.9	7.678148	72	13.71741	72.1	21.79395	72.1	36.69338
71.90000153	3.915300439	72	6.7963	72.10001	12.472	72.2	18.8936	72.2	33.2945	71.9	4.381356	72	7.66672	72.1	13.69316	72.2	21.74552	72.2	36.61024
72	3.903299951	72.1000061	6.7789	72.2	12.435	72.3	18.8432	72.3	33.1982	72	4.376377	72.1	7.655312	72.2	13.66897	72.3	21.69725	72.3	36.52746
72.09999847	3.896399421	72.19999695	6.76	72.3	12.4023	72.4	18.7965	72.4	33.1056	72.1	4.371404	72.2	7.643923	72.3	13.64482	72.4	21.64915	72.4	36.44503
72.19999695	3.883100059	72.30000305	6.7466	72.39999	12.3718	72.5	18.7454	72.5	33.0146	72.2	4.366437	72.3	7.632553	72.4	13.62073	72.5	21.60121	72.5	36.36295
72.29999542	3.871999377	72.3999939	6.7351	72.5	12.3319	72.6	18.6992	72.6	32.9165	72.3	4.361476	72.4	7.621201	72.5	13.5967	72.6	21.55343	72.6	36.28123
72.40000153	3.865800384	72.5	6.7179	72.60001	12.2989	72.7	18.6486	72.7	32.8184	72.4	4.356521	72.5	7.609869	72.6	13.57272	72.7	21.50581	72.7	36.19985
72.5	3.851899955	72.6000061	6.6984	72.7	12.2648	72.8	18.6037	72.8	32.7262	72.5	4.351572	72.6	7.598556	72.7	13.54879	72.8	21.45835	72.8	36.11882
72.59999847	3.840799412	72.69999695	6.68	72.8	12.235	72.9	18.5616	72.9	32.6311	72.6	4.346629	72.7	7.587262	72.8	13.52492	72.9	21.41104	72.9	36.03812
72.69999695	3.83020002	72.80000305	6.667	72.89999	12.2021	73	18.5199	73	32.5417	72.7	4.341692	72.8	7.575987	72.9	13.50109	73	21.3639	73	35.95776
72.79999542	3.821399281	72.8999939	6.6525	73	12.1702	73.1	18.4714	73.1	32.4459	72.8	4.336761	72.9	7.564729	73	13.47732	73.1	21.31691	73.1	35.87774
72.90000153	3.810100675	73	6.6406	73.10001	12.135	73.2	18.4222	73.2	32.3551	72.9	4.331835	73	7.553491	73.1	13.45361	73.2	21.27008	73.2	35.79806
73	3.802900022	73.1000061	6.6213	73.2	12.0994	73.3	18.3753	73.3	32.2621	73	4.326916	73.1	7.542272	73.2	13.42995	73.3	21.22341	73.3	35.7187
73.09999847	3.791299574	73.19999695	6.6082	73.3	12.0703	73.4	18.3272	73.4	32.1738	73.1	4.322002	73.2	7.531072	73.3	13.40633	73.4	21.17689	73.4	35.63966
73.19999695	3.786300027	73.30000305	6.5945	73.39999	12.0443	73.5	18.2809	73.5	32.0872	73.2	4.317095	73.3	7.51989	73.4	13.38277	73.5	21.13052	73.5	35.56096
73.29999542	3.788499434	73.3999939	6.5818	73.5	12.0151	73.6	18.2336	73.6	31.9971	73.3	4.312193	73.4	7.508726	73.5	13.35927	73.6	21.08431	73.6	35.48258
73.40000153	3.783100695	73.5	6.5701	73.60001	11.9821	73.7	18.1834	73.7	31.9037	73.4	4.307297	73.5	7.497582	73.6	13.33581	73.7	21.03824	73.7	35.40452
73.5	3.779900016	73.6000061	6.5532	73.7	11.9499	73.8	18.1371	73.8	31.82	73.5	4.302407	73.6	7.486456	73.7	13.31241	73.8	20.99233	73.8	35.32677
73.59999847	3.772599548	73.69999695	6.5403	73.8	11.9243	73.9	18.0904	73.9	31.7355	73.6	4.297522	73.7	7.475349	73.8	13.28906	73.9	20.94657	73.9	35.24934
73.69999695	3.761800004	73.80000305	6.5272	73.89999	11.8957	74	18.0405	74	31.6581	73.7	4.292644	73.8	7.46426	73.9	13.26575	74	20.90096	74	35.17222
73.79999542	3.762299289	73.8999939	6.514	74	11.8644	74.1	17.9945	74.1	31.5767	73.8	4.287772	73.9	7.453188	74	13.24251	74.1	20.8555	74.1	35.09541
73.90000153	3.755900454	74	6.497	74.10001	11.8313	74.2	17.9506	74.2	31.4972	73.9	4.282904	74	7.442136	74.1	13.21931	74.2	20.81018	74.2	35.01891
74	3.748400003	74.1000061	6.4865	74.2	11.7929	74.3	17.9104	74.3	31.4184	74	4.278044	74.1	7.431103	74.2	13.19616	74.3	20.76501	74.3	34.94272
74.09999847	3.738199525	74.19999695	6.4707	74.3	11.7582	74.4	17.8736	74.4	31.3402	74.1	4.273189	74.2	7.420087	74.3	13.17306	74.4	20.71999	74.4	34.86682
74.19999695	3.727999997	74.30000305	6.4573	74.39999	11.7321	74.5	17.8296	74.5	31.2632	74.2	4.268339	74.3	7.40909	74.4	13.15002	74.5	20.67511	74.5	34.79123
74.29999542	3.721599453	74.3999939	6.4489	74.5	11.7037	74.6	17.7918	74.6	31.1893	74.3	4.263496	74.4	7.39811	74.5	13.12702	74.6	20.63038	74.6	34.71594
74.40000153	3.719400705	74.5	6.4438	74.60001	11.6734	74.7	17.7528	74.7	31.1155	74.4	4.258658	74.5	7.38715	74.6	13.10408	74.7	20.58579	74.7	34.64094
74.5	3.714300017	74.6000061	6.4332	74.7	11.6456	74.8	17.7183	74.8	31.0376	74.5	4.253826	74.6	7.376207	74.7	13.08118	74.8	20.54135	74.8	34.56624
74.59999847	3.706799331	74.69999695	6.4217	74.8	11.6155	74.9	17.6727	74.9	30.955	74.6	4.249	74.7	7.365283	74.8	13.05834	74.9	20.49704	74.9	34.49183
74.69999695	3.69919999	74.80000305	6.41	74.89999	11.5873	75	17.6323	75	30.8757	74.7	4.244179	74.8	7.354377	74.9	13.03554	75	20.45288	75	34.41771
74.79999542	3.693199374	74.8999939	6.3956	75	11.5609	75.1	17.5924	75.1	30.7956	74.8	4.239365	74.9	7.343488	75	13.01279	75.1	20.40886	75.1	34.34387
74.90000153	3.686500423	75	6.379	75.10001	11.5296	75.2	17.5505	75.2	30.717	74.9	4.234555	75	7.332618	75.1	12.9901	75.2	20.36498	75.2	34.27033
75	3.677299981	75.1000061	6.3648	75.2	11.4941	75.3	17.5112	75.3	30.6412	75	4.229752	75.1	7.321766	75.2	12.96745	75.3	20.32124	75.3	34.19706
75.09999847	3.668399421	75.19999695	6.3543	75.3	11.462	75.4	17.4751	75.4	30.5651	75.1	4.224955	75.2	7.310931	75.3	12.94485	75.4	20.27763	75.4	34.12407
75.19999695	3.66339999	75.30000305	6.3416	75.39999	11.434	75.5	17.4396	75.5	30.4879	75.2	4.220163	75.3	7.300115	75.4	12.9223	75.5	20.23417	75.5	34.05137
75.29999542	3.657899305	75.3999939	6.3283	75.5	11.4052	75.6	17.4009	75.6	30.4147	75.3	4.215377	75.4	7.289316	75.5	12.89981	75.6	20.19084	75.6	33.97895
75.40000153	3.652300427	75.5	6.3155	75.60001	11.3797	75.7	17.3649	75.7	30.3413	75.4	4.210597	75.5	7.278535	75.6	12.87735	75.7	20.14765	75.7	33.90679
75.5	3.645000012	75.6000061	6.3054	75.7	11.3591	75.8	17.3286	75.8	30.2755	75.5	4.205822	75.6	7.267773	75.7	12.85495	75.8	20.1046	75.8	33.83492
75.59999847	3.63949927	75.69999695	6.2989	75.8	11.3322	75.9	17.2873	75.9	30.2024	75.6	4.201053	75.7	7.257028	75.8	12.8326	75.9	20.06168	75.9	33.76331
75.69999695	3.635800025	75.80000305	6.2906	75.89999	11.3038	76	17.2492	76	30.127	75.7	4.19629	75.8	7.246301	75.9	12.81029	76	20.01889	76	33.69197
75.79999542	3.632999297	75.8999939	6.2746	76	11.2797	76.1	17.2118	76.1	30.0581	75.8	4.191533	75.9	7.235591	76	12.78804	76.1	19.97624	76.1	33.6209

75.90000153	3.628400637	76	6.258	76.10001	11.2539	76.2	17.1733	76.2	29.9871	75.9	4.18678	76	7.224899	76.1	12.76583	76.2	19.93372	76.2	33.5501
76	3.621000021	76.1000061	6.2475	76.2	11.2251	76.3	17.1338	76.3	29.9154	76	4.182034	76.1	7.214225	76.2	12.74367	76.3	19.89134	76.3	33.47956
76.09999847	3.61299948	76.19999695	6.2331	76.3	11.1993	76.4	17.0915	76.4	29.8389	76.1	4.177294	76.2	7.203568	76.3	12.72155	76.4	19.84908	76.4	33.40927
76.19999695	3.604299989	76.30000305	6.2206	76.39999	11.1734	76.5	17.0503	76.5	29.7651	76.2	4.172559	76.3	7.192929	76.4	12.69948	76.5	19.80696	76.5	33.33926
76.29999542	3.598799518	76.3999939	6.2073	76.5	11.145	76.6	17.0129	76.6	29.7011	76.3	4.16783	76.4	7.182307	76.5	12.67747	76.6	19.76496	76.6	33.2695
76.40000153	3.590500569	76.5	6.1957	76.60001	11.1254	76.7	16.977	76.7	29.6353	76.4	4.163106	76.5	7.171703	76.6	12.6555	76.7	19.7231	76.7	33.2
76.5	3.586300009	76.6000061	6.188	76.7	11.1045	76.8	16.942	76.8	29.57	76.5	4.158388	76.6	7.161117	76.7	12.63357	76.8	19.68137	76.8	33.13075
76.59999847	3.583399271	76.69999695	6.1736	76.8	11.0867	76.9	16.9056	76.9	29.5053	76.6	4.153676	76.7	7.150548	76.8	12.6117	76.9	19.63976	76.9	33.06175
76.69999695	3.578999986	76.80000305	6.1621	76.89999	11.0681	77	16.8683	77	29.4352	76.7	4.148969	76.8	7.139996	76.9	12.58986	77	19.59828	77	32.99301
76.79999542	3.577699343	76.8999939	6.1522	77	11.0439	77.1	16.8261	77.1	29.3622	76.8	4.144268	76.9	7.129461	77	12.56808	77.1	19.55693	77.1	32.92451
76.90000153	3.573200505	77	6.1413	77.10001	11.0179	77.2	16.7847	77.2	29.2912	76.9	4.139572	77	7.118944	77.1	12.54635	77.2	19.51571	77.2	32.85627
77	3.5648	77.1000061	6.1249	77.2	10.9919	77.3	16.7542	77.3	29.2283	77	4.134882	77.1	7.108444	77.2	12.52466	77.3	19.47461	77.3	32.78827
77.09999847	3.554899621	77.19999695	6.1119	77.3	10.9687	77.4	16.7191	77.4	29.1695	77.1	4.130198	77.2	7.097962	77.3	12.50301	77.4	19.43363	77.4	32.72051
77.19999695	3.54729997	77.30000305	6.1021	77.39999	10.9458	77.5	16.6896	77.5	29.1104	77.2	4.125519	77.3	7.087497	77.4	12.48141	77.5	19.39278	77.5	32.653
77.29999542	3.539099443	77.3999939	6.0913	77.5	10.9257	77.6	16.6566	77.6	29.0449	77.3	4.120846	77.4	7.077048	77.5	12.45986	77.6	19.35206	77.6	32.58573
77.40000153	3.533800668	77.5	6.0799	77.60001	10.9036	77.7	16.6216	77.7	28.9795	77.4	4.116178	77.5	7.066617	77.6	12.43836	77.7	19.31146	77.7	32.5187
77.5	3.530900016	77.6000061	6.0681	77.7	10.8713	77.8	16.586	77.8	28.9036	77.5	4.111516	77.6	7.056204	77.7	12.4169	77.8	19.27098	77.8	32.45191
77.59999847	3.524799601	77.69999695	6.0553	77.8	10.8452	77.9	16.5491	77.9	28.8289	77.6	4.10686	77.7	7.045807	77.8	12.39548	77.9	19.23062	77.9	32.38535
77.69999695	3.5141	77.80000305	6.0401	77.89999	10.8199	78	16.521	78	28.7574	77.7	4.102209	77.8	7.035428	77.9	12.37411	78	19.19038	78	32.31903
77.79999542	3.508199621	77.8999939	6.0297	78	10.7973	78.1	16.4852	78.1	28.6873	77.8	4.097563	77.9	7.025064	78	12.35279	78.1	19.15027	78.1	32.25295
77.90000153	3.500200467	78	6.0199	78.10001	10.7726	78.2	16.4554	78.2	28.6239	77.9	4.092923	78	7.014719	78.1	12.33151	78.2	19.11028	78.2	32.18709
78	3.494100022	78.1000061	6.0058	78.2	10.751	78.3	16.4211	78.3	28.5603	78	4.088289	78.1	7.00439	78.2	12.31028	78.3	19.07041	78.3	32.12147
78.09999847	3.487399762	78.19999695	5.9996	78.3	10.7282	78.4	16.3806	78.4	28.4961	78.1	4.08366	78.2	6.994079	78.3	12.28909	78.4	19.03065	78.4	32.05607
78.19999695	3.484899977	78.30000305	5.9858	78.39999	10.702	78.5	16.3453	78.5	28.4272	78.2	4.079037	78.3	6.983784	78.4	12.26795	78.5	18.99101	78.5	31.99091
78.29999542	3.479799391	78.3999939	5.9731	78.5	10.6788	78.6	16.3102	78.6	28.3665	78.3	4.074419	78.4	6.973505	78.5	12.24685	78.6	18.9515	78.6	31.92597
78.40000153	3.472500253	78.5	5.9612	78.60001	10.6561	78.7	16.2777	78.7	28.3089	78.4	4.069806	78.5	6.963244	78.6	12.2258	78.7	18.9121	78.7	31.86126
78.5	3.463100026	78.6000061	5.9476	78.7	10.6357	78.8	16.2427	78.8	28.2481	78.5	4.065199	78.6	6.953	78.7	12.20479	78.8	18.87281	78.8	31.79677
78.59999847	3.457999542	78.69999695	5.9365	78.8	10.6122	78.9	16.2162	78.9	28.1879	78.6	4.060598	78.7	6.942772	78.8	12.18383	78.9	18.83365	78.9	31.73249
78.69999695	3.456099982	78.80000305	5.9262	78.89999	10.5934	79	16.1859	79	28.1198	78.7	4.056002	78.8	6.932561	78.9	12.16291	79	18.7946	79	31.66844
78.79999542	3.454799431	78.8999939	5.9205	79	10.5725	79.1	16.152	79.1	28.0589	78.8	4.051411	78.9	6.922366	79	12.14203	79.1	18.75566	79.1	31.60462
78.90000153	3.453400488	79	5.9088	79.10001	10.5518	79.2	16.1177	79.2	27.998	78.9	4.046826	79	6.912189	79.1	12.1212	79.2	18.71684	79.2	31.54101
79	3.44790001	79.1000061	5.8993	79.2	10.5263	79.3	16.0813	79.3	27.9327	79	4.042246	79.1	6.902028	79.2	12.10041	79.3	18.67814	79.3	31.47761
79.09999847	3.450299433	79.19999695	5.8897	79.3	10.5029	79.4	16.0442	79.4	27.8665	79.1	4.037672	79.2	6.891884	79.3	12.07967	79.4	18.63954	79.4	31.41443
79.19999695	3.442499995	79.30000305	5.8735	79.39999	10.4818	79.5	16.0144	79.5	27.8061	79.2	4.033104	79.3	6.881756	79.4	12.05896	79.5	18.60106	79.5	31.35146
79.29999542	3.431399732	79.3999939	5.8616	79.5	10.4584	79.6	15.9821	79.6	27.7478	79.3	4.02854	79.4	6.871644	79.5	12.03831	79.6	18.5627	79.6	31.28871
79.40000153	3.421600528	79.5	5.8525	79.60001	10.4384	79.7	15.9481	79.7	27.6924	79.4	4.023982	79.5	6.86155	79.6	12.01769	79.7	18.52444	79.7	31.22617
79.5	3.415400014	79.6000061	5.8395	79.7	10.4195	79.8	15.9175	79.8	27.6379	79.5	4.01943	79.6	6.851471	79.7	11.99712	79.8	18.4863	79.8	31.16384
79.59999847	3.410099618	79.69999695	5.8284	79.8	10.3959	79.9	15.8845	79.9	27.577	79.6	4.014883	79.7	6.84141	79.8	11.9766	79.9	18.44827	79.9	31.10171
79.69999695	3.406100012	79.80000305	5.8171	79.89999	10.3728	80	15.85	80	27.5141	79.7	4.010341	79.8	6.831364	79.9	11.95611	80	18.41035	80	31.03979
79.79999542	3.402399481	79.8999939	5.8061	80	10.3484	80.1	15.8203	80.1	27.4512	79.8	4.005805	79.9	6.821334	80	11.93567	80.1	18.37254	80.1	30.97809
79.90000153	3.392900238	80	5.7942	80.10001	10.3297	80.2	15.788	80.2	27.3922	79.9	4.001274	80	6.811322	80.1	11.91527	80.2	18.33484	80.2	30.91658
80	3.38760001	80.1000061	5.7871	80.2	10.3128	80.3	15.7606	80.3	27.3358	80	3.996748	80.1	6.801325	80.2	11.89491	80.3	18.29724	80.3	30.85528
80.09999847	3.379799496	80.19999695	5.7788	80.3	10.296	80.4	15.728	80.4	27.2824	80.1	3.992228	80.2	6.791345	80.3	11.8746	80.4	18.25976	80.4	30.79418
80.19999695	3.375399989	80.30000305	5.7696	80.39999	10.2758	80.5	15.694	80.5	27.2257	80.2	3.987714	80.3	6.781381	80.4	11.85433	80.5	18.22238	80.5	30.73328
80.29999542	3.371099419	80.3999939	5.7561	80.5	10.2497	80.6	15.6621	80.6	27.1653	80.3	3.983204	80.4	6.771433	80.5	11.8341	80.6	18.18512	80.6	30.67259
80.40000153	3.367300342	80.5	5.7468	80.60001	10.2268	80.7	15.6309	80.7	27.1058	80.4	3.9787	80.5	6.761501	80.6	11.81391	80.7	18.14796	80.7	30.61209
80.5	3.361799986	80.6000061	5.738	80.7	10.2038	80.8	15.6017	80.8	27.0514	80.5	3.974201	80.6	6.751586	80.7	11.79376	80.8	18.1109	80.8	30.55179
80.59999847	3.353999781	80.69999695	5.7271	80.8	10.1848	80.9	15.5699	80.9	26.9955	80.6	3.969708	80.7	6.741687	80.8	11.77366	80.9	18.07395	80.9	30.49169
80.69999695	3.351600012	80.80000305	5.7157	80.89999	10.1643	81	15.543	81	26.9348	80.7	3.96522	80.8	6.731804	80.9	11.7536	81	18.03711	81	30.43178
80.79999542	3.347099521	80.8999939	5.7039	81	10.1421	81.1	15.5117	81.1	26.8816	80.8	3.960737	80.9	6.721936	81	11.73358	81.1	18.00037	81.1	30.37207
80.90000153	3.340100447	81	5.6942	81.10001	10.1232	81.2	15.4808	81.2	26.831	80.9	3.95626	81	6.712085	81.1	11.7136	81.2	17.96374	81.2	30.31255
81	3.334900008	81.1000061	5.6864	81.2	10.105	81.3	15.4506	81.3	26.7809	81	3.951787	81.1	6.702249	81.2	11.69366	81.3	17.92722	81.3	30.25322
81.09999847	3.332899481	81.19999695	5.6779	81.3	10.084	81.4	15.4209	81.4	26.7312	81.1	3.947321	81.2	6.69243	81.3	11.67377	81.4	17.89079	81.4	30.19408
81.19999695	3.331099997	81.30000305	5.6694	81.39999	10.063	81.5	15.3906	81.5	26.6791	81.2	3.942859	81.3	6.682627	81.4	11.65391	81.5	17.85447	81.5	30.13513
81.29999542	3.324499657	81.3999939	5.6595	81.5	10.0431	81.6	15.3567	81.6	26.622	81.3	3.938403	81.4	6.672839	81.5	11.6341	81.6	17.81825	81.6	30.07637
81.40000153	3.319500258	81.5	5.6469	81.60001	10.023	81.7	15.3264	81.7	26.5706	81.4	3.933952	81.5	6.663068	81.6	11.61433	81.7	17.78214	81.7	30.0178
81.5	3.311399988	81.6000061	5.6333	81.7	10.0014	81.8	15.2942	81.8	26.5156	81.5	3.929506	81.6	6.653312	81.7	11.59459	81.8	17.74612	81.8	29.95942
81.59999847	3.302699833	81.69999695	5.6246	81.8	9.9769	81.9	15.2624	81.9	26.4606	81.6	3.925066	81.7	6.643572	81.8	11.5749	81.9	17.71021	81.9	29.90122
81.69999695	3.299600004	81.80000305	5.6097	81.89999	9.9547	82	15.2364	82	26.4102	81.7	3.920631	81.8	6.633848	81.9	11.55525	82	17.6744	82	29.84321
81.79999542	3.29119981	81.8999939	5.5983	82	9.9367	82.1	15.206	82.1	26.3566	81.8	3.916201	81.9	6.624139	82	11.53564	82.1	17.63869	82.1	29.78538
81.90000153	3.284100512	82	5.5932	82.10001	9.9115	82.2	15.1742	82.2	26.3022	81.9	3.911776	82	6.614446	82.1	11.51607	82.2	17.60308	82.2	29.72773
82	3.278699988	82.1000061	5.5834	82.2	9.8934	82.3	15.1469	82.3	26.2468	82	3.907357	82.1	6.604769	82.2	11.49654	82.3	17.56757	82.3	29.67027
82.09999847	3.274299761	82.19999695	5.5774	82.3	9.8817	82.4	15.1201	82.4	26.1955	82.1	3.902943	82.2	6.595108	82.3	11.47706	82.4	17.53216	82.4	29.61298
82.19999695	3.271499998	82.30000305	5.5733	82.39999	9.8683	82.5	15.0964	82.5	26.1514	82.2	3.898534	82.3	6.585462	82.4	11.45761	82.5	17.49684	82.5	29.55587
82.29999542	3.270599474	82.3999939	5.5691	82.5	9.8547	82.6	15.0693	82.6	26.1028	82.3	3.89413	82.4	6.575832	82.5	11.4382	82.6	17.46163	82.6	29.49895
82.40000153	3.271100679	82.5	5.5616	82.60001	9.8415	82.7	15.0381	82.7	26.0514	82.4	3.889731	82.5	6.566217	82.6	11.41883	82.7	17.42652	82.7	29.4422
82.5	3.270099997	82.6000061	5.5545	82.7	9.8205	82.8	15.009	82.8	25.9992	82.5	3.885338	82.6	6.556618	82.7	11.3995	82.8	17.3915	82.8	29.38563
82.59999847	3.270599338	82.69999695	5.5427	82.8	9.7984	82.9	14.9814	82.9	25.9491	82.6	3.88095	82.7	6.547035	82.8	11.38021	82.9	17.35658	82.9	29.32923
82.69999695	3.263000015	82.80000305	5.535	82.89999	9.7788	83	14.9551	83	25.8949	82.7	3.876567	82.8	6.537467	82.9	11.36096	83	17.32175	83	29.273
82.79999542	3.25669984	82.8999939	5.5222	83	9.7632	83.1	14.9298	83.1	25.8502	82.8	3.872189	82.9	6.527913	83	11.34175	83.1	17.28702	83.1	29.21695
82.90000153	3.250000428	83	5.5117	83.10001	9.7491	83.2	14.909	83.2	25.8079	82.9	3.867817	83	6.518376	83.1	11.32258	83.2	17.25239	83.2	29.16108
83	3.246100003	83.1000061	5.5059	83.2	9.7364	83.3	14.8826	83.3	25.7556	83	3.863449	83.1	6.508854	83.2	11.30344	83.3	17.21786	83.3	29.10538
83.09999847	3.244099456	83.19999695	5.503	83.3	9.7141	83.4	14.854	83.4	25.7046	83.1	3.859087	83.2	6.499348	83.3	11.28435	83.4	17.18341	83.4	29.04984
83.19999695	3.246499999	83.30000305	5.4919	83.39999	9.6928	83.5	14.8299	83.5	25.6505	83.2	3.85473	83.3	6.489857	83.4	11.26529	83.5	17.14907	83.5	28.99448
83.29999542	3.23649985	83.3999939	5.4806	83.5	9.6724	83.6	14.7991	83.6	25.5952	83.3	3.850378	83.4	6.48038	83.5	11.24628	83.6	17.11482	83.6	28.93928
83.40000153	3.231200349	83.5	5.4735	83.60001	9.6484	83.7	14.7729	83.7	25.5377	83.4	3.846031	83.5	6.47092	83.6	11.2273	83.7	17.08066	83.7	28.88426
83.5	3.225499993	83.6000061	5.465	83.7	9.6247	83.8	14.7486	83.8	25.4916	83.5	3.84169	83.6	6.461475	83.7	11.20836	83.8	17.04659	83.8	28.8294
83.59999847	3.219199788	83.69999695	5.4525	83.8	9.6118	83.9	14.7217	83.9	25.4463	83.6	3.837353	83.7	6.452045	83.8	11.18947	83.9	17.01262	83.9	28.7747
83.69999695	3.211599988	83.80000305	5.4447	83.89999	9.59	84	14.6922	84	25.3983	83.7	3.833022	83.8	6.44263	83.9	11.1706	84	16.97874	84	28.72018
83.79999542	3.208299468	83.8999939	5.4351	84	9.5712	84.1	14.6642	84.1	25.3497	83.8	3.828696	83.9	6.433229	84	11.15178	84.1	16.94496	84.1	28.66581
83.90000153	3.202100313	84	5.4246	84.10001	9.5565	84.2	14.6378	84.2	25.2997	83.9	3.824375	84	6.423845	84.1	11.133	84.2	16.91126	84.2	28.61162
84	3.199899997	84.1000061	5.4155	84.2	9.5437	84.3	14.607	84.3	25.2495	84	3.820059	84.1	6.414475	84.2	11.11425	84.3	16.87766	84.3	28.55758
84.09999847	3.195199486	84.19999695	5.4085	84.3	9.5245	84.4	14.5804	84.4	25.1997	84.1	3.815748	84.2	6.405121	84.3	11.09554	84.4	16.84414	84.4	28.5037
84.19999695	3.188500002	84.30000305	5.3987	84.39999	9.5061	84.5	14.5574	84.5	25.1532	84.2	3.811442	84.3	6.395781	84.4	11.07687	84.5	16.81072	84.5	28.44999
84.29999542	3.179799893	84.3999939	5.3915	84.5	9.4904	84.6	14.5291	84.6	25.1113	84.3	3.807141	84.4	6.386456	84.5	11.05824	84.6	16.77739	84.6	28.39644
84.40000153	3.174400408	84.5	5.3814	84.60001	9.4732	84.7	14.5044	84.7	25.0618	84.4	3.802845	84.5	6.377146	84.6	11.03964	84.7	16.74415	84.7	28.34305
84.5	3.170800006	84.6000061	5.377	84.7	9.4595	84.8	14.482	84.8	25.0161	84.5	3.798555	84.6	6.367852	84.7	11.02108	84.8	16.711	84.8	28.28981
84.59999847	3.167399492	84.69999695	5.3694	84.8	9.4451	84.9	14.4589	84.9	24.9679	84.6	3.794269	84.7	6.358572	84.8	11.00256	84.9	16.67794	84.9	28.23673
84.69999695	3.162999997	84.80000305	5.3643	84.89999	9.4279	85	14.4285	85	24.9144	84.7	3.789989	84.8	6.349308	84.9	10.98408	85	16.64497	85	28.18382
84.79999542	3.159399464	84.8999939	5.3586	85	9.4035	85.1	14.4003	85.1	24.8659	84.8	3.785714	84.9	6.340057	85	10.96564	85.1	16.61208	85.1	28.13105
84.90000153	3.152900113	85	5.3461	85.10001	9.3854	85.2	14.3735	85.2	24.8133	84.9	3.781443	85	6.330822	85.1	10.94723	85.2	16.57929	85.2	28.07845
85	3.144900004	85.1000061	5.34	85.2	9.3674	85.3	14.3462	85.3	24.7646	85	3.777178	85.1	6.321602	85.2	10.92886	85.3	16.54658	85.3	28.02599
85.09999847	3.137799661	85.19999695	5.33	85.3	9.3532	85.4	14.3226	85.4	24.7205	85.1	3.772918	85.2	6.312396	85.3	10.91052	85.4	16.51396	85.4	27.97369
85.19999695	3.1272	85.30000305	5.3223	85.39999	9.3362	85.5	14.302	85.5	24.6785	85.2	3.768663	85.3	6.303206	85.4	10.89222	85.5	16.48143	85.5	27.92155
85.29999542	3.121399634	85.3999939	5.3137	85.5	9.322	85.6	14.2731	85.6	24.6334	85.3	3.764412	85.4	6.294029	85.5	10.87396	85.6	16.44898	85.6	27.86955
85.40000153	3.119100565	85.5	5.3084	85.60001	9.3039	85.7	14.2492	85.7	24.592	85.4	3.760167	85.5	6.284868	85.6	10.85574	85.7	16.41662	85.7	27.81771
85.5	3.117300007	85.6000061	5.2985	85.7	9.2863	85.8	14.2237	85.8	24.5415	85.5	3.755927	85.6	6.275721	85.7	10.83755	85.8	16.38435	85.8	27.76602
85.59999847	3.115399736	85.69999695	5.2913	85.8	9.2647	85.9	14.1961	85.9	24.4939	85.6	3.751692	85.7	6.266589	85.8	10.8194	85.9	16.35216	85.9	27.71448
85.69999695	3.112999993	85.80000305	5.282	85.89999	9.2478	86	14.1695	86	24.4489	85.7	3.747462	85.8	6.257472	85.9	10.80129	86	16.32005	86	27.66309
85.79999542	3.10379986	85.8999939	5.2722	86	9.2355	86.1	14.1505	86.1	24.4061	85.8	3.743236	85.9	6.248368	86	10.78321	86.1	16.28804	86.1	27.61185
85.90000153	3.096500243	86	5.2618	86.10001	9.2245	86.2	14.1273	86.2	24.3605	85.9	3.739016	86	6.23928	86.1	10.76517	86.2	16.2561	86.2	27.56075
86	3.089599998	86.1000061	5.2553	86.2	9.2072	86.3	14.1072	86.3	24.3166	86	3.734801	86.1	6.230207	86.2	10.74716	86.3	16.22426	86.3	27.5098
86.09999847	3.086699375	86.19999695	5.2461	86.3	9.1911	86.4	14.0787	86.4	24.27	86.1	3.73059	86.2	6.221147	86.3	10.7292	86.4	16.19249	86.4	27.459
86.19999695	3.083599996	86.30000305	5.2377	86.39999	9.1755	86.5	14.054	86.5	24.2226	86.2	3.726385	86.3	6.212103	86.4	10.71126	86.5	16.16081	86.5	27.40834
86.29999542	3.081299679	86.3999939	5.2294	86.5	9.1601	86.6	14.0319	86.6	24.1778	86.3	3.722185	86.4	6.203072	86.5	10.69336	86.6	16.12921	86.6	27.35783
86.40000153	3.074000392	86.5	5.227	86.60001	9.1488	86.7	14.011	86.7	24.1355	86.4	3.717989	86.5	6.194056	86.6	10.6755	86.7	16.0977	86.7	27.30747
86.5	3.067900003	86.6000061	5.2188	86.7	9.1341	86.8	13.992	86.8	24.094	86.5	3.713799	86.6	6.185054	86.7	10.65767	86.8	16.06627	86.8	27.25725
86.59999847	3.067099527	86.69999695	5.2099	86.8	9.1247	86.9	13.9692	86.9	24.0436	86.6	3.709613	86.7	6.176067	86.8	10.63988	86.9	16.03491	86.9	27.20716
86.69999695	3.067399996	86.80000305	5.2085	86.89999	9.1082	87	13.945	87	23.9982	86.7	3.705433	86.8	6.167095	86.9	10.62213	87	16.00365	87	27.15722
86.79999542	3.064799497	86.8999939	5.1992	87	9.0901	87.1	13.9206	87.1	23.9515	86.8	3.701257	86.9	6.158135	87	10.60441	87.1	15.97246	87.1	27.10743
86.90000153	3.057000265	87	5.191	87.10001	9.0722	87.2	13.9013	87.2	23.9096	86.9	3.697086	87	6.149191	87.1	10.58672	87.2	15.94136	87.2	27.05777
87	3.052099996	87.1000061	5.1831	87.2	9.058	87.3	13.8814	87.3	23.8672	87	3.692921	87.1	6.140261	87.2	10.56907	87.3	15.91034	87.3	27.00826
87.09999847	3.047699716	87.19999695	5.1759	87.3	9.0448	87.4	13.8593	87.4	23.8245	87.1	3.68876	87.2	6.131346	87.3	10.55146	87.4	15.87939	87.4	26.95888
87.19999695	3.045000003	87.30000305	5.1722	87.39999	9.0297	87.5	13.8359	87.5	23.779	87.2	3.684604	87.3	6.122444	87.4	10.53388	87.5	15.84853	87.5	26.90964
87.29999542	3.043399478	87.3999939	5.1668	87.5	9.016	87.6	13.8161	87.6	23.7375	87.3	3.680453	87.4	6.113556	87.5	10.51633	87.6	15.81775	87.6	26.86054

87.40000153	3.040700291	87.5	5.1583	87.60001	9.0042	87.7	13.7984	87.7	23.6973	87.4	3.676306	87.5	6.104683	87.6	10.49883	87.7	15.78705	87.7	26.81158
87.5	3.033999999	87.6000061	5.158	87.7	8.9976	87.8	13.7791	87.8	23.6536	87.5	3.672165	87.6	6.095824	87.7	10.48135	87.8	15.75642	87.8	26.76276
87.59999847	3.037299378	87.69999695	5.158	87.8	8.9819	87.9	13.7571	87.9	23.6085	87.6	3.668028	87.7	6.086979	87.8	10.46391	87.9	15.72588	87.9	26.71406
87.69999695	3.038600001	87.80000305	5.1501	87.89999	8.9669	88	13.7323	88	23.5693	87.7	3.663897	87.8	6.078148	87.9	10.4465	88	15.69541	88	26.66551
87.79999542	3.031599757	87.8999939	5.1402	88	8.9558	88.1	13.7025	88.1	23.5261	87.8	3.65977	87.9	6.069331	88	10.42913	88.1	15.66503	88.1	26.61709
87.90000153	3.030700448	88	5.1296	88.10001	8.9362	88.2	13.6833	88.2	23.4885	87.9	3.655648	88	6.060528	88.1	10.4118	88.2	15.63472	88.2	26.56881
88	3.027299996	88.1000061	5.1189	88.2	8.9232	88.3	13.6625	88.3	23.4479	88	3.651531	88.1	6.051739	88.2	10.39449	88.3	15.60449	88.3	26.52066
88.09999847	3.021999905	88.19999695	5.1157	88.3	8.9068	88.4	13.6436	88.4	23.4123	88.1	3.647419	88.2	6.042965	88.3	10.37722	88.4	15.57434	88.4	26.47264
88.19999695	3.020200004	88.30000305	5.1046	88.39999	8.8926	88.5	13.6207	88.5	23.3675	88.2	3.643312	88.3	6.034204	88.4	10.35999	88.5	15.54426	88.5	26.42475
88.29999542	3.016699746	88.3999939	5.0919	88.5	8.8792	88.6	13.5953	88.6	23.3236	88.3	3.639209	88.4	6.025456	88.5	10.34279	88.6	15.51427	88.6	26.377
88.40000153	3.014600262	88.5	5.086	88.60001	8.8616	88.7	13.5769	88.7	23.2848	88.4	3.635111	88.5	6.016723	88.6	10.32562	88.7	15.48435	88.7	26.32938
88.5	3.010699999	88.6000061	5.0792	88.7	8.8451	88.8	13.5577	88.8	23.2414	88.5	3.631019	88.6	6.008004	88.7	10.30849	88.8	15.4545	88.8	26.28189
88.59999847	3.006699774	88.69999695	5.0721	88.8	8.8265	88.9	13.5368	88.9	23.2022	88.6	3.626931	88.7	5.999299	88.8	10.29139	88.9	15.42473	88.9	26.23452
88.69999695	3.000200001	88.80000305	5.0633	88.89999	8.8108	89	13.5162	89	23.1615	88.7	3.622847	88.8	5.990607	88.9	10.27432	89	15.39504	89	26.18729
88.79999542	2.996799726	88.8999939	5.0548	89	8.8004	89.1	13.4945	89.1	23.1165	88.8	3.618769	88.9	5.981929	89	10.25729	89.1	15.36543	89.1	26.14019
88.90000153	2.993500404	89	5.0491	89.10001	8.7852	89.2	13.4719	89.2	23.0729	88.9	3.614695	89	5.973265	89.1	10.24029	89.2	15.33589	89.2	26.09321
89	2.990899998	89.1000061	5.042	89.2	8.7693	89.3	13.4486	89.3	23.0325	89	3.610626	89.1	5.964614	89.2	10.22333	89.3	15.30642	89.3	26.04636
89.09999847	2.98519981	89.19999695	5.0339	89.3	8.7503	89.4	13.4312	89.4	22.9906	89.1	3.606562	89.2	5.955978	89.3	10.2064	89.4	15.27703	89.4	25.99964
89.19999695	2.981399999	89.30000305	5.02	89.39999	8.7393	89.5	13.4163	89.5	22.952	89.2	3.602503	89.3	5.947355	89.4	10.1895	89.5	15.24771	89.5	25.95305
89.29999542	2.975299994	89.3999939	5.0161	89.5	8.7259	89.6	13.3951	89.6	22.9163	89.3	3.598449	89.4	5.938745	89.5	10.17263	89.6	15.21847	89.6	25.90658
89.40000153	2.974900382	89.5	5.014	89.60001	8.7093	89.7	13.3771	89.7	22.8822	89.4	3.594398	89.5	5.930149	89.6	10.1558	89.7	15.1893	89.7	25.86023
89.5	2.970700001	89.6000061	5.0056	89.7	8.6988	89.8	13.3573	89.8	22.8451	89.5	3.590354	89.6	5.921567	89.7	10.139	89.8	15.16021	89.8	25.81402
89.59999847	2.966499788	89.69999695	4.9999	89.8	8.6861	89.9	13.3378	89.9	22.8036	89.6	3.586313	89.7	5.912999	89.8	10.12224	89.9	15.13118	89.9	25.76792
89.69999695	2.966099999	89.80000305	4.9933	89.89999	8.671199	90	13.3176	90	22.7628	89.7	3.582278	89.8	5.904444	89.9	10.1055	90	15.10223	90	25.72195
89.79999542	2.968699534	89.8999939	4.986199	90	8.6556	90.1	13.2982	90.1	22.7244	89.8	3.578247	89.9	5.895902	90	10.0888	90.1	15.07336	90.1	25.6761
89.90000153	2.968599641	90	4.9829	90.10001	8.6438	90.2	13.2774	90.2	22.681	89.9	3.574221	90	5.887374	90.1	10.07213	90.2	15.04455	90.2	25.63037
90	2.966099999	90.1000061	4.9772	90.2	8.6285	90.3	13.2582	90.3	22.6435	90	3.5702	90.1	5.87886	90.2	10.0555	90.3	15.01583	90.3	25.58477
90.09999847	2.962699652	90.19999695	4.9713	90.3	8.6131	90.4	13.2388	90.4	22.6055	90.1	3.566183	90.2	5.870358	90.3	10.03889	90.4	14.98716	90.4	25.53929
90.20000458	2.960400335	90.30000305	4.9637	90.40001	8.6015	90.50001	13.2183	90.50001	22.5663	90.2	3.562171	90.3	5.861872	90.4	10.02232	90.49999	14.95858	90.49999	25.49393
90.29999542	2.960399664	90.3999939	4.957799	90.5	8.5893	90.6	13.1955	90.6	22.5264	90.3	3.558164	90.4	5.853397	90.49999	10.00578	90.6	14.93006	90.6	25.44869
90.40000153	2.958799481	90.5	4.9523	90.60001	8.5776	90.7	13.1752	90.7	22.4843	90.4	3.554162	90.49999	5.844937	90.6	9.989277	90.7	14.90162	90.7	25.40356
90.49999237	2.955399687	90.59999084	4.945699	90.7	8.5634	90.8	13.1545	90.8	22.4472	90.49999	3.550164	90.6	5.836489	90.7	9.972801	90.8	14.87325	90.8	25.35856
90.59999847	2.949199733	90.69999695	4.9313	90.8	8.5499	90.9	13.131	90.9	22.4037	90.6	3.546171	90.7	5.828054	90.8	9.95636	90.9	14.84494	90.9	25.31368
90.70000458	2.944000335	90.80000305	4.9225	90.90001	8.5324	91.00001	13.1075	91.00001	22.3615	90.7	3.542182	90.8	5.819634	90.9	9.939949	90.99999	14.81671	90.99999	25.26892
90.79999542	2.944099665	90.8999939	4.9104	91	8.5153	91.1	13.0826	91.1	22.3212	90.8	3.538199	90.9	5.811226	90.99999	9.923572	91.1	14.78855	91.1	25.22427
90.90000153	2.938600069	91	4.8986	91.10001	8.5027	91.2	13.0651	91.2	22.2858	90.9	3.53422	90.99999	5.802833	91.1	9.907224	91.2	14.76046	91.2	25.17973
90.99999237	2.933199969	91.09999084	4.8916	91.2	8.4945	91.3	13.0504	91.3	22.2486	90.99999	3.530246	91.1	5.794451	91.2	9.890908	91.3	14.73244	91.3	25.13532
91.09999847	2.928100015	91.19999695	4.8875	91.3	8.4847	91.4	13.0343	91.4	22.2127	91.1	3.526276	91.2	5.786083	91.3	9.874626	91.4	14.70448	91.4	25.09102
91.20000458	2.928000337	91.30000305	4.8801	91.40001	8.4723	91.50001	13.0151	91.50001	22.1715	91.2	3.522311	91.3	5.777729	91.4	9.858374	91.49999	14.6766	91.49999	25.04685
91.29999542	2.927499664	91.3999939	4.871099	91.5	8.4594	91.6	12.9916	91.6	22.1303	91.3	3.51835	91.4	5.769387	91.49999	9.842155	91.6	14.64878	91.6	25.00278
91.40000153	2.925999725	91.5	4.8634	91.60001	8.4414	91.7	12.9704	91.7	22.0925	91.4	3.514395	91.49999	5.76106	91.6	9.825965	91.7	14.62104	91.7	24.95882
91.49999237	2.923699763	91.59999084	4.853	91.7	8.4255	91.8	12.9453	91.8	22.0542	91.49999	3.510444	91.6	5.752744	91.7	9.809806	91.8	14.59336	91.8	24.91499
91.59999847	2.918400045	91.69999695	4.8458	91.8	8.4081	91.9	12.9279	91.9	22.0225	91.6	3.506497	91.7	5.744441	91.8	9.793682	91.9	14.56575	91.9	24.87126
91.70000458	2.910100031	91.80000305	4.839	91.90001	8.3967	92.00001	12.9086	92.00001	21.9847	91.7	3.502555	91.8	5.736153	91.9	9.777586	91.99999	14.53821	91.99999	24.82766
91.79999542	2.906699971	91.8999939	4.834799	92	8.3852	92.1	12.8915	92.1	21.9486	91.8	3.498618	91.9	5.727877	91.99999	9.761523	92.1	14.51073	92.1	24.78416
91.90000153	2.90759961	92	4.8245	92.10001	8.3718	92.2	12.8716	92.2	21.9087	91.9	3.494685	91.99999	5.719614	92.1	9.745489	92.2	14.48333	92.2	24.74077
91.99999237	2.904599758	92.09999084	4.819099	92.2	8.3601	92.3	12.8503	92.3	21.8736	91.99999	3.490758	92.1	5.711364	92.2	9.729486	92.3	14.45599	92.3	24.6975
92.09999847	2.902699573	92.19999695	4.8138	92.3	8.3462	92.4	12.8249	92.4	21.8386	92.1	3.486834	92.2	5.703126	92.3	9.713516	92.4	14.42871	92.4	24.65434
92.20000458	2.901000245	92.30000305	4.8104	92.40001	8.3351	92.50001	12.8067	92.50001	21.805	92.2	3.482915	92.3	5.694903	92.4	9.697575	92.49999	14.40151	92.49999	24.61129
92.29999542	2.900399756	92.3999939	4.803499	92.5	8.3241	92.6	12.7857	92.6	21.773	92.3	3.479001	92.4	5.686692	92.49999	9.681666	92.6	14.37437	92.6	24.56835
92.40000153	2.898499748	92.5	4.796	92.60001	8.3114	92.7	12.7601	92.7	21.7345	92.4	3.475091	92.49999	5.678494	92.6	9.665786	92.7	14.3473	92.7	24.52551
92.49999237	2.898999681	92.59999084	4.786	92.7	8.2967	92.8	12.7423	92.8	21.6988	92.49999	3.471187	92.6	5.670308	92.7	9.649937	92.8	14.32029	92.8	24.48279
92.59999847	2.897099785	92.69999695	4.7783	92.8	8.2834	92.9	12.7206	92.9	21.6618	92.6	3.467286	92.7	5.662135	92.8	9.63412	92.9	14.29335	92.9	24.44018
92.70000458	2.889700175	92.80000305	4.7732	92.90001	8.2674	93.00001	12.7015	93.00001	21.6276	92.7	3.46339	92.8	5.653976	92.9	9.618331	92.99999	14.26648	92.99999	24.39768
92.79999542	2.883699823	92.8999939	4.7657	93	8.2508	93.1	12.6808	93.1	21.5927	92.8	3.459499	92.9	5.645829	92.99999	9.602575	93.1	14.23966	93.1	24.35528
92.90000153	2.878199861	93	4.7616	93.10001	8.2389	93.2	12.6657	93.2	21.5631	92.9	3.455612	92.99999	5.637695	93.1	9.586847	93.2	14.21292	93.2	24.31299
92.99999237	2.874299911	93.09999084	4.757299	93.2	8.2314	93.3	12.6458	93.3	21.5297	92.99999	3.45173	93.1	5.629574	93.2	9.571149	93.3	14.18624	93.3	24.27081
93.09999847	2.87349969	93.19999695	4.7548	93.3	8.2195	93.4	12.6258	93.4	21.4937	93.1	3.447852	93.2	5.621465	93.3	9.555484	93.4	14.15962	93.4	24.22874
93.20000458	2.875200177	93.30000305	4.7482	93.40001	8.2051	93.50001	12.6042	93.50001	21.4611	93.2	3.443979	93.3	5.613369	93.4	9.539846	93.49999	14.13307	93.49999	24.18677
93.29999542	2.872899821	93.3999939	4.7381	93.5	8.1919	93.6	12.5857	93.6	21.4289	93.3	3.440111	93.4	5.605286	93.49999	9.524241	93.6	14.10658	93.6	24.14491
93.40000153	2.870499981	93.5	4.7298	93.60001	8.1755	93.7	12.5654	93.7	21.3894	93.4	3.436246	93.49999	5.597216	93.6	9.508663	93.7	14.08016	93.7	24.10315
93.49999237	2.866099802	93.59999084	4.7264	93.7	8.1634	93.8	12.5429	93.8	21.3511	93.49999	3.432387	93.6	5.589157	93.7	9.493115	93.8	14.0538	93.8	24.0615
93.59999847	2.867499759	93.69999695	4.7166	93.8	8.1458	93.9	12.5182	93.9	21.3129	93.6	3.428532	93.7	5.581112	93.8	9.477599	93.9	14.02751	93.9	24.01995
93.70000458	2.861499925	93.80000305	4.7057	93.90001	8.1325	94.00001	12.5001	94.00001	21.281	93.7	3.424681	93.8	5.57308	93.9	9.46211	93.99999	14.00128	93.99999	23.97851
93.79999542	2.85850008	93.8999939	4.6961	94	8.1212	94.1	12.4829	94.1	21.2455	93.8	3.420836	93.9	5.565059	93.99999	9.446653	94.1	13.97511	94.1	23.93717
93.90000153	2.854300059	94	4.6921	94.10001	8.1056	94.2	12.464	94.2	21.2118	93.9	3.416994	93.99999	5.557052	94.1	9.431223	94.2	13.949	94.2	23.89594
93.99999237	2.849099805	94.09999084	4.6895	94.2	8.093	94.3	12.4412	94.3	21.1742	93.99999	3.413157	94.1	5.549057	94.2	9.415823	94.3	13.92296	94.3	23.85481
94.09999847	2.841599852	94.19999695	4.6846	94.3	8.0797	94.4	12.4225	94.4	21.1405	94.1	3.409325	94.2	5.541074	94.3	9.400454	94.4	13.89697	94.4	23.81377
94.20000458	2.836799979	94.30000305	4.6792	94.40001	8.068	94.50001	12.4006	94.50001	21.1042	94.2	3.405496	94.3	5.533104	94.4	9.385113	94.49999	13.87106	94.49999	23.77285
94.29999542	2.831500026	94.3999939	4.6717	94.5	8.0558	94.6	12.3846	94.6	21.0707	94.3	3.401673	94.4	5.525146	94.49999	9.369803	94.6	13.8452	94.6	23.73202
94.40000153	2.825099996	94.5	4.6675	94.60001	8.0454	94.7	12.3657	94.7	21.0357	94.4	3.397854	94.49999	5.517202	94.6	9.35452	94.7	13.8194	94.7	23.69129
94.49999237	2.822599954	94.59999084	4.660199	94.7	8.0378	94.8	12.3428	94.8	21.003	94.49999	3.394039	94.6	5.509269	94.7	9.339265	94.8	13.79367	94.8	23.65067
94.59999847	2.820799744	94.69999695	4.656	94.8	8.0299	94.9	12.3277	94.9	20.9702	94.6	3.390229	94.7	5.501348	94.8	9.324043	94.9	13.76799	94.9	23.61015
94.70000458	2.819100173	94.80000305	4.6479	94.90001	8.0215	95.00001	12.3123	95.00001	20.9429	94.7	3.386423	94.8	5.493441	94.9	9.308846	94.99999	13.74238	94.99999	23.56973
94.79999542	2.820899824	94.8999939	4.645399	95	8.0142	95.1	12.2939	95.1	20.9101	94.8	3.382622	94.9	5.485545	94.99999	9.293681	95.1	13.71683	95.1	23.5294
94.90000153	2.823699607	95	4.6448	95.10001	7.9995	95.2	12.2745	95.2	20.8763	94.9	3.378825	94.99999	5.477662	95.1	9.278543	95.2	13.69134	95.2	23.48918
94.99999237	2.82379947	95.09999084	4.6435	95.2	7.9883	95.3	12.2574	95.3	20.8446	94.99999	3.375033	95.1	5.469791	95.2	9.263433	95.3	13.66591	95.3	23.44905
95.09999847	2.821799883	95.19999695	4.6371	95.3	7.9776	95.4	12.2418	95.4	20.8127	95.1	3.371245	95.2	5.461931	95.3	9.248354	95.4	13.64054	95.4	23.40902
95.20000458	2.819700287	95.30000305	4.6314	95.40001	7.9696	95.50001	12.2237	95.50001	20.7844	95.2	3.367461	95.3	5.454086	95.4	9.233301	95.49999	13.61524	95.49999	23.3691
95.29999542	2.81679971	95.3999939	4.628999	95.5	7.9592	95.6	12.2055	95.6	20.7508	95.3	3.363683	95.4	5.446251	95.49999	9.21828	95.6	13.58999	95.6	23.32926
95.40000153	2.815899641	95.5	4.6196	95.60001	7.9493	95.7	12.1916	95.7	20.7188	95.4	3.359908	95.49999	5.43843	95.6	9.203284	95.7	13.5648	95.7	23.28953
95.49999237	2.815799736	95.59999084	4.611799	95.7	7.9455	95.8	12.1755	95.8	20.6855	95.49999	3.356138	95.6	5.430619	95.7	9.188317	95.8	13.53967	95.8	23.2499
95.59999847	2.818699734	95.69999695	4.608	95.8	7.9339	95.9	12.1574	95.9	20.6525	95.6	3.352372	95.7	5.422821	95.8	9.17338	95.9	13.5146	95.9	23.21035
95.70000458	2.817100227	95.80000305	4.6019	95.90001	7.921	96.00001	12.1367	96.00001	20.6173	95.7	3.34861	95.8	5.415036	95.9	9.15847	95.99999	13.48959	95.99999	23.17091
95.79999542	2.813699771	95.8999939	4.5986	96	7.9063	96.1	12.1182	96.1	20.5827	95.8	3.344853	95.9	5.407263	95.99999	9.143589	96.1	13.46463	96.1	23.13156
95.90000153	2.806499873	96	4.5861	96.10001	7.8964	96.2	12.0979	96.2	20.5473	95.9	3.3411	95.99999	5.399502	96.1	9.128735	96.2	13.43974	96.2	23.09231
95.99999237	2.797900165	96.09999084	4.5774	96.2	7.8832	96.3	12.079	96.3	20.5187	95.99999	3.337352	96.1	5.391752	96.2	9.113908	96.3	13.4149	96.3	23.05315
96.09999847	2.792199954	96.19999695	4.5722	96.3	7.873	96.4	12.063	96.4	20.4889	96.1	3.333608	96.2	5.384015	96.3	9.099112	96.4	13.39013	96.4	23.01409
96.20000458	2.785500194	96.30000305	4.5655	96.40001	7.8609	96.50001	12.0473	96.50001	20.4576	96.2	3.329869	96.3	5.37629	96.4	9.084342	96.49999	13.36541	96.49999	22.97512
96.29999542	2.785099806	96.3999939	4.5614	96.5	7.8486	96.6	12.0286	96.6	20.4247	96.3	3.326134	96.4	5.368577	96.49999	9.069601	96.6	13.34075	96.6	22.93624
96.40000153	2.779999878	96.5	4.5555	96.60001	7.8369	96.7	12.0095	96.7	20.3929	96.4	3.322403	96.49999	5.360876	96.6	9.054886	96.7	13.31614	96.7	22.89746
96.49999237	2.776899887	96.59999084	4.5503	96.7	7.825	96.8	11.9925	96.8	20.3609	96.49999	3.318677	96.6	5.353187	96.7	9.040199	96.8	13.2916	96.8	22.85878
96.59999847	2.776299945	96.69999695	4.5474	96.8	7.8155	96.9	11.9736	96.9	20.3271	96.6	3.314954	96.7	5.345509	96.8	9.025542	96.9	13.26711	96.9	22.82018
96.70000458	2.77680037	96.80000305	4.5501	96.90001	7.8034	97.00001	11.9566	97.00001	20.2952	96.7	3.311236	96.8	5.337845	96.9	9.01091	96.99999	13.24268	96.99999	22.78168
96.79999542	2.774999619	96.8999939	4.5481	97	7.7902	97.1	11.945	97.1	20.2702	96.8	3.307523	96.9	5.330191	96.99999	8.996308	97.1	13.2183	97.1	22.74327
96.90000153	2.771699894	97	4.5404	97.10001	7.7846	97.2	11.9321	97.2	20.2447	96.9	3.303814	96.99999	5.32255	97.1	8.981731	97.2	13.19398	97.2	22.70495
96.99999237	2.770399926	97.09999084	4.540199	97.2	7.7776	97.3	11.9159	97.3	20.2104	96.99999	3.300109	97.1	5.31492	97.2	8.967181	97.3	13.16973	97.3	22.66672
97.09999847	2.774199463	97.19999695	4.5355	97.3	7.7643	97.4	11.8936	97.4	20.1778	97.1	3.296409	97.2	5.307302	97.3	8.952661	97.4	13.14552	97.4	22.62858
97.20000458	2.774000163	97.30000305	4.5277	97.40001	7.747	97.50001	11.8772	97.50001	20.1431	97.2	3.292712	97.3	5.299697	97.4	8.938166	97.49999	13.12137	97.49999	22.59054
97.29999542	2.771699827	97.3999939	4.5223	97.5	7.7317	97.6	11.8591	97.6	20.1107	97.3	3.289021	97.4	5.292102	97.49999	8.9237	97.6	13.09728	97.6	22.55259
97.40000153	2.766899944	97.5	4.5175	97.60001	7.7198	97.7	11.8361	97.7	20.0785	97.4	3.285333	97.49999	5.284521	97.6	8.909259	97.7	13.07324	97.7	22.51472
97.49999237	2.758600026	97.59999084	4.5127	97.7	7.7066	97.8	11.8222	97.8	20.0489	97.49999	3.28165	97.6	5.27695	97.7	8.894845	97.8	13.04926	97.8	22.47695
97.59999847	2.752599888	97.69999695	4.505	97.8	7.7008	97.9	11.8117	97.9	20.0213	97.6	3.277971	97.7	5.269391	97.8	8.88046	97.9	13.02534	97.9	22.43926
97.70000458	2.747600467	97.80000305	4.5043	97.90001	7.6974	98.00001	11.7938	98.00001	19.9821	97.7	3.274296	97.8	5.261844	97.9	8.8661	97.99999	13.00147	97.99999	22.40167
97.79999542	2.748499528	97.8999939	4.502399	98	7.6877	98.1	11.7799	98.1	19.9522	97.8	3.270626	97.9	5.254308	97.99999	8.85177	98.1	12.97765	98.1	22.36416
97.90000153	2.748599418	98	4.4943	98.10001	7.6791	98.2	11.7617	98.2	19.9225	97.9	3.26696	97.99999	5.246785	98.1	8.837463	98.2	12.95389	98.2	22.32674
97.99999237	2.745199913	98.09999084	4.492899	98.2	7.6646	98.3	11.7397	98.3	19.8912	97.99999	3.263299	98.1	5.239273	98.2	8.823184	98.3	12.93019	98.3	22.28941
98.09999847	2.742199673	98.19999695	4.49	98.3	7.6492	98.4	11.7223	98.4	19.8614	98.1	3.259641	98.2	5.231772	98.3	8.808933	98.4	12.90654	98.4	22.25216
98.20000458	2.738299957	98.30000305	4.48	98.40001	7.6407	98.50001	11.7061	98.50001	19.8296	98.2	3.255987	98.3	5.224284	98.4	8.794707	98.49999	12.88294	98.49999	22.21501
98.29999542	2.730000038	98.3999939	4.470699	98.5	7.6259	98.6	11.6888	98.6	19.8015	98.3	3.252339	98.4	5.216806	98.49999	8.780509	98.6	12.8594	98.6	22.17794
98.40000153	2.725899701	98.5	4.4624	98.60001	7.6175	98.7	11.6727	98.7	19.7728	98.4	3.248694	98.49999	5.209341	98.6	8.766336	98.7	12.83591	98.7	22.14096
98.49999237	2.71859995	98.59999084	4.459099	98.7	7.6059	98.8	11.6629	98.8	19.7474	98.49999	3.245054	98.6	5.201887	98.7	8.752189	98.8	12.81248	98.8	22.10407
98.59999847	2.715999739	98.69999695	4.4532	98.8	7.5944	98.9	11.6439	98.9	19.7162	98.6	3.241417	98.7	5.194444	98.8	8.738071	98.9	12.78909	98.9	22.06726
98.70000458	2.714000467	98.80000305	4.4499	98.90001	7.5799	99.00001	11.6278	99.00001	19.6861	98.7	3.237785	98.8	5.187014	98.9	8.723976	98.99999	12.76577	98.99999	22.03054
98.79999542	2.714499542	98.8999939	4.4396	99	7.5685	99.1	11.6111	99.1	19.6538	98.8	3.234157	98.9	5.179594	98.99999	8.70991	99.1	12.74249	99.1	21.9939

98.90000153	2.710800002	99	4.4305	99.10001	7.5606	99.2	11.5963	99.2	19.6225	98.9	3.230534	98.99999	5.172186	99.1	8.695869	99.2	12.71927	99.2	21.95734
98.99999237	2.70669994	99.09999084	4.4195	99.2	7.549	99.3	11.5753	99.3	19.5904	98.99999	3.226915	99.1	5.164789	99.2	8.681853	99.3	12.69611	99.3	21.92088
99.09999847	2.706499877	99.19999695	4.4144	99.3	7.53	99.4	11.5591	99.4	19.5593	99.1	3.223299	99.2	5.157404	99.3	8.667865	99.4	12.67299	99.4	21.88449
99.20000458	2.70299981	99.30000305	4.4046	99.40001	7.5155	99.50001	11.5472	99.50001	19.5294	99.2	3.219688	99.3	5.15003	99.4	8.653901	99.49999	12.64993	99.49999	21.8482
99.29999542	2.697300185	99.3999939	4.3985	99.5	7.5041	99.6	11.5335	99.6	19.5002	99.3	3.216082	99.4	5.142668	99.49999	8.639966	99.6	12.62692	99.6	21.81198
99.40000153	2.694699993	99.5	4.3961	99.60001	7.4934	99.7	11.5176	99.7	19.4719	99.4	3.212479	99.49999	5.135317	99.6	8.626054	99.7	12.60396	99.7	21.77585
99.49999237	2.688499873	99.59999084	4.3931	99.7	7.4837	99.8	11.5007	99.8	19.4455	99.49999	3.208881	99.6	5.127977	99.7	8.612168	99.8	12.58106	99.8	21.7398
99.59999847	2.688499773	99.69999695	4.3867	99.8	7.4687	99.9	11.4875	99.9	19.4203	99.6	3.205287	99.7	5.120648	99.8	8.598309	99.9	12.55821	99.9	21.70384
99.70000458	2.685100239	99.80000305	4.382	99.90001	7.4596	100	11.4765	100	19.3959	99.7	3.201697	99.8	5.113332	99.9	8.584474	99.99999	12.53541	99.99999	21.66796
99.79999542	2.68279977	99.8999939	4.379899	100	7.451	100.1	11.4623	100.1	19.3651	99.8	3.198111	99.9	5.106026	99.99999	8.570667	100.1	12.51266	100.1	21.63216
99.90000153	2.684599591	100	4.3752	100.1	7.4422	100.2	11.4469	100.2	19.3379	99.9	3.194529	99.99999	5.098732	100.1	8.556884	100.2	12.48996	100.2	21.59644
99.99999237	2.685399702	100.0999908	4.37	100.2	7.4345	100.3	11.435	100.3	19.3105	99.99999	3.190952	100.1	5.091449	100.2	8.543126	100.3	12.46731	100.3	21.56081
100.0999985	2.682799914	100.1999969	4.364	100.3	7.4235	100.4	11.4231	100.4	19.2875	100.1	3.187379	100.2	5.084176	100.3	8.529395	100.4	12.44472	100.4	21.52525
100.2000046	2.683200264	100.3000031	4.3586	100.4	7.4123	100.5	11.4102	100.5	19.2623	100.2	3.183809	100.3	5.076916	100.4	8.515688	100.5	12.42217	100.5	21.48979
100.2999954	2.68349974	100.3999939	4.350299	100.5	7.4016	100.6	11.3966	100.6	19.2335	100.3	3.180245	100.4	5.069666	100.5	8.502008	100.6	12.39968	100.6	21.45439
100.4000015	2.684699604	100.5	4.346	100.6	7.3936	100.7	11.3789	100.7	19.2079	100.4	3.176684	100.5	5.062428	100.6	8.488351	100.7	12.37724	100.7	21.41908
100.4999924	2.684999666	100.5999908	4.341899	100.7	7.3831	100.8	11.3636	100.8	19.1788	100.5	3.173127	100.6	5.055201	100.7	8.47472	100.8	12.35485	100.8	21.38385
100.5999985	2.680899727	100.6999969	4.3363	100.8	7.3688	100.9	11.3495	100.9	19.1496	100.6	3.169575	100.7	5.047984	100.8	8.461115	100.9	12.3325	100.9	21.3487
100.7000046	2.676499922	100.8000031	4.327	100.9	7.359	101	11.3317	101	19.1213	100.7	3.166026	100.8	5.04078	100.9	8.447534	101	12.31021	101	21.31364
100.7999954	2.671900071	100.8999939	4.327299	101	7.3474	101.1	11.3166	101.1	19.0942	100.8	3.162482	100.9	5.033585	101	8.43398	101.1	12.28797	101.1	21.27864
100.9000015	2.668599682	101	4.321	101.1	7.3416	101.2	11.3004	101.2	19.0653	100.9	3.158942	101	5.026403	101.1	8.420448	101.2	12.26578	101.2	21.24373
100.9999924	2.661399974	101.0999908	4.318199	101.2	7.334	101.3	11.2893	101.3	19.0394	101	3.155406	101.1	5.019231	101.2	8.406942	101.3	12.24364	101.3	21.2089
101.0999985	2.663499723	101.1999969	4.3083	101.3	7.3272	101.4	11.2792	101.4	19.0131	101.1	3.151874	101.2	5.01207	101.3	8.393462	101.4	12.22155	101.4	21.17415
101.2000046	2.658300363	101.3000031	4.3051	101.4	7.3165	101.5	11.2645	101.5	18.9861	101.2	3.148346	101.3	5.004921	101.4	8.380006	101.5	12.1995	101.5	21.13948
101.2999954	2.65869964	101.3999939	4.302599	101.5	7.3086	101.6	11.252	101.6	18.9576	101.3	3.144822	101.4	4.997781	101.5	8.366576	101.6	12.17751	101.6	21.10488
101.4000015	2.65829973	101.5	4.2975	101.6	7.2989	101.7	11.2393	101.7	18.9353	101.4	3.141303	101.5	4.990654	101.6	8.353168	101.7	12.15556	101.7	21.07036
101.4999924	2.653899741	101.5999908	4.2931	101.7	7.2899	101.8	11.2254	101.8	18.9113	101.5	3.137787	101.6	4.983537	101.7	8.339786	101.8	12.13367	101.8	21.03592
101.5999985	2.648999835	101.6999969	4.2898	101.8	7.2786	101.9	11.211	101.9	18.8828	101.6	3.134276	101.7	4.976431	101.8	8.32643	101.9	12.11182	101.9	21.00156
101.7000046	2.645300247	101.8000031	4.2871	101.9	7.2714	102	11.1972	102	18.8596	101.7	3.130768	101.8	4.969336	101.9	8.313096	102	12.09003	102	20.96727
101.7999954	2.646299765	101.8999939	4.2843	102	7.2644	102.1	11.1871	102.1	18.8394	101.8	3.127265	101.9	4.962252	102	8.299789	102.1	12.06828	102.1	20.93306
101.9000015	2.641999832	102	4.2787	102.1	7.2598	102.2	11.1722	102.2	18.8217	101.9	3.123766	102	4.955179	102.1	8.286504	102.2	12.04657	102.2	20.89893
101.9999924	2.640799813	102.0999908	4.280999	102.2	7.2532	102.3	11.1594	102.3	18.7933	102	3.120271	102.1	4.948117	102.2	8.273243	102.3	12.02492	102.3	20.86487
102.0999985	2.642999544	102.1999969	4.2795	102.3	7.2447	102.4	11.1493	102.4	18.7649	102.1	3.11678	102.2	4.941065	102.3	8.260009	102.4	12.00332	102.4	20.83089
102.2000046	2.645300205	102.3000031	4.2745	102.4	7.236	102.5	11.1338	102.5	18.7409	102.2	3.113292	102.3	4.934025	102.4	8.246797	102.5	11.98176	102.5	20.79699
102.2999954	2.644699788	102.3999939	4.2733	102.5	7.2274	102.6	11.1181	102.6	18.715	102.3	3.10981	102.4	4.926994	102.5	8.233612	102.6	11.96025	102.6	20.76316
102.4000015	2.639199753	102.5	4.2708	102.6	7.2201	102.7	11.1047	102.7	18.6922	102.4	3.106331	102.5	4.919976	102.6	8.220448	102.7	11.93879	102.7	20.7294
102.4999924	2.640399715	102.5999908	4.2651	102.7	7.2117	102.8	11.0874	102.8	18.663	102.5	3.102856	102.6	4.912967	102.7	8.207308	102.8	11.91738	102.8	20.69573
102.5999985	2.638599792	102.6999969	4.2592	102.8	7.2023	102.9	11.0721	102.9	18.6326	102.6	3.099385	102.7	4.905969	102.8	8.194195	102.9	11.89601	102.9	20.66212
102.7000046	2.634299843	102.8000031	4.2493	102.9	7.1944	103	11.0555	103	18.6047	102.7	3.095918	102.8	4.898983	102.9	8.181103	103	11.8747	103	20.6286
102.7999954	2.62740015	102.8999939	4.2394	103	7.1858	103.1	11.0395	103.1	18.5792	102.8	3.092455	102.9	4.892006	103	8.168037	103.1	11.85342	103.1	20.59514
102.9000015	2.622799975	103	4.2313	103.1	7.1769	103.2	11.0237	103.2	18.5522	102.9	3.088996	103	4.885042	103.1	8.154993	103.2	11.8322	103.2	20.56176
102.9999924	2.617199992	103.0999908	4.2287	103.2	7.1657	103.3	11.0117	103.3	18.5259	103	3.085542	103.1	4.878086	103.2	8.141973	103.3	11.81102	103.3	20.52846
103.0999985	2.612899748	103.1999969	4.2218	103.3	7.1573	103.4	10.998	103.4	18.5008	103.1	3.082091	103.2	4.871142	103.3	8.128979	103.4	11.78989	103.4	20.49522
103.2000046	2.608200165	103.3000031	4.2188	103.4	7.1491	103.5	10.9846	103.5	18.4719	103.2	3.078644	103.3	4.864209	103.4	8.116006	103.5	11.7688	103.5	20.46206
103.2999954	2.605699832	103.3999939	4.2147	103.5	7.1434	103.6	10.9735	103.6	18.4492	103.3	3.075201	103.4	4.857286	103.5	8.103059	103.6	11.74777	103.6	20.42897
103.4000015	2.604399889	103.5	4.2087	103.6	7.1403	103.7	10.9603	103.7	18.4251	103.4	3.071762	103.5	4.850374	103.6	8.090134	103.7	11.72677	103.7	20.39596
103.4999924	2.601399813	103.5999908	4.206199	103.7	7.1336	103.8	10.946	103.8	18.3956	103.5	3.068328	103.6	4.843472	103.7	8.077232	103.8	11.70583	103.8	20.36302
103.5999985	2.606399541	103.6999969	4.2049	103.8	7.1256	103.9	10.9296	103.9	18.3657	103.6	3.064897	103.7	4.83658	103.8	8.064355	103.9	11.68493	103.9	20.33015
103.7000046	2.607900158	103.8000031	4.2011	103.9	7.1125	104	10.915	104	18.3346	103.7	3.06147	103.8	4.8297	103.9	8.0515	104	11.66407	104	20.29735
103.7999954	2.604499847	103.8999939	4.1916	104	7.0982	104.1	10.901	104.1	18.3091	103.8	3.058047	103.9	4.82283	104	8.038671	104.1	11.64326	104.1	20.26463
103.9000015	2.60239996	104	4.1852	104.1	7.0855	104.2	10.8898	104.2	18.2811	103.9	3.054628	104	4.815971	104.1	8.025862	104.2	11.6225	104.2	20.23197
103.9999924	2.596200039	104.0999908	4.1821	104.2	7.0766	104.3	10.8753	104.3	18.2529	104	3.051214	104.1	4.809121	104.2	8.013077	104.3	11.60178	104.3	20.19939
104.0999985	2.594999884	104.1999969	4.1765	104.3	7.073	104.4	10.8652	104.4	18.2272	104.1	3.047802	104.2	4.802282	104.3	8.000317	104.4	11.58111	104.4	20.16688
104.2000046	2.594200203	104.3000031	4.1702	104.4	7.067	104.5	10.8551	104.5	18.2038	104.2	3.044395	104.3	4.795455	104.4	7.987578	104.5	11.56048	104.5	20.13444
104.2999954	2.5939998	104.3999939	4.167799	104.5	7.0617	104.6	10.8434	104.6	18.1803	104.3	3.040993	104.4	4.788637	104.5	7.974865	104.6	11.5399	104.6	20.10207
104.4000015	2.595399702	104.5	4.1657	104.6	7.0579	104.7	10.8308	104.7	18.1552	104.4	3.037593	104.5	4.78183	104.6	7.962172	104.7	11.51936	104.7	20.06977
104.4999924	2.596299733	104.5999908	4.164399	104.7	7.0487	104.8	10.8162	104.8	18.1297	104.5	3.034198	104.6	4.775033	104.7	7.949502	104.8	11.49887	104.8	20.03754
104.5999985	2.600999573	104.6999969	4.1619	104.8	7.04	104.9	10.8014	104.9	18.105	104.6	3.030807	104.7	4.768246	104.8	7.936858	104.9	11.47842	104.9	20.00538
104.7000046	2.59780011	104.8000031	4.1586	104.9	7.0319	105	10.7926	105	18.083	104.7	3.027419	104.8	4.76147	104.9	7.924234	105	11.45802	105	19.9733
104.7999954	2.593799895	104.8999939	4.1531	105	7.0283	105.1	10.7774	105.1	18.0552	104.8	3.024036	104.9	4.754704	105	7.911635	105.1	11.43766	105.1	19.94127
104.9000015	2.590199939	105	4.1514	105.1	7.0201	105.2	10.7657	105.2	18.0298	104.9	3.020657	105	4.747949	105.1	7.899057	105.2	11.41734	105.2	19.90932
104.9999924	2.59239961	105.0999908	4.1451	105.2	7.0111	105.3	10.7527	105.3	18.0083	105	3.017281	105.1	4.741203	105.2	7.886501	105.3	11.39707	105.3	19.87744
105.0999985	2.592199865	105.1999969	4.1453	105.3	7.0066	105.4	10.7395	105.4	17.9864	105.1	3.01391	105.2	4.734468	105.3	7.873971	105.4	11.37684	105.4	19.84563
105.2000046	2.593300311	105.3000031	4.1429	105.4	7.0033	105.5	10.7282	105.5	17.9662	105.2	3.010542	105.3	4.727744	105.4	7.861461	105.5	11.35666	105.5	19.81388
105.2999954	2.594399706	105.3999939	4.142799	105.5	6.9991	105.6	10.7158	105.6	17.941	105.3	3.007178	105.4	4.721029	105.5	7.848975	105.6	11.33652	105.6	19.7822
105.4000015	2.595599576	105.5	4.1412	105.6	6.9943	105.7	10.703	105.7	17.9129	105.4	3.003818	105.5	4.714326	105.6	7.83651	105.7	11.31642	105.7	19.75059
105.4999924	2.592699594	105.5999908	4.135399	105.7	6.9861	105.8	10.6843	105.8	17.8886	105.5	3.000462	105.6	4.707631	105.7	7.824067	105.8	11.29637	105.8	19.71906
105.5999985	2.593799728	105.6999969	4.1276	105.8	6.9805	105.9	10.6711	105.9	17.8668	105.6	2.99711	105.7	4.700947	105.8	7.811649	105.9	11.27636	105.9	19.68758
105.7000046	2.589600089	105.8000031	4.1235	105.9	6.9724	106	10.6583	106	17.8413	105.7	2.993762	105.8	4.694274	105.9	7.799252	106	11.2564	106	19.65618
105.7999954	2.586799901	105.8999939	4.1166	106	6.9626	106.1	10.6407	106.1	17.8143	105.8	2.990417	105.9	4.68761	106	7.786878	106.1	11.23647	106.1	19.62484
105.9000015	2.585699868	106	4.1114	106.1	6.9499	106.2	10.6272	106.2	17.7934	105.9	2.987077	106	4.680958	106.1	7.774525	106.2	11.21659	106.2	19.59357
105.9999924	2.579199928	106.0999908	4.1029	106.2	6.9431	106.3	10.6156	106.3	17.7727	106	2.98374	106.1	4.674314	106.2	7.762194	106.3	11.19676	106.3	19.56237
106.0999985	2.573599978	106.1999969	4.0961	106.3	6.9351	106.4	10.6045	106.4	17.7509	106.1	2.980407	106.2	4.667681	106.3	7.749887	106.4	11.17696	106.4	19.53123
106.2000046	2.569000188	106.3000031	4.095	106.4	6.9305	106.5	10.5911	106.5	17.7255	106.2	2.977078	106.3	4.661058	106.4	7.737601	106.5	11.15721	106.5	19.50016
106.2999954	2.567299815	106.3999939	4.092499	106.5	6.924	106.6	10.5776	106.6	17.7004	106.3	2.973753	106.4	4.654445	106.5	7.725338	106.6	11.1375	106.6	19.46915
106.4000015	2.567999631	106.5	4.0877	106.6	6.9191	106.7	10.5631	106.7	17.6743	106.4	2.970432	106.5	4.647843	106.6	7.713096	106.7	11.11783	106.7	19.43821
106.4999924	2.564699989	106.5999908	4.0855	106.7	6.9102	106.8	10.5495	106.8	17.65	106.5	2.967115	106.6	4.64125	106.7	7.700875	106.8	11.09821	106.8	19.40734
106.5999985	2.564999844	106.6999969	4.0811	106.8	6.9017	106.9	10.5368	106.9	17.6274	106.6	2.963801	106.7	4.634666	106.8	7.688679	106.9	11.07862	106.9	19.37653
106.7000046	2.564100222	106.8000031	4.0767	106.9	6.8964	107	10.5212	107	17.6015	106.7	2.960491	106.8	4.628094	106.9	7.676502	107	11.05908	107	19.34579
106.7999954	2.562999793	106.8999939	4.073199	107	6.884	107.1	10.5106	107.1	17.5768	106.8	2.957186	106.9	4.621531	107	7.664349	107.1	11.03958	107.1	19.31511
106.9000015	2.563099662	107	4.0634	107.1	6.8772	107.2	10.4933	107.2	17.5465	106.9	2.953884	107	4.614979	107.1	7.652216	107.2	11.02012	107.2	19.28449
106.9999924	2.554500112	107.0999908	4.0603	107.2	6.8654	107.3	10.4814	107.3	17.523	107	2.950586	107.1	4.608436	107.2	7.640105	107.3	11.00071	107.3	19.25395
107.0999985	2.553999748	107.1999969	4.0506	107.3	6.8576	107.4	10.4665	107.4	17.4991	107.1	2.947291	107.2	4.601902	107.3	7.628017	107.4	10.98133	107.4	19.22346
107.2000046	2.547100317	107.3000031	4.0477	107.4	6.847	107.5	10.4517	107.5	17.4741	107.2	2.944	107.3	4.59538	107.4	7.615949	107.5	10.962	107.5	19.19305
107.2999954	2.547099673	107.3999939	4.042	107.5	6.8398	107.6	10.4398	107.6	17.4497	107.3	2.940714	107.4	4.588867	107.5	7.603905	107.6	10.9427	107.6	19.16269
107.4000015	2.543200028	107.5	4.0358	107.6	6.8304	107.7	10.426	107.7	17.4306	107.4	2.93743	107.5	4.582364	107.6	7.59188	107.7	10.92345	107.7	19.1324
107.4999924	2.540400074	107.5999908	4.0328	107.7	6.8237	107.8	10.4091	107.8	17.4043	107.5	2.934152	107.6	4.57587	107.7	7.579877	107.8	10.90424	107.8	19.10217
107.5999985	2.537099988	107.6999969	4.0309	107.8	6.8184	107.9	10.3936	107.9	17.3772	107.6	2.930876	107.7	4.569386	107.8	7.567897	107.9	10.88507	107.9	19.07201
107.7000046	2.533900038	107.8000031	4.0254	107.9	6.809	108	10.3782	108	17.3531	107.7	2.927604	107.8	4.562913	107.9	7.555936	108	10.86594	108	19.04191
107.7999954	2.529899972	107.8999939	4.017	108	6.8013	108.1	10.3694	108.1	17.3349	107.8	2.924337	107.9	4.556449	108	7.543999	108.1	10.84686	108.1	19.01187
107.9000015	2.523500029	108	4.0087	108.1	6.7966	108.2	10.3572	108.2	17.3146	107.9	2.921072	108	4.549996	108.1	7.532081	108.2	10.82781	108.2	18.98189
107.9999924	2.517999953	108.0999908	4.006099	108.2	6.7893	108.3	10.3456	108.3	17.2898	108	2.917812	108.1	4.543551	108.2	7.520185	108.3	10.8088	108.3	18.95198
108.0999985	2.520199607	108.1999969	4.0054	108.3	6.7802	108.4	10.3316	108.4	17.2724	108.1	2.914556	108.2	4.537116	108.3	7.508311	108.4	10.78983	108.4	18.92213
108.2000046	2.519100039	108.3000031	4.0007	108.4	6.7722	108.5	10.3152	108.5	17.2506	108.2	2.911303	108.3	4.530692	108.4	7.496457	108.5	10.77091	108.5	18.89235
108.2999954	2.515499946	108.3999939	3.9974	108.5	6.7643	108.6	10.3071	108.6	17.227	108.3	2.908054	108.4	4.524277	108.5	7.484626	108.6	10.75202	108.6	18.86262
108.4000015	2.512999844	108.5	3.9934	108.6	6.7585	108.7	10.2971	108.7	17.2015	108.4	2.904808	108.5	4.517872	108.6	7.472815	108.7	10.73317	108.7	18.83296
108.4999924	2.513099838	108.5999908	3.9886	108.7	6.7478	108.8	10.2869	108.8	17.1829	108.5	2.901567	108.6	4.511476	108.7	7.461024	108.8	10.71437	108.8	18.80336
108.5999985	2.511399846	108.6999969	3.9873	108.8	6.7408	108.9	10.2787	108.9	17.1627	108.6	2.898329	108.7	4.50509	108.8	7.449256	108.9	10.6956	108.9	18.77382
108.7000046	2.50820022	108.8000031	3.9871	108.9	6.7322	109	10.2717	109	17.1439	108.7	2.895095	108.8	4.498715	108.9	7.437507	109	10.67687	109	18.74434
108.7999954	2.507299791	108.8999939	3.986099	109	6.728	109.1	10.2646	109.1	17.1227	108.8	2.891865	108.9	4.492348	109	7.425781	109.1	10.65818	109.1	18.71492
108.9000015	2.508199693	109	3.9856	109.1	6.72	109.2	10.2555	109.2	17.0982	108.9	2.888638	109	4.485991	109.1	7.414074	109.2	10.63953	109.2	18.68556
108.9999924	2.513999616	109.0999908	3.9826	109.2	6.7138	109.3	10.2477	109.3	17.0791	109	2.885416	109.1	4.479644	109.2	7.402388	109.3	10.62092	109.3	18.65627
109.0999985	2.514699769	109.1999969	3.9793	109.3	6.7098	109.4	10.2375	109.4	17.0627	109.1	2.882197	109.2	4.473306	109.3	7.390724	109.4	10.60235	109.4	18.62703
109.2000046	2.514600389	109.3000031	3.9788	109.4	6.7027	109.5	10.2267	109.5	17.0435	109.2	2.878981	109.3	4.466978	109.4	7.379079	109.5	10.58382	109.5	18.59786
109.2999954	2.516599613	109.3999939	3.978399	109.5	6.696	109.6	10.2175	109.6	17.0221	109.3	2.87577	109.4	4.460659	109.5	7.367457	109.6	10.56533	109.6	18.56874
109.4000015	2.516199684	109.5	3.977	109.6	6.6893	109.7	10.2037	109.7	16.9987	109.4	2.872562	109.5	4.454351	109.6	7.355854	109.7	10.54687	109.7	18.53969
109.4999924	2.511999801	109.5999908	3.9738	109.7	6.6847	109.8	10.1902	109.8	16.9813	109.5	2.869358	109.6	4.448051	109.7	7.344271	109.8	10.52846	109.8	18.5107
109.5999985	2.513299874	109.6999969	3.9706	109.8	6.68	109.9	10.1858	109.9	16.9683	109.6	2.866157	109.7	4.441761	109.8	7.33271	109.9	10.51008	109.9	18.48176
109.7000046	2.511700186	109.8000031	3.9659	109.9	6.6789	110	10.1753	110	16.9447	109.7	2.86296	109.8	4.435481	109.9	7.321168	110	10.49175	110	18.45289
109.7999954	2.509799823	109.8999939	3.962699	110	6.6703	110.1	10.1653	110.1	16.9277	109.8	2.859767	109.9	4.42921	110	7.309649	110.1	10.47345	110.1	18.42407
109.9000015	2.515599474	110	3.9604	110.1	6.6644	110.2	10.1547	110.2	16.9106	109.9	2.856577	110	4.422949	110.1	7.298148	110.2	10.45518	110.2	18.39531
109.9999924	2.513399852	110.0999908	3.959299	110.2	6.6611	110.3	10.1474	110.3	16.8938	110	2.853392	110.1	4.416696	110.2	7.286667	110.3	10.43696	110.3	18.36661
110.0999985	2.513899748	110.1999969	3.9568	110.3	6.6567	110.4	10.1391	110.4	16.8746	110.1	2.850209	110.2	4.410453	110.3	7.275209	110.4	10.41878	110.4	18.33797
110.2000046	2.513700514	110.3000031	3.959	110.4	6.648	110.5	10.1296	110.5	16.8554	110.2	2.847031	110.3	4.404221	110.4	7.263769	110.5	10.40063	110.5	18.30939
110.2999954	2.51879948	110.3999939	3.9579	110.5	6.639	110.6	10.1169	110.6	16.8327	110.3	2.843856	110.4	4.397997	110.5	7.252351	110.6	10.38252	110.6	18.28087

110.4000015	2.517499792	110.5	3.9564	110.6	6.6317	110.7	10.1054	110.7	16.812	110.4	2.840685	110.5	4.391783	110.6	7.240951	110.7	10.36445	110.7	18.2524
110.4999924	2.514499833	110.5999908	3.9519	110.7	6.6257	110.8	10.0921	110.8	16.7921	110.5	2.837518	110.6	4.385578	110.7	7.229572	110.8	10.34641	110.8	18.224
110.5999985	2.510700048	110.6999969	3.9483	110.8	6.6194	110.9	10.0816	110.9	16.7726	110.6	2.834354	110.7	4.379382	110.8	7.218214	110.9	10.32842	110.9	18.19565
110.7000046	2.507400059	110.8000031	3.9413	110.9	6.6153	111	10.069	111	16.7505	110.7	2.831193	110.8	4.373196	110.9	7.206875	111	10.31046	111	18.16736
110.7999954	2.504599936	110.8999939	3.9381	111	6.6099	111.1	10.0565	111.1	16.7304	110.8	2.828037	110.9	4.367019	111	7.195557	111.1	10.29254	111.1	18.13913
110.9000015	2.503799735	111	3.9376	111.1	6.6054	111.2	10.0474	111.2	16.7137	110.9	2.824884	111	4.360852	111.1	7.184258	111.2	10.27465	111.2	18.11095
110.9999924	2.504599799	111.0999908	3.939199	111.2	6.5989	111.3	10.0353	111.3	16.6942	111	2.821735	111.1	4.354693	111.2	7.172979	111.3	10.25681	111.3	18.08283
111.0999985	2.505099725	111.1999969	3.9427	111.3	6.5952	111.4	10.0205	111.4	16.6663	111.1	2.818589	111.2	4.348544	111.3	7.161721	111.4	10.239	111.4	18.05477
111.2000046	2.507500253	111.3000031	3.9392	111.4	6.5822	111.5	10.0077	111.5	16.6449	111.2	2.815447	111.3	4.342404	111.4	7.150481	111.5	10.22122	111.5	18.02677
111.2999954	2.50509977	111.3999939	3.9307	111.5	6.5735	111.6	9.9976	111.6	16.6226	111.3	2.812309	111.4	4.336273	111.5	7.139263	111.6	10.20349	111.6	17.99882
111.4000015	2.49680017	111.5	3.9255	111.6	6.5652	111.7	9.9818	111.7	16.604	111.4	2.809174	111.5	4.330153	111.6	7.128063	111.7	10.18579	111.7	17.97093
111.4999924	2.492599972	111.5999908	3.9226	111.7	6.5578	111.8	9.9692	111.8	16.5852	111.5	2.806043	111.6	4.32404	111.7	7.116882	111.8	10.16812	111.8	17.9431
111.5999985	2.490599926	111.6999969	3.9218	111.8	6.5511	111.9	9.9617	111.9	16.5624	111.6	2.802916	111.7	4.317937	111.8	7.105723	111.9	10.1505	111.9	17.91532
111.7000046	2.486900264	111.8000031	3.9212	111.9	6.5476	112	9.9501	112	16.5387	111.7	2.799792	111.8	4.311844	111.9	7.094582	112	10.13291	112	17.8876
111.7999954	2.485399739	111.8999939	3.9183	112	6.5435	112.1	9.9377	112.1	16.5182	111.8	2.796671	111.9	4.305759	112	7.083462	112.1	10.11535	112.1	17.85993
111.9000015	2.482999839	112	3.9188	112.1	6.5371	112.2	9.9259	112.2	16.4966	111.9	2.793555	112	4.299684	112.1	7.07236	112.2	10.09783	112.2	17.83231
111.9999924	2.48219983	112.0999908	3.9155	112.2	6.5321	112.3	9.9159	112.3	16.4728	112	2.790442	112.1	4.293618	112.2	7.061278	112.3	10.08036	112.3	17.80476
112.0999985	2.478899936	112.1999969	3.9153	112.3	6.5251	112.4	9.9015	112.4	16.4498	112.1	2.787332	112.2	4.28756	112.3	7.050217	112.4	10.06291	112.4	17.77726
112.2000046	2.477100205	112.3000031	3.9146	112.4	6.5198	112.5	9.8916	112.5	16.4275	112.2	2.784226	112.3	4.281513	112.4	7.039173	112.5	10.0455	112.5	17.74982
112.2999954	2.479699812	112.3999939	3.91	112.5	6.5141	112.6	9.8848	112.6	16.4084	112.3	2.781124	112.4	4.275474	112.5	7.028151	112.6	10.02813	112.6	17.72243
112.4000015	2.477899908	112.5	3.9075	112.6	6.5104	112.7	9.873	112.7	16.3819	112.4	2.778025	112.5	4.269444	112.6	7.017146	112.7	10.01079	112.7	17.69509
112.4999924	2.473899948	112.5999908	3.907899	112.7	6.4998	112.8	9.8641	112.8	16.3603	112.5	2.77493	112.6	4.263423	112.7	7.00616	112.8	9.993487	112.8	17.66781
112.5999985	2.473799738	112.6999969	3.8998	112.8	6.4919	112.9	9.8537	112.9	16.3382	112.6	2.771838	112.7	4.257411	112.8	6.995196	112.9	9.976219	112.9	17.64058
112.7000046	2.468200125	112.8000031	3.8929	112.9	6.483	113	9.8402	113	16.3155	112.7	2.76875	112.8	4.251409	112.9	6.984249	113	9.958989	113	17.61341
112.7999954	2.463499897	112.8999939	3.8868	113	6.4741	113.1	9.8255	113.1	16.2957	112.8	2.765665	112.9	4.245415	113	6.973323	113.1	9.941792	113.1	17.5863
112.9000015	2.460099953	113	3.8826	113.1	6.469	113.2	9.8133	113.2	16.2767	112.9	2.762584	113	4.239431	113.1	6.962414	113.2	9.924631	113.2	17.55923
112.9999924	2.457500039	113.0999908	3.8806	113.2	6.4627	113.3	9.8035	113.3	16.256	113	2.759507	113.1	4.233455	113.2	6.951525	113.3	9.907507	113.3	17.53222
113.0999985	2.458699881	113.1999969	3.8766	113.3	6.4532	113.4	9.7953	113.4	16.2356	113.1	2.756433	113.2	4.227488	113.3	6.940656	113.4	9.890415	113.4	17.50527
113.2000046	2.458600163	113.3000031	3.8731	113.4	6.448	113.5	9.7851	113.5	16.2181	113.2	2.753362	113.3	4.221531	113.4	6.929804	113.5	9.873361	113.5	17.47837
113.2999954	2.456599866	113.3999939	3.8729	113.5	6.4442	113.6	9.7747	113.6	16.2009	113.3	2.750296	113.4	4.215582	113.5	6.918973	113.6	9.85634	113.6	17.45152
113.4000015	2.453699856	113.5	3.8717	113.6	6.4381	113.7	9.7608	113.7	16.1815	113.4	2.747232	113.5	4.209642	113.6	6.90816	113.7	9.839353	113.7	17.42472
113.4999924	2.453599702	113.5999908	3.868999	113.7	6.4285	113.8	9.7508	113.8	16.1612	113.5	2.744173	113.6	4.203711	113.7	6.897365	113.8	9.822403	113.8	17.39798
113.5999985	2.45269971	113.6999969	3.8623	113.8	6.4241	113.9	9.7403	113.9	16.1398	113.6	2.741116	113.7	4.197789	113.8	6.886591	113.9	9.805486	113.9	17.37129
113.7000046	2.450900208	113.8000031	3.8609	113.9	6.4155	114	9.731	114	16.1222	113.7	2.738064	113.8	4.191876	113.9	6.875833	114	9.788605	114	17.34466
113.7999954	2.451599805	113.8999939	3.86	114	6.41	114.1	9.7186	114.1	16.1061	113.8	2.735015	113.9	4.185971	114	6.865097	114.1	9.771757	114.1	17.31807
113.9000015	2.450199901	114	3.8611	114.1	6.3999	114.2	9.705	114.2	16.0858	113.9	2.731969	114	4.180076	114.1	6.854377	114.2	9.754942	114.2	17.29154
113.9999924	2.45069977	114.0999908	3.8587	114.2	6.3918	114.3	9.6916	114.3	16.0612	114	2.728927	114.1	4.174189	114.2	6.843676	114.3	9.738165	114.3	17.26506
114.0999985	2.45229982	114.1999969	3.8555	114.3	6.3834	114.4	9.6823	114.4	16.0392	114.1	2.725888	114.2	4.168311	114.3	6.832995	114.4	9.721419	114.4	17.23864
114.2000046	2.446799863	114.3000031	3.848	114.4	6.3798	114.5	9.6712	114.5	16.0164	114.2	2.722852	114.3	4.162443	114.4	6.822332	114.5	9.70471	114.5	17.21227
114.2999954	2.442500144	114.3999939	3.844999	114.5	6.3749	114.6	9.6607	114.6	15.9929	114.3	2.719821	114.4	4.156582	114.5	6.811688	114.6	9.688032	114.6	17.18594
114.4000015	2.444799728	114.5	3.8404	114.6	6.368	114.7	9.6469	114.7	15.9722	114.4	2.716793	114.5	4.150731	114.6	6.801061	114.7	9.671388	114.7	17.15967
114.4999924	2.446399821	114.5999908	3.8345	114.7	6.3606	114.8	9.6361	114.8	15.9587	114.5	2.713768	114.6	4.144888	114.7	6.790453	114.8	9.65478	114.8	17.13345
114.5999985	2.442700053	114.6999969	3.8323	114.8	6.3564	114.9	9.6245	114.9	15.9356	114.6	2.710747	114.7	4.139054	114.8	6.779865	114.9	9.638203	114.9	17.10728
114.7000046	2.438600269	114.8000031	3.8297	114.9	6.347	115	9.6119	115	15.9116	114.7	2.707729	114.8	4.133229	114.9	6.769294	115	9.621663	115	17.08117
114.7999954	2.441899717	114.8999939	3.8244	115	6.3394	115.1	9.5951	115.1	15.8876	114.8	2.704715	114.9	4.127413	115	6.758743	115.1	9.605153	115.1	17.05511
114.9000015	2.440199926	115	3.8191	115.1	6.3252	115.2	9.5826	115.2	15.8695	114.9	2.701704	115	4.121605	115.1	6.748208	115.2	9.588677	115.2	17.02909
114.9999924	2.436400012	115.0999908	3.812101	115.2	6.3199	115.3	9.5697	115.3	15.8514	115	2.698696	115.1	4.115806	115.2	6.737692	115.3	9.572237	115.3	17.00313
115.0999985	2.424300355	115.1999969	3.8083	115.3	6.3152	115.4	9.5527	115.4	15.8285	115.1	2.695692	115.2	4.110015	115.3	6.727196	115.4	9.555827	115.4	16.97722
115.2000046	2.42310019	115.3000031	3.8091	115.4	6.3065	115.5	9.5399	115.5	15.8039	115.2	2.692692	115.3	4.104234	115.4	6.716716	115.5	9.539454	115.5	16.95136
115.2999954	2.42369983	115.3999939	3.8022	115.5	6.2971	115.6	9.529	115.6	15.7828	115.3	2.689695	115.4	4.098461	115.5	6.706256	115.6	9.52311	115.6	16.92555
115.4000015	2.416300253	115.5	3.7922	115.6	6.2902	115.7	9.52	115.7	15.7649	115.4	2.686701	115.5	4.092697	115.6	6.695813	115.7	9.5068	115.7	16.89978
115.4999924	2.411100159	115.5999908	3.7862	115.7	6.2842	115.8	9.5088	115.8	15.7485	115.5	2.683711	115.6	4.086941	115.7	6.685388	115.8	9.490525	115.8	16.87408
115.5999985	2.408299961	115.6999969	3.7862	115.8	6.2732	115.9	9.5009	115.9	15.727	115.6	2.680724	115.7	4.081193	115.8	6.674982	115.9	9.474281	115.9	16.84841
115.7000046	2.403600032	115.8000031	3.7817	115.9	6.2666	116	9.4861	116	15.7035	115.7	2.677741	115.8	4.075455	115.9	6.664593	116	9.458071	116	16.82281
115.7999954	2.397399989	115.8999939	3.7779	116	6.2587	116.1	9.4724	116.1	15.688	115.8	2.674761	115.9	4.069724	116	6.654223	116.1	9.441892	116.1	16.79724
115.9000015	2.396399006	116	3.7731	116.1	6.2539	116.2	9.4579	116.2	15.6661	115.9	2.671785	116	4.064004	116.1	6.64387	116.2	9.425745	116.2	16.77173
115.9999924	2.392100134	116.0999908	3.7677	116.2	6.2483	116.3	9.4453	116.3	15.6464	116	2.668812	116.1	4.05829	116.2	6.633535	116.3	9.409634	116.3	16.74627
116.0999985	2.390299916	116.1999969	3.7618	116.3	6.241	116.4	9.4283	116.4	15.6215	116.1	2.665842	116.2	4.052585	116.3	6.623219	116.4	9.393552	116.4	16.72086
116.2000046	2.390000085	116.3000031	3.7585	116.4	6.232	116.5	9.4162	116.5	15.5996	116.2	2.662876	116.3	4.04689	116.4	6.61292	116.5	9.377505	116.5	16.6955
116.2999954	2.385699953	116.3999939	3.749	116.5	6.2253	116.6	9.4038	116.6	15.5787	116.3	2.659913	116.4	4.041202	116.5	6.60264	116.6	9.361487	116.6	16.67019
116.4000015	2.378900257	116.5	3.7432	116.6	6.2174	116.7	9.3949	116.7	15.5598	116.4	2.656954	116.5	4.035524	116.6	6.592376	116.7	9.345502	116.7	16.64492
116.4999924	2.376099982	116.5999908	3.733	116.7	6.2061	116.8	9.382	116.8	15.5396	116.5	2.653998	116.6	4.029853	116.7	6.58213	116.8	9.329552	116.8	16.61971
116.5999985	2.374699972	116.6999969	3.7311	116.8	6.1959	116.9	9.3715	116.9	15.5181	116.6	2.651045	116.7	4.024191	116.8	6.571903	116.9	9.31363	116.9	16.59454
116.7000046	2.372600019	116.8000031	3.7277	116.9	6.186	117	9.3594	117	15.4992	116.7	2.648096	116.8	4.018538	116.9	6.561692	117	9.297743	117	16.56942
116.7999954	2.369299976	116.8999939	3.7268	117	6.1766	117.1	9.3535	117.1	15.4812	116.8	2.64515	116.9	4.012892	117	6.5515	117.1	9.281886	117.1	16.54435
116.9000015	2.369499946	117	3.722	117.1	6.1713	117.2	9.3456	117.2	15.4596	116.9	2.642208	117	4.007256	117.1	6.541325	117.2	9.26606	117.2	16.51933
116.9999924	2.36659996	117.0999908	3.7175	117.2	6.1605	117.3	9.3387	117.3	15.4408	117	2.639269	117.1	4.001628	117.2	6.531166	117.3	9.250269	117.3	16.49436
117.0999985	2.366599832	117.1999969	3.7087	117.3	6.1529	117.4	9.3321	117.4	15.4224	117.1	2.636333	117.2	3.996007	117.3	6.521027	117.4	9.234506	117.4	16.46943
117.2000046	2.362100133	117.3000031	3.7003	117.4	6.1448	117.5	9.3216	117.5	15.4054	117.2	2.6334	117.3	3.990396	117.4	6.510904	117.5	9.218777	117.5	16.44455
117.2999954	2.358099835	117.3999939	3.6984	117.5	6.1414	117.6	9.3129	117.6	15.3865	117.3	2.630472	117.4	3.984793	117.5	6.5008	117.6	9.203078	117.6	16.41972
117.4000015	2.353000041	117.5	3.6956	117.6	6.1341	117.7	9.302	117.7	15.3724	117.4	2.627546	117.5	3.979199	117.6	6.490712	117.7	9.187409	117.7	16.39494
117.4999924	2.351799915	117.5999908	3.6927	117.7	6.1272	117.8	9.29	117.8	15.3475	117.5	2.624624	117.6	3.973612	117.7	6.480641	117.8	9.171774	117.8	16.37021
117.5999985	2.351999919	117.6999969	3.6918	117.8	6.114	117.9	9.2792	117.9	15.3234	117.6	2.621705	117.7	3.968033	117.8	6.47059	117.9	9.156168	117.9	16.34552
117.7000046	2.350599804	117.8000031	3.6837	117.9	6.106	118	9.2686	118	15.3027	117.7	2.618789	117.8	3.962464	117.9	6.460553	118	9.140596	118	16.32088
117.7999954	2.347200181	117.8999939	3.6804	118	6.0925	118.1	9.2578	118.1	15.2847	117.8	2.615877	117.9	3.956902	118	6.450536	118.1	9.125052	118.1	16.29628
117.9000015	2.343800111	118	3.6757	118.1	6.081	118.2	9.252501	118.2	15.269	117.9	2.612968	118	3.951349	118.1	6.440535	118.2	9.109539	118.2	16.27174
117.9999924	2.339600117	118.0999908	3.6697	118.2	6.0784	118.3	9.249999	118.3	15.2574	118	2.610063	118.1	3.945804	118.2	6.43055	118.3	9.094059	118.3	16.24724
118.0999985	2.332300044	118.1999969	3.6701	118.3	6.0799	118.4	9.2429	118.4	15.2438	118.1	2.607161	118.2	3.940267	118.3	6.420585	118.4	9.078607	118.4	16.22279
118.2000046	2.335000661	118.3000031	3.6735	118.4	6.0769	118.5	9.2336	118.5	15.2285	118.2	2.604262	118.3	3.934739	118.4	6.410635	118.5	9.063189	118.5	16.19838
118.2999954	2.337199372	118.3999939	3.672399	118.5	6.0702	118.6	9.2219	118.6	15.213	118.3	2.601367	118.4	3.929218	118.5	6.400703	118.6	9.047798	118.6	16.17402
118.4000015	2.338099721	118.5	3.6691	118.6	6.0623	118.7	9.2107	118.7	15.1914	118.4	2.598474	118.5	3.923707	118.6	6.390787	118.7	9.032439	118.7	16.14971
118.4999924	2.338199822	118.5999908	3.6663	118.7	6.0541	118.8	9.2001	118.8	15.1777	118.5	2.595586	118.6	3.918202	118.7	6.380888	118.8	9.017112	118.8	16.12544
118.5999985	2.338199928	118.6999969	3.6612	118.8	6.0507	118.9	9.1868	118.9	15.162	118.6	2.5927	118.7	3.912706	118.8	6.371008	118.9	9.001813	118.9	16.10122
118.7000046	2.33520021	118.8000031	3.6581	118.9	6.0454	119	9.1772	119	15.1468	118.7	2.589818	118.8	3.907219	118.9	6.361143	119	8.986547	119	16.07705
118.7999954	2.336099781	118.8999939	3.6525	119	6.0433	119.1	9.1727	119.1	15.1345	118.8	2.586939	118.9	3.90174	119	6.351296	119.1	8.971308	119.1	16.05292
118.9000015	2.334799966	119	3.6522	119.1	6.0418	119.2	9.1695	119.2	15.1172	118.9	2.584063	119	3.896269	119.1	6.341465	119.2	8.9561	119.2	16.02883
118.9999924	2.329999775	119.0999908	3.655399	119.2	6.0372	119.3	9.1584	119.3	15.0988	119	2.581191	119.1	3.890805	119.2	6.33165	119.3	8.940924	119.3	16.0048
119.0999985	2.331699608	119.1999969	3.6512	119.3	6.0317	119.4	9.1506	119.4	15.0842	119.1	2.578322	119.2	3.88535	119.3	6.321854	119.4	8.925775	119.4	15.98081
119.2000046	2.331800056	119.3000031	3.6466	119.4	6.0304	119.5	9.1372	119.5	15.0687	119.2	2.575456	119.3	3.879904	119.4	6.312073	119.5	8.910659	119.5	15.95686
119.2999954	2.32649996	119.3999939	3.6445	119.5	6.0228	119.6	9.1277	119.6	15.0495	119.3	2.572594	119.4	3.874465	119.5	6.302311	119.6	8.895571	119.6	15.93296
119.4000015	2.322999878	119.5	3.6378	119.6	6.0159	119.7	9.1169	119.7	15.0293	119.4	2.569734	119.5	3.869034	119.6	6.292564	119.7	8.880512	119.7	15.9091
119.4999924	2.32000008	119.5999908	3.6315	119.7	6.0087	119.8	9.1106	119.8	15.0142	119.5	2.566879	119.6	3.863611	119.7	6.282833	119.8	8.865485	119.8	15.88529
119.5999985	2.317400076	119.6999969	3.6293	119.8	6.0059	119.9	9.1014	119.9	14.996	119.6	2.564026	119.7	3.858197	119.8	6.27312	119.9	8.850486	119.9	15.86152
119.7000046	2.316000288	119.8000031	3.6314	119.9	5.9972	120	9.0947	120	14.9837	119.7	2.561176	119.8	3.85279	119.9	6.263423	120	8.835518	120	15.8378
119.7999954	2.319199683	119.8999939	3.6292	120	5.9947	120.1	9.0866	120.1	14.9687	119.8	2.55833	119.9	3.847392	120	6.253743	120.1	8.820577	120.1	15.81413
119.9000015	2.316500018	120	3.6224	120.1	5.9933	120.2	9.0793	120.2	14.9484	119.9	2.555487	120	3.842002	120.1	6.244079	120.2	8.805666	120.2	15.79049
119.9999924	2.3187998	120.0999908	3.6177	120.2	5.9868	120.3	9.0739	120.3	14.9298	120	2.552648	120.1	3.836619	120.2	6.234431	120.3	8.790786	120.3	15.76691
120.0999985	2.319099851	120.1999969	3.6141	120.3	5.9819	120.4	9.0708	120.4	14.9175	120.1	2.549811	120.2	3.831244	120.3	6.224801	120.4	8.775934	120.4	15.74336
120.2000046	2.319800172	120.3000031	3.6093	120.4	5.9771	120.5	9.0581	120.5	14.8985	120.2	2.546978	120.3	3.825878	120.4	6.215187	120.5	8.761112	120.5	15.71986
120.2999954	2.321599829	120.3999939	3.610199	120.5	5.9689	120.6	9.0492	120.6	14.8805	120.3	2.544149	120.4	3.820519	120.5	6.205589	120.6	8.746318	120.6	15.69641
120.4000015	2.325399609	120.5	3.6091	120.6	5.9595	120.7	9.0412	120.7	14.8659	120.4	2.541322	120.5	3.815169	120.6	6.196007	120.7	8.731553	120.7	15.67299
120.4999924	2.320100092	120.5999908	3.6047	120.7	5.9539	120.8	9.0292	120.8	14.8492	120.5	2.538499	120.6	3.809826	120.7	6.186442	120.8	8.716818	120.8	15.64963
120.5999985	2.314900035	120.6999969	3.6024	120.8	5.9506	120.9	9.0148	120.9	14.8281	120.6	2.535678	120.7	3.80449	120.8	6.176894	120.9	8.702111	120.9	15.6263
120.7000046	2.313700049	120.8000031	3.5977	120.9	5.9407	121	9.0031	121	14.8112	120.7	2.532861	120.8	3.799164	120.9	6.16736	121	8.687434	121	15.60303
120.7999954	2.31369997	120.8999939	3.5915	121	5.937	121.1	8.9954	121.1	14.7986	120.8	2.530048	120.9	3.793845	121	6.157845	121.1	8.672784	121.1	15.57979
120.9000015	2.307700112	121	3.5847	121.1	5.932	121.2	8.9889	121.2	14.7887	120.9	2.527237	121	3.788534	121.1	6.148344	121.2	8.658162	121.2	15.55659
120.9999924	2.306500009	121.0999908	3.583	121.2	5.9302	121.3	8.9776	121.3	14.7734	121	2.52443	121.1	3.78323	121.2	6.13886	121.3	8.643572	121.3	15.53344
121.0999985	2.309399749	121.1999969	3.5839	121.3	5.9246	121.4	8.965	121.4	14.755	121.1	2.521626	121.2	3.777935	121.3	6.129393	121.4	8.629007	121.4	15.51034
121.2000046	2.314400218	121.3000031	3.5828	121.4	5.9183	121.5	8.9584	121.5	14.7368	121.2	2.518825	121.3	3.772647	121.4	6.11994	121.5	8.614474	121.5	15.48727
121.2999954	2.313099789	121.3999939	3.5738	121.5	5.9109	121.6	8.9506	121.6	14.7213	121.3	2.516028	121.4	3.767367	121.5	6.110506	121.6	8.599966	121.6	15.46425
121.4000015	2.307699884	121.5	3.5711	121.6	5.9097	121.7	8.9417	121.7	14.7054	121.4	2.513233	121.5	3.762096	121.6	6.101085	121.7	8.585487	121.7	15.44127
121.4999924	2.307099875	121.5999908	3.572199	121.7	5.903	121.8	8.9331	121.8	14.6912	121.5	2.510442	121.6	3.756831	121.7	6.091681	121.8	8.571038	121.8	15.41834
121.5999985	2.308199724	121.6999969	3.5701	121.8	5.8951	121.9	8.9272	121.9	14.6744	121.6	2.507654	121.7	3.751575	121.8	6.082294	121.9	8.556615	121.9	15.39544
121.7000046	2.305400059	121.8000031	3.5667	121.9	5.8941	122	8.9217	122	14.6606	121.7	2.504869	121.8	3.746327	121.9	6.072922	122	8.542222	122	15.37259
121.7999954	2.304599927	121.8999939	3.566	122	5.8894	122.1	8.9148	122.1	14.6476	121.8	2.502088	121.9	3.741085	122	6.063567	122.1	8.527855	122.1	15.34979

121.9000015	2.307699822	122	3.5627	122.1	5.8824	122.2	8.9114	122.2	14.6318	121.9	2.499309	122	3.735853	122.1	6.054226	122.2	8.513516	122.2	15.32702
121.9999924	2.307299892	122.0999908	3.5615	122.2	5.8792	122.3	8.9049	122.3	14.616	122	2.496534	122.1	3.730627	122.2	6.044902	122.3	8.499207	122.3	15.3043
122.0999985	2.303999787	122.1999969	3.5622	122.3	5.8702	122.4	8.8975	122.4	14.6017	122.1	2.493762	122.2	3.725409	122.3	6.035594	122.4	8.484924	122.4	15.28161
122.2000046	2.300699983	122.3000031	3.5567	122.4	5.8657	122.5	8.8941	122.5	14.591	122.2	2.490993	122.3	3.7202	122.4	6.026301	122.5	8.470671	122.5	15.25897
122.2999954	2.296600053	122.3999939	3.5543	122.5	5.8651	122.6	8.8866	122.6	14.5759	122.3	2.488227	122.4	3.714997	122.5	6.017026	122.6	8.456443	122.6	15.23638
122.4000015	2.297699795	122.5	3.5585	122.6	5.8588	122.7	8.8798	122.7	14.5634	122.4	2.485464	122.5	3.709803	122.6	6.007764	122.7	8.442243	122.7	15.21382
122.4999924	2.30229945	122.5999908	3.5556	122.7	5.8586	122.8	8.8706	122.8	14.5468	122.5	2.482705	122.6	3.704616	122.7	5.998518	122.8	8.428072	122.8	15.1913
122.5999985	2.300799894	122.6999969	3.558	122.8	5.8534	122.9	8.8607	122.9	14.5291	122.6	2.479949	122.7	3.699437	122.8	5.98929	122.9	8.413927	122.9	15.16883
122.7000046	2.303300111	122.8000031	3.5518	122.9	5.8493	123	8.8529	123	14.5128	122.7	2.477195	122.8	3.694266	122.9	5.980075	123	8.399811	123	15.1464
122.7999954	2.302699856	122.8999939	3.5476	123	5.8463	123.1	8.8446	123.1	14.4934	122.8	2.474446	122.9	3.689101	123	5.970877	123.1	8.385721	123.1	15.12401
122.9000015	2.299999893	123	3.5414	123.1	5.8421	123.2	8.8357	123.2	14.4775	122.9	2.471699	123	3.683946	123.1	5.961694	123.2	8.371658	123.2	15.10166
122.9999924	2.29709993	123.0999908	3.5371	123.2	5.8382	123.3	8.828	123.3	14.4646	123	2.468955	123.1	3.678797	123.2	5.952526	123.3	8.357624	123.3	15.07935
123.0999985	2.294100053	123.1999969	3.5329	123.3	5.8335	123.4	8.8153	123.4	14.4469	123.1	2.466214	123.2	3.673655	123.3	5.943376	123.4	8.343615	123.4	15.05708
123.2000046	2.290500189	123.3000031	3.5331	123.4	5.8275	123.5	8.807	123.5	14.4316	123.2	2.463477	123.3	3.668522	123.4	5.934239	123.5	8.329635	123.5	15.03486
123.2999954	2.288899812	123.3999939	3.5287	123.5	5.8237	123.6	8.8006	123.6	14.4161	123.3	2.460743	123.4	3.663396	123.5	5.925119	123.6	8.31568	123.6	15.01267
123.4000015	2.283900126	123.5	3.5249	123.6	5.8204	123.7	8.7926	123.7	14.4054	123.4	2.458011	123.5	3.658278	123.6	5.916013	123.7	8.301753	123.7	14.99052
123.4999924	2.283399862	123.5999908	3.5264	123.7	5.821	123.8	8.7854	123.8	14.3887	123.5	2.455284	123.6	3.653167	123.7	5.906922	123.8	8.287854	123.8	14.96842
123.5999985	2.28319981	123.6999969	3.5247	123.8	5.818	123.9	8.7781	123.9	14.3752	123.6	2.452559	123.7	3.648063	123.8	5.897848	123.9	8.273979	123.9	14.94636
123.7000046	2.281899953	123.8000031	3.5177	123.9	5.8174	124	8.773401	124	14.3642	123.7	2.449837	123.8	3.642968	123.9	5.888788	124	8.260134	124	14.92433
123.7999954	2.279800049	123.8999939	3.5143	124	5.8168	124.1	8.7681	124.1	14.354	123.8	2.447118	123.9	3.63788	124	5.879745	124.1	8.246313	124.1	14.90235
123.9000015	2.278399861	124	3.5083	124.1	5.8169	124.2	8.7634	124.2	14.3411	123.9	2.444402	124	3.632799	124.1	5.870716	124.2	8.232519	124.2	14.88041
123.9999924	2.278499727	124.0999908	3.508899	124.2	5.8176	124.3	8.7511	124.3	14.3269	124	2.44169	124.1	3.627726	124.2	5.861701	124.3	8.218753	124.3	14.8585
124.0999985	2.283899504	124.1999969	3.5089	124.3	5.8113	124.4	8.7427	124.4	14.3115	124.1	2.43898	124.2	3.62266	124.3	5.852704	124.4	8.205012	124.4	14.83664
124.2000046	2.285699989	124.3000031	3.5013	124.4	5.8014	124.5	8.7302	124.5	14.2932	124.2	2.436274	124.3	3.617602	124.4	5.84372	124.5	8.191299	124.5	14.81482
124.2999954	2.282199989	124.3999939	3.4973	124.5	5.7911	124.6	8.7225	124.6	14.2758	124.3	2.433571	124.4	3.612551	124.5	5.834753	124.6	8.177611	124.6	14.79303
124.4000015	2.283600042	124.5	3.4913	124.6	5.7864	124.7	8.7128	124.7	14.257	124.4	2.430871	124.5	3.607508	124.6	5.825799	124.7	8.163949	124.7	14.77129
124.4999924	2.27770016	124.5999908	3.4888	124.7	5.7827	124.8	8.7059	124.8	14.2452	124.5	2.428174	124.6	3.602472	124.7	5.816861	124.8	8.150315	124.8	14.74958
124.5999985	2.277999935	124.6999969	3.4839	124.8	5.7812	124.9	8.7014	124.9	14.2281	124.6	2.42548	124.7	3.597443	124.8	5.807939	124.9	8.136705	124.9	14.72792
124.7000046	2.275300174	124.8000031	3.485	124.9	5.7747	125	8.6978	125	14.2141	124.7	2.422789	124.8	3.592422	124.9	5.79903	125	8.123124	125	14.70629
124.7999954	2.277599866	124.8999939	3.4849	125	5.7686	125.1	8.6926	125.1	14.2021	124.8	2.420101	124.9	3.587408	125	5.790138	125.1	8.109566	125.1	14.68471
124.9000015	2.276999851	125	3.4882	125.1	5.7637	125.2	8.6808	125.2	14.1823	124.9	2.417416	125	3.582402	125.1	5.78126	125.2	8.096034	125.2	14.66316
124.9999924	2.278599733	125.0999908	3.4855	125.2	5.758	125.3	8.6713	125.3	14.1658	125	2.414735	125.1	3.577403	125.2	5.772396	125.3	8.08253	125.3	14.64165
125.0999985	2.282699833	125.1999969	3.4769	125.3	5.7532	125.4	8.6633	125.4	14.1484	125.1	2.412056	125.2	3.572411	125.3	5.763549	125.4	8.069051	125.4	14.62018
125.2000046	2.277200139	125.3000031	3.4784	125.4	5.745	125.5	8.6546	125.5	14.1313	125.2	2.40938	125.3	3.567427	125.4	5.754715	125.5	8.055598	125.5	14.59875
125.2999954	2.275699859	125.3999939	3.4756	125.5	5.7374	125.6	8.6497	125.6	14.1118	125.3	2.406708	125.4	3.56245	125.5	5.745897	125.6	8.04217	125.6	14.57736
125.4000015	2.276700011	125.5	3.475	125.6	5.7325	125.7	8.6411	125.7	14.098	125.4	2.404038	125.5	3.557481	125.6	5.737093	125.7	8.028767	125.7	14.556
125.4999924	2.276999902	125.5999908	3.4737	125.7	5.7265	125.8	8.6347	125.8	14.0855	125.5	2.401372	125.6	3.552518	125.7	5.728304	125.8	8.015392	125.8	14.53469
125.5999985	2.280799807	125.6999969	3.4764	125.8	5.7236	125.9	8.628	125.9	14.0671	125.6	2.398709	125.7	3.547563	125.8	5.719531	125.9	8.00204	125.9	14.51341
125.7000046	2.281500057	125.8000031	3.4724	125.9	5.718	126	8.6184	126	14.0476	125.7	2.396048	125.8	3.542616	125.9	5.710771	126	7.988716	126	14.49218
125.7999954	2.280199908	125.8999939	3.4684	126	5.7122	126.1	8.6088	126.1	14.0283	125.8	2.393391	125.9	3.537675	126	5.702027	126.1	7.975416	126.1	14.47098
125.9000015	2.277699948	126	3.461	126.1	5.7005	126.2	8.6007	126.2	14.0098	125.9	2.390737	126	3.532742	126.1	5.693297	126.2	7.96214	126.2	14.44981
125.9999924	2.275700064	126.0999908	3.4584	126.2	5.6923	126.3	8.5938	126.3	13.9955	126	2.388086	126.1	3.527816	126.2	5.684581	126.3	7.948892	126.3	14.42869
126.0999985	2.270200195	126.1999969	3.455	126.3	5.6899	126.4	8.5865	126.4	13.9852	126.1	2.385437	126.2	3.522897	126.3	5.675881	126.4	7.935667	126.4	14.4076
126.2000046	2.267400092	126.3000031	3.4552	126.4	5.6868	126.5	8.5783	126.5	13.97	126.2	2.382792	126.3	3.517986	126.4	5.667195	126.5	7.92247	126.5	14.38656
126.2999954	2.266399917	126.3999939	3.4563	126.5	5.6835	126.6	8.5694	126.6	13.956	126.3	2.38015	126.4	3.513081	126.5	5.658524	126.6	7.909295	126.6	14.36555
126.4000015	2.263199868	126.5	3.452	126.6	5.68	126.7	8.5607	126.7	13.9454	126.4	2.377511	126.5	3.508184	126.6	5.649867	126.7	7.896145	126.7	14.34457
126.4999924	2.258699961	126.5999908	3.4428	126.7	5.6772	126.8	8.5507	126.8	13.9317	126.5	2.374875	126.6	3.503294	126.7	5.641224	126.8	7.883023	126.8	14.32364
126.5999985	2.257099922	126.6999969	3.4374	126.8	5.6729	126.9	8.5474	126.9	13.9155	126.6	2.372242	126.7	3.498411	126.8	5.632596	126.9	7.869923	126.9	14.30274
126.7000046	2.256700068	126.8000031	3.4344	126.9	5.6657	127	8.5407	127	13.8998	126.7	2.369611	126.8	3.493536	126.9	5.623982	127	7.856851	127	14.28189
126.7999954	2.254599919	126.8999939	3.4335	127	5.6636	127.1	8.5333	127.1	13.8866	126.8	2.366984	126.9	3.488667	127	5.615384	127.1	7.843801	127.1	14.26106
126.9000015	2.252999949	127	3.4306	127.1	5.6566	127.2	8.5256	127.2	13.872	126.9	2.36436	127	3.483807	127.1	5.606798	127.2	7.830775	127.2	14.24028
126.9999924	2.250899893	127.0999908	3.4302	127.2	5.6516	127.3	8.5149	127.3	13.8542	127	2.361739	127.1	3.478952	127.2	5.598227	127.3	7.817777	127.3	14.21953
127.0999985	2.249499914	127.1999969	3.4274	127.3	5.6434	127.4	8.5104	127.4	13.8357	127.1	2.359121	127.2	3.474105	127.3	5.589672	127.4	7.804801	127.4	14.19882
127.2000046	2.246299934	127.3000031	3.4233	127.4	5.6354	127.5	8.5086	127.5	13.8215	127.2	2.356506	127.3	3.469265	127.4	5.58113	127.5	7.791852	127.5	14.17815
127.2999954	2.243000083	127.3999939	3.4204	127.5	5.6325	127.6	8.5009	127.6	13.8101	127.3	2.353894	127.4	3.464432	127.5	5.572603	127.6	7.778925	127.6	14.15751
127.4000015	2.241100125	127.5	3.4224	127.6	5.628	127.7	8.4926	127.7	13.7934	127.4	2.351284	127.5	3.459607	127.6	5.564089	127.7	7.766023	127.7	14.13691
127.4999924	2.241099861	127.5999908	3.4193	127.7	5.6179	127.8	8.4834	127.8	13.7769	127.5	2.348678	127.6	3.454788	127.7	5.555589	127.8	7.753147	127.8	14.11635
127.5999985	2.239999852	127.6999969	3.4158	127.8	5.6152	127.9	8.4722	127.9	13.7594	127.6	2.346075	127.7	3.449976	127.8	5.547105	127.9	7.740293	127.9	14.09582
127.7000046	2.234999957	127.8000031	3.4127	127.9	5.6123	128	8.4615	128	13.7443	127.7	2.343474	127.8	3.445172	127.9	5.538634	128	7.727466	128	14.07533
127.7999954	2.234100047	127.8999939	3.4089	128	5.6024	128.1	8.4535	128.1	13.7327	127.8	2.340877	127.9	3.440375	128	5.530178	128.1	7.71466	128.1	14.05487
127.9000015	2.234699924	128	3.4033	128.1	5.5976	128.2	8.4439	128.2	13.7165	127.9	2.338283	128	3.435585	128.1	5.521734	128.2	7.701879	128.2	14.03446
127.9999924	2.229000056	128.0999908	3.4009	128.2	5.5922	128.3	8.4343	128.3	13.6992	128	2.335692	128.1	3.4308	128.2	5.513305	128.3	7.689127	128.3	14.01408
128.1000061	2.228200001	128.1999969	3.3981	128.3	5.5849	128.4	8.4264	128.4	13.6846	128.1	2.333103	128.2	3.426024	128.3	5.504893	128.4	7.676394	128.4	13.99373
128.2000122	2.227400111	128.3000031	3.3932	128.4	5.5809	128.5	8.4176	128.5	13.6662	128.2	2.330517	128.3	3.421256	128.4	5.496492	128.5	7.663685	128.5	13.97342
128.2999878	2.223999886	128.3999939	3.3936	128.5	5.571	128.6	8.4081	128.6	13.649	128.3	2.327935	128.4	3.416493	128.5	5.488105	128.6	7.651001	128.6	13.95315
128.3999939	2.219899681	128.5	3.3883	128.6	5.5612	128.7	8.3974	128.7	13.6368	128.4	2.325356	128.5	3.411737	128.6	5.479732	128.7	7.63834	128.7	13.93291
128.5	2.213000028	128.5999908	3.3829	128.7	5.5592	128.8	8.3919	128.8	13.6205	128.5	2.322779	128.6	3.406988	128.7	5.471373	128.8	7.625707	128.8	13.91272
128.6000061	2.20499999	128.6999969	3.3803	128.8	5.5559	128.9	8.3815	128.9	13.6116	128.6	2.320205	128.7	3.402246	128.8	5.46303	128.9	7.613094	128.9	13.89255
128.7000122	2.203600169	128.8000031	3.3782	128.9	5.5513	129	8.3737	129	13.5994	128.7	2.317634	128.8	3.397513	128.9	5.454699	129	7.600504	129	13.87242
128.7999878	2.199099789	128.8999939	3.3806	129	5.5462	129.1	8.3654	129.1	13.5873	128.8	2.315067	128.9	3.392785	129	5.446382	129.1	7.587939	129.1	13.85232
128.8999939	2.195399852	129	3.3772	129.1	5.5425	129.2	8.3595	129.2	13.5768	128.9	2.312501	129	3.388064	129.1	5.438078	129.2	7.575397	129.2	13.83226
129	2.192799972	129.0999908	3.377	129.2	5.5354	129.3	8.3523	129.3	13.5607	129	2.309939	129.1	3.38335	129.2	5.429789	129.3	7.562882	129.3	13.81224
129.1000061	2.192999985	129.1999969	3.3759	129.3	5.5307	129.4	8.3431	129.4	13.5448	129.1	2.30738	129.2	3.378643	129.3	5.421515	129.4	7.550387	129.4	13.79225
129.2000122	2.190700019	129.3000031	3.3752	129.4	5.5303	129.5	8.3362	129.5	13.5311	129.2	2.304824	129.3	3.373944	129.4	5.413253	129.5	7.537915	129.5	13.7723
129.2999878	2.190500019	129.3999939	3.377	129.5	5.5301	129.6	8.3287	129.6	13.5176	129.3	2.302271	129.4	3.36925	129.5	5.405005	129.6	7.525467	129.6	13.75238
129.3999939	2.194199563	129.5	3.3812	129.6	5.5288	129.7	8.32	129.7	13.5019	129.4	2.29972	129.5	3.364564	129.6	5.39677	129.7	7.513042	129.7	13.7325
129.5	2.198600016	129.5999908	3.382399	129.7	5.5206	129.8	8.3132	129.8	13.488	129.5	2.297173	129.6	3.359884	129.7	5.388549	129.8	7.500644	129.8	13.71266
129.6000061	2.2006	129.6999969	3.3775	129.8	5.5142	129.9	8.3049	129.9	13.4712	129.6	2.294628	129.7	3.355211	129.8	5.380344	129.9	7.488266	129.9	13.69284
129.7000122	2.196700215	129.8000031	3.375	129.9	5.5107	130	8.3005	130	13.4584	129.7	2.292086	129.8	3.350546	129.9	5.37215	130	7.475911	130	13.67306
129.7999878	2.195399749	129.8999939	3.3751	130	5.5087	130.1	8.2939	130.1	13.4478	129.8	2.289548	129.9	3.345887	130	5.36397	130.1	7.463579	130.1	13.65332
129.8999939	2.196799584	130	3.3752	130.1	5.5031	130.2	8.2847	130.2	13.4301	129.9	2.287012	130	3.341235	130.1	5.355803	130.2	7.45127	130.2	13.63361
130	2.196099983	130.0999908	3.3768	130.2	5.4978	130.3	8.2774	130.3	13.411	130	2.284479	130.1	3.336589	130.2	5.34765	130.3	7.438988	130.3	13.61394
130.1000061	2.196400007	130.1999969	3.3716	130.3	5.4932	130.4	8.2705	130.4	13.4002	130.1	2.281948	130.2	3.33195	130.3	5.339513	130.4	7.426725	130.4	13.5943
130.2000122	2.19229995	130.3000031	3.3688	130.4	5.4894	130.5	8.2591	130.5	13.3849	130.2	2.279421	130.3	3.327319	130.4	5.331387	130.5	7.414485	130.5	13.57469
130.2999878	2.18840002	130.3999939	3.3689	130.5	5.4857	130.6	8.2513	130.6	13.368	130.3	2.276897	130.4	3.322694	130.5	5.323274	130.6	7.402268	130.6	13.55512
130.3999939	2.1875996	130.5	3.3623	130.6	5.48	130.7	8.242	130.7	13.3556	130.4	2.274376	130.5	3.318075	130.6	5.315175	130.7	7.390073	130.7	13.53558
130.5	2.183800004	130.5999908	3.3578	130.7	5.4739	130.8	8.2332	130.8	13.3472	130.5	2.271857	130.6	3.313464	130.7	5.307089	130.8	7.377905	130.8	13.51609
130.6000061	2.180599983	130.6999969	3.3541	130.8	5.4682	130.9	8.224	130.9	13.3358	130.6	2.269341	130.7	3.308858	130.8	5.299019	130.9	7.365756	130.9	13.49662
130.7000122	2.178500407	130.8000031	3.3514	130.9	5.462	131	8.2177	131	13.3244	130.7	2.266828	130.8	3.304261	130.9	5.29096	131	7.35363	131	13.47718
130.7999878	2.177999561	130.8999939	3.3484	131	5.4586	131.1	8.2024	131.1	13.3088	130.8	2.264318	130.9	3.29967	131	5.282914	131.1	7.341527	131.1	13.45778
130.8999939	2.175099872	131	3.3472	131.1	5.4559	131.2	8.189701	131.2	13.2962	130.9	2.261811	131	3.295085	131.1	5.274881	131.2	7.329445	131.2	13.43841
131	2.169200005	131.0999908	3.3454	131.2	5.4529	131.3	8.188199	131.3	13.2861	131	2.259307	131.1	3.290506	131.2	5.266862	131.3	7.317391	131.3	13.41908
131.1000061	2.165899985	131.1999969	3.344	131.3	5.4475	131.4	8.183	131.4	13.2732	131.1	2.256805	131.2	3.285935	131.3	5.258859	131.4	7.305354	131.4	13.39978
131.2000122	2.165701059	131.3000031	3.3485	131.4	5.4433	131.5	8.1791	131.5	13.2652	131.2	2.254306	131.3	3.281371	131.4	5.250866	131.5	7.293341	131.5	13.38052
131.2999878	2.170998969	131.3999939	3.346	131.5	5.4415	131.6	8.1717	131.6	13.2499	131.3	2.251811	131.4	3.276813	131.5	5.242886	131.6	7.281349	131.6	13.36128
131.3999939	2.169799833	131.5	3.3469	131.6	5.4363	131.7	8.1619	131.7	13.2352	131.4	2.249318	131.5	3.272261	131.6	5.23492	131.7	7.26938	131.7	13.34208
131.5	2.169399994	131.5999908	3.3418	131.7	5.4327	131.8	8.1514	131.8	13.2204	131.5	2.246827	131.6	3.267716	131.7	5.226967	131.8	7.257437	131.8	13.32292
131.6000061	2.16699997	131.6999969	3.3341	131.8	5.4303	131.9	8.143599	131.9	13.2096	131.6	2.24434	131.7	3.263178	131.8	5.219029	131.9	7.245512	131.9	13.30379
131.7000122	2.160599765	131.8000031	3.3279	131.9	5.4251	132	8.134	132	13.197	131.7	2.241855	131.8	3.258648	131.9	5.211102	132	7.233609	132	13.28469
131.7999878	2.157400274	131.8999939	3.328299	132	5.4214	132.1	8.127	132.1	13.1868	131.8	2.239374	131.9	3.254122	132	5.203189	132.1	7.221729	132.1	13.26562
131.8999939	2.153399494	132	3.3243	132.1	5.4181	132.2	8.1237	132.2	13.1775	131.9	2.236895	132	3.249604	132.1	5.195288	132.2	7.209871	132.2	13.24659
132	2.149499977	132.0999908	3.3221	132.2	5.4168	132.3	8.117	132.3	13.1644	132	2.234419	132.1	3.245092	132.2	5.1874	132.3	7.198038	132.3	13.22759
132.1000061	2.149499988	132.1999969	3.3232	132.3	5.4094	132.4	8.1115	132.4	13.1516	132.1	2.231946	132.2	3.240586	132.3	5.179528	132.4	7.186223	132.4	13.20863
132.2000122	2.150500046	132.3000031	3.3211	132.4	5.4055	132.5	8.1033	132.5	13.1368	132.2	2.229475	132.3	3.236089	132.4	5.171666	132.5	7.174431	132.5	13.18969
132.2999878	2.147799934	132.3999939	3.3194	132.5	5.3985	132.6	8.0946	132.6	13.1242	132.3	2.227008	132.4	3.231597	132.5	5.163817	132.6	7.16266	132.6	13.17079
132.3999939	2.146499564	132.5	3.3144	132.6	5.3938	132.7	8.091201	132.7	13.112	132.4	2.224543	132.5	3.227111	132.6	5.155981	132.7	7.150911	132.7	13.15192
132.5	2.148399995	132.5999908	3.3111	132.7	5.3896	132.8	8.085699	132.8	13.1027	132.5	2.222081	132.6	3.222632	132.7	5.148158	132.8	7.139187	132.8	13.13309
132.6000061	2.148299971	132.6999969	3.3152	132.8	5.3875	132.9	8.0778	132.9	13.0931	132.6	2.219622	132.7	3.218159	132.8	5.140351	132.9	7.127481	132.9	13.11428
132.7000122	2.148200767	132.8000031	3.3199	132.9	5.3833	133	8.0716	133	13.0871	132.7	2.217165	132.8	3.213694	132.9	5.132553	133	7.115798	133	13.09551
132.7999878	2.153299256	132.8999939	3.3195	133	5.3825	133.1	8.0702	133.1	13.0769	132.8	2.214712	132.9	3.209234	133	5.124769	133.1	7.104135	133.1	13.07677
132.8999939	2.157099829	133	3.3209	133.1	5.3808	133.2	8.068001	133.2	13.0651	132.9	2.212262	133	3.204781	133.1	5.116997	133.2	7.092494	133.2	13.05806
133	2.156899983	133.0999908	3.321099	133.2	5.3801	133.3	8.061399	133.3	13.0553	133	2.209813	133.1	3.200334	133.2	5.109238	133.3	7.080879	133.3	13.0394
133.1000061	2.156200027	133.1999969	3.3194	133.3	5.3803	133.4	8.0547	133.4	13.0438	133.1	2.207368	133.2	3.195894	133.3	5.101495	133.4	7.069281	133.4	13.02075
133.2000122	2.155100621	133.3000031	3.3194	133.4	5.3789	133.5	8.0479	133.5	13.0339	133.2	2.204925	133.3	3.191462	133.4	5.093761	133.5	7.057704	133.5	13.00214
133.2999878	2.156599394	133.3999939	3.3174	133.5	5.3764	133.6	8.0405	133.6	13.0199	133.3	2.202486	133.4	3.187034	133.5	5.086041	133.6	7.046149	133.6	12.98356

133.3999939	2.155499762	133.5	3.3141	133.6	5.3672	133.7	8.033901	133.7	13.0038	133.4	2.200049	133.5	3.182613	133.6	5.078333	133.7	7.034615	133.7	12.96502
133.5	2.153700024	133.5999908	3.3109	133.7	5.3632	133.8	8.031399	133.8	12.9969	133.5	2.197615	133.6	3.178199	133.7	5.070638	133.8	7.023106	133.8	12.94651
133.6000061	2.153300021	133.6999969	3.3111	133.8	5.3641	133.9	8.0284	133.9	12.986	133.6	2.195183	133.7	3.173791	133.8	5.062957	133.9	7.011615	133.9	12.92802
133.7000122	2.151900767	133.8000031	3.3126	133.9	5.3543	134	8.0223	134	12.9747	133.7	2.192755	133.8	3.169391	133.9	5.055287	134	7.000145	134	12.90957
133.7999878	2.159099229	133.8999939	3.3114	134	5.35	134.1	8.0129	134.1	12.961	133.8	2.190329	133.9	3.164995	134	5.04763	134.1	6.988695	134.1	12.89115
133.8999939	2.161999505	134	3.3118	134.1	5.3446	134.2	8.0076	134.2	12.9458	133.9	2.187906	134	3.160606	134.1	5.039985	134.2	6.977267	134.2	12.87276
134	2.159800009	134.0999908	3.3093	134.2	5.3414	134.3	8.0032	134.3	12.9363	134	2.185486	134.1	3.156224	134.2	5.032353	134.3	6.965864	134.3	12.85441
134.1000061	2.158200012	134.1999969	3.3091	134.3	5.3362	134.4	7.9954	134.4	12.9255	134.1	2.183068	134.2	3.151848	134.3	5.024735	134.4	6.954478	134.4	12.83608
134.2000122	2.154800231	134.3000031	3.3087	134.4	5.3346	134.5	7.9886	134.5	12.9157	134.2	2.180653	134.3	3.147479	134.4	5.017128	134.5	6.943113	134.5	12.81779
134.2999878	2.155399738	134.3999939	3.308	134.5	5.3339	134.6	7.9844	134.6	12.9047	134.3	2.178241	134.4	3.143116	134.5	5.009533	134.6	6.931768	134.6	12.79953
134.3999939	2.159299659	134.5	3.3053	134.6	5.3352	134.7	7.9773	134.7	12.8926	134.4	2.175832	134.5	3.138759	134.6	5.001951	134.7	6.920445	134.7	12.7813
134.5	2.164400014	134.5999908	3.303299	134.7	5.3341	134.8	7.973	134.8	12.8815	134.5	2.173425	134.6	3.134408	134.7	4.994381	134.8	6.909146	134.8	12.7631
134.6000061	2.165799989	134.6999969	3.3007	134.8	5.3299	134.9	7.9643	134.9	12.8693	134.6	2.171021	134.7	3.130064	134.8	4.986825	134.9	6.897864	134.9	12.74493
134.7000122	2.165300348	134.8000031	3.2976	134.9	5.3215	135	7.959	135	12.8577	134.7	2.16862	134.8	3.125727	134.9	4.97928	135	6.886602	135	12.72679
134.7999878	2.165899656	134.8999939	3.2943	135	5.3229	135.1	7.9539	135.1	12.8427	134.8	2.166222	134.9	3.121395	135	4.971747	135.1	6.875362	135.1	12.70868
134.8999939	2.16420012	135	3.2904	135.1	5.3193	135.2	7.948901	135.2	12.828	134.9	2.163826	135	3.117069	135.1	4.964227	135.2	6.864142	135.2	12.69061
135	2.166700015	135.0999908	3.2903	135.2	5.3136	135.3	7.947299	135.3	12.8164	135	2.161433	135.1	3.11275	135.2	4.956719	135.3	6.852946	135.3	12.67257
135.1000061	2.165699989	135.1999969	3.2888	135.3	5.3144	135.4	7.9392	135.4	12.8048	135.1	2.159042	135.2	3.108437	135.3	4.949225	135.4	6.841767	135.4	12.65455
135.2000122	2.164400576	135.3000031	3.2883	135.4	5.3083	135.5	7.9307	135.5	12.7908	135.2	2.156655	135.3	3.104132	135.4	4.941741	135.5	6.830608	135.5	12.63657
135.2999878	2.168599418	135.3999939	3.2843	135.5	5.2995	135.6	7.9226	135.6	12.7776	135.3	2.15427	135.4	3.099831	135.5	4.93427	135.6	6.81947	135.6	12.61861
135.3999939	2.166199998	135.5	3.2776	135.6	5.2925	135.7	7.915701	135.7	12.7652	135.4	2.151888	135.5	3.095537	135.6	4.926811	135.7	6.808352	135.7	12.60069
135.5	2.160399994	135.5999908	3.2729	135.7	5.2853	135.8	7.913099	135.8	12.7579	135.5	2.149508	135.6	3.091249	135.7	4.919364	135.8	6.797258	135.8	12.5828
135.6000061	2.156399984	135.6999969	3.2706	135.8	5.2854	135.9	7.9014	135.9	12.7464	135.6	2.147131	135.7	3.086967	135.8	4.911931	135.9	6.786181	135.9	12.56494
135.7000122	2.152300937	135.8000031	3.2721	135.9	5.2834	136	7.8932	136	12.7347	135.7	2.144757	135.8	3.082693	135.9	4.904508	136	6.775124	136	12.54711
135.7999878	2.154999045	135.8999939	3.2675	136	5.279	136.1	7.8838	136.1	12.7196	135.8	2.142386	135.9	3.078423	136	4.897098	136.1	6.764088	136.1	12.5293
135.8999939	2.154800069	136	3.2637	136.1	5.2721	136.2	7.8689	136.2	12.7021	135.9	2.140018	136	3.07416	136.1	4.889699	136.2	6.753071	136.2	12.51153
136	2.152500029	136.0999908	3.2631	136.2	5.265	136.3	7.8686	136.3	12.6904	136	2.137651	136.1	3.069903	136.2	4.882313	136.3	6.742078	136.3	12.49379
136.1000061	2.151500005	136.1999969	3.2562	136.3	5.2582	136.4	7.8647	136.4	12.6768	136.1	2.135288	136.2	3.065652	136.3	4.874941	136.4	6.731101	136.4	12.47608
136.2000122	2.149000103	136.3000031	3.2555	136.4	5.253	136.5	7.8583	136.5	12.6637	136.2	2.132927	136.3	3.061409	136.4	4.867579	136.5	6.720145	136.5	12.4584
136.2999878	2.146999922	136.3999939	3.2547	136.5	5.2466	136.6	7.8568	136.6	12.6526	136.3	2.13057	136.4	3.05717	136.5	4.860228	136.6	6.709208	136.6	12.44075
136.3999939	2.14699977	136.5	3.255	136.6	5.2429	136.7	7.8544	136.7	12.6445	136.4	2.128214	136.5	3.052937	136.6	4.85289	136.7	6.698292	136.7	12.42313
136.5	2.143500012	136.5999908	3.256199	136.7	5.2395	136.8	7.8517	136.8	12.6325	136.5	2.125862	136.6	3.048711	136.7	4.845564	136.8	6.687399	136.8	12.40554
136.6000061	2.145099984	136.6999969	3.2617	136.8	5.2369	136.9	7.8476	136.9	12.6201	136.6	2.123512	136.7	3.044491	136.8	4.838252	136.9	6.676522	136.9	12.38797
136.7000122	2.143400154	136.8000031	3.2624	136.9	5.2332	137	7.8452	137	12.6066	136.7	2.121164	136.8	3.040278	136.9	4.830949	137	6.665665	137	12.37044
136.7999878	2.139299817	136.8999939	3.2634	137	5.2289	137.1	7.8398	137.1	12.5954	136.8	2.11882	136.9	3.03607	137	4.823659	137.1	6.654827	137.1	12.35294
136.8999939	2.140199636	137	3.2607	137.1	5.2282	137.2	7.8343	137.2	12.5855	136.9	2.116478	137	3.031868	137.1	4.81638	137.2	6.64401	137.2	12.33546
137	2.142099994	137.0999908	3.2597	137.2	5.2269	137.3	7.8303	137.3	12.5735	137	2.114138	137.1	3.027672	137.2	4.809113	137.3	6.633215	137.3	12.31802
137.1000061	2.143800001	137.1999969	3.258	137.3	5.2203	137.4	7.819401	137.4	12.5569	137.1	2.111802	137.2	3.023483	137.3	4.80186	137.4	6.622437	137.4	12.30061
137.2000122	2.146100055	137.3000031	3.256	137.4	5.2144	137.5	7.8122	137.5	12.5454	137.2	2.109468	137.3	3.0193	137.4	4.794617	137.5	6.611678	137.5	12.28322
137.2999878	2.142099942	137.3999939	3.249901	137.5	5.2085	137.6	7.805	137.6	12.5301	137.3	2.107137	137.4	3.015123	137.5	4.787386	137.6	6.600939	137.6	12.26587
137.3999939	2.134100554	137.5	3.2484	137.6	5.2058	137.7	7.803701	137.7	12.5206	137.4	2.104808	137.5	3.010951	137.6	4.780166	137.7	6.590219	137.7	12.24854
137.5	2.130000028	137.5999908	3.2479	137.7	5.2072	137.8	7.803099	137.8	12.5123	137.5	2.102482	137.6	3.006786	137.7	4.772958	137.8	6.579522	137.8	12.23124
137.6000061	2.126100011	137.6999969	3.2481	137.8	5.2034	137.9	7.802199	137.9	12.5058	137.6	2.100158	137.7	3.002626	137.8	4.765764	137.9	6.568841	137.9	12.21397
137.7000122	2.127800582	137.8000031	3.2513	137.9	5.1997	138	7.7957	138	12.4921	137.7	2.097837	137.8	2.998474	137.9	4.75858	138	6.558179	138	12.19673
137.7999878	2.127699425	137.8999939	3.254399	138	5.194	138.1	7.7883	138.1	12.477	137.8	2.095519	137.9	2.994326	138	4.751407	138.1	6.547537	138.1	12.17952
137.8999939	2.132999215	138	3.2527	138.1	5.187	138.2	7.7825	138.2	12.468	137.9	2.093204	138	2.990185	138.1	4.744246	138.2	6.536914	138.2	12.16234
138	2.135300009	138.0999908	3.2487	138.2	5.185	138.3	7.7712	138.3	12.4541	138	2.090891	138.1	2.986049	138.2	4.737096	138.3	6.526313	138.3	12.14519
138.1000061	2.133599972	138.1999969	3.2481	138.3	5.1785	138.4	7.7638	138.4	12.4424	138.1	2.08858	138.2	2.98192	138.3	4.72996	138.4	6.515729	138.4	12.12806
138.2000122	2.134599798	138.3000031	3.2434	138.4	5.1753	138.5	7.7541	138.5	12.4258	138.2	2.086272	138.3	2.977797	138.4	4.722834	138.5	6.505163	138.5	12.11097
138.2999878	2.130300221	138.3999939	3.2424	138.5	5.1689	138.6	7.7471	138.6	12.4136	138.3	2.083968	138.4	2.97368	138.5	4.715719	138.6	6.494617	138.6	12.0939
138.3999939	2.128499824	138.5	3.2381	138.6	5.1689	138.7	7.7412	138.7	12.4034	138.4	2.081665	138.5	2.969568	138.6	4.708616	138.7	6.48409	138.7	12.07686
138.5	2.123299989	138.5999908	3.2378	138.7	5.1653	138.8	7.7386	138.8	12.3897	138.5	2.079365	138.6	2.965462	138.7	4.701525	138.8	6.473585	138.8	12.05985
138.6000061	2.121799999	138.6999969	3.238	138.8	5.1584	138.9	7.7317	138.9	12.3738	138.6	2.077068	138.7	2.961362	138.8	4.694447	138.9	6.463096	138.9	12.04287
138.7000122	2.123399862	138.8000031	3.2337	138.9	5.1535	139	7.7264	139	12.3586	138.7	2.074773	138.8	2.95727	138.9	4.687378	139	6.452625	139	12.02592
138.7999878	2.121000173	138.8999939	3.2291	139	5.1477	139.1	7.719	139.1	12.339	138.8	2.072481	138.9	2.953182	139	4.680321	139.1	6.442174	139.1	12.00899
138.8999939	2.112800301	139	3.2249	139.1	5.1419	139.2	7.7097	139.2	12.3223	138.9	2.070192	139	2.9491	139.1	4.673275	139.2	6.431741	139.2	11.99209
139	2.108999974	139.0999908	3.2191	139.2	5.1354	139.3	7.6992	139.3	12.3073	139	2.067905	139.1	2.945024	139.2	4.666241	139.3	6.421331	139.3	11.97523
139.1000061	2.105500016	139.1999969	3.2165	139.3	5.1308	139.4	7.6922	139.4	12.2932	139.1	2.06562	139.2	2.940953	139.3	4.65922	139.4	6.410936	139.4	11.95838
139.2000122	2.104500058	139.3000031	3.2158	139.4	5.126	139.5	7.6867	139.5	12.281	139.2	2.063339	139.3	2.93689	139.4	4.652208	139.5	6.400559	139.5	11.94157
139.2999878	2.102599929	139.3999939	3.2163	139.5	5.1234	139.6	7.6823	139.6	12.2712	139.3	2.06106	139.4	2.932832	139.5	4.645208	139.6	6.390202	139.6	11.92479
139.3999939	2.100800138	139.5	3.2156	139.6	5.1229	139.7	7.673801	139.7	12.2635	139.4	2.058783	139.5	2.928779	139.6	4.638219	139.7	6.379863	139.7	11.90803
139.5	2.101199999	139.5999908	3.2182	139.7	5.1225	139.8	7.668499	139.8	12.2557	139.5	2.056509	139.6	2.924732	139.7	4.631242	139.8	6.369546	139.8	11.8913
139.6000061	2.10080002	139.6999969	3.2187	139.8	5.1213	139.9	7.6618	139.9	12.2431	139.6	2.054238	139.7	2.920691	139.8	4.624278	139.9	6.359244	139.9	11.8746
139.7000122	2.101200603	139.8000031	3.2201	139.9	5.1186	140	7.6529	140	12.2311	139.7	2.051969	139.8	2.916657	139.9	4.617323	140	6.348961	140	11.85793
139.7999878	2.105299446	139.8999939	3.2196	140	5.1231	140.1	7.6452	140.1	12.2184	139.8	2.049703	139.9	2.912628	140	4.610379	140.1	6.338696	140.1	11.84128
139.8999939	2.101699781	140	3.2211	140.1	5.1204	140.2	7.6424	140.2	12.2068	139.9	2.047439	140	2.908604	140.1	4.603446	140.2	6.32845	140.2	11.82466
140	2.098700021	140.0999908	3.2183	140.2	5.1212	140.3	7.6338	140.3	12.1944	140	2.045178	140.1	2.904586	140.2	4.596525	140.3	6.318225	140.3	11.80807
140.1000061	2.099899971	140.1999969	3.2184	140.3	5.1142	140.4	7.6265	140.4	12.183	140.1	2.042919	140.2	2.900575	140.3	4.589617	140.4	6.308016	140.4	11.79151
140.2000122	2.101500148	140.3000031	3.2142	140.4	5.1137	140.5	7.6207	140.5	12.1702	140.2	2.040663	140.3	2.89657	140.4	4.582718	140.5	6.297824	140.5	11.77497
140.2999878	2.098899863	140.3999939	3.2143	140.5	5.1109	140.6	7.6176	140.6	12.1558	140.3	2.03841	140.4	2.892569	140.5	4.57583	140.6	6.287652	140.6	11.75846
140.3999939	2.095899745	140.5	3.2131	140.6	5.1082	140.7	7.614	140.7	12.1443	140.4	2.036159	140.5	2.888575	140.6	4.568954	140.7	6.277497	140.7	11.74198
140.5	2.09230002	140.5999908	3.2101	140.7	5.1058	140.8	7.6068	140.8	12.1319	140.5	2.033911	140.6	2.884586	140.7	4.562088	140.8	6.267364	140.8	11.72553
140.6000061	2.089799993	140.6999969	3.2107	140.8	5.1021	140.9	7.601	140.9	12.1169	140.6	2.031665	140.7	2.880603	140.8	4.555236	140.9	6.257245	140.9	11.7091
140.7000122	2.090600116	140.8000031	3.2074	140.9	5.0994	141	7.5936	141	12.1036	140.7	2.029421	140.8	2.876626	140.9	4.548392	141	6.247145	141	11.6927
140.7999878	2.091699848	140.8999939	3.2081	141	5.0962	141.1	7.5862	141.1	12.0907	140.8	2.027181	140.9	2.872655	141	4.54156	141.1	6.237063	141.1	11.67633
140.8999939	2.089499756	141	3.2087	141.1	5.0914	141.2	7.5812	141.2	12.0835	140.9	2.024943	141	2.868689	141.1	4.534739	141.2	6.226999	141.2	11.65998
141	2.089199988	141.0999908	3.2106	141.2	5.0881	141.3	7.5727	141.3	12.0746	141	2.022707	141.1	2.864729	141.2	4.527928	141.3	6.216957	141.3	11.64367
141.1000061	2.087100015	141.1999969	3.2122	141.3	5.0857	141.4	7.564	141.4	12.0661	141.1	2.020474	141.2	2.860774	141.3	4.521131	141.4	6.206929	141.4	11.62738
141.2000122	2.092800056	141.3000031	3.2088	141.4	5.0817	141.5	7.5533	141.5	12.0513	141.2	2.018243	141.3	2.856826	141.4	4.514343	141.5	6.196919	141.5	11.61111
141.2999878	2.091799927	141.3999939	3.2089	141.5	5.076	141.6	7.5487	141.6	12.0408	141.3	2.016015	141.4	2.852883	141.5	4.507565	141.6	6.186927	141.6	11.59487
141.3999939	2.090799977	141.5	3.2065	141.6	5.0758	141.7	7.5485	141.7	12.031	141.4	2.01379	141.5	2.848946	141.6	4.500799	141.7	6.176952	141.7	11.57866
141.5	2.086000013	141.5999908	3.2057	141.7	5.0748	141.8	7.5407	141.8	12.0195	141.5	2.011566	141.6	2.845014	141.7	4.494043	141.8	6.166999	141.8	11.56248
141.6000061	2.083000014	141.6999969	3.2023	141.8	5.072	141.9	7.5355	141.9	12.01	141.6	2.009346	141.7	2.841088	141.8	4.487301	141.9	6.15706	141.9	11.54632
141.7000122	2.081199869	141.8000031	3.1973	141.9	5.0676	142	7.5365	142	12.0016	141.7	2.007128	141.8	2.837169	141.9	4.480567	142	6.14714	142	11.53019
141.7999878	2.08140011	141.8999939	3.1963	142	5.0649	142.1	7.5297	142.1	11.9916	141.8	2.004913	141.9	2.833254	142	4.473844	142.1	6.137236	142.1	11.51408
141.8999939	2.083199641	142	3.1983	142.1	5.0582	142.2	7.5217	142.2	11.9834	141.9	2.002699	142	2.829344	142.1	4.467132	142.2	6.127351	142.2	11.498
142	2.084199977	142.0999908	3.1982	142.2	5.0544	142.3	7.5187	142.3	11.9748	142	2.000489	142.1	2.825441	142.2	4.46043	142.3	6.117486	142.3	11.48195
142.1000061	2.084000024	142.1999969	3.196	142.3	5.0519	142.4	7.5084	142.4	11.9632	142.1	1.998281	142.2	2.821543	142.3	4.453742	142.4	6.107636	142.4	11.46593
142.2000122	2.082900412	142.3000031	3.1958	142.4	5.0502	142.5	7.5025	142.5	11.9506	142.2	1.996075	142.3	2.817652	142.4	4.447062	142.5	6.097803	142.5	11.44993
142.2999878	2.082299609	142.3999939	3.1897	142.5	5.0452	142.6	7.4965	142.6	11.9405	142.3	1.993873	142.4	2.813765	142.5	4.440393	142.6	6.087988	142.6	11.43395
142.3999939	2.078100307	142.5	3.1827	142.6	5.0433	142.7	7.4857	142.7	11.9276	142.4	1.991672	142.5	2.809884	142.6	4.433735	142.7	6.07819	142.7	11.41801
142.5	2.076399992	142.5999908	3.1757	142.7	5.0402	142.8	7.4781	142.8	11.9151	142.5	1.989474	142.6	2.806008	142.7	4.427087	142.8	6.068413	142.8	11.40209
142.6000061	2.077399975	142.6999969	3.173	142.8	5.0363	142.9	7.4693	142.9	11.9004	142.6	1.987278	142.7	2.802138	142.8	4.420452	142.9	6.05865	142.9	11.38619
142.7000122	2.071299835	142.8000031	3.1669	142.9	5.0296	143	7.4656	143	11.8858	142.7	1.985085	142.8	2.798275	142.9	4.413826	143	6.048905	143	11.37032
142.7999878	2.070000146	142.8999939	3.1629	143	5.0288	143.1	7.457	143.1	11.8721	142.8	1.982895	142.9	2.794416	143	4.40721	143.1	6.039177	143.1	11.35448
142.8999939	2.068600159	143	3.1632	143.1	5.0264	143.2	7.452	143.2	11.8621	142.9	1.980706	143	2.790562	143.1	4.400605	143.2	6.029466	143.2	11.33866
143	2.067300016	143.0999908	3.1615	143.2	5.0211	143.3	7.4487	143.3	11.8522	143	1.978521	143.1	2.786715	143.2	4.394011	143.3	6.019776	143.3	11.32287
143.1000061	2.063000026	143.1999969	3.1602	143.3	5.0166	143.4	7.4374	143.4	11.8406	143.1	1.976337	143.2	2.782872	143.3	4.387429	143.4	6.010099	143.4	11.30711
143.2000122	2.065200116	143.3000031	3.1587	143.4	5.0134	143.5	7.4299	143.5	11.8306	143.2	1.974156	143.3	2.779037	143.4	4.380856	143.5	6.00044	143.5	11.29137
143.2999878	2.065099918	143.3999939	3.1552	143.5	5.0119	143.6	7.4261	143.6	11.821	143.3	1.971979	143.4	2.775205	143.5	4.374294	143.6	5.990798	143.6	11.27565
143.3999939	2.068600116	143.5	3.1525	143.6	5.0128	143.7	7.4191	143.7	11.8116	143.4	1.969803	143.5	2.77138	143.6	4.367741	143.7	5.981173	143.7	11.25996
143.5	2.070599971	143.5999908	3.1533	143.7	5.009	143.8	7.414	143.8	11.8031	143.5	1.967629	143.6	2.767559	143.7	4.3612	143.8	5.971569	143.8	11.2443
143.6000061	2.075699975	143.6999969	3.1492	143.8	5.005	143.9	7.4076	143.9	11.7926	143.6	1.965458	143.7	2.763745	143.8	4.354671	143.9	5.961978	143.9	11.22866
143.7000122	2.075600217	143.8000031	3.1515	143.9	5.0026	144	7.4051	144	11.788	143.7	1.96329	143.8	2.759936	143.9	4.34815	144	5.952404	144	11.21305
143.7999878	2.073399794	143.8999939	3.1511	144	5.0022	144.1	7.4015	144.1	11.7836	143.8	1.961124	143.9	2.756132	144	4.34164	144.1	5.942848	144.1	11.19746
143.8999939	2.074799708	144	3.1535	144.1	5.0032	144.2	7.4014	144.2	11.7788	143.9	1.95896	144	2.752334	144.1	4.33514	144.2	5.933308	144.2	11.1819
144	2.073899974	144.0999908	3.153899	144.2	5.0038	144.3	7.3987	144.3	11.7685	144	1.956799	144.1	2.748541	144.2	4.328651	144.3	5.923788	144.3	11.16637
144.1000061	2.074499999	144.1999969	3.1592	144.3	4.9982	144.4	7.3951	144.4	11.7585	144.1	1.95464	144.2	2.744754	144.3	4.322174	144.4	5.914282	144.4	11.15086
144.2000122	2.079900123	144.3000031	3.1598	144.4	4.9963	144.5	7.3909	144.5	11.7473	144.2	1.952484	144.3	2.740973	144.4	4.315705	144.5	5.904793	144.5	11.13537
144.2999878	2.080099855	144.3999939	3.1578	144.5	4.9943	144.6	7.3882	144.6	11.7417	144.3	1.950331	144.4	2.737196	144.5	4.309247	144.6	5.89532	144.6	11.11991
144.3999939	2.083199641	144.5	3.1551	144.6	4.9964	144.7	7.3826	144.7	11.7305	144.4	1.948179	144.5	2.733425	144.6	4.302799	144.7	5.885865	144.7	11.10448
144.5	2.081600019	144.5999908	3.155299	144.7	4.9896	144.8	7.3772	144.8	11.7167	144.5	1.94603	144.6	2.729659	144.7	4.296362	144.8	5.876429	144.8	11.08907
144.6000061	2.084199981	144.6999969	3.1514	144.8	4.9905	144.9	7.371499	144.9	11.7116	144.6	1.943884	144.7	2.725899	144.8	4.289937	144.9	5.867007	144.9	11.07368
144.7000122	2.079799894	144.8000031	3.1474	144.9	4.9923	145	7.3662	145	11.7071	144.7	1.941739	144.8	2.722145	144.9	4.28352	145	5.857601	145	11.05832
144.7999878	2.075000119	144.8999939	3.150099	145	4.9898	145.1	7.3593	145.1	11.7	144.8	1.939598	144.9	2.718395	145	4.277113	145.1	5.848212	145.1	11.04299

144.8999939	2.078899273	145	3.1477	145.1	4.9916	145.2	7.3541	145.2	11.6939	144.9	1.937459	145	2.714651	145.1	4.270717	145.2	5.83884	145.2	11.02767
145	2.081999973	145.0999908	3.150599	145.2	4.9892	145.3	7.3503	145.3	11.6854	145	1.935322	145.1	2.710912	145.2	4.264331	145.3	5.829487	145.3	11.01239
145.1000061	2.083000027	145.1999969	3.1513	145.3	4.9863	145.4	7.3457	145.4	11.6745	145.1	1.933187	145.2	2.707178	145.3	4.257957	145.4	5.820148	145.4	10.99713
145.2000122	2.082400415	145.3000031	3.1495	145.4	4.9796	145.5	7.3399	145.5	11.6619	145.2	1.931055	145.3	2.703451	145.4	4.251591	145.5	5.810825	145.5	10.98189
145.2999878	2.080999568	145.3999939	3.1425	145.5	4.9743	145.6	7.3357	145.6	11.6564	145.3	1.928926	145.4	2.699729	145.5	4.245235	145.6	5.801519	145.6	10.96668
145.3999939	2.080500204	145.5	3.1371	145.6	4.9701	145.7	7.3294	145.7	11.6465	145.4	1.926799	145.5	2.696011	145.6	4.23889	145.7	5.792229	145.7	10.95149
145.5	2.078399972	145.5999908	3.136399	145.7	4.9653	145.8	7.3241	145.8	11.6341	145.5	1.924674	145.6	2.692299	145.7	4.232555	145.8	5.782958	145.8	10.93633
145.6000061	2.08149998	145.6999969	3.132	145.8	4.9586	145.9	7.3194	145.9	11.6255	145.6	1.922551	145.7	2.688592	145.8	4.226231	145.9	5.773701	145.9	10.92119
145.7000122	2.084199739	145.8000031	3.1261	145.9	4.9571	146	7.3129	146	11.6132	145.7	1.920431	145.8	2.684892	145.9	4.219916	146	5.76446	146	10.90608
145.7999878	2.080200248	145.8999939	3.1215	146	4.9517	146.1	7.3111	146.1	11.6023	145.8	1.918314	145.9	2.681195	146	4.213611	146.1	5.755236	146.1	10.89099
145.8999939	2.076899967	146	3.1177	146.1	4.9483	146.2	7.3045	146.2	11.5849	145.9	1.916199	146	2.677504	146.1	4.207317	146.2	5.746027	146.2	10.87592
146	2.075700026	146.0999908	3.117	146.2	4.9411	146.3	7.2951	146.3	11.573	146	1.914086	146.1	2.673819	146.2	4.201032	146.3	5.736838	146.3	10.86089
146.1000061	2.075099993	146.1999969	3.1126	146.3	4.9329	146.4	7.2939	146.4	11.5623	146.1	1.911975	146.2	2.670138	146.3	4.194759	146.4	5.727662	146.4	10.84587
146.2000122	2.069199701	146.3000031	3.1072	146.4	4.9269	146.5	7.2915	146.5	11.5503	146.2	1.909867	146.3	2.666464	146.4	4.188494	146.5	5.718502	146.5	10.83087
146.2999878	2.066400306	146.3999939	3.1071	146.5	4.9252	146.6	7.2903	146.6	11.5423	146.3	1.907762	146.4	2.662794	146.5	4.182239	146.6	5.709359	146.6	10.8159
146.3999939	2.064699833	146.5	3.1071	146.6	4.9231	146.7	7.2862	146.7	11.5338	146.4	1.905658	146.5	2.65913	146.6	4.175994	146.7	5.700231	146.7	10.80096
146.5	2.068400009	146.5999908	3.1073	146.7	4.9179	146.8	7.2835	146.8	11.523	146.5	1.903557	146.6	2.65547	146.7	4.16976	146.8	5.691122	146.8	10.78604
146.6000061	2.071899987	146.6999969	3.1071	146.8	4.9125	146.9	7.2787	146.9	11.513	146.6	1.901459	146.7	2.651816	146.8	4.163537	146.9	5.682027	146.9	10.77115
146.7000122	2.071600276	146.8000031	3.1037	146.9	4.9081	147	7.2722	147	11.5054	146.7	1.899362	146.8	2.648168	146.9	4.157322	147	5.672947	147	10.75627
146.7999878	2.07159974	146.8999939	3.1013	147	4.9043	147.1	7.2708	147.1	11.495	146.8	1.897269	146.9	2.644525	147	4.151116	147.1	5.663884	147.1	10.74142
146.8999939	2.069000031	147	3.099	147.1	4.9054	147.2	7.2653	147.2	11.4863	146.9	1.895177	147	2.640886	147.1	4.144921	147.2	5.654836	147.2	10.7266
147	2.067899985	147.0999908	3.0977	147.2	4.905	147.3	7.2613	147.3	11.4767	147	1.893088	147.1	2.637253	147.2	4.138736	147.3	5.645807	147.3	10.7118
147.1000061	2.067000012	147.1999969	3.0987	147.3	4.9044	147.4	7.2583	147.4	11.4649	147.1	1.891001	147.2	2.633625	147.3	4.132562	147.4	5.636791	147.4	10.69702
147.2000122	2.068800327	147.3000031	3.098	147.4	4.8985	147.5	7.2522	147.5	11.4531	147.2	1.888917	147.3	2.630003	147.4	4.126397	147.5	5.627791	147.5	10.68227
147.2999878	2.067599636	147.3999939	3.0961	147.5	4.8925	147.6	7.2494	147.6	11.4461	147.3	1.886835	147.4	2.626385	147.5	4.120241	147.6	5.618806	147.6	10.66754
147.3999939	2.065399941	147.5	3.0947	147.6	4.889	147.7	7.2412	147.7	11.4339	147.4	1.884755	147.5	2.622773	147.6	4.114095	147.7	5.609838	147.7	10.65283
147.5	2.065200021	147.5999908	3.0929	147.7	4.8816	147.8	7.2333	147.8	11.4211	147.5	1.882678	147.6	2.619166	147.7	4.107959	147.8	5.600888	147.8	10.63815
147.6000061	2.064000005	147.6999969	3.09	147.8	4.8759	147.9	7.232899	147.9	11.4129	147.6	1.880602	147.7	2.615563	147.8	4.101834	147.9	5.59195	147.9	10.62349
147.7000122	2.061399898	147.8000031	3.0887	147.9	4.8755	148	7.2286	148	11.3991	147.7	1.87853	147.8	2.611967	147.9	4.095717	148	5.583029	148	10.60885
147.7999878	2.061900096	147.8999939	3.091299	148	4.8721	148.1	7.2232	148.1	11.3889	147.8	1.87646	147.9	2.608375	148	4.08961	148.1	5.574123	148.1	10.59424
147.8999939	2.063499432	148	3.0854	148.1	4.8668	148.2	7.2192	148.2	11.3816	147.9	1.874392	148	2.604789	148.1	4.083513	148.2	5.565232	148.2	10.57965
148	2.061399998	148.0999908	3.0837	148.2	4.8646	148.3	7.2141	148.3	11.3724	148	1.872326	148.1	2.601207	148.2	4.077425	148.3	5.55636	148.3	10.56509
148.1000061	2.061500005	148.1999969	3.082	148.3	4.8598	148.4	7.2073	148.4	11.3641	148.1	1.870262	148.2	2.59763	148.3	4.071349	148.4	5.547501	148.4	10.55054
148.2000122	2.062700045	148.3000031	3.0791	148.4	4.8573	148.5	7.2051	148.5	11.3561	148.2	1.868201	148.3	2.59406	148.4	4.065281	148.5	5.538657	148.5	10.53602
148.2999878	2.061299959	148.3999939	3.0774	148.5	4.854	148.6	7.2024	148.6	11.3491	148.3	1.866143	148.4	2.590494	148.5	4.059222	148.6	5.529829	148.6	10.52152
148.3999939	2.06079988	148.5	3.0791	148.6	4.8548	148.7	7.1998	148.7	11.3376	148.4	1.864086	148.5	2.586933	148.6	4.053173	148.7	5.521016	148.7	10.50705
148.5	2.066299981	148.5999908	3.0818	148.7	4.8505	148.8	7.1958	148.8	11.3283	148.5	1.862032	148.6	2.583376	148.7	4.047134	148.8	5.512221	148.8	10.4926
148.6000061	2.071199985	148.6999969	3.08	148.8	4.8454	148.9	7.1927	148.9	11.3174	148.6	1.85998	148.7	2.579825	148.8	4.041106	148.9	5.503439	148.9	10.47818
148.7000122	2.071800406	148.8000031	3.0776	148.9	4.844	149	7.1895	149	11.3092	148.7	1.857931	148.8	2.57628	148.9	4.035086	149	5.494672	149	10.46377
148.7999878	2.073399559	148.8999939	3.0765	149	4.8433	149.1	7.182	149.1	11.2994	148.8	1.855884	148.9	2.572739	149	4.029075	149.1	5.48592	149.1	10.44939
148.8999939	2.07599967	149	3.0744	149.1	4.8392	149.2	7.1791	149.2	11.29	148.9	1.853839	149	2.569204	149.1	4.023074	149.2	5.477184	149.2	10.43503
149	2.078599989	149.0999908	3.068	149.2	4.8371	149.3	7.1761	149.3	11.2799	149	1.851796	149.1	2.565673	149.2	4.017082	149.3	5.468465	149.3	10.4207
149.1000061	2.077499986	149.1999969	3.0651	149.3	4.8347	149.4	7.1688	149.4	11.269	149.1	1.849756	149.2	2.562147	149.3	4.011102	149.4	5.45976	149.4	10.40638
149.2000122	2.078300112	149.3000031	3.0611	149.4	4.832	149.5	7.1678	149.5	11.2591	149.2	1.847718	149.3	2.558627	149.4	4.005129	149.5	5.451069	149.5	10.39209
149.2999878	2.071499887	149.3999939	3.0581	149.5	4.8304	149.6	7.1661	149.6	11.2525	149.3	1.845683	149.4	2.555111	149.5	3.999166	149.6	5.442393	149.6	10.37782
149.3999939	2.065299884	149.5	3.0574	149.6	4.8255	149.7	7.160801	149.7	11.2416	149.4	1.843649	149.5	2.551601	149.6	3.993212	149.7	5.433733	149.7	10.36358
149.5	2.063300027	149.5999908	3.0586	149.7	4.8177	149.8	7.156999	149.8	11.2328	149.5	1.841618	149.6	2.548095	149.7	3.987268	149.8	5.42509	149.8	10.34936
149.6000061	2.059100015	149.6999969	3.0526	149.8	4.8155	149.9	7.154099	149.9	11.223	149.6	1.839589	149.7	2.544594	149.8	3.981335	149.9	5.41646	149.9	10.33516
149.7000122	2.054600547	149.8000031	3.0504	149.9	4.8173	150	7.1486	150	11.2155	149.7	1.837563	149.8	2.5411	149.9	3.97541	150	5.407845	150	10.32098
149.7999878	2.057999459	149.8999939	3.046599	150	4.8175	150.1	7.1417	150.1	11.1993	149.8	1.835539	149.9	2.537609	150	3.969494	150.1	5.399244	150.1	10.30682
149.8999939	2.062299454	150	3.0451	150.1	4.8102	150.2	7.135	150.2	11.1844	149.9	1.833517	150	2.534123	150.1	3.963587	150.2	5.390659	150.2	10.29269
150	2.063800027	150.0999908	3.0382	150.2	4.8043	150.3	7.1246	150.3	11.172	150	1.831497	150.1	2.530642	150.2	3.95769	150.3	5.382091	150.3	10.27858
150.1000061	2.058000003	150.1999969	3.0324	150.3	4.7996	150.4	7.1135	150.4	11.1594	150.1	1.829479	150.2	2.527166	150.3	3.951804	150.4	5.373536	150.4	10.26449
150.2000122	2.054499897	150.3000031	3.0263	150.4	4.7975	150.5	7.1109	150.5	11.1537	150.2	1.827464	150.3	2.523697	150.4	3.945925	150.5	5.364995	150.5	10.25043
150.2999878	2.047800122	150.3999939	3.0213	150.5	4.7974	150.6	7.1061	150.6	11.1452	150.3	1.825452	150.4	2.520231	150.5	3.940056	150.6	5.356469	150.6	10.23638
150.3999939	2.044500082	150.5	3.0198	150.6	4.7943	150.7	7.103501	150.7	11.1392	150.4	1.823441	150.5	2.51677	150.6	3.934195	150.7	5.347958	150.7	10.22236
150.5	2.045600003	150.5999908	3.0175	150.7	4.7927	150.8	7.102499	150.8	11.135	150.5	1.821433	150.6	2.513314	150.7	3.928345	150.8	5.339464	150.8	10.20837
150.6000061	2.045599961	150.6999969	3.0179	150.8	4.7928	150.9	7.0974	150.9	11.1247	150.6	1.819426	150.7	2.509862	150.8	3.922505	150.9	5.330983	150.9	10.19439
150.7000122	2.04710078	150.8000031	3.0173	150.9	4.7853	151	7.0903	151	11.1122	150.7	1.817423	150.8	2.506417	150.9	3.916673	151	5.322516	151	10.18043
150.7999878	2.051899259	150.8999939	3.019	151	4.7793	151.1	7.0824	151.1	11.0969	150.8	1.815422	150.9	2.502976	151	3.91085	151.1	5.314064	151.1	10.1665
150.8999939	2.051899677	151	3.0103	151.1	4.7719	151.2	7.0745	151.2	11.0858	150.9	1.813422	151	2.499539	151.1	3.905036	151.2	5.305627	151.2	10.15259
151	2.048100054	151.0999908	3.0068	151.2	4.7652	151.3	7.0653	151.3	11.0761	151	1.811425	151.1	2.496108	151.2	3.899231	151.3	5.297206	151.3	10.1387
151.1000061	2.045899951	151.1999969	2.9973	151.3	4.7604	151.4	7.0595	151.4	11.0679	151.1	1.80943	151.2	2.492681	151.3	3.893437	151.4	5.288798	151.4	10.12483
151.2000122	2.040199997	151.3000031	2.9924	151.4	4.7573	151.5	7.0543	151.5	11.0577	151.2	1.807437	151.3	2.489261	151.4	3.887651	151.5	5.280405	151.5	10.11099
151.2999878	2.03959998	151.3999939	2.9916	151.5	4.7557	151.6	7.0467	151.6	11.0481	151.3	1.805448	151.4	2.485844	151.5	3.881874	151.6	5.272026	151.6	10.09716
151.3999939	2.040899785	151.5	2.9948	151.6	4.7539	151.7	7.0414	151.7	11.0369	151.4	1.803459	151.5	2.482432	151.6	3.876106	151.7	5.263661	151.7	10.08336
151.5	2.041799975	151.5999908	2.9949	151.7	4.7514	151.8	7.0361	151.8	11.0257	151.5	1.801473	151.6	2.479025	151.7	3.870347	151.8	5.255313	151.8	10.06958
151.6000061	2.043599969	151.6999969	2.994	151.8	4.747	151.9	7.0314	151.9	11.0188	151.6	1.79949	151.7	2.475622	151.8	3.864599	151.9	5.246978	151.9	10.05583
151.7000122	2.041100188	151.8000031	2.9932	151.9	4.7462	152	7.0219	152	11.0087	151.7	1.797508	151.8	2.472226	151.9	3.858858	152	5.238657	152	10.04209
151.7999878	2.038299774	151.8999939	2.9931	152	4.7389	152.1	7.019	152.1	11	151.8	1.795529	151.9	2.468833	152	3.853126	152.1	5.23035	152.1	10.02837
151.8999939	2.041199679	152	2.988	152.1	4.7393	152.2	7.013901	152.2	10.9939	151.9	1.793552	152	2.465445	152.1	3.847403	152.2	5.222057	152.2	10.01468
152	2.039999957	152.0999908	2.9845	152.2	4.735	152.3	7.011099	152.3	10.9925	152	1.791578	152.1	2.462062	152.2	3.84169	152.3	5.213782	152.3	10.00101
152.1000061	2.037800023	152.1999969	2.9812	152.3	4.737	152.4	7.0081	152.4	10.9867	152.1	1.789605	152.2	2.458684	152.3	3.835987	152.4	5.205518	152.4	9.987358
152.2000122	2.040800832	152.3000031	2.9848	152.4	4.7367	152.5	7.0038	152.5	10.9774	152.2	1.787635	152.3	2.455312	152.4	3.830291	152.5	5.197268	152.5	9.973728
152.2999878	2.044999122	152.3999939	2.9849	152.5	4.7342	152.6	6.9994	152.6	10.9688	152.3	1.785667	152.4	2.451943	152.5	3.824604	152.6	5.189033	152.6	9.960119
152.3999939	2.045399603	152.5	2.9829	152.6	4.7288	152.7	6.9908	152.7	10.9567	152.4	1.783701	152.5	2.44858	152.6	3.818927	152.7	5.180812	152.7	9.946532
152.5	2.047600041	152.5999908	2.9772	152.7	4.7267	152.8	6.9839	152.8	10.9437	152.5	1.781737	152.6	2.445221	152.7	3.813258	152.8	5.172607	152.8	9.93297
152.6000061	2.044799958	152.6999969	2.9704	152.8	4.7239	152.9	6.9797	152.9	10.938	152.6	1.779776	152.7	2.441866	152.8	3.8076	152.9	5.164415	152.9	9.919425
152.7000122	2.040999809	152.8000031	2.9675	152.9	4.7214	153	6.9737	153	10.9269	152.7	1.777817	152.8	2.438518	152.9	3.801949	153	5.156236	153	9.905902
152.7999878	2.036700119	152.8999939	2.9681	153	4.7177	153.1	6.9701	153.1	10.9186	152.8	1.77586	152.9	2.435173	153	3.796307	153.1	5.148072	153.1	9.892399
152.8999939	2.034799703	153	2.9658	153.1	4.716	153.2	6.9628	153.2	10.9113	152.9	1.773905	153	2.431833	153.1	3.790674	153.2	5.139921	153.2	9.878918
153	2.033700021	153.0999908	2.9659	153.2	4.7071	153.3	6.9636	153.3	10.9052	153	1.771952	153.1	2.428498	153.2	3.78505	153.3	5.131787	153.3	9.865462
153.1000061	2.033999995	153.1999969	2.9635	153.3	4.702	153.4	6.956701	153.4	10.8915	153.1	1.770002	153.2	2.425168	153.3	3.779436	153.4	5.123665	153.4	9.852023
153.2000122	2.033800389	153.3000031	2.962	153.4	4.6933	153.5	6.9521	153.5	10.883	153.2	1.768054	153.3	2.421843	153.4	3.773829	153.5	5.115557	153.5	9.838604
153.2999878	2.032599595	153.3999939	2.955701	153.5	4.6926	153.6	6.9504	153.6	10.8787	153.3	1.766108	153.4	2.418522	153.5	3.768232	153.6	5.107462	153.6	9.825207
153.3999939	2.024700512	153.5	2.9572	153.6	4.6946	153.7	6.9493	153.7	10.8705	153.4	1.764164	153.5	2.415206	153.6	3.762643	153.7	5.099382	153.7	9.811831
153.5	2.025799959	153.5999908	2.9567	153.7	4.6945	153.8	6.9451	153.8	10.8616	153.5	1.762222	153.6	2.411894	153.7	3.757063	153.8	5.091318	153.8	9.798479
153.6000061	2.026999964	153.6999969	2.96	153.8	4.693	153.9	6.940299	153.9	10.8552	153.6	1.760283	153.7	2.408587	153.8	3.751493	153.9	5.083265	153.9	9.785145
153.7000122	2.034400339	153.8000031	2.9582	153.9	4.6936	154	6.9333	154	10.8456	153.7	1.758345	153.8	2.405286	153.9	3.74593	154	5.075226	154	9.771831
153.7999878	2.037699631	153.8999939	2.957399	154	4.6849	154.1	6.9295	154.1	10.838	153.8	1.756411	153.9	2.401989	154	3.740377	154.1	5.067201	154.1	9.758538
153.8999939	2.037599464	154	2.9527	154.1	4.6815	154.2	6.9241	154.2	10.8262	153.9	1.754478	154	2.398696	154.1	3.734831	154.2	5.05919	154.2	9.745265
154	2.036899963	154.0999908	2.9532	154.2	4.6774	154.3	6.9211	154.3	10.8188	154	1.752547	154.1	2.395408	154.2	3.729295	154.3	5.051195	154.3	9.732017
154.1000061	2.03809996	154.1999969	2.9488	154.3	4.6782	154.4	6.921299	154.4	10.8124	154.1	1.750618	154.2	2.392124	154.3	3.723769	154.4	5.043211	154.4	9.718786
154.2000122	2.034500407	154.3000031	2.9468	154.4	4.6816	154.5	6.9203	154.5	10.8042	154.2	1.748691	154.3	2.388847	154.4	3.71825	154.5	5.035241	154.5	9.705576
154.2999878	2.03209963	154.3999939	2.950699	154.5	4.6798	154.6	6.9154	154.6	10.7974	154.3	1.746768	154.4	2.385572	154.5	3.71274	154.6	5.027285	154.6	9.692386
154.3999939	2.037199389	154.5	2.9533	154.6	4.679	154.7	6.9064	154.7	10.7887	154.4	1.744845	154.5	2.382303	154.6	3.707238	154.7	5.019342	154.7	9.679216
154.5	2.03780001	154.5999908	2.953	154.7	4.6728	154.8	6.9009	154.8	10.7814	154.5	1.742925	154.6	2.379038	154.7	3.701745	154.8	5.011416	154.8	9.666071
154.6000061	2.036299987	154.6999969	2.9488	154.8	4.6687	154.9	6.8975	154.9	10.7758	154.6	1.741008	154.7	2.375778	154.8	3.696262	154.9	5.003501	154.9	9.652942
154.7000122	2.034000096	154.8000031	2.949	154.9	4.6644	155	6.8921	155	10.7689	154.7	1.739092	154.8	2.372523	154.9	3.690787	155	4.995599	155	9.639834
154.7999878	2.032799924	154.8999939	2.9505	155	4.6601	155.1	6.8861	155.1	10.7643	154.8	1.737179	154.9	2.369272	155	3.685319	155.1	4.98771	155.1	9.626746
154.8999939	2.034499815	155	2.9483	155.1	4.6565	155.2	6.8823	155.2	10.759	154.9	1.735267	155	2.366026	155.1	3.679861	155.2	4.979836	155.2	9.613678
155	2.032799994	155.0999908	2.9501	155.2	4.6542	155.3	6.877	155.3	10.7528	155	1.733358	155.1	2.362784	155.2	3.674411	155.3	4.971977	155.3	9.600635
155.1000061	2.028299941	155.1999969	2.9496	155.3	4.6473	155.4	6.8663	155.4	10.7414	155.1	1.731451	155.2	2.359547	155.3	3.668971	155.4	4.964129	155.4	9.587608
155.2000122	2.028899916	155.3000031	2.9471	155.4	4.6398	155.5	6.8606	155.5	10.7331	155.2	1.729546	155.3	2.356316	155.4	3.663538	155.5	4.956295	155.5	9.574601
155.2999878	2.028500071	155.3999939	2.9407	155.5	4.6387	155.6	6.8549	155.6	10.7242	155.3	1.727644	155.4	2.353088	155.5	3.658113	155.6	4.948474	155.6	9.561614
155.3999939	2.030800441	155.5	2.9411	155.6	4.635	155.7	6.8504	155.7	10.7147	155.4	1.725743	155.5	2.349864	155.6	3.652698	155.7	4.940667	155.7	9.548647
155.5	2.035600041	155.5999908	2.9375	155.7	4.6325	155.8	6.8471	155.8	10.7068	155.5	1.723844	155.6	2.346645	155.7	3.64729	155.8	4.932875	155.8	9.535704
155.6000061	2.031199955	155.6999969	2.9359	155.8	4.6321	155.9	6.8426	155.9	10.6991	155.6	1.721948	155.7	2.343431	155.8	3.641893	155.9	4.925094	155.9	9.522778
155.7000122	2.035200462	155.8000031	2.9361	155.9	4.6272	156	6.8323	156	10.6912	155.7	1.720053	155.8	2.340222	155.9	3.636502	156	4.917327	156	9.509871
155.7999878	2.036499513	155.8999939	2.9347	156	4.6173	156.1	6.8256	156.1	10.6824	155.8	1.718162	155.9	2.337017	156	3.63112	156.1	4.909572	156.1	9.496984
155.8999939	2.031000112	156	2.93	156.1	4.6125	156.2	6.8169	156.2	10.6729	155.9	1.716272	156	2.333817	156.1	3.625747	156.2	4.901831	156.2	9.484118
156	2.022799949	156.0999908	2.9255	156.2	4.6099	156.3	6.8081	156.3	10.6637	156	1.714384	156.1	2.330621	156.2	3.620381	156.3	4.894106	156.3	9.471275
156.1000061	2.020199996	156.1999969	2.9228	156.3	4.6087	156.4	6.8056	156.4	10.655	156.1	1.712498	156.2	2.327429	156.3	3.615026	156.4	4.886392	156.4	9.458448
156.2000122	2.020699943	156.3000031	2.9208	156.4	4.6047	156.5	6.8061	156.5	10.649	156.2	1.710614	156.3	2.324243	156.4	3.609678	156.5	4.87869	156.5	9.44564
156.2999878	2.018800065	156.3999939	2.9208	156.5	4.6028	156.6	6.7991	156.6	10.6383	156.3	1.708733	156.4	2.321061	156.5	3.604338	156.6	4.871002	156.6	9.432853

156.3999939	2.018499979	156.5	2.924	156.6	4.5971	156.7	6.7982	156.7	10.6314	156.4	1.706854	156.5	2.317883	156.6	3.599006	156.7	4.863327	156.7	9.420085
156.5	2.019799953	156.5999908	2.9222	156.7	4.5948	156.8	6.7917	156.8	10.6179	156.5	1.704976	156.6	2.314709	156.7	3.593683	156.8	4.855668	156.8	9.407341
156.6000061	2.015899973	156.6999969	2.9257	156.8	4.5898	156.9	6.7834	156.9	10.6084	156.6	1.703101	156.7	2.31154	156.8	3.588369	156.9	4.848019	156.9	9.394612
156.7000122	2.019099692	156.8000031	2.9219	156.9	4.5889	157	6.777	157	10.601	156.7	1.701228	156.8	2.308377	156.9	3.583063	157	4.840383	157	9.381903
156.7999878	2.017400281	156.8999939	2.921	157	4.5882	157.1	6.7711	157.1	10.5922	156.8	1.699357	156.9	2.305217	157	3.577764	157.1	4.83276	157.1	9.369214
156.8999939	2.014000042	157	2.9181	157.1	4.587	157.2	6.766501	157.2	10.5821	156.9	1.697488	157	2.302061	157.1	3.572474	157.2	4.825151	157.2	9.356544
157	2.010999946	157.0999908	2.9222	157.2	4.5851	157.3	6.769099	157.3	10.5791	157	1.695621	157.1	2.29891	157.2	3.567192	157.3	4.817556	157.3	9.343898
157.1000061	2.009199969	157.1999969	2.9231	157.3	4.5848	157.4	6.7704	157.4	10.5714	157.1	1.693757	157.2	2.295763	157.3	3.56192	157.4	4.809972	157.4	9.331267
157.2000122	2.006601081	157.3000031	2.9311	157.4	4.5824	157.5	6.7708	157.5	10.5634	157.2	1.691894	157.3	2.292622	157.4	3.556655	157.5	4.802401	157.5	9.318656
157.2999878	2.01409899	157.3999939	2.933	157.5	4.5804	157.6	6.7685	157.6	10.56	157.3	1.690034	157.4	2.289484	157.5	3.551398	157.6	4.794843	157.6	9.306064
157.3999939	2.015599591	157.5	2.9295	157.6	4.5813	157.7	6.7653	157.7	10.5554	157.4	1.688175	157.5	2.286351	157.6	3.546149	157.7	4.787298	157.7	9.293491
157.5	2.012999945	157.5999908	2.9304	157.7	4.5796	157.8	6.7593	157.8	10.5434	157.5	1.686319	157.6	2.283222	157.7	3.540908	157.8	4.779768	157.8	9.280942
157.6000061	2.011699989	157.6999969	2.9299	157.8	4.5755	157.9	6.7522	157.9	10.5356	157.6	1.684465	157.7	2.280098	157.8	3.535678	157.9	4.772249	157.9	9.268408
157.7000122	2.007799728	157.8000031	2.9259	157.9	4.5742	158	6.7512	158	10.5273	157.7	1.682612	157.8	2.276979	157.9	3.530453	158	4.764742	158	9.255893
157.7999878	2.00240035	157.8999939	2.9249	158	4.5753	158.1	6.7495	158.1	10.5164	157.8	1.680763	157.9	2.273863	158	3.525237	158.1	4.757248	158.1	9.243398
157.8999939	2.001800151	158	2.9258	158.1	4.5739	158.2	6.7409	158.2	10.507	157.9	1.678915	158	2.270752	158.1	3.520029	158.2	4.749767	158.2	9.230921
158	2.000699969	158.0999908	2.9266	158.2	4.569	158.3	6.7367	158.3	10.5008	158	1.677068	158.1	2.267645	158.2	3.514829	158.3	4.742301	158.3	9.218468
158.1000061	2.000299994	158.1999969	2.9236	158.3	4.5675	158.4	6.7317	158.4	10.4907	158.1	1.675224	158.2	2.264543	158.3	3.509639	158.4	4.734845	158.4	9.206029
158.2000122	1.998900238	158.3000031	2.9203	158.4	4.5663	158.5	6.7277	158.5	10.4832	158.2	1.673383	158.3	2.261446	158.4	3.504455	158.5	4.727402	158.5	9.19361
158.2999878	1.997899719	158.3999939	2.9166	158.5	4.5681	158.6	6.7249	158.6	10.4778	158.3	1.671543	158.4	2.258352	158.5	3.49928	158.6	4.719972	158.6	9.18121
158.3999939	1.997599835	158.5	2.9167	158.6	4.5736	158.7	6.7207	158.7	10.4688	158.4	1.669706	158.5	2.255263	158.6	3.494112	158.7	4.712554	158.7	9.168829
158.5	1.999800045	158.5999908	2.9146	158.7	4.5697	158.8	6.7164	158.8	10.4607	158.5	1.66787	158.6	2.252178	158.7	3.488953	158.8	4.705151	158.8	9.156471
158.6000061	2.000600049	158.6999969	2.9142	158.8	4.5644	158.9	6.7147	158.9	10.4563	158.6	1.666036	158.7	2.249098	158.8	3.483803	158.9	4.697759	158.9	9.144127
158.7000122	1.999000218	158.8000031	2.9162	158.9	4.5647	159	6.7094	159	10.4476	158.7	1.664205	158.8	2.246022	158.9	3.47866	159	4.690379	159	9.131803
158.7999878	1.999399804	158.8999939	2.917	159	4.5628	159.1	6.7051	159.1	10.4425	158.8	1.662376	158.9	2.242951	159	3.473524	159.1	4.683011	159.1	9.119497
158.8999939	2.000499607	159	2.9159	159.1	4.5609	159.2	6.7002	159.2	10.4348	158.9	1.660548	159	2.239883	159.1	3.468397	159.2	4.675656	159.2	9.107211
159	2.003500031	159.0999908	2.9153	159.2	4.5572	159.3	6.7001	159.3	10.4246	159	1.658723	159.1	2.23682	159.2	3.463278	159.3	4.668316	159.3	9.094946
159.1000061	2.002900047	159.1999969	2.9133	159.3	4.5568	159.4	6.690201	159.4	10.4088	159.1	1.656899	159.2	2.233761	159.3	3.458168	159.4	4.660986	159.4	9.082697
159.2000122	2.000400299	159.3000031	2.9139	159.4	4.5498	159.5	6.6862	159.5	10.4016	159.2	1.655078	159.3	2.230708	159.4	3.453064	159.5	4.653668	159.5	9.070467
159.2999878	2.000699677	159.3999939	2.906601	159.5	4.5507	159.6	6.6817	159.6	10.3929	159.3	1.653259	159.4	2.227658	159.5	3.447969	159.6	4.646363	159.6	9.058255
159.3999939	1.998500739	159.5	2.9094	159.6	4.5473	159.7	6.6763	159.7	10.3805	159.4	1.651442	159.5	2.224612	159.6	3.442881	159.7	4.63907	159.7	9.046062
159.5	2.001500047	159.5999908	2.9067	159.7	4.5419	159.8	6.6705	159.8	10.3735	159.5	1.649627	159.6	2.22157	159.7	3.437802	159.8	4.631792	159.8	9.033891
159.6000061	2.003300025	159.6999969	2.9007	159.8	4.5385	159.9	6.670299	159.9	10.3727	159.6	1.647813	159.7	2.218533	159.8	3.432732	159.9	4.624524	159.9	9.021735
159.7000122	1.999699723	159.8000031	2.8942	159.9	4.5398	160	6.6692	160	10.3684	159.7	1.646002	159.8	2.215501	159.9	3.427668	160	4.617268	160	9.009597
159.7999878	1.994600292	159.8999939	2.895899	160	4.5394	160.1	6.6674	160.1	10.3634	159.8	1.644194	159.9	2.212472	160	3.422612	160.1	4.610024	160.1	8.997478
159.8999939	1.996399389	160	2.899	160.1	4.5431	160.2	6.6616	160.2	10.3562	159.9	1.642387	160	2.209448	160.1	3.417564	160.2	4.602793	160.2	8.985377
160	1.998099943	160.0999908	2.901799	160.2	4.5404	160.3	6.6515	160.3	10.3452	160	1.640581	160.1	2.206428	160.2	3.412524	160.3	4.595576	160.3	8.973299
160.1000061	1.999100044	160.1999969	2.903	160.3	4.5352	160.4	6.6463	160.4	10.3334	160.1	1.638778	160.2	2.203412	160.3	3.407493	160.4	4.588369	160.4	8.961235
160.2000122	1.99679968	160.3000031	2.9002	160.4	4.5292	160.5	6.6387	160.5	10.3229	160.2	1.636977	160.3	2.200401	160.4	3.402468	160.5	4.581175	160.5	8.949189
160.2999878	1.993400355	160.3999939	2.8987	160.5	4.522	160.6	6.6339	160.6	10.3154	160.3	1.635179	160.4	2.197394	160.5	3.397452	160.6	4.573992	160.6	8.937162
160.3999939	1.994000156	160.5	2.8941	160.6	4.5169	160.7	6.630801	160.7	10.3065	160.4	1.633382	160.5	2.194391	160.6	3.392443	160.7	4.566822	160.7	8.925153
160.5	1.989700006	160.5999908	2.8923	160.7	4.5175	160.8	6.630399	160.8	10.2985	160.5	1.631587	160.6	2.191392	160.7	3.387442	160.8	4.559666	160.8	8.913166
160.6000061	1.990699984	160.6999969	2.8895	160.8	4.52	160.9	6.626699	160.9	10.2931	160.6	1.629794	160.7	2.188397	160.8	3.38245	160.9	4.55252	160.9	8.901194
160.7000122	1.991900537	160.8000031	2.8904	160.9	4.5234	161	6.6217	161	10.2848	160.7	1.628003	160.8	2.185408	160.9	3.377464	161	4.545386	161	8.88924
160.7999878	1.992799396	160.8999939	2.891099	161	4.5249	161.1	6.6185	161.1	10.2781	160.8	1.626214	160.9	2.182421	161	3.372486	161.1	4.538264	161.1	8.877304
160.8999939	1.995599325	161	2.8848	161.1	4.5263	161.2	6.6171	161.2	10.2709	160.9	1.624427	161	2.179439	161.1	3.367516	161.2	4.531154	161.2	8.865386
161	1.989900037	161.0999908	2.8835	161.2	4.5284	161.3	6.6133	161.3	10.2623	161	1.622643	161.1	2.176462	161.2	3.362554	161.3	4.524058	161.3	8.85349
161.1000061	1.992199998	161.1999969	2.885	161.3	4.5293	161.4	6.6094	161.4	10.2543	161.1	1.62086	161.2	2.173488	161.3	3.357601	161.4	4.516972	161.4	8.841608
161.2000122	1.99799995	161.3000031	2.8856	161.4	4.5311	161.5	6.6128	161.5	10.2534	161.2	1.619079	161.3	2.17052	161.4	3.352654	161.5	4.509898	161.5	8.829744
161.2999878	1.99639995	161.3999939	2.8862	161.5	4.5357	161.6	6.6096	161.6	10.2457	161.3	1.6173	161.4	2.167554	161.5	3.347714	161.6	4.502836	161.6	8.817898
161.3999939	1.995799999	161.5	2.8914	161.6	4.5305	161.7	6.6048	161.7	10.2367	161.4	1.615523	161.5	2.164593	161.6	3.342783	161.7	4.495785	161.7	8.80607
161.5	2.001200041	161.5999908	2.8888	161.7	4.5281	161.8	6.6026	161.8	10.2292	161.5	1.613748	161.6	2.161636	161.7	3.337859	161.8	4.488749	161.8	8.794264
161.6000061	1.998300008	161.6999969	2.8883	161.8	4.5284	161.9	6.597401	161.9	10.2169	161.6	1.611975	161.7	2.158684	161.8	3.332944	161.9	4.481723	161.9	8.782472
161.7000122	1.999600084	161.8000031	2.8832	161.9	4.5212	162	6.594	162	10.2086	161.7	1.610204	161.8	2.155736	161.9	3.328035	162	4.474708	162	8.770697
161.7999878	1.995199842	161.8999939	2.878701	162	4.5223	162.1	6.59	162.1	10.1974	161.8	1.608436	161.9	2.152792	162	3.323134	162.1	4.467705	162.1	8.758941
161.8999939	1.992800503	162	2.8804	162.1	4.5215	162.2	6.5888	162.2	10.1885	161.9	1.606669	162	2.149852	162.1	3.318241	162.2	4.460714	162.2	8.747202
162	1.997899998	162.0999908	2.8808	162.2	4.5157	162.3	6.5866	162.3	10.1786	162	1.604904	162.1	2.146916	162.2	3.313355	162.3	4.453737	162.3	8.735485
162.1000061	2.000599998	162.1999969	2.8806	162.3	4.5129	162.4	6.5856	162.4	10.1703	162.1	1.60314	162.2	2.143984	162.3	3.308478	162.4	4.44677	162.4	8.723781
162.2000122	2.001199809	162.3000031	2.878	162.4	4.5091	162.5	6.5805	162.5	10.1614	162.2	1.601379	162.3	2.141057	162.4	3.303607	162.5	4.439814	162.5	8.712096
162.2999878	2.002400206	162.3999939	2.8789	162.5	4.5099	162.6	6.5728	162.6	10.1502	162.3	1.599621	162.4	2.138133	162.5	3.298744	162.6	4.43287	162.6	8.700428
162.3999939	2.003999915	162.5	2.877	162.6	4.5028	162.7	6.5678	162.7	10.1392	162.4	1.597864	162.5	2.135214	162.6	3.293888	162.7	4.425938	162.7	8.688778
162.5	2.003699978	162.5999908	2.8702	162.7	4.4989	162.8	6.5597	162.8	10.1296	162.5	1.596108	162.6	2.132298	162.7	3.28904	162.8	4.419019	162.8	8.677148
162.6000061	2.00149999	162.6999969	2.8686	162.8	4.4966	162.9	6.5538	162.9	10.1187	162.6	1.594355	162.7	2.129387	162.8	3.284201	162.9	4.41211	162.9	8.665533
162.7000122	2.000899705	162.8000031	2.8642	162.9	4.4876	163	6.55	163	10.1088	162.7	1.592604	162.8	2.12648	162.9	3.279368	163	4.405212	163	8.653936
162.7999878	1.996500257	162.8999939	2.8633	163	4.4812	163.1	6.5487	163.1	10.1008	162.8	1.590855	162.9	2.123577	163	3.274542	163.1	4.398326	163.1	8.642355
162.8999939	1.991300267	163	2.8643	163.1	4.477	163.2	6.546601	163.2	10.0916	162.9	1.589108	163	2.120678	163.1	3.269724	163.2	4.391452	163.2	8.630793
163	1.991000031	163.0999908	2.8625	163.2	4.471	163.3	6.545999	163.3	10.0877	163	1.587362	163.1	2.117783	163.2	3.264913	163.3	4.384591	163.3	8.619251
163.1000061	1.993899972	163.1999969	2.8594	163.3	4.4713	163.4	6.5398	163.4	10.0765	163.1	1.585619	163.2	2.114892	163.3	3.260112	163.4	4.37774	163.4	8.607723
163.2000122	1.994300559	163.3000031	2.8597	163.4	4.4706	163.5	6.5354	163.5	10.0648	163.2	1.583877	163.3	2.112007	163.4	3.255316	163.5	4.3709	163.5	8.596212
163.2999878	1.996099454	163.3999939	2.8557	163.5	4.4708	163.6	6.5275	163.6	10.0563	163.3	1.582138	163.4	2.109124	163.5	3.250527	163.6	4.364072	163.6	8.584719
163.3999939	1.992900473	163.5	2.8495	163.6	4.4688	163.7	6.5204	163.7	10.0466	163.4	1.580401	163.5	2.106245	163.6	3.245746	163.7	4.357255	163.7	8.573243
163.5	1.992499999	163.5999908	2.8485	163.7	4.4665	163.8	6.5168	163.8	10.0382	163.5	1.578665	163.6	2.10337	163.7	3.240973	163.8	4.350452	163.8	8.561788
163.6000061	1.992599959	163.6999969	2.845	163.8	4.4633	163.9	6.517299	163.9	10.0336	163.6	1.576931	163.7	2.1005	163.8	3.236208	163.9	4.343658	163.9	8.550347
163.7000122	1.992099909	163.8000031	2.842	163.9	4.4658	164	6.5173	164	10.0267	163.7	1.575199	163.8	2.097634	163.9	3.231449	164	4.336875	164	8.538922
163.7999878	1.992200098	163.8999939	2.844699	164	4.4662	164.1	6.5097	164.1	10.0178	163.8	1.57347	163.9	2.094772	164	3.226698	164.1	4.330104	164.1	8.527515
163.8999939	1.994799258	164	2.8448	164.1	4.4615	164.2	6.5047	164.2	10.0101	163.9	1.571742	164	2.091913	164.1	3.221954	164.2	4.323344	164.2	8.516125
164	1.993100044	164.0999908	2.8432	164.2	4.4587	164.3	6.5018	164.3	10.0032	164	1.570016	164.1	2.089059	164.2	3.217217	164.3	4.316598	164.3	8.504756
164.1000061	1.989800051	164.1999969	2.8395	164.3	4.4554	164.4	6.4936	164.4	9.9926	164.1	1.568292	164.2	2.086208	164.3	3.212489	164.4	4.309861	164.4	8.4934
164.2000122	1.986300262	164.3000031	2.8405	164.4	4.4518	164.5	6.4925	164.5	9.987	164.2	1.56657	164.3	2.083363	164.4	3.207767	164.5	4.303135	164.5	8.482061
164.2999878	1.984699796	164.3999939	2.8404	164.5	4.4499	164.6	6.4907	164.6	9.9814	164.3	1.56485	164.4	2.08052	164.5	3.203052	164.6	4.29642	164.6	8.470739
164.3999939	1.980600282	164.5	2.8405	164.6	4.4454	164.7	6.4825	164.7	9.9729	164.4	1.563132	164.5	2.077682	164.6	3.198344	164.7	4.289717	164.7	8.459435
164.5	1.979900001	164.5999908	2.8421	164.7	4.4389	164.8	6.4775	164.8	9.9676	164.5	1.561416	164.6	2.074847	164.7	3.193644	164.8	4.283027	164.8	8.44815
164.6000061	1.978299945	164.6999969	2.8419	164.8	4.4401	164.9	6.4705	164.9	9.96	164.6	1.559701	164.7	2.072017	164.8	3.188952	164.9	4.276346	164.9	8.436879
164.7000122	1.975500331	164.8000031	2.8417	164.9	4.439	165	6.4561	165	9.952	164.7	1.557989	164.8	2.069191	164.9	3.184267	165	4.269676	165	8.425625
164.7999878	1.978499693	164.8999939	2.8378	165	4.4323	165.1	6.4516	165.1	9.9439	164.8	1.556279	164.9	2.066369	165	3.179588	165.1	4.263017	165.1	8.414388
164.8999939	1.978099981	165	2.8354	165.1	4.4295	165.2	6.449	165.2	9.9367	164.9	1.55457	165	2.06355	165.1	3.174917	165.2	4.25637	165.2	8.403168
165	1.975100007	165.0999908	2.8365	165.2	4.4273	165.3	6.4419	165.3	9.9262	165	1.552863	165.1	2.060736	165.2	3.170253	165.3	4.249736	165.3	8.391968
165.1000061	1.972100007	165.1999969	2.8371	165.3	4.4234	165.4	6.4348	165.4	9.9174	165.1	1.551158	165.2	2.057925	165.3	3.165597	165.4	4.24311	165.4	8.380781
165.2000122	1.975299809	165.3000031	2.8338	165.4	4.4176	165.5	6.4281	165.5	9.9108	165.2	1.549455	165.3	2.055119	165.4	3.160947	165.5	4.236496	165.5	8.369611
165.2999878	1.976400206	165.3999939	2.83	165.5	4.4129	165.6	6.4196	165.6	9.9029	165.3	1.547755	165.4	2.052317	165.5	3.156305	165.6	4.229893	165.6	8.358457
165.3999939	1.97320009	165.5	2.8257	165.6	4.4061	165.7	6.4103	165.7	9.8933	165.4	1.546056	165.5	2.049518	165.6	3.15167	165.7	4.223301	165.7	8.347321
165.5	1.969699971	165.5999908	2.8202	165.7	4.4026	165.8	6.403	165.8	9.8856	165.5	1.544358	165.6	2.046723	165.7	3.147041	165.8	4.216722	165.8	8.336204
165.6000061	1.971900043	165.6999969	2.8152	165.8	4.3995	165.9	6.3969	165.9	9.8765	165.6	1.542663	165.7	2.043932	165.8	3.142422	165.9	4.210152	165.9	8.325101
165.7000122	1.967400069	165.8000031	2.8148	165.9	4.3969	166	6.3922	166	9.8661	165.7	1.54097	165.8	2.041146	165.9	3.137808	166	4.203593	166	8.314014
165.7999878	1.969299999	165.8999939	2.8142	166	4.3912	166.1	6.3884	166.1	9.8584	165.8	1.539279	165.9	2.038363	166	3.133201	166.1	4.197045	166.1	8.302943
165.8999939	1.96699993	166	2.8156	166.1	4.387	166.2	6.3841	166.2	9.8486	165.9	1.537589	166	2.035584	166.1	3.128601	166.2	4.190507	166.2	8.29189
166	1.966300033	166.0999908	2.8123	166.2	4.3832	166.3	6.3795	166.3	9.8401	166	1.535901	166.1	2.032808	166.2	3.124008	166.3	4.183983	166.3	8.280856
166.1000061	1.965599999	166.1999969	2.8057	166.3	4.3823	166.4	6.3773	166.4	9.8348	166.1	1.534215	166.2	2.030037	166.3	3.119424	166.4	4.177468	166.4	8.269835
166.2000122	1.963700128	166.3000031	2.8035	166.4	4.3826	166.5	6.3714	166.5	9.8301	166.2	1.532531	166.3	2.02727	166.4	3.114846	166.5	4.170963	166.5	8.25883
166.2999878	1.96339987	166.3999939	2.8033	166.5	4.381	166.6	6.362	166.6	9.8219	166.3	1.53085	166.4	2.024507	166.5	3.110274	166.6	4.164469	166.6	8.247842
166.3999939	1.963499639	166.5	2.8023	166.6	4.3786	166.7	6.3548	166.7	9.8123	166.4	1.529169	166.5	2.021747	166.6	3.10571	166.7	4.157986	166.7	8.236871
166.5	1.962699974	166.5999908	2.8033	166.7	4.3747	166.8	6.3505	166.8	9.8045	166.5	1.527491	166.6	2.018991	166.7	3.101152	166.8	4.151516	166.8	8.225919
166.6000061	1.962600057	166.6999969	2.801	166.8	4.3655	166.9	6.3446	166.9	9.7968	166.6	1.525814	166.7	2.016239	166.8	3.096603	166.9	4.145055	166.9	8.21498
166.7000122	1.959999719	166.8000031	2.8024	166.9	4.359	167	6.3377	167	9.7868	166.7	1.52414	166.8	2.013492	166.9	3.09206	167	4.138604	167	8.204057
166.7999878	1.956100185	166.8999939	2.8026	167	4.3551	167.1	6.3336	167.1	9.7755	166.8	1.522468	166.9	2.010748	167	3.087524	167.1	4.132164	167.1	8.19315
166.8999939	1.955600069	167	2.8005	167.1	4.3527	167.2	6.3284	167.2	9.7685	166.9	1.520797	167	2.008007	167.1	3.082994	167.2	4.125735	167.2	8.18226
167	1.954300045	167.0999908	2.8018	167.2	4.3482	167.3	6.3213	167.3	9.7585	167	1.519128	167.1	2.005271	167.2	3.078472	167.3	4.119319	167.3	8.171389
167.1000061	1.958299962	167.1999969	2.8016	167.3	4.3403	167.4	6.3172	167.4	9.749	167.1	1.517461	167.2	2.002538	167.3	3.073958	167.4	4.112911	167.4	8.160531
167.2000122	1.95749966	167.3000031	2.7948	167.4	4.3366	167.5	6.3117	167.5	9.742	167.2	1.515795	167.3	1.99981	167.4	3.069449	167.5	4.106514	167.5	8.14969
167.2999878	1.953200368	167.3999939	2.7942	167.5	4.3316	167.6	6.3039	167.6	9.7297	167.3	1.514132	167.4	1.997085	167.5	3.064948	167.6	4.100127	167.6	8.138864
167.3999939	1.952299819	167.5	2.7926	167.6	4.3255	167.7	6.3007	167.7	9.7242	167.4	1.512471	167.5	1.994364	167.6	3.060453	167.7	4.093752	167.7	8.128054
167.5	1.948400034	167.5999908	2.7898	167.7	4.3211	167.8	6.2946	167.8	9.7176	167.5	1.510811	167.6	1.991646	167.7	3.055965	167.8	4.087388	167.8	8.117264
167.6000061	1.94130001	167.6999969	2.7877	167.8	4.3183	167.9	6.2891	167.9	9.7117	167.6	1.509153	167.7	1.988933	167.8	3.051486	167.9	4.081034	167.9	8.106487
167.7000122	1.939900268	167.8000031	2.7873	167.9	4.3148	168	6.2808	168	9.7095	167.7	1.507497	167.8	1.986224	167.9	3.047012	168	4.07469	168	8.095725
167.7999878	1.938299699	167.8999939	2.7887	168	4.3148	168.1	6.275	168.1	9.7014	167.8	1.505844	167.9	1.983518	168	3.042545	168.1	4.068357	168.1	8.084979

167.8999939	1.937599902	168	2.7889	168.1	4.3076	168.2	6.2679	168.2	9.6968	167.9	1.504191	168	1.980816	168.1	3.038085	168.2	4.062034	168.2	8.07425
168	1.937999962	168.0999908	2.7835	168.2	4.3041	168.3	6.2606	168.3	9.6891	168	1.502541	168.1	1.978117	168.2	3.033631	168.3	4.055723	168.3	8.063539
168.1000061	1.933099957	168.1999969	2.7835	168.3	4.3054	168.4	6.2539	168.4	9.6805	168.1	1.500892	168.2	1.975423	168.3	3.029186	168.4	4.049421	168.4	8.052841
168.2000122	1.936500071	168.3000031	2.784	168.4	4.3015	168.5	6.2464	168.5	9.6716	168.2	1.499245	168.3	1.972733	168.4	3.024746	168.5	4.043129	168.5	8.042159
168.2999878	1.937099899	168.3999939	2.7813	168.5	4.2983	168.6	6.2418	168.6	9.6617	168.3	1.497601	168.4	1.970045	168.5	3.020314	168.6	4.036848	168.6	8.031493
168.3999939	1.934000087	168.5	2.7779	168.6	4.2936	168.7	6.2399	168.7	9.6579	168.4	1.495958	168.5	1.967362	168.6	3.015888	168.7	4.030578	168.7	8.020843
168.5	1.93140004	168.5999908	2.7776	168.7	4.2961	168.8	6.2355	168.8	9.6524	168.5	1.494317	168.6	1.964683	168.7	3.011468	168.8	4.024319	168.8	8.010211
168.6000061	1.930699944	168.6999969	2.7813	168.8	4.2969	168.9	6.23	168.9	9.6437	168.6	1.492677	168.7	1.962007	168.8	3.007057	168.9	4.018069	168.9	7.999593
168.7000122	1.9336002	168.8000031	2.7823	168.9	4.295	169	6.2271	169	9.6405	168.7	1.491039	168.8	1.959336	168.9	3.002652	169	4.01183	169	7.988989
168.7999878	1.931999751	168.8999939	2.7803	169	4.2966	169.1	6.2226	169.1	9.6339	168.8	1.489404	168.9	1.956667	169	2.998253	169.1	4.005601	169.1	7.978402
168.8999939	1.92909992	169	2.7802	169.1	4.2962	169.2	6.2131	169.2	9.6265	168.9	1.48777	169	1.954003	169.1	2.993861	169.2	3.999382	169.2	7.96783
169	1.929099977	169.0999908	2.778	169.2	4.2946	169.3	6.2087	169.3	9.6213	169	1.486138	169.1	1.951342	169.2	2.989475	169.3	3.993176	169.3	7.957277
169.1000061	1.927900023	169.1999969	2.7761	169.3	4.2953	169.4	6.2026	169.4	9.6141	169.1	1.484508	169.2	1.948685	169.3	2.985098	169.4	3.986977	169.4	7.946736
169.2000122	1.924900063	169.3000031	2.7761	169.4	4.2941	169.5	6.1975	169.5	9.6041	169.2	1.482879	169.3	1.946032	169.4	2.980726	169.5	3.980789	169.5	7.936211
169.2999878	1.927399838	169.3999939	2.7759	169.5	4.291	169.6	6.197	169.6	9.5994	169.3	1.481253	169.4	1.943383	169.5	2.976361	169.6	3.974612	169.6	7.925702
169.3999939	1.927899868	169.5	2.7754	169.6	4.2874	169.7	6.1975	169.7	9.5938	169.4	1.479628	169.5	1.940737	169.6	2.972002	169.7	3.968444	169.7	7.915208
169.5	1.926900051	169.5999908	2.7783	169.7	4.2863	169.8	6.195	169.8	9.587	169.5	1.478005	169.6	1.938095	169.7	2.96765	169.8	3.962289	169.8	7.904732
169.6000061	1.928100028	169.6999969	2.7825	169.8	4.2834	169.9	6.1902	169.9	9.5799	169.6	1.476384	169.7	1.935456	169.8	2.963307	169.9	3.956142	169.9	7.894269
169.7000122	1.93050004	169.8000031	2.7795	169.9	4.2805	170	6.1794	170	9.5695	169.7	1.474765	169.8	1.932822	169.9	2.958968	170	3.950005	170	7.883821
169.7999878	1.92919997	169.8999939	2.7778	170	4.2734	170.1	6.1797	170.1	9.5656	169.8	1.473148	169.9	1.930191	170	2.954636	170.1	3.943878	170.1	7.873389
169.8999939	1.933599845	170	2.771	170.1	4.2732	170.2	6.178001	170.2	9.560001	169.9	1.471532	170	1.927563	170.1	2.950311	170.2	3.937761	170.2	7.862972
170	1.933300052	170.0999908	2.7711	170.2	4.2723	170.3	6.177299	170.3	9.557299	170	1.469918	170.1	1.92494	170.2	2.945992	170.3	3.931657	170.3	7.852574
170.1000061	1.939100059	170.1999969	2.77	170.3	4.2727	170.4	6.1721	170.4	9.5486	170.1	1.468306	170.2	1.922319	170.3	2.941682	170.4	3.92556	170.4	7.842188
170.2000122	1.942100577	170.3000031	2.7735	170.4	4.2678	170.5	6.1723	170.5	9.5403	170.2	1.466695	170.3	1.919704	170.4	2.937376	170.5	3.919474	170.5	7.831817
170.2999878	1.945999403	170.3999939	2.7693	170.5	4.2676	170.6	6.1673	170.6	9.5314	170.3	1.465087	170.4	1.917091	170.5	2.933078	170.6	3.913398	170.6	7.821461
170.3999939	1.947000068	170.5	2.7698	170.6	4.2624	170.7	6.1669	170.7	9.5235	170.4	1.46348	170.5	1.914482	170.6	2.928785	170.7	3.907332	170.7	7.811121
170.5	1.950500045	170.5999908	2.7674	170.7	4.2631	170.8	6.1628	170.8	9.5136	170.5	1.461875	170.6	1.911876	170.7	2.9245	170.8	3.901277	170.8	7.800799
170.6000061	1.951099965	170.6999969	2.7656	170.8	4.263	170.9	6.158	170.9	9.5067	170.6	1.460272	170.7	1.909275	170.8	2.920222	170.9	3.895231	170.9	7.790488
170.7000122	1.950700079	170.8000031	2.7636	170.9	4.2601	171	6.1588	171	9.5035	170.7	1.458671	170.8	1.906677	170.9	2.91595	171	3.889195	171	7.780194
170.7999878	1.948400009	170.8999939	2.7624	171	4.2581	171.1	6.1632	171.1	9.5011	170.8	1.457071	170.9	1.904083	171	2.911684	171.1	3.883169	171.1	7.769914
170.8999939	1.944700201	171	2.7674	171.1	4.2606	171.2	6.160801	171.2	9.495201	170.9	1.455474	171	1.901492	171.1	2.907424	171.2	3.877152	171.2	7.759649
171	1.945499967	171.0999908	2.772099	171.2	4.2592	171.3	6.162299	171.3	9.491299	171	1.453877	171.1	1.898905	171.2	2.903171	171.3	3.871148	171.3	7.749403
171.1000061	1.950199962	171.1999969	2.77	171.3	4.2601	171.4	6.1621	171.4	9.4871	171.1	1.452283	171.2	1.896321	171.3	2.898926	171.4	3.865151	171.4	7.739168
171.2000122	1.952300671	171.3000031	2.7722	171.4	4.2598	171.5	6.1552	171.5	9.4811	171.2	1.450691	171.3	1.893742	171.4	2.894686	171.5	3.859165	171.5	7.728949
171.2999878	1.955099325	171.3999939	2.7722	171.5	4.2612	171.6	6.1558	171.6	9.4752	171.3	1.4491	171.4	1.891165	171.5	2.890453	171.6	3.853188	171.6	7.718744
171.3999939	1.956099604	171.5	2.7712	171.6	4.2683	171.7	6.1573	171.7	9.47	171.4	1.447511	171.5	1.888592	171.6	2.886226	171.7	3.847222	171.7	7.708555
171.5	1.957399992	171.5999908	2.775199	171.7	4.2717	171.8	6.156	171.8	9.4662	171.5	1.445924	171.6	1.886023	171.7	2.882006	171.8	3.841266	171.8	7.698383
171.6000061	1.96449999	171.6999969	2.7781	171.8	4.2705	171.9	6.1534	171.9	9.4574	171.6	1.444339	171.7	1.883457	171.8	2.877793	171.9	3.835319	171.9	7.688223
171.7000122	1.963800319	171.8000031	2.7785	171.9	4.269	172	6.1485	172	9.4496	171.7	1.442755	171.8	1.880896	171.9	2.873585	172	3.829382	172	7.678079
171.7999878	1.962799697	171.8999939	2.7786	172	4.2662	172.1	6.1402	172.1	9.438	171.8	1.441174	171.9	1.878338	172	2.869384	172.1	3.823454	172.1	7.667949
171.8999939	1.962800022	172	2.7776	172.1	4.2641	172.2	6.134	172.2	9.4313	171.9	1.439594	172	1.875783	172.1	2.865189	172.2	3.817537	172.2	7.657834
172	1.963500055	172.0999908	2.7717	172.2	4.2598	172.3	6.127	172.3	9.4223	172	1.438015	172.1	1.873231	172.2	2.861001	172.3	3.81163	172.3	7.647737
172.1000061	1.960000031	172.1999969	2.7692	172.3	4.2545	172.4	6.1199	172.4	9.4122	172.1	1.436439	172.2	1.870684	172.3	2.856821	172.4	3.805732	172.4	7.637651
172.2000122	1.959100028	172.3000031	2.7706	172.4	4.2517	172.5	6.1158	172.5	9.4042	172.2	1.434864	172.3	1.86814	172.4	2.852645	172.5	3.799844	172.5	7.62758
172.2999878	1.959799872	172.3999939	2.769	172.5	4.2504	172.6	6.1133	172.6	9.396	172.3	1.433291	172.4	1.8656	172.5	2.848476	172.6	3.793965	172.6	7.617524
172.3999939	1.957600305	172.5	2.7688	172.6	4.2486	172.7	6.116	172.7	9.39	172.4	1.43172	172.5	1.863062	172.6	2.844313	172.7	3.788095	172.7	7.607483
172.5	1.960499961	172.5999908	2.7676	172.7	4.252	172.8	6.1135	172.8	9.3847	172.5	1.43015	172.6	1.860529	172.7	2.840157	172.8	3.782238	172.8	7.59746
172.6000061	1.959200047	172.6999969	2.7669	172.8	4.2539	172.9	6.1098	172.9	9.3787	172.6	1.428582	172.7	1.857999	172.8	2.836008	172.9	3.776388	172.9	7.587448
172.7000122	1.961199972	172.8000031	2.7652	172.9	4.2543	173	6.109	173	9.3795	172.7	1.427016	172.8	1.855473	172.9	2.831864	173	3.770547	173	7.57745
172.7999878	1.961899988	172.8999939	2.7645	173	4.2537	173.1	6.1051	173.1	9.3731	172.8	1.425452	172.9	1.85295	173	2.827727	173.1	3.764717	173.1	7.567468
172.8999939	1.962099813	173	2.7677	173.1	4.2474	173.2	6.1035	173.2	9.3697	172.9	1.42389	173	1.850431	173.1	2.823596	173.2	3.758896	173.2	7.5575
173	1.964199947	173.0999908	2.7667	173.2	4.2419	173.3	6.102599	173.3	9.364099	173	1.422329	173.1	1.847915	173.2	2.819471	173.3	3.753086	173.3	7.547549
173.1000061	1.962699947	173.1999969	2.7659	173.3	4.2426	173.4	6.0974	173.4	9.3564	173.1	1.42077	173.2	1.845403	173.3	2.815354	173.4	3.747284	173.4	7.53761
173.2000122	1.960200474	173.3000031	2.7705	173.4	4.2392	173.5	6.0965	173.5	9.3561	173.2	1.419213	173.3	1.842894	173.4	2.811242	173.5	3.741491	173.5	7.527686
173.2999878	1.961399472	173.3999939	2.7722	173.5	4.2348	173.6	6.0943	173.6	9.3488	173.3	1.417657	173.4	1.840389	173.5	2.807136	173.6	3.735708	173.6	7.517776
173.3999939	1.961000124	173.5	2.774	173.6	4.2347	173.7	6.0953	173.7	9.3449	173.4	1.416103	173.5	1.837887	173.6	2.803036	173.7	3.729935	173.7	7.50788
173.5	1.963800048	173.5999908	2.7747	173.7	4.2352	173.8	6.0931	173.8	9.3362	173.5	1.414551	173.6	1.835389	173.7	2.798943	173.8	3.724173	173.8	7.498002
173.6000061	1.966800045	173.6999969	2.7784	173.8	4.2345	173.9	6.0868	173.9	9.3263	173.6	1.413001	173.7	1.832894	173.8	2.794857	173.9	3.718418	173.9	7.488135
173.7000122	1.972200252	173.8000031	2.7793	173.9	4.2328	174	6.0798	174	9.3141	173.7	1.411452	173.8	1.830403	173.9	2.790776	174	3.712673	174	7.478283
173.7999878	1.971899733	173.8999939	2.7786	174	4.2287	174.1	6.0792	174.1	9.3059	173.8	1.409906	173.9	1.827915	174	2.786701	174.1	3.706938	174.1	7.468445
173.8999939	1.973199846	174	2.7731	174.1	4.2304	174.2	6.0752	174.2	9.3021	173.9	1.40836	174	1.825431	174.1	2.782633	174.2	3.701211	174.2	7.458621
174	1.975800024	174.0999908	2.7725	174.2	4.2302	174.3	6.076099	174.3	9.300599	174	1.406817	174.1	1.82295	174.2	2.77857	174.3	3.695496	174.3	7.448815
174.1000061	1.976700051	174.1999969	2.7747	174.3	4.2334	174.4	6.075	174.4	9.2964	174.1	1.405275	174.2	1.820472	174.3	2.774515	174.4	3.689789	174.4	7.43902
174.2000122	1.976000449	174.3000031	2.779	174.4	4.232	174.5	6.0741	174.5	9.2921	174.2	1.403735	174.3	1.817999	174.4	2.770465	174.5	3.684091	174.5	7.429239
174.2999878	1.9741995	174.3999939	2.7813	174.5	4.2352	174.6	6.0744	174.6	9.2887	174.3	1.402197	174.4	1.815528	174.5	2.766422	174.6	3.678402	174.6	7.419472
174.3999939	1.973099627	174.5	2.7824	174.6	4.2347	174.7	6.0748	174.7	9.2882	174.4	1.40066	174.5	1.813061	174.6	2.762384	174.7	3.672723	174.7	7.40972
174.5	1.973899986	174.5999908	2.783199	174.7	4.2379	174.8	6.071	174.8	9.2858	174.5	1.399125	174.6	1.810597	174.7	2.758353	174.8	3.667055	174.8	7.399985
174.6000061	1.9752	174.6999969	2.7838	174.8	4.2345	174.9	6.0702	174.9	9.2835	174.6	1.397592	174.7	1.808137	174.8	2.754329	174.9	3.661394	174.9	7.390261
174.7000122	1.981600248	174.8000031	2.7819	174.9	4.234	175	6.0709	175	9.2854	174.7	1.396061	174.8	1.805681	174.9	2.75031	175	3.655742	175	7.380551
174.7999878	1.981399728	174.8999939	2.7814	175	4.2347	175.1	6.065	175.1	9.2802	174.8	1.394531	174.9	1.803228	175	2.746297	175.1	3.6501	175.1	7.370855
174.8999939	1.984999614	175	2.7827	175.1	4.2357	175.2	6.065201	175.2	9.278601	174.9	1.393003	175	1.800778	175.1	2.74229	175.2	3.644467	175.2	7.361173
175	1.990799953	175.0999908	2.7793	175.2	4.2371	175.3	6.069099	175.3	9.279499	175	1.391477	175.1	1.798331	175.2	2.738289	175.3	3.638845	175.3	7.351508
175.1000061	1.996400033	175.1999969	2.7826	175.3	4.2358	175.4	6.0664	175.4	9.2763	175.1	1.389952	175.2	1.795888	175.3	2.734295	175.4	3.633231	175.4	7.341855
175.2000122	2.000000832	175.3000031	2.785	175.4	4.2348	175.5	6.0626	175.5	9.2701	175.2	1.388429	175.3	1.793449	175.4	2.730307	175.5	3.627625	175.5	7.332215
175.2999878	2.003599175	175.3999939	2.7838	175.5	4.2332	175.6	6.0604	175.6	9.2705	175.3	1.386908	175.4	1.791012	175.5	2.726324	175.6	3.622029	175.6	7.322589
175.3999939	2.001299696	175.5	2.7816	175.6	4.231	175.7	6.0559	175.7	9.2687	175.4	1.385388	175.5	1.788579	175.6	2.722348	175.7	3.616442	175.7	7.312978
175.5	2.001800017	175.5999908	2.7825	175.7	4.2328	175.8	6.0508	175.8	9.2642	175.5	1.38387	175.6	1.78615	175.7	2.718377	175.8	3.610866	175.8	7.303383
175.6000061	2.002899991	175.6999969	2.7835	175.8	4.2304	175.9	6.050599	175.9	9.261499	175.6	1.382354	175.7	1.783724	175.8	2.714414	175.9	3.605297	175.9	7.293799
175.7000122	2.006300062	175.8000031	2.782	175.9	4.2312	176	6.0518	176	9.2588	175.7	1.38084	175.8	1.781302	175.9	2.710456	176	3.599738	176	7.284229
175.7999878	2.006899993	175.8999939	2.785799	176	4.2305	176.1	6.0537	176.1	9.2554	175.8	1.379327	175.9	1.778882	176	2.706503	176.1	3.594187	176.1	7.274673
175.8999939	2.010399362	176	2.7885	176.1	4.2271	176.2	6.0456	176.2	9.2481	175.9	1.377816	176	1.776466	176.1	2.702557	176.2	3.588646	176.2	7.265131
176	2.008599965	176.0999908	2.7877	176.2	4.2215	176.3	6.0418	176.3	9.2434	176	1.376306	176.1	1.774053	176.2	2.698617	176.3	3.583115	176.3	7.255605
176.1000061	2.008400009	176.1999969	2.7835	176.3	4.2195	176.4	6.0376	176.4	9.2382	176.1	1.374799	176.2	1.771644	176.3	2.694684	176.4	3.577591	176.4	7.246091
176.2000122	2.005600257	176.3000031	2.7843	176.4	4.217	176.5	6.0383	176.5	9.2379	176.2	1.373292	176.3	1.769239	176.4	2.690755	176.5	3.572077	176.5	7.23659
176.2999878	2.007199704	176.3999939	2.781	176.5	4.2206	176.6	6.0345	176.6	9.235	176.3	1.371788	176.4	1.766836	176.5	2.686833	176.6	3.566572	176.6	7.227103
176.3999939	2.008499854	176.5	2.7821	176.6	4.2158	176.7	6.0262	176.7	9.2277	176.4	1.370286	176.5	1.764437	176.6	2.682916	176.7	3.561075	176.7	7.217629
176.5	2.006999944	176.5999908	2.7798	176.7	4.214	176.8	6.018	176.8	9.2214	176.5	1.368784	176.6	1.762041	176.7	2.679006	176.8	3.55559	176.8	7.208173
176.6000061	2.004599994	176.6999969	2.7769	176.8	4.2104	176.9	6.0124	176.9	9.2191	176.6	1.367285	176.7	1.759649	176.8	2.675103	176.9	3.550111	176.9	7.198727
176.7000122	2.004899825	176.8000031	2.7751	176.9	4.2087	177	6.0069	177	9.2136	176.7	1.365787	176.8	1.75726	176.9	2.671204	177	3.544642	177	7.189294
176.7999878	2.007100136	176.8999939	2.7752	177	4.2106	177.1	6.0013	177.1	9.2106	176.8	1.364292	176.9	1.754874	177	2.667311	177.1	3.539181	177.1	7.179876
176.8999939	2.011999597	177	2.7743	177.1	4.2079	177.2	6.001	177.2	9.2059	176.9	1.362797	177	1.752491	177.1	2.663425	177.2	3.53373	177.2	7.17047
177	2.012899998	177.0999908	2.7727	177.2	4.2055	177.3	5.9982	177.3	9.2001	177	1.361304	177.1	1.750112	177.2	2.659544	177.3	3.528289	177.3	7.161082
177.1000061	2.013800048	177.1999969	2.7693	177.3	4.2041	177.4	5.9929	177.4	9.19	177.1	1.359813	177.2	1.747736	177.3	2.65567	177.4	3.522855	177.4	7.151704
177.2000122	2.013900319	177.3000031	2.7663	177.4	4.1985	177.5	5.9872	177.5	9.1843	177.2	1.358324	177.3	1.745364	177.4	2.651801	177.5	3.51743	177.5	7.142339
177.2999878	2.011599784	177.3999939	2.7629	177.5	4.1961	177.6	5.9873	177.6	9.1809	177.3	1.356836	177.4	1.742995	177.5	2.647938	177.6	3.512014	177.6	7.132988
177.3999939	2.007400392	177.5	2.7641	177.6	4.1945	177.7	5.9854	177.7	9.1803	177.4	1.35535	177.5	1.740629	177.6	2.64408	177.7	3.506606	177.7	7.123651
177.5	2.007899985	177.5999908	2.765	177.7	4.1918	177.8	5.9786	177.8	9.1748	177.5	1.353866	177.6	1.738266	177.7	2.640229	177.8	3.50121	177.8	7.11433
177.6000061	2.009799975	177.6999969	2.7678	177.8	4.1846	177.9	5.9789	177.9	9.1702	177.6	1.352383	177.7	1.735907	177.8	2.636384	177.9	3.49582	177.9	7.105019
177.7000122	2.005399529	177.8000031	2.7632	177.9	4.1861	178	5.9763	178	9.1636	177.7	1.350902	177.8	1.733551	177.9	2.632544	178	3.490439	178	7.095722
177.7999878	1.999100461	177.8999939	2.7644	178	4.1825	178.1	5.9743	178.1	9.1607	177.8	1.349423	177.9	1.731198	178	2.62871	178.1	3.485067	178.1	7.086439
177.8999939	2.000599565	178	2.7637	178.1	4.1827	178.2	5.9721	178.2	9.158	177.9	1.347945	178	1.728849	178.1	2.624882	178.2	3.479704	178.2	7.077168
178	1.997300025	178.0999908	2.7639	178.2	4.182	178.3	5.9672	178.3	9.1486	178	1.346468	178.1	1.726502	178.2	2.62106	178.3	3.474351	178.3	7.067914
178.1000061	1.997300049	178.1999969	2.7651	178.3	4.1789	178.4	5.9648	178.4	9.1422	178.1	1.344994	178.2	1.724159	178.3	2.617244	178.4	3.469005	178.4	7.05867
178.2000122	1.995299702	178.3000031	2.765	178.4	4.1772	178.5	5.9624	178.5	9.1393	178.2	1.343521	178.3	1.72182	178.4	2.613434	178.5	3.463667	178.5	7.04944
178.2999878	1.991200254	178.3999939	2.7624	178.5	4.1737	178.6	5.9641	178.6	9.1372	178.3	1.34205	178.4	1.719483	178.5	2.609629	178.6	3.458339	178.6	7.040223
178.3999939	1.987700191	178.5	2.7577	178.6	4.1722	178.7	5.9618	178.7	9.1324	178.4	1.34058	178.5	1.71715	178.6	2.605829	178.7	3.453019	178.7	7.031019
178.5	1.988600022	178.5999908	2.7594	178.7	4.1703	178.8	5.9624	178.8	9.1279	178.5	1.339112	178.6	1.71482	178.7	2.602036	178.8	3.447709	178.8	7.021832
178.6000061	1.989800016	178.6999969	2.7584	178.8	4.166	178.9	5.9604	178.9	9.12	178.6	1.337646	178.7	1.712493	178.8	2.598249	178.9	3.442407	178.9	7.012654
178.7000122	1.989900218	178.8000031	2.7584	178.9	4.1646	179	5.9553	179	9.1142	178.7	1.336181	178.8	1.71017	178.9	2.594467	179	3.437113	179	7.00349
178.7999878	1.991199752	178.8999939	2.7605	179	4.1633	179.1	5.949	179.1	9.1038	178.8	1.334718	178.9	1.70785	179	2.590691	179.1	3.431828	179.1	6.994339
178.8999939	1.992099587	179	2.7596	179.1	4.1622	179.2	5.9464	179.2	9.0983	178.9	1.333257	179	1.705533	179.1	2.586921	179.2	3.426551	179.2	6.985201
179	1.991699995	179.0999908	2.752601	179.2	4.1581	179.3	5.9395	179.3	9.0888	179	1.331797	179.1	1.703219	179.2	2.583156	179.3	3.421284	179.3	6.976079
179.1000061	1.98540003	179.1999969	2.7494	179.3	4.1544	179.4	5.9346	179.4	9.0827	179.1	1.330338	179.2	1.700908	179.3	2.579398	179.4	3.416025	179.4	6.966968
179.2000122	1.983299735	179.3000031	2.7465	179.4	4.1515	179.5	5.9343	179.5	9.0789	179.2	1.328882	179.3	1.698601	179.4	2.575644	179.5	3.410774	179.5	6.957869
179.2999878	1.979000254	179.3999939	2.7423	179.5	4.153	179.6	5.9268	179.6	9.0694	179.3	1.327427	179.4	1.696297	179.5	2.571897	179.6	3.405531	179.6	6.948784

179.3999939	1.976400191	179.5	2.7419	179.6	4.1516	179.7	5.921	179.7	9.0643	179.4	1.325974	179.5	1.693996	179.6	2.568155	179.7	3.400297	179.7	6.939711
179.5	1.977699987	179.5999908	2.7382	179.7	4.1487	179.8	5.912	179.8	9.0593	179.5	1.324522	179.6	1.691698	179.7	2.564418	179.8	3.395073	179.8	6.930655
179.6000061	1.974299971	179.6999969	2.7377	179.8	4.1442	179.9	5.9047	179.9	9.0532	179.6	1.323072	179.7	1.689404	179.8	2.560689	179.9	3.389856	179.9	6.921608
179.7000122	1.972199851	179.8000031	2.7358	179.9	4.1431	180	5.9048	180	9.0507	179.7	1.321623	179.8	1.687113	179.9	2.556964	180	3.384648	180	6.912575
179.7999878	1.967900109	179.8999939	2.7339	180	4.1439	180.1	5.9069	180.1	9.0494	179.8	1.320177	179.9	1.684824	180	2.553244	180.1	3.379448	180.1	6.903554
179.8999939	1.965799901	180	2.7363	180.1	4.142	180.2	5.9006	180.2	9.0425	179.9	1.318731	180	1.682539	180.1	2.54953	180.2	3.374256	180.2	6.894547
180	1.967700005	180.0999908	2.739699	180.2	4.1371	180.3	5.8934	180.3	9.0331	180	1.317287	180.1	1.680257	180.2	2.545822	180.3	3.369075	180.3	6.885555
180.1000061	1.9683	180.1999969	2.7399	180.3	4.131	180.4	5.883901	180.4	9.024701	180.1	1.315845	180.2	1.677979	180.3	2.542121	180.4	3.3639	180.4	6.876573
180.2000122	1.966499513	180.3000031	2.7349	180.4	4.1263	180.5	5.8723	180.5	9.0163	180.2	1.314405	180.3	1.675704	180.4	2.538424	180.5	3.358733	180.5	6.867604
180.2999878	1.965100462	180.3999939	2.733001	180.5	4.1241	180.6	5.8628	180.6	9.0055	180.3	1.312966	180.4	1.673431	180.5	2.534732	180.6	3.353575	180.6	6.858648
180.3999939	1.964200686	180.5	2.7284	180.6	4.1202	180.7	5.854499	180.7	8.992799	180.4	1.311529	180.5	1.671162	180.6	2.531046	180.7	3.348426	180.7	6.849705
180.5	1.960800042	180.5999908	2.726701	180.7	4.1068	180.8	5.844701	180.8	8.976101	180.5	1.310093	180.6	1.668896	180.7	2.527366	180.8	3.343286	180.8	6.840777
180.6000061	1.955100024	180.6999969	2.7247	180.8	4.0953	180.9	5.834601	180.9	8.962801	180.6	1.308659	180.7	1.666633	180.8	2.523693	180.9	3.338153	180.9	6.83186
180.7000122	1.949599072	180.8000031	2.7231	180.9	4.0893	181	5.8209	181	8.9473	180.7	1.307226	180.8	1.664374	180.9	2.520024	181	3.333028	181	6.822955
180.7999878	1.939101007	180.8999939	2.723901	181	4.0757	181.1	5.8173	181.1	8.9375	180.8	1.305796	180.9	1.662117	181	2.51636	181.1	3.327912	181.1	6.814063
180.8999939	1.932300529	181	2.7216	181.1	4.0685	181.2	5.814901	181.2	8.930701	180.9	1.304366	181	1.659863	181.1	2.512702	181.2	3.322804	181.2	6.805183
181	1.921600035	181.0999908	2.7181	181.2	4.0616	181.3	5.820999	181.3	8.928299	181	1.302939	181.1	1.657613	181.2	2.509049	181.3	3.317706	181.3	6.796319
181.1000061	1.919599969	181.1999969	2.7154	181.3	4.0658	181.4	5.8211	181.4	8.9218	181.1	1.301512	181.2	1.655366	181.3	2.505404	181.4	3.312614	181.4	6.787465
181.2000122	1.919401307	181.3000031	2.7193	181.4	4.0683	181.5	5.8175	181.5	8.9123	181.2	1.300088	181.3	1.653122	181.4	2.501762	181.5	3.307531	181.5	6.778623
181.2999878	1.928198715	181.3999939	2.7176	181.5	4.0672	181.6	5.8194	181.6	8.9089	181.3	1.298665	181.4	1.650881	181.5	2.498126	181.6	3.302456	181.6	6.769795
181.3999939	1.928099517	181.5	2.7171	181.6	4.0708	181.7	5.819	181.7	8.9049	181.4	1.297244	181.5	1.648643	181.6	2.494496	181.7	3.297389	181.7	6.760978
181.5	1.927699968	181.5999908	2.717699	181.7	4.07	181.8	5.8148	181.8	8.8974	181.5	1.295824	181.6	1.646408	181.7	2.490871	181.8	3.292332	181.8	6.752177
181.6000061	1.926299964	181.6999969	2.718	181.8	4.0648	181.9	5.817399	181.9	8.891299	181.6	1.294406	181.7	1.644177	181.8	2.487252	181.9	3.287282	181.9	6.743386
181.7000122	1.927900369	181.8000031	2.715	181.9	4.0686	182	5.8212	182	8.8932	181.7	1.292989	181.8	1.641949	181.9	2.483638	182	3.28224	182	6.734608
181.7999878	1.926899678	181.8999939	2.713199	182	4.0717	182.1	5.8241	182.1	8.8982	181.8	1.291574	181.9	1.639723	182	2.48003	182.1	3.277206	182.1	6.725842
181.8999939	1.926399282	182	2.7161	182.1	4.0763	182.2	5.8262	182.2	8.8996	181.9	1.290161	182	1.637501	182.1	2.476427	182.2	3.27218	182.2	6.717088
182	1.928099961	182.0999908	2.724698	182.2	4.0788	182.3	5.825	182.3	8.889	182	1.288749	182.1	1.635281	182.2	2.472829	182.3	3.267163	182.3	6.70835
182.1000061	1.932900045	182.1999969	2.7286	182.3	4.0757	182.4	5.8215	182.4	8.8797	182.1	1.287338	182.2	1.633065	182.3	2.469238	182.4	3.262154	182.4	6.699621
182.2000122	1.939300349	182.3000031	2.7246	182.4	4.0745	182.5	5.8197	182.5	8.875	182.2	1.285929	182.3	1.630852	182.4	2.465651	182.5	3.257152	182.5	6.690905
182.2999878	1.935699728	182.3999939	2.7218	182.5	4.075	182.6	5.8202	182.6	8.8731	182.3	1.284522	182.4	1.628642	182.5	2.46207	182.6	3.252159	182.6	6.682201
182.3999939	1.934099718	182.5	2.7243	182.6	4.0737	182.7	5.821101	182.7	8.872201	182.4	1.283117	182.5	1.626435	182.6	2.458493	182.7	3.247173	182.7	6.67351
182.5	1.932900038	182.5999908	2.725999	182.7	4.0782	182.8	5.823299	182.8	8.871899	182.5	1.281713	182.6	1.624231	182.7	2.454923	182.8	3.242197	182.8	6.664834
182.6000061	1.932999941	182.6999969	2.7238	182.8	4.0798	182.9	5.824699	182.9	8.867499	182.6	1.28031	182.7	1.62203	182.8	2.451359	182.9	3.237228	182.9	6.656167
182.7000122	1.929501056	182.8000031	2.7248	182.9	4.0773	183	5.8311	183	8.8679	182.7	1.278909	182.8	1.619833	182.9	2.447799	183	3.232267	183	6.647513
182.7999878	1.931098949	182.8999939	2.720099	183	4.08	183.1	5.8316	183.1	8.8637	182.8	1.27751	182.9	1.617638	183	2.444244	183.1	3.227313	183.1	6.638871
182.8999939	1.934199286	183	2.7247	183.1	4.0746	183.2	5.836701	183.2	8.863001	182.9	1.276112	183	1.615446	183.1	2.440695	183.2	3.222368	183.2	6.630241
183	1.939400011	183.0999908	2.727799	183.2	4.0797	183.3	5.833599	183.3	8.854799	183	1.274716	183.1	1.613257	183.2	2.437151	183.3	3.217432	183.3	6.621627
183.1000061	1.939000059	183.1999969	2.7268	183.3	4.0775	183.4	5.833899	183.4	8.853699	183.1	1.273321	183.2	1.611072	183.3	2.433614	183.4	3.212503	183.4	6.613021
183.2000122	1.945200559	183.3000031	2.7265	183.4	4.0799	183.5	5.8362	183.5	8.8494	183.2	1.271927	183.3	1.60889	183.4	2.430081	183.5	3.207582	183.5	6.604429
183.2999878	1.945699402	183.3999939	2.728599	183.5	4.082	183.6	5.8393	183.6	8.8473	183.3	1.270536	183.4	1.60671	183.5	2.426553	183.6	3.202668	183.6	6.595848
183.3999939	1.950798997	183.5	2.7312	183.6	4.086	183.7	5.843301	183.7	8.849601	183.4	1.269146	183.5	1.604533	183.6	2.423031	183.7	3.197763	183.7	6.587279
183.5	1.956099973	183.5999908	2.735798	183.7	4.0913	183.8	5.844599	183.8	8.850699	183.5	1.267757	183.6	1.60236	183.7	2.419514	183.8	3.192867	183.8	6.578726
183.6000061	1.965200033	183.6999969	2.7397	183.8	4.092	183.9	5.847999	183.9	8.847699	183.6	1.26637	183.7	1.600189	183.8	2.416003	183.9	3.187977	183.9	6.570181
183.7000122	1.971801232	183.8000031	2.7327	183.9	4.0934	184	5.8455	184	8.8447	183.7	1.264985	183.8	1.598022	183.9	2.412496	184	3.183095	184	6.561649
183.7999878	1.976198692	183.8999939	2.730099	184	4.0911	184.1	5.8446	184.1	8.8447	183.8	1.263601	183.9	1.595857	184	2.408995	184.1	3.178221	184.1	6.553129
183.8999939	1.981298864	184	2.7224	184.1	4.0867	184.2	5.846802	184.2	8.841602	183.9	1.262218	184	1.593696	184.1	2.405499	184.2	3.173355	184.2	6.544622
184	1.979699947	184.0999908	2.722499	184.2	4.0897	184.3	5.852098	184.3	8.842598	184	1.260837	184.1	1.591537	184.2	2.402008	184.3	3.168498	184.3	6.536128
184.1000061	1.979599976	184.1999969	2.7234	184.3	4.0937	184.4	5.853499	184.4	8.841299	184.1	1.259458	184.2	1.589382	184.3	2.398524	184.4	3.163648	184.4	6.527645
184.2000122	1.98760176	184.3000031	2.7254	184.4	4.1005	184.5	5.8615	184.5	8.8445	184.2	1.25808	184.3	1.58723	184.4	2.395044	184.5	3.158805	184.5	6.519173
184.2999878	1.997798202	184.3999939	2.728799	184.5	4.1099	184.6	5.8665	184.6	8.8509	184.3	1.256704	184.4	1.58508	184.5	2.391569	184.6	3.153971	184.6	6.510713
184.3999939	2.003298658	184.5	2.7352	184.6	4.1152	184.7	5.869202	184.7	8.848402	184.4	1.255329	184.5	1.582933	184.6	2.388099	184.7	3.149144	184.7	6.502266
184.5	2.008200004	184.5999908	2.741498	184.7	4.1151	184.8	5.872698	184.8	8.853198	184.5	1.253956	184.6	1.58079	184.7	2.384635	184.8	3.144326	184.8	6.493832
184.6000061	2.016199949	184.6999969	2.7447	184.8	4.126	184.9	5.876998	184.9	8.853898	184.6	1.252584	184.7	1.578649	184.8	2.381176	184.9	3.139515	184.9	6.485409
184.7000122	2.01810162	184.8000031	2.7484	184.9	4.1298	185	5.877	185	8.8507	184.7	1.251214	184.8	1.576512	184.9	2.377722	185	3.134711	185	6.476997
184.7999878	2.022698356	184.8999939	2.748498	185	4.1281	185.1	5.8792	185.1	8.8473	184.8	1.249846	184.9	1.574377	185	2.374273	185.1	3.129915	185.1	6.468597
184.8999939	2.030098413	185	2.7452	185.1	4.1291	185.2	5.8769	185.2	8.8437	184.9	1.248478	185	1.572245	185.1	2.37083	185.2	3.125127	185.2	6.460209
185	2.035400057	185.0999908	2.747699	185.2	4.1282	185.3	5.8694	185.3	8.8337	185	1.247113	185.1	1.570116	185.2	2.367391	185.3	3.120348	185.3	6.451835
185.1000061	2.040300047	185.1999969	2.7463	185.3	4.1248	185.4	5.8647	185.4	8.825	185.1	1.245749	185.2	1.56799	185.3	2.363959	185.4	3.115575	185.4	6.443471
185.2000122	2.037999674	185.3000031	2.7416	185.4	4.1233	185.5	5.8571	185.5	8.8115	185.2	1.244386	185.3	1.565868	185.4	2.360531	185.5	3.11081	185.5	6.435118
185.2999878	2.032200295	185.3999939	2.7425	185.5	4.1192	185.6	5.8483	185.6	8.7967	185.3	1.243025	185.4	1.563748	185.5	2.357108	185.6	3.106052	185.6	6.426778
185.3999939	2.028000199	185.5	2.7406	185.6	4.1146	185.7	5.843599	185.7	8.792199	185.4	1.241666	185.5	1.561631	185.6	2.35369	185.7	3.101302	185.7	6.418449
185.5	2.027199967	185.5999908	2.737001	185.7	4.1133	185.8	5.833101	185.8	8.784501	185.5	1.240307	185.6	1.559517	185.7	2.350277	185.8	3.096562	185.8	6.410134
185.6000061	2.028500048	185.6999969	2.7408	185.8	4.1115	185.9	5.823101	185.9	8.773201	185.6	1.238951	185.7	1.557405	185.8	2.34687	185.9	3.091827	185.9	6.401829
185.7000122	2.023699342	185.8000031	2.7446	185.9	4.1104	186	5.8112	186	8.76	185.7	1.237596	185.8	1.555298	185.9	2.343468	186	3.0871	186	6.393535
185.7999878	2.021900586	185.8999939	2.740801	186	4.1067	186.1	5.8015	186.1	8.7501	185.8	1.236242	185.9	1.553192	186	2.340071	186.1	3.082381	186.1	6.385253
185.8999939	2.019400938	186	2.7313	186.1	4.0993	186.2	5.7987	186.2	8.745	185.9	1.23489	186	1.55109	186.1	2.336678	186.2	3.077669	186.2	6.376983
186	2.011800057	186.0999908	2.722001	186.2	4.0994	186.3	5.7935	186.3	8.7388	186	1.23354	186.1	1.54899	186.2	2.333291	186.3	3.072966	186.3	6.368727
186.1000061	2.001999988	186.1999969	2.7184	186.3	4.1003	186.4	5.789001	186.4	8.726901	186.1	1.232191	186.2	1.546894	186.3	2.32991	186.4	3.068269	186.4	6.36048
186.2000122	1.997499719	186.3000031	2.716	186.4	4.097	186.5	5.7856	186.5	8.721	186.2	1.230843	186.3	1.544801	186.4	2.326533	186.5	3.06358	186.5	6.352245
186.2999878	1.992600324	186.3999939	2.712601	186.5	4.0953	186.6	5.7762	186.6	8.7152	186.3	1.229497	186.4	1.54271	186.5	2.323161	186.6	3.058899	186.6	6.344021
186.3999939	1.988800651	186.5	2.7106	186.6	4.0938	186.7	5.7683	186.7	8.7071	186.4	1.228153	186.5	1.540622	186.6	2.319794	186.7	3.054225	186.7	6.335809
186.5	1.98520002	186.5999908	2.7089	186.7	4.0898	186.8	5.7629	186.8	8.7012	186.5	1.226809	186.6	1.538537	186.7	2.316433	186.8	3.04956	186.8	6.327611
186.6000061	1.984600019	186.6999969	2.7068	186.8	4.0909	186.9	5.7625	186.9	8.6968	186.6	1.225468	186.7	1.536454	186.8	2.313077	186.9	3.044901	186.9	6.319422
186.7000122	1.978899779	186.8000031	2.7079	186.9	4.0901	187	5.761	187	8.6891	186.7	1.224128	186.8	1.534376	186.9	2.309725	187	3.040249	187	6.311245
186.7999878	1.973900176	186.8999939	2.7091	187	4.0832	187.1	5.7608	187.1	8.6866	186.8	1.222789	186.9	1.532299	187	2.306378	187.1	3.035605	187.1	6.303079
186.8999939	1.974200251	187	2.7068	187.1	4.0837	187.2	5.761999	187.2	8.687099	186.9	1.221452	187	1.530226	187.1	2.303037	187.2	3.030968	187.2	6.294925
187	1.972599956	187.0999908	2.7131	187.2	4.0857	187.3	5.756601	187.3	8.676401	187	1.220117	187.1	1.528155	187.2	2.2997	187.3	3.02634	187.3	6.286785
187.1000061	1.974200033	187.1999969	2.7174	187.3	4.0827	187.4	5.7498	187.4	8.6708	187.1	1.218782	187.2	1.526087	187.3	2.29637	187.4	3.021719	187.4	6.278653
187.2000122	1.975499245	187.3000031	2.7143	187.4	4.0807	187.5	5.7496	187.5	8.6623	187.2	1.21745	187.3	1.524023	187.4	2.293043	187.5	3.017104	187.5	6.270533
187.2999878	1.974600713	187.3999939	2.7115	187.5	4.0785	187.6	5.7533	187.6	8.6627	187.3	1.216119	187.4	1.521961	187.5	2.289721	187.6	3.012497	187.6	6.262425
187.3999939	1.97370014	187.5	2.7061	187.6	4.0841	187.7	5.7512	187.7	8.6521	187.4	1.214789	187.5	1.519901	187.6	2.286405	187.7	3.007898	187.7	6.254328
187.5	1.97210003	187.5999908	2.706399	187.7	4.0821	187.8	5.7506	187.8	8.6418	187.5	1.213461	187.6	1.517845	187.7	2.283093	187.8	3.003307	187.8	6.246245
187.6000061	1.978499948	187.6999969	2.705	187.8	4.079	187.9	5.747899	187.9	8.635499	187.6	1.212134	187.7	1.515791	187.8	2.279787	187.9	2.998722	187.9	6.23817
187.7000122	1.979600163	187.8000031	2.7056	187.9	4.0788	188	5.7464	188	8.6367	187.7	1.210808	187.8	1.513741	187.9	2.276486	188	2.994144	188	6.230107
187.7999878	1.982199921	187.8999939	2.710999	188	4.0815	188.1	5.7432	188.1	8.6343	187.8	1.209485	187.9	1.511693	188	2.273189	188.1	2.989574	188.1	6.222056
187.8999939	1.988699462	188	2.7112	188.1	4.076	188.2	5.741499	188.2	8.620099	187.9	1.208162	188	1.509648	188.1	2.269897	188.2	2.985011	188.2	6.214016
188	1.995500011	188.0999908	2.7143	188.2	4.073	188.3	5.7367	188.3	8.6091	188	1.206841	188.1	1.507606	188.2	2.26661	188.3	2.980457	188.3	6.205989
188.1000061	1.995399999	188.1999969	2.7155	188.3	4.0699	188.4	5.727501	188.4	8.593401	188.1	1.205522	188.2	1.505567	188.3	2.263329	188.4	2.975909	188.4	6.197971
188.2000122	1.996899472	188.3000031	2.717	188.4	4.0648	188.5	5.7254	188.5	8.5881	188.2	1.204204	188.3	1.503531	188.4	2.260052	188.5	2.971368	188.5	6.189965
188.2999878	1.995500491	188.3999939	2.710101	188.5	4.0661	188.6	5.7182	188.6	8.5852	188.3	1.202888	188.4	1.501497	188.5	2.25678	188.6	2.966834	188.6	6.18197
188.3999939	1.989001101	188.5	2.7133	188.6	4.0714	188.7	5.7185	188.7	8.5849	188.4	1.201573	188.5	1.499466	188.6	2.253513	188.7	2.962308	188.7	6.173986
188.5	1.986400009	188.5999908	2.715799	188.7	4.0774	188.8	5.7153	188.8	8.5803	188.5	1.200259	188.6	1.497438	188.7	2.250251	188.8	2.95779	188.8	6.166015
188.6000061	1.99040001	188.6999969	2.718	188.8	4.0746	188.9	5.7136	188.9	8.5706	188.6	1.198947	188.7	1.495413	188.8	2.246994	188.9	2.953278	188.9	6.158054
188.7000122	1.993099779	188.8000031	2.7192	188.9	4.0734	189	5.7131	189	8.5593	188.7	1.197636	188.8	1.493391	188.9	2.243742	189	2.948774	189	6.150104
188.7999878	1.989500315	188.8999939	2.716	189	4.0764	189.1	5.7045	189.1	8.5516	188.8	1.196327	188.9	1.491371	189	2.240494	189.1	2.944276	189.1	6.142164
188.8999939	1.985900288	189	2.7185	189.1	4.0665	189.2	5.689499	189.2	8.529099	188.9	1.195019	189	1.489354	189.1	2.237251	189.2	2.939786	189.2	6.134236
189	1.98900004	189.0999908	2.715001	189.2	4.0537	189.3	5.686201	189.3	8.513401	189	1.193713	189.1	1.48734	189.2	2.234013	189.3	2.935304	189.3	6.126322
189.1000061	1.985800051	189.1999969	2.7058	189.3	4.0424	189.4	5.697898	189.4	8.503198	189.1	1.192408	189.2	1.485328	189.3	2.230781	189.4	2.930828	189.4	6.118416
189.2000122	1.975999381	189.3000031	2.6949	189.4	4.0446	189.5	5.7109	189.5	8.5002	189.2	1.191105	189.3	1.48332	189.4	2.227553	189.5	2.926359	189.5	6.110521
189.2999878	1.972100556	189.3999939	2.710798	189.5	4.0492	189.6	5.7268	189.6	8.4856	189.3	1.189803	189.4	1.481315	189.5	2.22433	189.6	2.921897	189.6	6.102637
189.3999939	1.984498116	189.5	2.7234	189.6	4.0641	189.7	5.729099	189.7	8.473799	189.4	1.188502	189.5	1.479311	189.6	2.221111	189.7	2.917443	189.7	6.094764
189.5	1.999000014	189.5999908	2.742298	189.7	4.0692	189.8	5.719001	189.8	8.481401	189.5	1.187203	189.6	1.477311	189.7	2.217897	189.8	2.912997	189.8	6.086905
189.6000061	2.009499988	189.6999969	2.7449	189.8	4.0676	189.9	5.709101	189.9	8.487401	189.6	1.185905	189.7	1.475314	189.8	2.214689	189.9	2.908557	189.9	6.079055
189.7000122	2.008498897	189.8000031	2.7451	189.9	4.0711	190	5.6924	190	8.4852	189.7	1.184609	189.8	1.473319	189.9	2.211485	190	2.904123	190	6.071215
189.7999878	2.00460104	189.8999939	2.749101	190	4.0669					189.8	1.183315	189.9	1.471327	190	2.208286				
189.8999939	1.982900896	190	2.7461							189.9	1.182021	190	1.469338						
190	1.955700017									190	1.180729								

Fig. 2F

Protein expression of HnRNPA1 (pixels)

Ctrl	LPS	Cel	SiRNA
2400.477	3358.138	2006.536	1880.7
1625.818	4306.487	2392.538	2353.7
1733.608	2482.511	1866.329	2171.1
2304.745	2883.574	1498.528	2593.7

Fig. 2G

TNFα/GAPDH

		control			LPS			50nM			100nM			200nM	
0h	0.0018	0.0002	3	0.0018	0.0001	3	0.0015	0.0001	3	0.0017	0.0001	3	0.0017	0.0001	3
2h	0.0017	7E-05	3	0.016	0.0076	3	0.0015	0.0001	3	0.0019	0.0004	3	0.0015	0.0002	3
4h	0.0013	0.0002	3	0.009	0.0005	3	0.0017	0.0001	3	0.001	6E-05	3	0.0007	6E-05	3
6h	0.0018	0.0002	3	0.0026	0.0003	3	0.0024	0.0005	3	0.0014	2E-05	3	0.0009	6E-05	3

1981.462	3576.853	1662.069	1930.9

n=5 wells of BV2 cells

12h	0.0013	0.0001	3	0.0014	0.0001	3	0.001	5E-05	3	0.0015	7E-05	3	0.001	9E-05	3

n=3 well of cells

Protein expression of IKBα (pixels)

Ctrl	LPS	Cel	SiRNA
13153	8914	26200	20226
12985	13000	24392	15564
22731	8920	26950	22426
13862	12059	18368	13384
10036	9455	27746	17103

n=5 wells of BV2 cells

Protein expression of NFkB (percentage)

Ctrl	LPS	Cel	SiRNA
0	10	2	2
1	9	0	3
0	20	1	4
1	11	0	1
0	11	1	1

n=5 wells of BV2 cells

Protein expression of TNFα (pixels)

Ctrl	LPS	Cel	SiRNA
1671	8435	4642	4256
2825	7114	4922	4942
4798	7342	4962	4203
1699	8995	2557	4509
2573	8008	3391	4892

n=5 wells of BV2 cells

Fig. 2H

E1011/GAPDH

Ctrl	LPS	Cel-L	Cel-M	Cel-H
0.0162	0.0156	0.0154	0.022	0.0196
0.0156	0.0154	0.0184	0.0161	0.0155
0.0121	0.0171	0.0156	0.015	0.0161
0.0159	0.0182	0.0154	0.0129	0.0153
0.0149	0.0153	0.014	0.0133	0.0125

n=5 well of cells

E1011/E23

Ctrl	LPS	Cel-L	Cel-M	Cel-H
0.1668	0.1189	0.1553	0.1489	0.1553
0.1467	0.1371	0.1463	0.1372	0.1468
0.1921	0.1356	0.1346	0.165	0.1472
0.155	0.1521	0.1469	0.147	0.1309
0.1645	0.1528	0.1587	0.1424	0.1368

n=5 well of cells

Protein expression of HnRNPA1 (pixels-Immunofluorescence)

LPS	CEL	LPS-CEL-MG132-200nM
81268	59932	62910
72259	50128	56584
93781	37764	103897
72170	34199	94649
103079	37366	119957

n=5 well of cells

Protein expression of HnRNPA1/GAPDH (Westernblot)

LPS	CEL	LPS-CEL-MG132-200nM
1.37	0.77	1.26
1.52	0.56	1.24
0.78	0.43	1.16

n=3 well of cells

Fig. 2I

TNFα/GAPDH mRNA expression in BLA

Chow	Com	Cel-2.0
0.00001	0.00082	0.00012
0.00007	0.00132	0.00008
0.00002	0.00083	0.00004
0.00005	0.00095	0.00001

n=4 mice/group

Fig. 2J

E1011/GAPDH			E1011/E23			Protein expression of HnRNPA1/GAPDH		
Chow	Com	Cel	Chow	Com	Cel	Chow	Com	Cel
0.0336	0.0235	0.0206	0.3411	0.4789	0.3195	0.4056	0.765	0.7052
0.0167	0.0247	0.0311	0.3101	0.5055	0.424	0.4907	0.5434	0.4927
0.0182	0.0279	0.0244	0.4316	0.652	0.4726	0.3906	0.7804	0.5495
0.0221	0.024	0.023	0.473	0.5231	0.4032	0.3148	0.8007	0.3932
	0.0203	0.025		0.3852	0.5844		0.8114	
n=4-5mice/group			n=4-5mice/group			n=4-5mice/group		

Fig. 2K

Expression of IKBα in BLA (pixels)			Expression of NFkB in BLA (pixels)		
Chow	Com	Cel	Chow	Com	Cel
3291684	637440	2850944	2057325	8775122	895419
2087294	619649	1772799	862757	11032040	2021067
2884892	1335102	2987833	1755869	3006894	1309499
4322379	655699	1564256	1690760	4345306	979746
			2699185		
n=4 mice/group			n=4-5 mice/group		

Fig. 3B

Protein expression of HnRNPA1 (pixels)

Chow-non	Com-non	A1-sh-AAV
4734699	5399224	2611322
2301483	4903360	2063476
2395454	4660993	2143357
2889301	5513554	1909150
2454437	4433620	1659812
2024157	4563407	2065669

n=6 mice/group

Area .cell soma stained with TMEM119 (pixels)

Chow-non	Com-non	A1-sh-AAV
1365	2885	1436
1009	3026	1084
881.4	3039	1186
894	4038	1315
928	3606	1129
995	6561	1101

n=6 mice/group

Protein expression of TNFα (pixels)

Chow-non	Com-non	A1-sh-AAV
2806461	7170891	3059845
3340714	7821563	2805671
3190083	5977082	3316294
2512974	5065937	2009972
2378895	8120772	2954055

n=5 mice/group

Fig. 3C

Weight (g)

		Chow-non			Com-non			A1-sh-AAV	
Day	Mean	SEM	N	Mean	SEM	N	Mean	SEM	N
-6	14.73636	0.213306	11	15.462	0.2632	13	15.671	0.2088	14
-5	18.86364	0.311826	11	20.431	0.2817	13	20.7	0.2845	14
-4	20.86364	0.320452	11	23.162	0.3103	13	22.943	0.3354	14
-3	22.03636	0.369783	11	25.108	0.3992	13	24.8	0.4308	14
-2	24.28	0.371968	10	27.242	0.4073	12	27.138	0.5989	13
-1	24.92	0.445825	10	28.958	0.4531	12	28.515	0.5649	13
0	25.53	0.478131781	10	30.258	0.5324	12	30.592	0.7234	13
1	26.85	0.477126818	10	31.808	0.6671	12	29.662	0.6996	13
2	27.55	0.563249501	10	32.875	0.6938	12	29.908	0.6473	13
3	27.07	0.44	10	32.93	0.65	12	29.18	0.54	13
4	27.34	0.5538	10	33.66	0.5294	12	29.44	0.7158	13

White adipose (g)

Chow-non	Com-non	A1-sh-AAV
0.5602	3.7	1.6188
0.4089	2.41	0.8557
0.3919	3.42	0.8014
0.4644	3.01	1.3794
0.4377	2.92	0.3605
0.612	2.8	0.7738
0.839	2.03	0.6963
0.659	3.53	0.6115
0.5541	3.18	1.3718
0.5816	2.83	0.6085
	2.66	0.3586
	3.45	1.3081
		0.3184

Blood sugar (mmol/L)

		Chow-non			Com-non			A1-sh-AAV	
	Mean	SEM	N	Mean	SEM	N	Mean	SEM	N
0mim	10.07	0.4203	10	12.49	0.3034	12	11.55	0.487	13
15min	19.11	1.338	10	27.56	0.7985	12	24.94	0.9188	13
30min	17.93	1.233	10	30.5	1.01	12	22.6	1.585	13
60min	12.92	0.5122	10	27.08	1.406	12	18.17	1.431	13
120min	8.77	0.5394	10	15.11	0.6834	12	10.5	0.4499	13

Immobility time in the tail suspension tests (s)

Chow-non	Com-non	A1-sh-AAV
153	173	144
52	100	52
70	154	83
92	185	5
78	59	86
57	125	106
108	126	144
108	158	88
72	147	13
96	162	6
	125	54
	112	78
		138

Food Intake (Kcal)

Day		Chow-non			Com-non			A1-sh-AAV	
3	9.18	10.54	10.389	12.424	16.071	12.264	12.905	16.351	15.149
7	11.78667	10.61556	10.351	17.193	18.155	16.912	15.069	14.869	15.911
11	12.05111	10.72889	10.842	16.391	15.39	16.672	14.849	13.506	15.37
15	11.97556	11.40889	11.484	15.87	15.5	15.87	14.371	12.157	14.371
19	10.88	11.22	12.202	16.472	17.554	16.111	14.545	12.895	14.892
23	11.33333	10.76667	11.636	15.109	15.51	14.187	13.503	13.329	13.329
27	11.40889	11.22	11.182	14.869	15.51	15.47	13.72	13.633	13.286

n = 3 cages mice/ group

Central duration time in the open field tests (s)

Chow-non	Com-non	A1-sh-AAV
31.99	14	14
31.75	20	20
21.9	17	15
22.71	15	13
22.29	22	16
15.7	12	15
29.71	19	15
25.51	24	11
20.58	12	18
12.29	20	21
	14	20
	9	11
		21

RER

			Chow-non									Com-non								A1-sh-AAV				
8:00-9:00	0.9	0.92	0.92	0.91	0.83	0.93	0.78	1.01	0.81	0.76	0.78	0.79	0.8	0.73	0.8	0.77	0.72	0.78	0.83	0.78	0.82	0.79	0.84	0.84
9:00-10:00	0.82	0.95	0.95	0.99	0.82	0.91	0.82	1.02	0.87	0.8	0.77	0.81	0.79	0.76	0.81	0.8	0.77	0.83	0.83	0.8	0.82	0.78	0.79	0.81
10:00-11:00	0.81	0.95	0.96	0.97	0.8	0.83	0.83	0.96	0.83	0.79	0.77	0.77	0.78	0.74	0.79	0.81	0.76	0.8	0.79	0.79	0.82	0.76	0.83	0.81
11:00-12:00	0.77	0.94	0.92	0.92	0.83	0.87	0.91	0.87	0.81	0.75	0.8	0.76	0.76	0.74	0.78	0.8	0.74	0.81	0.77	0.75	0.82	0.75	0.81	0.79
12:00-13:00	0.79	0.81	0.91	0.91	0.81	0.86	0.94	0.91	0.83	0.77	0.78	0.83	0.81	0.75	0.78	0.81	0.75	0.77	0.77	0.79	0.79	0.78	0.79	0.77
13:00-14:00	0.88	0.84	0.93	0.79	0.82	0.8	0.96	0.81	0.81	0.79	0.81	0.82	0.77	0.75	0.81	0.79	0.77	0.75	0.78	0.8	0.76	0.81	0.83	0.79
14:00-15:00	0.92	0.81	0.88	0.83	0.81	0.83	0.92	0.84	0.8	0.77	0.84	0.82	0.81	0.75	0.74	0.8	0.76	0.75	0.77	0.77	0.8	0.78	0.81	0.75
15:00-16:00	0.87	0.81	0.84	0.8	0.83	0.79	0.89	0.81	0.81	0.8	0.8	0.78	0.8	0.74	0.81	0.79	0.75	0.78	0.79	0.77	0.78	0.77	0.82	0.75
16:00-17:00	0.84	0.9	0.9	0.84	0.83	0.83	0.86	0.84	0.81	0.79	0.81	0.82	0.77	0.73	0.78	0.78	0.75	0.75	0.77	0.76	0.82	0.78	0.76	0.78
17:00-18:00	0.9	0.88	0.88	0.8	0.81	0.79	0.85	0.87	0.79	0.76	0.81	0.85	0.81	0.75	0.78	0.77	0.8	0.75	0.78	0.79	0.79	0.79	0.76	0.78
18:00-19:00	0.83	0.85	0.81	0.81	0.81	0.78	0.8	0.83	0.81	0.8	0.79	0.79	0.82	0.75	0.75	0.8	0.77	0.78	0.79	0.78	0.83	0.83	0.8	0.8
19:00-20:00	0.8	0.95	0.82	0.82	0.84	0.81	0.79	0.83	0.79	0.82	0.83	0.86	0.82	0.75	0.8	0.79	0.82	0.79	0.8	0.81	0.77	0.78	0.76	0.78
20:00-21:00	0.85	0.92	0.85	0.83	0.83	0.79	0.86	0.85	0.76	0.78	0.78	0.79	0.81	0.73	0.78	0.78	0.77	0.75	0.81	0.75	0.81	0.85	0.8	0.78
21:00-22:00	0.81	0.93	0.94	0.95	0.88	0.84	0.89	0.83	0.81	0.78	0.82	0.84	0.85	0.76	0.8	0.82	0.8	0.77	0.82	0.83	0.8	0.86	0.83	0.79
22:00-23:00	0.92	0.95	0.84	0.86	0.82	0.82	0.92	0.91	0.76	0.77	0.83	0.87	0.86	0.74	0.79	0.77	0.78	0.75	0.81	0.81	0.81	0.83	0.81	0.8
23:00-24:00	0.94	0.95	0.82	0.87	0.84	0.83	0.93	0.89	0.77	0.75	0.83	0.79	0.84	0.75	0.78	0.78	0.79	0.79	0.78	0.81	0.87	0.77	0.85	0.8
0:00-1:00	0.97	0.96	0.87	0.91	0.85	0.89	0.93	0.97	0.84	0.77	0.82	0.8	0.82	0.77	0.8	0.81	0.82	0.84	0.83	0.82	0.87	0.79	0.79	0.84
1:00-2:00	0.97	0.86	0.94	0.88	0.82	0.96	1	0.98	0.82	0.78	0.81	0.78	0.83	0.76	0.8	0.8	0.81	0.86	0.83	0.83	0.82	0.8	0.81	0.83
2:00-3:00	0.97	0.87	0.95	0.9	0.78	0.96	0.98	0.98	0.8	0.76	0.84	0.79	0.81	0.74	0.82	0.82	0.78	0.83	0.81	0.8	0.81	0.83	0.75	0.82
3:00-4:00	1	0.93	0.94	0.93	0.84	0.95	0.98	0.98	0.83	0.76	0.81	0.76	0.8	0.74	0.83	0.79	0.8	0.8	0.83	0.77	0.8	0.79	0.78	0.8
4:00-5:00	0.96	0.89	0.95	0.93	0.82	0.95	1	1.01	0.82	0.79	0.8	0.81	0.79	0.74	0.81	0.79	0.78	0.84	0.8	0.81	0.79	0.79	0.77	0.8
5:00-6:00	1	0.86	0.96	0.91	0.84	0.89	0.98	0.96	0.83	0.79	0.81	0.82	0.77	0.74	0.81	0.77	0.78	0.83	0.78	0.79	0.76	0.79	0.81	0.75
6:00-7:00	0.99	0.91	0.97	0.94	0.83	0.88	0.91	0.95	0.86	0.79	0.81	0.82	0.83	0.75	0.79	0.81	0.78	0.77	0.79	0.84	0.75	0.79	0.84	0.79
7:00-8:00	0.97	0.94	0.95	0.92	0.82	0.91	0.77	0.95	0.83	0.79	0.82	0.81	0.8	0.74	0.76	0.79	0.76	0.79	0.79	0.81	0.78	0.77	0.82	0.77

Fig. 3D

Protein expression of TPH2 (pixels)

Chow-non	Com-non	A1-sh-AAV
6489191	3327561	6203896
5016890	2660306	5937624
8459539	3003681	5341887
5276599	2167280	4365325
4441387	2986248	5539061

n=5 mice/group

Protein expression of NPY (pixels)

Chow-non	Com-non	A1-sh-AAV
4097338	6875880	4906448
4524266	7844495	5493764
3711087	7025621	5055862
3866012	5988000	5197456
4255588	7462556	1536844
		3238706

n=5-6 mice/group

Fig. 3F

Protein expression of HnRNPA1 (pixels)

Chow	COM	Cel	Cel-A1-AAV	COM-A1-AAV
1472957	6043419	3120000	6148438	15588925
2589553	5543019	2230000	8071688	16514105
1762188	6843333	2025586	7984206	13729038
3098374	6175566	3464059	7757866	9729038
2803256	6559039	2433370	6600218	8039478
			10559817	

n=5-6 mice/group

Protein expression of TNFα (pixels)

Chow	COM	Cel	Cel-A1-AAV	COM-A1-AAV
1860450	3171019	3690304	4778382	5495565
1255256	3929843	1141042	4673178	9630122
2111169	2625450	1492723	4625372	8114273
1646738	6451687	467532	6872835	6456270
615896	4887922	2070235	5049652	6910942

n=5 mice/group

Fig. 3G

Weight (g)

	Time	Chow-non Mean	Chow-non SEM	Chow-non N	Com-non Mean	Com-non SEM	Com-non N	Cel-non Mean	Cel-non SEM	Cel-non N	Cel-A1-AAV Mean	Cel-A1-AAV SEM	Cel-A1-AAV N	COM-A1-AAV Mean	COM-A1-AAV SEM	COM-A1-AAV N
WeeK	0	23.98	0.28	21	29.8	0.72	9	30.13	0.83	10	30.59	0.84	9	31.1	0.69	5
WeeK	1	25.52	0.27	21	31.88	0.66	9	31.8	1.04	10	29.94	1.3	9	31.36	0.28	5
WeeK	2	26.35	0.29	21	33.74	0.75	9	33.78	1.21	10	32.57	1.03	9	32.66	1.97	5
WeeK	3	26.81	0.32	21	35	0.8	9	35.71	1.33	10	34.91	1.4	9	35.04	1.93	5
WeeK	4	27.05	0.34	21	37.36	0.88	9	37.9	1.48	10	37.74	1.59	9	38.22	2.07	5
	1	27.07	0.32	21	37	0.9	9	37.7	1.49	10	37.89	1.78	9	37.92	2	5
	2	27.42	0.36	21	37.46	0.87	9	37.71	1.43	10	37.76	1.64	9	38.7	2.02	5
	3	27.41	0.35	21	37.68	0.95	9	37.75	1.47	10	37.62	1.49	9	38.96	1.97	5
Week(5)	4	27.22	0.34	21	37.76	0.96	9	37.72	1.44	10	37.27	1.43	9	39.5	2.01	5
	5	27.22	0.34	21	37.78	1	9	37.32	1.44	10	37.47	1.42	9	39.92	1.96	5
	6	27.58	0.35	21	37.8	0.98	9	37.04	1.36	10	37.98	1.5	9	40.26	1.9	5
	7	27.5	0.34	21	37.82	0.94	9	37.09	1.4	10	38.46	1.66	9	40.6	1.88	5
	1	27.49	0.35	21	38	0.93	9	36.89	1.34	10	38.76	1.75	9	41.08	1.76	5
	2	27.46	0.34	21	38.13	0.95	9	36.64	1.35	10	39.12	1.79	9	41.74	1.87	5
	3	27.58	0.35	21	38.19	0.93	9	36.75	1.39	10	39.81	1.86	9	41.82	1.88	5
Week(6)	4	27.59	0.36	21	38.37	0.93	9	36.55	1.33	10	39.9	1.86	9	42.28	1.81	5
	5	27.59	0.36	21	38.57	0.96	9	36.44	1.24	10	40.23	1.85	9	42.6	1.73	5
	6	27.49	0.34	21	38.69	0.95	9	36.43	1.26	10	40.39	1.8	9	43	1.69	5
	7	27.77	0.35	21	38.87	0.97	9	35.49	1.11	10	40.77	1.83	9	43.26	1.66	5
Week(7)	1	27.75	0.37	21	39.1	0.95	9	35.07	1.111	10	41.04	1.81	9	43.28	1.75	5
	2	27.9	0.37	21	39.07	0.94	9	33.89	1.078	10	41.48	1.91	9	43.58	1.73	5

Immobility time in the tail suspension tests (s)

Chow-non	Com-non	Cel-non	Cel-A1-AAV	COM-A1-AAV
54	172	102	126	153
44	105	128	104	92
117	181	35	120	142
108	137	33	118	132
92	169	50	176	128
107	144	120	158	
76	158	118	144	
82	129	110	144	
99	101	111	131	
86	98	128		

Blood sugar (mmol/L)

Time (min)	Chow-non Mean	Chow-non SEM	Chow-non N	Com-non Mean	Com-non SEM	Com-non N	Cel-non Mean	Cel-non SEM	Cel-non N	Cel-A1-AAV Mean	Cel-A1-AAV SEM	Cel-A1-AAV N	COM-A1-AAV Mean	COM-A1-AAV SEM	COM-A1-AAV N
0min	9.37	0.39	10	13.19	0.31	9	10.76	0.28	10	11.92	0.5	9	14.1	1.27	5
15min	20.83	0.46	10	31.18	0.57	9	26.62	0.64	10	30.17	0.85	9	32.2	0.6	5
30min	19.35	0.72	10	32.31	0.43	9	26.7	0.83	10	31.98	0.41	9	33.3	0	5
60min	13.59	0.4	10	28	0.94	9	21.19	1.36	10	28.53	1.26	9	31.58	0.96	5
120min	9.01	0.39	10	17.93	0.87	9	12.86	0.66	10	18.53	1.68	9	24.5	2.71	5

Food Intake (Kcal)

Day	Chow-non Ctrl			Chow-non COM			Chow-non CEL			Chow-non hnRNPA1			Chow-non hnRNPA1+CEL		
3	11.492	10.506	9.418	13.372	13.068	13.068	14.119	12.139	12.765	16.22	16.429	14.935	14.119	17.506	15.526
6	13.668	11.152	10.948	15.239	15.891	15.239	16.62	15.422	14.796	16.151	16.325	15.699	19.121	15.161	15.317
9	12.308	10.778	11.39	15.934	15.109	14.849	15.891	14.536	14.484	16.568	15.943	14.588	15.838	16.828	15.995
12	11.9	11.56	11.934	15.934	15.066	12.244	15.213	15.265	11.202	17.054	16.359	11.427	17.61	14.536	11.775
15	11.22	12.682	11.016	13.459	15.283	14.371	13.129	14.588	13.233	12.504	14.38	12.99	11.097	15.213	16.255
18	11.39	12.036	11.798	14.11	16.412	16.021	13.39	14.484	15.109	13.963	15.248	16.151	12.869	15.943	17.766
21	11.39	10.404	10.336	15.63	13.98	14.796	13.155	12.66	12.869	15.769	13.65	13.129	18.079	13.233	12.608
24	11.186	10.948	11.424	15.239	13.754	14.024	13.546	12.817	12.4	14.97	13.998	13.372	14.223	13.546	12.244
27	12.24	10.506	12.138	15.022	13.702	15.37	12.921	12.712	13.242	14.796	13.025	14.623	14.953	13.129	14.901
30	11.798	11.152	11.798	14.328	13.503	13.702	11.94	12.244	11.879	13.789	12.851	12.122	13.859	13.025	12.296
33	12.308	10.982	11.254	14.892	14.38	13.416	12.417	11.149	12.66	16.116	11.844	14.032	13.077	12.608	13.546
36	11.696	10.948	11.356	14.588	13.155	14.718	12.494	12.191	12.39	14.692	13.025	15.422	14.067	12.139	15.057

n=3cages mice/group

RER

Hrs	Chow-non Mean	Chow-non SEM	Chow-non N	Com-non Mean	Com-non SEM	Com-non N	Cel-non Mean	Cel-non SEM	Cel-non N	Cel-A1-AAV Mean	Cel-A1-AAV SEM	Cel-A1-AAV N	COM-A1-AAV Mean	COM-A1-AAV SEM	COM-A1-AAV N
8:00-9:00	0.8842	0.0208	8	0.7996	0.0107	8	0.8004	0.0057	8	0.7805	0.0086	8	0.7965	0.005	8
9:00-10:00	0.8529	0.0232	8	0.7971	0.0045	8	0.7926	0.0052	8	0.7819	0.0104	8	0.7887	0.0071	8
10:00-11:00	0.9246	0.0154	8	0.8033	0.0075	8	0.7904	0.0059	8	0.7701	0.0055	8	0.7863	0.0043	8
11:00-12:00	0.9101	0.0156	8	0.7818	0.0062	8	0.7766	0.0077	8	0.7628	0.0038	8	0.7694	0.0076	8
12:00-13:00	0.8641	0.0211	8	0.7736	0.0059	8	0.7598	0.0082	8	0.7614	0.0085	8	0.7577	0.0057	8
13:00-14:00	0.8715	0.016	8	0.7932	0.0084	8	0.7796	0.0118	8	0.764	0.0067	8	0.7735	0.0061	8
14:00-15:00	0.872	0.0127	8	0.7891	0.0062	8	0.7833	0.0095	8	0.772	0.0082	8	0.7712	0.006	8
15:00-16:00	0.8566	0.0159	8	0.7856	0.0087	8	0.7776	0.0078	8	0.7665	0.0046	8	0.7738	0.0108	8
16:00-17:00	0.7796	0.024	8	0.7971	0.0098	8	0.7661	0.0057	8	0.7795	0.0064	8	0.7662	0.0049	8
17:00-18:00	0.8167	0.0103	8	0.7872	0.0082	8	0.7801	0.0071	8	0.7756	0.0061	8	0.771	0.0051	8
18:00-19:00	0.8333	0.0134	8	0.7862	0.0076	8	0.7737	0.0089	8	0.7577	0.005	8	0.7583	0.0054	8
19:00-20:00	0.8223	0.0199	8	0.7909	0.0094	8	0.7717	0.0089	8	0.768	0.0128	8	0.7697	0.0056	8
20:00-21:00	0.8527	0.0237	8	0.7946	0.0138	8	0.7789	0.0091	8	0.7814	0.0127	8	0.7683	0.0054	8
21:00-22:00	0.8819	0.0193	8	0.7914	0.013	8	0.7664	0.0073	8	0.7831	0.0137	8	0.7755	0.0084	8
22:00-23:00	0.9106	0.0236	8	0.8115	0.0104	8	0.7939	0.0092	8	0.7841	0.0104	8	0.7923	0.0101	8
23:00-24:00	0.8988	0.0215	8	0.7916	0.0086	8	0.8024	0.007	8	0.7787	0.0122	8	0.801	0.0128	8
0:00-1:00	0.9469	0.0192	8	0.8015	0.0108	8	0.8081	0.0103	8	0.7732	0.0063	8	0.7906	0.0098	8
1:00-2:00	0.9505	0.0187	8	0.7895	0.0111	8	0.821	0.0089	8	0.7898	0.0126	8	0.8001	0.0127	8
2:00-3:00	0.938	0.017	8	0.8044	0.0068	8	0.8027	0.0059	8	0.784	0.0108	8	0.7749	0.0064	8
3:00-4:00	0.9191	0.0206	8	0.8099	0.0069	8	0.7921	0.0079	8	0.7841	0.0103	8	0.7705	0.0068	8
4:00-5:00	0.9299	0.0149	8	0.7986	0.0079	8	0.7859	0.0081	8	0.7905	0.0087	8	0.7805	0.0056	8
5:00-6:00	0.9013	0.0221	8	0.7831	0.0061	8	0.7869	0.0054	8	0.7904	0.007	8	0.7782	0.003	8
6:00-7:00	0.8915	0.0164	8	0.7831	0.0066	8	0.771	0.0043	8	0.7851	0.0058	8	0.7792	0.012	8
7:00-8:00	0.91	0.0152	8	0.8071	0.0059	8	0.7972	0.0081	8	0.7892	0.011	8	0.7858	0.0071	8

Fig. 3H

NPY (pixels)

Chow-non	Com-non	Cel-non	Cel-A1-AAV	COM-A1-AAV
1224656	4120654	2779682	6714669	6170463
2790600	4312447	2875654	4076394	4082418
1663244	3366740	2322386	5190813	8562128
1654522	5097615	2494354	5097582	6245032
1626959	5222311	2013950	6657587	7402616

n=5 mice/group

TPH2 (pixels)

Chow-non	Com-non	Cel-non	Cel-A1-AAV	COM-A1-AAV
6667226	322927	2198634	783276	1169799
5117232	1081217	2786005	851437	701380
4981722	574771	1475050	595007	469281
4823663	340675	3113780	363985	433955
3275803	906010	2347901	674098	788219

n=5 mice/group
