# Supplementary material for: Celastrol alleviates comorbid obesity and depression by directly binding amygdala HnRNPA1 in a mouse model
Source: Clin Transl Med. 2021 Jun 6;11(6):e394. doi: 10.1002/ctm2.394 (PMC8181197; doi:10.1002/ctm2.394)
Supplement: Supplementary file 6 — Supporting Information [file CTM2-11-e394-s005.pdf]

Coverage [%]	Accession	Description	# Peptides	p-value	ratio
60	P63038	60 kDa heat shock protein, mitochondrial OS=Mus musculus OX=10090 GN=Hspd1 PE=1 SV=1	56	9.09E-08	12.978
14	Q9QXS1	Plectin OS=Mus musculus OX=10090 GN=Plec PE=1 SV=3	27	2.54E-06	5.5103
20	Q68FD5	Clathrin heavy chain 1 OS=Mus musculus OX=10090 GN=Cltc PE=1 SV=3	26	9.57E-06	3.9455
43	P52480	Pyruvate kinase PKM OS=Mus musculus OX=10090 GN=Pkm PE=1 SV=4	25	1.24E-05	5.6871
42	P38647	Stress-70 protein, mitochondrial OS=Mus musculus OX=10090 GN=Hspa9 PE=1 SV=3	25	4.33E-07	7.0573
46	P63017	Heat shock cognate 71 kDa protein OS=Mus musculus OX=10090 GN=Hspa8 PE=1 SV=1	24	1.32E-08	5.4161
27	Q05920	Pyruvate carboxylase, mitochondrial OS=Mus musculus OX=10090 GN=Pc PE=1 SV=1	24	0.000317	1.433
15	Q8VDD5	Myosin-9 OS=Mus musculus OX=10090 GN=Myh9 PE=1 SV=4	23	4.52E-06	5.1306
32	P20029	Endoplasmic reticulum chaperone BiP OS=Mus musculus OX=10090 GN=Hspa5 PE=1 SV=3	22	6.95E-06	8.519
36	Q91ZA3	Propionyl-CoA carboxylase alpha chain, mitochondrial OS=Mus musculus OX=10090 GN=Pcca PE=1 SV=2	21	8.33E-05	1.5155
24	O70133	ATP-dependent RNA helicase A OS=Mus musculus OX=10090 GN=Dhx9 PE=1 SV=2	21	1.59E-06	5.7456
35	P48678	Prelamin-A/C OS=Mus musculus OX=10090 GN=Lmna PE=1 SV=2	21	9.62E-07	7.7619
46	P08249	Malate dehydrogenase, mitochondrial OS=Mus musculus OX=10090 GN=Mdh2 PE=1 SV=3	20	3.35E-06	5.7307
24	Q3V3R1	Monofunctional C1-tetrahydrofolate synthase, mitochondrial OS=Mus musculus OX=10090 GN=Mthfd1l PE=1 SV=2	20	3.46E-07	6.8154
12	Q6P4T2	U5 small nuclear ribonucleoprotein 200 kDa helicase OS=Mus musculus OX=10090 GN=Snrnp200 PE=1 SV=1	19	7.28E-06	3.646
31	Q03265	ATP synthase subunit alpha, mitochondrial OS=Mus musculus OX=10090 GN=Atp5f1a PE=1 SV=1	18	9.55E-08	6.0996
15	Q7TPV4	Myb-binding protein 1A OS=Mus musculus OX=10090 GN=Mybbp1a PE=1 SV=2	18	3.11E-07	6.8167
26	P08113	Endoplasmin OS=Mus musculus OX=10090 GN=Hsp90b1 PE=1 SV=2	18	1.08E-06	7.3780
14	O55143	Sarcoplasmic/endoplasmic reticulum calcium ATPase 2 OS=Mus musculus OX=10090 GN=Atp2a2 PE=1 SV=2	18	1.99E-07	6.3320
42	P60710	Actin, cytoplasmic 1 OS=Mus musculus OX=10090 GN=Actb PE=1 SV=1	18	9.57E-06	5.8414
15	Q6PB66	Leucine-rich PPR motif-containing protein, mitochondrial OS=Mus musculus OX=10090 GN=Lrpprc PE=1 SV=2	17	7.56E-08	4.600
43	P16858	Glyceraldehyde-3-phosphate dehydrogenase OS=Mus musculus OX=10090 GN=Gapdh PE=1 SV=2	16	6.39E-08	6.4984
26	Q99KI0	Aconitate hydratase, mitochondrial OS=Mus musculus OX=10090 GN=Aco2 PE=1 SV=1	16	5.67E-06	3.1261
23	P11499	Heat shock protein HSP 90-beta OS=Mus musculus OX=10090 GN=Hsp90ab1 PE=1 SV=3	16	2.91E-08	8.0729
41	P20152	Vimentin OS=Mus musculus OX=10090 GN=Vim PE=1 SV=3	16	5.12E-06	6.1653
30	P09405	Nucleolin OS=Mus musculus OX=10090 GN=Ncl PE=1 SV=2	15	7.45E-07	12.842
24	Q8VEK3	Heterogeneous nuclear ribonucleoprotein U OS=Mus musculus OX=10090 GN=Hnrnpu PE=1 SV=1	15	3.07E-07	6.7020
21	P07901	Heat shock protein HSP 90-alpha OS=Mus musculus OX=10090 GN=Hsp90aa1 PE=1 SV=4	14	1.19E-07	7.0994
41	P68369	Tubulin alpha-1A chain OS=Mus musculus OX=10090 GN=Tuba1a PE=1 SV=1	14	1.01E-05	5.059
27	Q8BH04	Phosphoenolpyruvate carboxykinase [GTP], mitochondrial OS=Mus musculus OX=10090 GN=Pck2 PE=1 SV=1	14	2.14E-10	5.8749
8	Q9ERU9	E3 SUMO-protein ligase RanBP2 OS=Mus musculus OX=10090 GN=Ranbp2 PE=1 SV=2	14	1.56E-05	4.8931
19	Q8K411	Presequence protease, mitochondrial OS=Mus musculus OX=10090 GN=Pitrm1 PE=1 SV=1	14	1.78E-05	4.0310
20	Q99MR8	Methylcrotonoyl-CoA carboxylase subunit alpha, mitochondrial OS=Mus musculus OX=10090 GN=Mccc1 PE=1 SV=2	14	1.24E-05	1.6632
25	P26443	Glutamate dehydrogenase 1, mitochondrial OS=Mus musculus OX=10090 GN=Glud1 PE=1 SV=1	13	1.92E-06	4.3911
35	P17182	Alpha-enolase OS=Mus musculus OX=10090 GN=Eno1 PE=1 SV=3	13	7.77E-05	6.1741
18	Q8BMS1	Trifunctional enzyme subunit alpha, mitochondrial OS=Mus musculus OX=10090 GN=Hadha PE=1 SV=1	13	6.5E-07	6.4082
15	Q8CGK3	Lon protease homolog, mitochondrial OS=Mus musculus OX=10090 GN=Lonp1 PE=1 SV=2	12	5.45E-06	5.4439
29	Q9CZN7	Serine hydroxymethyltransferase, mitochondrial OS=Mus musculus OX=10090 GN=Shmt2 PE=1 SV=1	12	3.82E-06	3.0169
21	Q8R081	Heterogeneous nuclear ribonucleoprotein L OS=Mus musculus OX=10090 GN=Hnrnpl PE=1 SV=2	12	3.52E-06	4.8879
28	O88569	Heterogeneous nuclear ribonucleoproteins A2/B1 OS=Mus musculus OX=10090 GN=Hnrnpa2b1 PE=1 SV=2	12	2.05E-07	8.2143
36	P51881	ADP/ATP translocase 2 OS=Mus musculus OX=10090 GN=Slc25a5 PE=1 SV=3	12	4.17E-07	7.6114
9	Q99PV0	Pre-mRNA-processing-splicing factor 8 OS=Mus musculus OX=10090 GN=Prpf8 PE=1 SV=2	12	4.45E-06	3.3670
8	E9Q7G0	Nuclear mitotic apparatus protein 1 OS=Mus musculus OX=10090 GN=Numa1 PE=1 SV=1	12	2.3E-07	4.9753
37	Q61937	Nucleophosmin OS=Mus musculus OX=10090 GN=Npm1 PE=1 SV=1	12	8.61E-07	10.113
28	P56480	ATP synthase subunit beta, mitochondrial OS=Mus musculus OX=10090 GN=Atp5f1b PE=1 SV=2	12	4.89E-06	4.2619
22	Q9CQN1	Heat shock protein 75 kDa, mitochondrial OS=Mus musculus OX=10090 GN=Trap1 PE=1 SV=1	12	3.54E-06	4.938
22	P47738	Aldehyde dehydrogenase, mitochondrial OS=Mus musculus OX=10090 GN=Aldh2 PE=1 SV=1	12	5.29E-06	4.3876
15	Q8BG05	Heterogeneous nuclear ribonucleoprotein A3 OS=Mus musculus OX=10090 GN=Hnrnpa3 PE=1 SV=1	12	5.54E-06	6.128
19	Q8C4J7	Transducin beta-like protein 3 OS=Mus musculus OX=10090 GN=Tbl3 PE=2 SV=1	12	8.31E-07	4.6951
23	P54987	Cis-aconitate decarboxylase OS=Mus musculus OX=10090 GN=Acod1 PE=1 SV=2	12	5.12E-06	4.9556
17	Q8VEM8	Phosphate carrier protein, mitochondrial OS=Mus musculus OX=10090 GN=Slc25a3 PE=1 SV=1	11	2.58E-05	6.4464
44	P67778	Prohibitin OS=Mus musculus OX=10090 GN=Phb PE=1 SV=1	11	7.37E-07	5.8536
7	P19096	Fatty acid synthase OS=Mus musculus OX=10090 GN=Fasn PE=1 SV=2	11	1.38E-05	3.4707
27	Q8BFR5	Elongation factor Tu, mitochondrial OS=Mus musculus OX=10090 GN=Tufm PE=1 SV=1	11	2.62E-08	5.7323
18	Q62351	Transferrin receptor protein 1 OS=Mus musculus OX=10090 GN=Tfrc PE=1 SV=1	11	1.05E-06	2.7167
22	Q8CI11	Guanine nucleotide-binding protein-like 3 OS=Mus musculus OX=10090 GN=Gnl3 PE=1 SV=2	11	7.74E-07	5.1779
21	Q921H8	3-ketoacyl-CoA thiolase A, peroxisomal OS=Mus musculus OX=10090 GN=Acaa1a PE=1 SV=1	11	9.03E-08	6.8800
29	Q9Z204	Heterogeneous nuclear ribonucleoproteins C1/C2 OS=Mus musculus OX=10090 GN=Hnrnpc PE=1 SV=1	10	3.64E-09	5.4567
25	P54071	Isocitrate dehydrogenase [NADP], mitochondrial OS=Mus musculus OX=10090 GN=Idh2 PE=1 SV=3	10	2.69E-06	5.7722
13	Q78PY7	Staphylococcal nuclease domain-containing protein 1 OS=Mus musculus OX=10090 GN=Snd1 PE=1 SV=1	10	1.94E-08	6.158
15	O08810	116 kDa U5 small nuclear ribonucleoprotein component OS=Mus musculus OX=10090 GN=Eftud2 PE=1 SV=1	10	6.14E-06	4.4675
23	P35564	Calnexin OS=Mus musculus OX=10090 GN=Canx PE=1 SV=1	10	1.1E-07	7.259
14	Q60597	2-oxoglutarate dehydrogenase, mitochondrial OS=Mus musculus OX=10090 GN=Ogdh PE=1 SV=3	10	1.01E-07	3.7843
13	Q91ZW3	lated matrix-associated actin-dependent regulator of chromatin subfamily A member 5 OS=Mus musculus OX=10090 GN=Smarca5	10	1.99E-05	6.0780
17	Q64521	Glycerol-3-phosphate dehydrogenase, mitochondrial OS=Mus musculus OX=10090 GN=Gpd2 PE=1 SV=2	10	1.56E-07	3.8268
19	Q9Z110	Delta-1-pyrroline-5-carboxylate synthase OS=Mus musculus OX=10090 GN=Aldh18a1 PE=1 SV=2	10	2.37E-06	4.8213
12	O35286	Pre-mRNA-splicing factor ATP-dependent RNA helicase DHX15 OS=Mus musculus OX=10090 GN=Dhx15 PE=1 SV=2	10	1.82E-05	4.0631
2	Q9QXZ0	Microtubule-actin cross-linking factor 1 OS=Mus musculus OX=10090 GN=Macf1 PE=1 SV=2	10	1.23E-05	4.928
13	P02769	SWISS-PROT:P02769 (Bos taurus) Bovine serum albumin precursor	10	5.25E-06	8.0004
11	Q7TPR4	Alpha-actinin-1 OS=Mus musculus OX=10090 GN=Actn1 PE=1 SV=1	10	1.63E-07	4.1892
20	O08749	Dihydrolipoyl dehydrogenase, mitochondrial OS=Mus musculus OX=10090 GN=Dld PE=1 SV=2	10	3.3E-07	6.1991
28	Q8BVY0	Ribosomal L1 domain-containing protein 1 OS=Mus musculus OX=10090 GN=Rsl1d1 PE=1 SV=1	9	3.87E-07	8.2732
29	Q99020	Heterogeneous nuclear ribonucleoprotein A/B OS=Mus musculus OX=10090 GN=Hnrnpab PE=1 SV=1	9	1.07E-05	5.6990
25	P99024	Tubulin beta-5 chain OS=Mus musculus OX=10090 GN=Tubb5 PE=1 SV=1	9	8.25E-05	3.1115
11	O08528	Hexokinase-2 OS=Mus musculus OX=10090 GN=Hk2 PE=1 SV=1	9	3.86E-05	5.501
25	P68372	Tubulin beta-4B chain OS=Mus musculus OX=10090 GN=Tubb4b PE=1 SV=1	9	8.95E-06	4.6582
8	Q9JKF1	Ras GTPase-activating-like protein IQGAP1 OS=Mus musculus OX=10090 GN=Iqgap1 PE=1 SV=2	9	1.07E-06	3.0276
20	P05064	Fructose-bisphosphate aldolase A OS=Mus musculus OX=10090 GN=Aldoa PE=1 SV=2	9	2.75E-09	6.1864
14	Q04750	DNA topoisomerase 1 OS=Mus musculus OX=10090 GN=Top1 PE=1 SV=2	9	1.17E-05	3.7999
17	D3Z7P3	Glutaminase kidney isoform, mitochondrial OS=Mus musculus OX=10090 GN=Gls PE=1 SV=1	9	7.31E-07	6.3549
22	P26041	Moesin OS=Mus musculus OX=10090 GN=Msn PE=1 SV=3	9	1.38E-05	6.6565
18	Q8K2B3	Succinate dehydrogenase [ubiquinone] flavoprotein subunit, mitochondrial OS=Mus musculus OX=10090 GN=Sdha PE=1 SV=1	9	3.19E-06	4.738
14	Q9QXX4	Calcium-binding mitochondrial carrier protein Aralar2 OS=Mus musculus OX=10090 GN=Slc25a13 PE=1 SV=1	9	1.47E-07	5.469
11	Q3U821	WD repeat-containing protein 75 OS=Mus musculus OX=10090 GN=Wdr75 PE=1 SV=1	9	3.49E-05	5.7900
16	Q921N6	Probable ATP-dependent RNA helicase DDX27 OS=Mus musculus OX=10090 GN=Ddx27 PE=1 SV=3	9	1.22E-05	4.4854
18	P37040	NADPH--cytochrome P450 reductase OS=Mus musculus OX=10090 GN=Por PE=1 SV=2	9	2.17E-06	4.9086
11	P17710	Hexokinase-1 OS=Mus musculus OX=10090 GN=Hk1 PE=1 SV=3	9	6.19E-07	4.6412
22	P06151	L-lactate dehydrogenase A chain OS=Mus musculus OX=10090 GN=Ldha PE=1 SV=3	9	1.99E-07	4.258
11	Q8BH59	Calcium-binding mitochondrial carrier protein Aralar1 OS=Mus musculus OX=10090 GN=Slc25a12 PE=1 SV=1	9	1.65E-06	3.4216
26	P61979	Heterogeneous nuclear ribonucleoprotein K OS=Mus musculus OX=10090 GN=Hnrnpk PE=1 SV=1	9	1.87E-08	8.2985
30	P17751	Triosephosphate isomerase OS=Mus musculus OX=10090 GN=Tpi1 PE=1 SV=4	9	2.02E-06	5.8728
17	Q8BMF4	/sine-residue acetyltransferase component of pyruvate dehydrogenase complex, mitochondrial OS=Mus musculus OX=10090 GN=D	9	2.45E-07	5.8231
16	Q8BSY0	Aspartyl/asparaginyl beta-hydroxylase OS=Mus musculus OX=10090 GN=Asph PE=1 SV=1	9	0.000192	6.0449
27	P05202	Aspartate aminotransferase, mitochondrial OS=Mus musculus OX=10090 GN=Got2 PE=1 SV=1	9	2.32E-08	4.4416

18	Q91YQ5	Dolichyl-diphosphooligosaccharide--protein glycosyltransferase subunit 1 OS=Mus musculus OX=10090 GN=Rpn1 PE=1 SV=1	9	1.61E-06	4.625
5	F6ZDS4	Nucleoprotein TPR OS=Mus musculus OX=10090 GN=Tpr PE=1 SV=1	9	1.83E-05	4.4842
23	Q922R8	Protein disulfide-isomerase A6 OS=Mus musculus OX=10090 GN=Pdia6 PE=1 SV=3	9	1.26E-06	4.4209
13	Q6ZQL4	WD repeat-containing protein 43 OS=Mus musculus OX=10090 GN=Wdr43 PE=1 SV=2	8	1.78E-06	5.6997
8	Q99NB9	Splicing factor 3B subunit 1 OS=Mus musculus OX=10090 GN=Sf3b1 PE=1 SV=1	8	1.8E-06	4.239
18	Q9D0K2	Succinyl-CoA:3-ketoacid coenzyme A transferase 1, mitochondrial OS=Mus musculus OX=10090 GN=Oxct1 PE=1 SV=1	8	4.51E-06	6.1283
17	Q7TMK9	Heterogeneous nuclear ribonucleoprotein Q OS=Mus musculus OX=10090 GN=Syncrip PE=1 SV=2	8	5.36E-06	5.2875
16	Q61656	Probable ATP-dependent RNA helicase DDX5 OS=Mus musculus OX=10090 GN=Ddx5 PE=1 SV=2	8	8.54E-08	5.997
17	Q9EQ61	Pescadillo homolog OS=Mus musculus OX=10090 GN=Pes1 PE=1 SV=1	8	3.74E-06	6.8605
20	O35737	Heterogeneous nuclear ribonucleoprotein H OS=Mus musculus OX=10090 GN=Hnrnph1 PE=1 SV=3	8	1.13E-08	7.1571
11	P80317	T-complex protein 1 subunit zeta OS=Mus musculus OX=10090 GN=Cct6a PE=1 SV=3	8	0.000279	6.0328
8	Q921M3	Splicing factor 3B subunit 3 OS=Mus musculus OX=10090 GN=Sf3b3 PE=1 SV=1	8	1.51E-06	4.0945
38	P35278	Ras-related protein Rab-5C OS=Mus musculus OX=10090 GN=Rab5c PE=1 SV=2	8	0.000118	5.8140
10	Q9Z1X4	Interleukin enhancer-binding factor 3 OS=Mus musculus OX=10090 GN=Ilf3 PE=1 SV=2	8	3.42E-05	4.8616
49	P62806	Histone H4 OS=Mus musculus OX=10090 GN=H4c1 PE=1 SV=2	8	2.75E-06	4.3331
23	Q9D051	Pyruvate dehydrogenase E1 component subunit beta, mitochondrial OS=Mus musculus OX=10090 GN=Pdhb PE=1 SV=1	8	5.14E-06	6.4463
23	P48962	ADP/ATP translocase 1 OS=Mus musculus OX=10090 GN=Slc25a4 PE=1 SV=4	8	8.41E-06	5.1624
8	Q8R0G9	Nuclear pore complex protein Nup133 OS=Mus musculus OX=10090 GN=Nup133 PE=1 SV=2	8	2.73E-07	4.4343
16	Q9CZU6	Citrate synthase, mitochondrial OS=Mus musculus OX=10090 GN=Cs PE=1 SV=1	8	3.4E-07	3.8184
52	P62821	Ras-related protein Rab-1A OS=Mus musculus OX=10090 GN=Rab1A PE=1 SV=3	8	6.7E-06	6.4918
11	Q8BHN3	Neutral alpha-glucosidase AB OS=Mus musculus OX=10090 GN=Ganab PE=1 SV=1	8	2.46E-07	2.2861
12	P70168	Importin subunit beta-1 OS=Mus musculus OX=10090 GN=Kpnb1 PE=1 SV=2	8	2.46E-06	9.9300
26	P29758	Ornithine aminotransferase, mitochondrial OS=Mus musculus OX=10090 GN=Oat PE=1 SV=1	8	3.14E-08	5.6191
16	Q925I1	ATPase family AAA domain-containing protein 3 OS=Mus musculus OX=10090 GN=Atad3 PE=1 SV=1	8	2.05E-06	7.2097
23	Q9DCW4	Electron transfer flavoprotein subunit beta OS=Mus musculus OX=10090 GN=Etfb PE=1 SV=3	7	6.31E-08	7.4168
11	P11103	Poly [ADP-ribose] polymerase 1 OS=Mus musculus OX=10090 GN=Parp1 PE=1 SV=3	7	1.94E-05	3.6121
25	P27773	Protein disulfide-isomerase A3 OS=Mus musculus OX=10090 GN=Pdia3 PE=1 SV=2	7	5.97E-07	6.6087
16	P24270	Catalase OS=Mus musculus OX=10090 GN=Cat PE=1 SV=4	7	3.95E-06	4.4652
12	Q8BJ71	Nuclear pore complex protein Nup93 OS=Mus musculus OX=10090 GN=Nup93 PE=1 SV=1	7	3.79E-06	4.5065
10	Q8K363	ATP-dependent RNA helicase DDX18 OS=Mus musculus OX=10090 GN=Ddx18 PE=1 SV=1	7	6.53E-06	6.8007
15	Q62167	ATP-dependent RNA helicase DDX3X OS=Mus musculus OX=10090 GN=Ddx3x PE=1 SV=3	7	4.89E-05	5.6836
10	Q9CZU3	Exosome RNA helicase MTR4 OS=Mus musculus OX=10090 GN=Mtrex PE=1 SV=1	7	1.15E-09	6.4522
3	Q9JHU4	Cytoplasmic dynein 1 heavy chain 1 OS=Mus musculus OX=10090 GN=Dync1h1 PE=1 SV=2	7	3.19E-06	3.5711
15	Q8QZT1	Acetyl-CoA acetyltransferase, mitochondrial OS=Mus musculus OX=10090 GN=Acat1 PE=1 SV=1	7	3.38E-08	3.3259
17	Q9CPY7	Cytosol aminopeptidase OS=Mus musculus OX=10090 GN=Lap3 PE=1 SV=3	7	2.5E-07	5.5751
14	P80314	T-complex protein 1 subunit beta OS=Mus musculus OX=10090 GN=Cct2 PE=1 SV=4	7	1.96E-05	5.2267
24	Q9WV55	Vesicle-associated membrane protein-associated protein A OS=Mus musculus OX=10090 GN=Vapa PE=1 SV=2	7	1.38E-06	8.0519
7	P17225	Polypyrimidine tract-binding protein 1 OS=Mus musculus OX=10090 GN=Ptbp1 PE=1 SV=2	7	1.31E-05	5.8948
11	Q9D8E6	60S ribosomal protein L4 OS=Mus musculus OX=10090 GN=Rpl4 PE=1 SV=3	7	8.17E-07	5.8208
15	Q9Z2I8	Succinate--CoA ligase [GDP-forming] subunit beta, mitochondrial OS=Mus musculus OX=10090 GN=Suclg2 PE=1 SV=3	7	4.93E-05	5.1216
7	Q61595	Kinectin OS=Mus musculus OX=10090 GN=Ktn1 PE=1 SV=1	7	1.76E-06	4.9880
5	B2RQC6	CAD protein OS=Mus musculus OX=10090 GN=Cad PE=1 SV=1	7	6.38E-05	2.6317
9	P58252	Elongation factor 2 OS=Mus musculus OX=10090 GN=Eef2 PE=1 SV=2	7	4.15E-07	5.1745
23	Q9CXY6	Interleukin enhancer-binding factor 2 OS=Mus musculus OX=10090 GN=Ilf2 PE=1 SV=1	7	3.72E-07	7.4156
15	O35857	Mitochondrial import inner membrane translocase subunit TIM44 OS=Mus musculus OX=10090 GN=Timm44 PE=1 SV=2	7	0.000312	5.2838
17	Q9D2G2	-residue succinyltransferase component of 2-oxoglutarate dehydrogenase complex, mitochondrial OS=Mus musculus OX=10090 G[illegible]	7	2.58E-06	6.3800
16	Q99JR1	Sideroflexin-1 OS=Mus musculus OX=10090 GN=Sfxn1 PE=1 SV=3	7	4.01E-05	4.7616
11	Q9D6Z1	Nucleolar protein 56 OS=Mus musculus OX=10090 GN=Nop56 PE=1 SV=2	7	9.56E-05	6.4446
18	P35486	uvate dehydrogenase E1 component subunit alpha, somatic form, mitochondrial OS=Mus musculus OX=10090 GN=Pdha1 PE=1 S[illegible]	6	4.3E-07	6.3209
17	O35855	Branched-chain-amino-acid aminotransferase, mitochondrial OS=Mus musculus OX=10090 GN=Bcat2 PE=1 SV=2	6	2.22E-05	5.3250
5	O35134	DNA-directed RNA polymerase I subunit RPA1 OS=Mus musculus OX=10090 GN=Polr1a PE=1 SV=2	6	3.48E-06	4.5224
12	Q9Z0N1	Eukaryotic translation initiation factor 2 subunit 3, X-linked OS=Mus musculus OX=10090 GN=Eif2s3x PE=1 SV=2	6	2.65E-05	6.7660
28	Q64012	RNA-binding protein Raly OS=Mus musculus OX=10090 GN=Raly PE=1 SV=3	6	1.66E-06	5.5327
15	P27659	60S ribosomal protein L3 OS=Mus musculus OX=10090 GN=Rpl3 PE=1 SV=3	6	3.31E-07	4.9812
6	Q6NS46	Protein RRP5 homolog OS=Mus musculus OX=10090 GN=Pdcd11 PE=1 SV=2	6	2.37E-05	6.486
6	P57780	Alpha-actinin-4 OS=Mus musculus OX=10090 GN=Actn4 PE=1 SV=1	6	1.37E-09	6.3769
10	Q9DBY8	Nuclear valosin-containing protein-like OS=Mus musculus OX=10090 GN=Nvl PE=1 SV=1	6	5.99E-05	4.1227
17	P47911	60S ribosomal protein L6 OS=Mus musculus OX=10090 GN=Rpl6 PE=1 SV=3	6	1.01E-06	6.4714
23	P62908	40S ribosomal protein S3 OS=Mus musculus OX=10090 GN=Rps3 PE=1 SV=1	6	6.62E-07	5.5653
17	P10126	Elongation factor 1-alpha 1 OS=Mus musculus OX=10090 GN=Eef1a1 PE=1 SV=3	6	4.7E-06	5.8314
9	Q8VDN2	Sodium/potassium-transporting ATPase subunit alpha-1 OS=Mus musculus OX=10090 GN=Atp1a1 PE=1 SV=1	6	5.69E-07	5.82
4	Q5SWU9	Acetyl-CoA carboxylase 1 OS=Mus musculus OX=10090 GN=Acaca PE=1 SV=1	6	1.55E-05	2.4541
9	Q8VHE0	Translocation protein SEC63 homolog OS=Mus musculus OX=10090 GN=Sec63 PE=1 SV=4	6	8.47E-07	5.8642
40	P18760	Cofilin-1 OS=Mus musculus OX=10090 GN=Cfl1 PE=1 SV=3	6	7.32E-06	2.8422
23	P49312	Heterogeneous nuclear ribonucleoprotein A1 OS=Mus musculus OX=10090 GN=Hnrnpa1 PE=1 SV=2	6	8.46E-09	7.3188
10	P41216	Long-chain-fatty-acid--CoA ligase 1 OS=Mus musculus OX=10090 GN=Acsl1 PE=1 SV=2	6	4.17E-07	5.4814
16	Q9D6R2	Isocitrate dehydrogenase [NAD] subunit alpha, mitochondrial OS=Mus musculus OX=10090 GN=Idh3a PE=1 SV=1	6	1.59E-05	8.2148
15	Q9R112	Sulfide:quinone oxidoreductase, mitochondrial OS=Mus musculus OX=10090 GN=Sqor PE=1 SV=3	6	1.9E-05	3.8406
5	P53569	CCAAT/enhancer-binding protein zeta OS=Mus musculus OX=10090 GN=Cebpz PE=1 SV=2	6	7.68E-07	6.1554
24	P62702	40S ribosomal protein S4, X isoform OS=Mus musculus OX=10090 GN=Rps4x PE=1 SV=2	6	4.29E-06	6.3401
9	P46460	Vesicle-fusing ATPase OS=Mus musculus OX=10090 GN=Nsf PE=1 SV=2	6	6.16E-06	5.0535
9	Q8BU03	Periodic tryptophan protein 2 homolog OS=Mus musculus OX=10090 GN=Pwp2 PE=1 SV=1	6	5.19E-05	4.7085
14	Q61233	Plastin-2 OS=Mus musculus OX=10090 GN=Lcp1 PE=1 SV=4	6	3.75E-06	4.2131
8	P97452	Ribosome biogenesis protein BOP1 OS=Mus musculus OX=10090 GN=Bop1 PE=1 SV=1	6	1.41E-06	3.727
20	P02533	SWISS-PROT:P02533 Tax_Id=9606 Gene_Symbol=KRT14 Keratin, type I cytoskeletal 14	6	0.000362	2.0467
12	Q9EP69	Phosphatidylinositide phosphatase SAC1 OS=Mus musculus OX=10090 GN=Sacm1l PE=1 SV=1	6	2.97E-05	4.4672
11	Q8JZQ2	AFG3-like protein 2 OS=Mus musculus OX=10090 GN=Afg3l2 PE=1 SV=1	6	2.34E-05	6.2813
10	Q9DCD0	6-phosphogluconate dehydrogenase, decarboxylating OS=Mus musculus OX=10090 GN=Pgd PE=1 SV=3	6	1.35E-05	4.8052
24	P62267	40S ribosomal protein S23 OS=Mus musculus OX=10090 GN=Rps23 PE=1 SV=3	6	2.78E-05	5.2300
5	P26039	Talin-1 OS=Mus musculus OX=10090 GN=Tln1 PE=1 SV=2	6	6.56E-07	7.9577
8	Q9CZW4	Long-chain-fatty-acid--CoA ligase 3 OS=Mus musculus OX=10090 GN=Acsl3 PE=1 SV=2	6	1.13E-05	4.355
27	P63101	14-3-3 protein zeta/delta OS=Mus musculus OX=10090 GN=Ywhaz PE=1 SV=1	6	3.23E-06	6.9988
6	Q61316	Heat shock 70 kDa protein 4 OS=Mus musculus OX=10090 GN=Hspa4 PE=1 SV=1	6	1.04E-07	4.9032
18	Q07417	Short-chain specific acyl-CoA dehydrogenase, mitochondrial OS=Mus musculus OX=10090 GN=Acads PE=1 SV=2	6	1.76E-06	4.3960
11	Q8C7V3	U3 small nucleolar RNA-associated protein 15 homolog OS=Mus musculus OX=10090 GN=Utp15 PE=1 SV=1	6	9.85E-06	4.8146
12	Q9DC61	Mitochondrial-processing peptidase subunit alpha OS=Mus musculus OX=10090 GN=Pmpca PE=1 SV=1	6	1.12E-07	4.9199
6	Q3TRM8	Hexokinase-3 OS=Mus musculus OX=10090 GN=Hk3 PE=1 SV=2	6	1.49E-05	5.8369
17	Q91VC3	Eukaryotic initiation factor 4A-III OS=Mus musculus OX=10090 GN=Eif4a3 PE=1 SV=3	6	2.28E-07	4.4844
8	Q61753	D-3-phosphoglycerate dehydrogenase OS=Mus musculus OX=10090 GN=Phgdh PE=1 SV=3	6	2.22E-06	6.4566
9	Q920B9	FACT complex subunit SPT16 OS=Mus musculus OX=10090 GN=Supt16h PE=1 SV=2	5	0.000231	4.9110
12	Q501J6	Probable ATP-dependent RNA helicase DDX17 OS=Mus musculus OX=10090 GN=Ddx17 PE=1 SV=1	5	4.16E-05	4.3502
36	P61027	Ras-related protein Rab-10 OS=Mus musculus OX=10090 GN=Rab10 PE=1 SV=1	5	5.33E-05	6.0778
17	P35550	rRNA 2'-O-methyltransferase fibrillarin OS=Mus musculus OX=10090 GN=Fbl PE=1 SV=2	5	3.67E-07	7.5256
13	Q9DB77	Cytochrome b-c1 complex subunit 2, mitochondrial OS=Mus musculus OX=10090 GN=Uqcrc2 PE=1 SV=1	5	9.35E-06	5.5453
14	P80318	T-complex protein 1 subunit gamma OS=Mus musculus OX=10090 GN=Cct3 PE=1 SV=1	5	2.61E-07	5.1917

11	P14824	Annexin A6 OS=Mus musculus OX=10090 GN=Anxa6 PE=1 SV=3	5	4.52E-06	5.4624
24	Q60930	Voltage-dependent anion-selective channel protein 2 OS=Mus musculus OX=10090 GN=Vdac2 PE=1 SV=2	5	3.4E-05	6.1421
16	P70372	ELAV-like protein 1 OS=Mus musculus OX=10090 GN=Elavl1 PE=1 SV=2	5	6.91E-06	4.8598
22	Q9WTP6	Adenylate kinase 2, mitochondrial OS=Mus musculus OX=10090 GN=Ak2 PE=1 SV=5	5	1.92E-05	5.1215
11	Q9Z2I0	Mitochondrial proton/calcium exchanger protein OS=Mus musculus OX=10090 GN=Letm1 PE=1 SV=1	5	1.14E-05	5.0168
12	P50544	Very long-chain specific acyl-CoA dehydrogenase, mitochondrial OS=Mus musculus OX=10090 GN=Acadvl PE=1 SV=3	5	1.05E-05	3.4866
7	Q8K1R3	Polyribonucleotide nucleotidyltransferase 1, mitochondrial OS=Mus musculus OX=10090 GN=Pnpt1 PE=1 SV=1	5	1.69E-06	5.3041
3	A2AGT5	Cytoskeleton-associated protein 5 OS=Mus musculus OX=10090 GN=Ckap5 PE=1 SV=1	5	7.38E-05	5.9951
11	P97807	Fumarate hydratase, mitochondrial OS=Mus musculus OX=10090 GN=Fh PE=1 SV=3	5	0.000659	4.991
8	Q8BIJ6	Isoleucine--tRNA ligase, mitochondrial OS=Mus musculus OX=10090 GN=Iars2 PE=1 SV=1	5	3.01E-05	3.565
6	Q99P91	Transmembrane glycoprotein NMB OS=Mus musculus OX=10090 GN=Gpnmb PE=1 SV=2	5	1.14E-05	5.7580
3	Q9Z1Q9	Valine--tRNA ligase OS=Mus musculus OX=10090 GN=Vars PE=1 SV=1	5	9.18E-06	4.5686
10	Q9DBG7	Signal recognition particle receptor subunit alpha OS=Mus musculus OX=10090 GN=Srpra PE=1 SV=1	5	9E-05	5.8007
14	P11983	T-complex protein 1 subunit alpha OS=Mus musculus OX=10090 GN=Tcp1 PE=1 SV=3	5	6.09E-05	5.4112
41	Q64475	Histone H2B type 1-B OS=Mus musculus OX=10090 GN=Hist1h2bb PE=1 SV=3	5	9.13E-07	9.4745
9	Q9Z0X1	Apoptosis-inducing factor 1, mitochondrial OS=Mus musculus OX=10090 GN=Aifm1 PE=1 SV=1	5	1.7E-06	5.196
6	Q8BHB4	WD repeat-containing protein 3 OS=Mus musculus OX=10090 GN=Wdr3 PE=1 SV=1	5	3.94E-06	5.2163
8	Q6PIC6	Sodium/potassium-transporting ATPase subunit alpha-3 OS=Mus musculus OX=10090 GN=Atp1a3 PE=1 SV=1	5	6.74E-06	4.9981
29	Q91V41	Ras-related protein Rab-14 OS=Mus musculus OX=10090 GN=Rab14 PE=1 SV=3	5	4.28E-05	4.0819
10	P60843	Eukaryotic initiation factor 4A-I OS=Mus musculus OX=10090 GN=Eif4a1 PE=1 SV=1	5	1.79E-05	4.1302
12	P32067	Lupus La protein homolog OS=Mus musculus OX=10090 GN=Ssb PE=1 SV=1	5	8.27E-07	10.280
17	Q6ZWX6	Eukaryotic translation initiation factor 2 subunit 1 OS=Mus musculus OX=10090 GN=Eif2s1 PE=1 SV=3	5	1.85E-05	5.3367
5	Q8CGC7	Bifunctional glutamate/proline--tRNA ligase OS=Mus musculus OX=10090 GN=Eprs PE=1 SV=4	5	4.7E-07	4.373
6	Q9Z0W3	Nuclear pore complex protein Nup160 OS=Mus musculus OX=10090 GN=Nup160 PE=1 SV=2	5	5.03E-08	4.1488
9	Q9QUJ7	Long-chain-fatty-acid--CoA ligase 4 OS=Mus musculus OX=10090 GN=Acsl4 PE=1 SV=2	5	1.29E-05	3.693
10	Q9JIH2	Nuclear pore complex protein Nup50 OS=Mus musculus OX=10090 GN=Nup50 PE=1 SV=3	5	8.08E-07	5.5812
9	P58742	Aladin OS=Mus musculus OX=10090 GN=Aaas PE=1 SV=1	5	7.51E-05	4.4136
20	Q8BK72	28S ribosomal protein S27, mitochondrial OS=Mus musculus OX=10090 GN=Mrps27 PE=1 SV=2	5	8.37E-06	6.6300
9	P24547	Inosine-5'-monophosphate dehydrogenase 2 OS=Mus musculus OX=10090 GN=Impdh2 PE=1 SV=2	5	5.95E-06	3.973
11	Q99ME9	Nucleolar GTP-binding protein 1 OS=Mus musculus OX=10090 GN=Gtpbp4 PE=1 SV=3	5	7.89E-06	6.0329
14	P16110	Galectin-3 OS=Mus musculus OX=10090 GN=Lgals3 PE=1 SV=3	5	2.22E-06	6.1816
9	Q9JKR6	Hypoxia up-regulated protein 1 OS=Mus musculus OX=10090 GN=Hyou1 PE=1 SV=1	5	5.68E-07	5.4515
20	Q9D1D4	Transmembrane emp24 domain-containing protein 10 OS=Mus musculus OX=10090 GN=Tmed10 PE=1 SV=1	5	6.72E-07	7.0791
19	P17742	Peptidyl-prolyl cis-trans isomerase A OS=Mus musculus OX=10090 GN=Ppia PE=1 SV=2	5	5.21E-06	6.8427
5	Q9CW03	Structural maintenance of chromosomes protein 3 OS=Mus musculus OX=10090 GN=Smc3 PE=1 SV=2	5	1.91E-05	5.0090
10	O35658	Complement component 1 Q subcomponent-binding protein, mitochondrial OS=Mus musculus OX=10090 GN=C1qbp PE=1 SV=1	5	9.56E-06	7.9553
36	P62962	Profilin-1 OS=Mus musculus OX=10090 GN=Pfn1 PE=1 SV=2	5	1.64E-05	5.4144
17	Q8CBB9	Radical S-adenosyl methionine domain-containing protein 2 OS=Mus musculus OX=10090 GN=Rsad2 PE=1 SV=1	5	2.11E-05	7.6311
31	P63242	Eukaryotic translation initiation factor 5A-1 OS=Mus musculus OX=10090 GN=Eif5a PE=1 SV=2	5	5.41E-06	5.4067
8	P42932	T-complex protein 1 subunit theta OS=Mus musculus OX=10090 GN=Cct8 PE=1 SV=3	5	1.33E-06	4.9381
23	P35980	60S ribosomal protein L18 OS=Mus musculus OX=10090 GN=Rpl18 PE=1 SV=3	5	2.58E-06	4.9711
11	Q9CXT8	Mitochondrial-processing peptidase subunit beta OS=Mus musculus OX=10090 GN=Pmpcb PE=1 SV=1	5	9.75E-07	4.52
12	P14733	Lamin-B1 OS=Mus musculus OX=10090 GN=Lmnb1 PE=1 SV=3	5	2.85E-06	4.550
8	Q6NZF1	Zinc finger CCCH domain-containing protein 11A OS=Mus musculus OX=10090 GN=Zc3h11a PE=1 SV=1	5	2.1E-06	6.0703
16	Q9D883	Splicing factor U2AF 35 kDa subunit OS=Mus musculus OX=10090 GN=U2af1 PE=1 SV=4	5	0.000115	6.8358
17	Q9D903	Probable rRNA-processing protein EBP2 OS=Mus musculus OX=10090 GN=Ebna1bp2 PE=2 SV=1	4	8.9E-06	10.091
9	Q9D0E1	Heterogeneous nuclear ribonucleoprotein M OS=Mus musculus OX=10090 GN=Hnrnpm PE=1 SV=3	4	4.48E-07	7.136
16	P09103	Protein disulfide-isomerase OS=Mus musculus OX=10090 GN=P4hb PE=1 SV=2	4	7.23E-06	4.8776
9	P26369	Splicing factor U2AF 65 kDa subunit OS=Mus musculus OX=10090 GN=U2af2 PE=1 SV=3	4	8.05E-07	3.9946
15	Q60932	Voltage-dependent anion-selective channel protein 1 OS=Mus musculus OX=10090 GN=Vdac1 PE=1 SV=3	4	1.11E-06	4.6944
4	Q6P5E4	UDP-glucose:glycoprotein glucosyltransferase 1 OS=Mus musculus OX=10090 GN=Uggt1 PE=1 SV=4	4	1.92E-05	2.8043
12	O70503	Very-long-chain 3-oxoacyl-CoA reductase OS=Mus musculus OX=10090 GN=Hsd17b12 PE=1 SV=1	4	3.08E-06	6.4048
25	Q99LP6	GrpE protein homolog 1, mitochondrial OS=Mus musculus OX=10090 GN=Grpel1 PE=1 SV=1	4	3.25E-07	4.4400
9	Q1HFZ0	RNA cytosine C(5)-methyltransferase NSUN2 OS=Mus musculus OX=10090 GN=Nsun2 PE=1 SV=2	4	3.07E-05	3.2468
3	Q6P5D8	ctural maintenance of chromosomes flexible hinge domain-containing protein 1 OS=Mus musculus OX=10090 GN=Smchd1 PE=1 S	4	5.14E-08	2.7629
9	Q99JX7	Nuclear RNA export factor 1 OS=Mus musculus OX=10090 GN=Nxf1 PE=1 SV=3	4	1.97E-05	4.0286
6	Q5SUA5	Unconventional myosin-Ig OS=Mus musculus OX=10090 GN=Myo1g PE=1 SV=1	4	2.4E-06	4.8008
14	P14869	60S acidic ribosomal protein P0 OS=Mus musculus OX=10090 GN=Rplp0 PE=1 SV=3	4	5.4E-06	8.9462
6	Q8VH51	RNA-binding protein 39 OS=Mus musculus OX=10090 GN=Rbm39 PE=1 SV=2	4	3.95E-05	5.8608
27	P35700	Peroxiredoxin-1 OS=Mus musculus OX=10090 GN=Prdx1 PE=1 SV=1	4	5.59E-09	6.0014
10	P40124	Adenylyl cyclase-associated protein 1 OS=Mus musculus OX=10090 GN=Cap1 PE=1 SV=4	4	1.93E-06	4.8246
8	Q640M1	U3 small nucleolar RNA-associated protein 14 homolog A OS=Mus musculus OX=10090 GN=Utp14a PE=1 SV=1	4	3.55E-07	5.7199
9	Q8R2N2	U3 small nucleolar RNA-associated protein 4 homolog OS=Mus musculus OX=10090 GN=Utp4 PE=2 SV=3	4	6.49E-05	4.0923
14	Q922Q4	Pyrroline-5-carboxylate reductase 2 OS=Mus musculus OX=10090 GN=Pycr2 PE=1 SV=1	4	0.000152	3.576
5	Q9WV70	Nucleolar complex protein 2 homolog OS=Mus musculus OX=10090 GN=Noc2l PE=1 SV=2	4	8.78E-08	6.595
5	Q8BKC5	Importin-5 OS=Mus musculus OX=10090 GN=Ipo5 PE=1 SV=3	4	1.82E-07	4.6744
8	Q9WUA2	Phenylalanine--tRNA ligase beta subunit OS=Mus musculus OX=10090 GN=Farsb PE=1 SV=2	4	8.15E-06	5.1188
11	P29341	Polyadenylate-binding protein 1 OS=Mus musculus OX=10090 GN=Pabpc1 PE=1 SV=2	4	1.4E-05	7.0327
10	Q8C0I1	Alkyldihydroxyacetonephosphate synthase, peroxisomal OS=Mus musculus OX=10090 GN=Agps PE=1 SV=1	4	6.27E-05	7.107
12	Q8QZS1	3-hydroxyisobutyryl-CoA hydrolase, mitochondrial OS=Mus musculus OX=10090 GN=Hibch PE=1 SV=1	4	2.88E-06	7.6628
8	Q8BH74	Nuclear pore complex protein Nup107 OS=Mus musculus OX=10090 GN=Nup107 PE=1 SV=1	4	3.97E-05	3.6775
25	P14131	40S ribosomal protein S16 OS=Mus musculus OX=10090 GN=Rps16 PE=1 SV=4	4	1.19E-06	5.4449
14	P50580	Proliferation-associated protein 2G4 OS=Mus musculus OX=10090 GN=Pa2g4 PE=1 SV=3	4	1.01E-06	6.0702
4	Q6PFD9	Nuclear pore complex protein Nup98-Nup96 OS=Mus musculus OX=10090 GN=Nup98 PE=1 SV=2	4	7.16E-06	5.3804
12	P09411	Phosphoglycerate kinase 1 OS=Mus musculus OX=10090 GN=Pgk1 PE=1 SV=4	4	1.15E-06	7.5020
4	Q64737	Trifunctional purine biosynthetic protein adenosine-3 OS=Mus musculus OX=10090 GN=Gart PE=1 SV=3	4	6.01E-06	5.1619
4	Q61879	Myosin-10 OS=Mus musculus OX=10090 GN=Myh10 PE=1 SV=2	4	0.000658	7.4256
7	Q76MZ3	e/threonine-protein phosphatase 2A 65 kDa regulatory subunit A alpha isoform OS=Mus musculus OX=10090 GN=Ppp2r1a PE=1 S	4	6.6E-05	8.775
10	Q14C51	Pentatricopeptide repeat domain-containing protein 3, mitochondrial OS=Mus musculus OX=10090 GN=Ptcd3 PE=1 SV=2	4	7.61E-06	4.1146
4	Q60864	Stress-induced-phosphoprotein 1 OS=Mus musculus OX=10090 GN=Stip1 PE=1 SV=1	4	0.000208	8.7708
11	Q9D8N0	Elongation factor 1-gamma OS=Mus musculus OX=10090 GN=Eef1g PE=1 SV=3	4	2.9E-05	5.103
3	P06800	Receptor-type tyrosine-protein phosphatase C OS=Mus musculus OX=10090 GN=Ptprc PE=1 SV=4	4	8.54E-06	7.7532
20	Q99JI6	Ras-related protein Rap-1b OS=Mus musculus OX=10090 GN=Rap1b PE=1 SV=2	4	6.54E-06	5.4047
13	P62814	V-type proton ATPase subunit B, brain isoform OS=Mus musculus OX=10090 GN=Atp6v1b2 PE=1 SV=1	4	1.75E-06	4.9953
33	P63276	40S ribosomal protein S17 OS=Mus musculus OX=10090 GN=Rps17 PE=1 SV=2	4	0.00024	6.188
5	Q80VL1	Tudor and KH domain-containing protein OS=Mus musculus OX=10090 GN=Tdrkh PE=1 SV=1	4	2.13E-07	6.8489
7	Q8VIJ6	Splicing factor, proline- and glutamine-rich OS=Mus musculus OX=10090 GN=Sfpq PE=1 SV=1	4	0.000129	5.871
14	Q9ESX5	H/ACA ribonucleoprotein complex subunit DKC1 OS=Mus musculus OX=10090 GN=Dkc1 PE=1 SV=4	4	0.000558	4.488
4	Q9JIX8	Apoptotic chromatin condensation inducer in the nucleus OS=Mus musculus OX=10090 GN=Acin1 PE=1 SV=3	4	6.82E-05	5.4371
7	Q8K224	RNA cytidine acetyltransferase OS=Mus musculus OX=10090 GN=Nat10 PE=1 SV=1	4	3.56E-06	5.1726
4	Q01320	DNA topoisomerase 2-alpha OS=Mus musculus OX=10090 GN=Top2a PE=1 SV=2	4	6.6E-07	8.3134
6	Q62318	Transcription intermediary factor 1-beta OS=Mus musculus OX=10090 GN=Trim28 PE=1 SV=3	4	4.58E-06	4.5501
9	Q922K7	Probable 28S rRNA (cytosine-C(5))-methyltransferase OS=Mus musculus OX=10090 GN=Nop2 PE=1 SV=1	4	7.41E-08	6.5478
8	P46471	26S proteasome regulatory subunit 7 OS=Mus musculus OX=10090 GN=Psmc2 PE=1 SV=5	4	3.98E-05	9.6818
3	Q99PL5	Ribosome-binding protein 1 OS=Mus musculus OX=10090 GN=Rrbp1 PE=1 SV=2	4	4.41E-07	7.921

7	B9EJ86	Oxysterol-binding protein-related protein 8 OS=Mus musculus OX=10090 GN=Osbpl8 PE=1 SV=1	4	2.11E-06	5.9120
5	Q5SYD0	Unconventional myosin-Id OS=Mus musculus OX=10090 GN=Myo1d PE=1 SV=1	4	4.92E-06	3.7575
7	P06745	Glucose-6-phosphate isomerase OS=Mus musculus OX=10090 GN=Gpi PE=1 SV=4	4	1.32E-05	4.7268
5	Q99P88	Nuclear pore complex protein Nup155 OS=Mus musculus OX=10090 GN=Nup155 PE=1 SV=1	4	2.76E-05	4.0037
6	P10852	4F2 cell-surface antigen heavy chain OS=Mus musculus OX=10090 GN=Slc3a2 PE=1 SV=1	4	9.57E-06	5.7220
6	Q8K0D5	Elongation factor G, mitochondrial OS=Mus musculus OX=10090 GN=Gfm1 PE=1 SV=1	4	3.96E-06	3.7575
24	Q9WVA4	Transgelin-2 OS=Mus musculus OX=10090 GN=Tagln2 PE=1 SV=4	4	2.71E-06	8.3292
5	P58281	Dynamin-like 120 kDa protein, mitochondrial OS=Mus musculus OX=10090 GN=Opa1 PE=1 SV=1	4	9.56E-06	5.3864
3	Q8BTM8	Filamin-A OS=Mus musculus OX=10090 GN=Flna PE=1 SV=5	4	1E-06	3.588
11	Q3U0V1	Far upstream element-binding protein 2 OS=Mus musculus OX=10090 GN=Khsrp PE=1 SV=2	4	7.32E-05	2.6147
37	P62984	Ubiquitin-60S ribosomal protein L40 OS=Mus musculus OX=10090 GN=Uba52 PE=1 SV=2	4	4.23E-06	5.1285
2	Q9EPU4	Cleavage and polyadenylation specificity factor subunit 1 OS=Mus musculus OX=10090 GN=Cpsf1 PE=1 SV=1	4	0.000103	4.0066
10	Q9Z1N5	Spliceosome RNA helicase Ddx39b OS=Mus musculus OX=10090 GN=Ddx39b PE=1 SV=1	4	2.59E-06	6.4739
11	P14148	60S ribosomal protein L7 OS=Mus musculus OX=10090 GN=Rpl7 PE=1 SV=2	4	6.2E-07	3.2219
13	P30681	High mobility group protein B2 OS=Mus musculus OX=10090 GN=Hmgb2 PE=1 SV=3	4	7.23E-06	6.7944
8	Q9CWJ9	Bifunctional purine biosynthesis protein PURH OS=Mus musculus OX=10090 GN=Atic PE=1 SV=2	4	3.01E-06	4.2026
30	Q9D1G1	Ras-related protein Rab-1B OS=Mus musculus OX=10090 GN=Rab1b PE=1 SV=1	4	7.99E-05	4.750
10	P20108	Thioredoxin-dependent peroxide reductase, mitochondrial OS=Mus musculus OX=10090 GN=Prdx3 PE=1 SV=1	4	8.51E-06	3.9806
13	Q9CQA3	Succinate dehydrogenase [ubiquinone] iron-sulfur subunit, mitochondrial OS=Mus musculus OX=10090 GN=Sdhb PE=1 SV=1	4	7.19E-06	4.8654
11	Q9CY58	Plasminogen activator inhibitor 1 RNA-binding protein OS=Mus musculus OX=10090 GN=Serbp1 PE=1 SV=2	4	9.11E-07	7.5901
11	Q60766	Immunity-related GTPase family M protein 1 OS=Mus musculus OX=10090 GN=Irgm1 PE=1 SV=1	4	5.67E-05	5.7087
10	Q9R0X4	Acyl-coenzyme A thioesterase 9, mitochondrial OS=Mus musculus OX=10090 GN=Acot9 PE=1 SV=1	4	8.76E-05	5.7041
8	Q6ZQ58	La-related protein 1 OS=Mus musculus OX=10090 GN=Larp1 PE=1 SV=3	4	8.68E-05	10.790
9	Q6A068	Cell division cycle 5-like protein OS=Mus musculus OX=10090 GN=Cdc5l PE=1 SV=2	4	3.82E-07	6.9685
8	Q9R233	Tapasin OS=Mus musculus OX=10090 GN=Tapbp PE=1 SV=2	4	3.22E-06	5.9263
7	Q91WM3	U3 small nucleolar RNA-interacting protein 2 OS=Mus musculus OX=10090 GN=Rrp9 PE=1 SV=1	4	0.000123	6.4939
11	Q922Q8	Leucine-rich repeat-containing protein 59 OS=Mus musculus OX=10090 GN=Lrrc59 PE=1 SV=1	4	1.2E-05	9.7092
12	P53026	60S ribosomal protein L10a OS=Mus musculus OX=10090 GN=Rpl10a PE=1 SV=3	4	0.001338	4.4207
8	Q8VDF2	E3 ubiquitin-protein ligase UHRF1 OS=Mus musculus OX=10090 GN=Uhrf1 PE=1 SV=2	4	5.39E-08	5.7847
13	Q6PHZ2	Calcium/calmodulin-dependent protein kinase type II subunit delta OS=Mus musculus OX=10090 GN=Camk2d PE=1 SV=1	4	2.15E-05	3.4608
11	Q9Z2X1	Heterogeneous nuclear ribonucleoprotein F OS=Mus musculus OX=10090 GN=Hnrnpf PE=1 SV=3	4	2.94E-05	11.520
4	P97386	DNA ligase 3 OS=Mus musculus OX=10090 GN=Lig3 PE=1 SV=2	4	7.41E-05	4.9959
20	Q6PDM2	Serine/arginine-rich splicing factor 1 OS=Mus musculus OX=10090 GN=Srsf1 PE=1 SV=3	4	7.36E-06	5.5026
10	Q8VDW0	ATP-dependent RNA helicase DDX39A OS=Mus musculus OX=10090 GN=Ddx39a PE=1 SV=1	4	4.93E-05	3.7661
5	Q02053	Ubiquitin-like modifier-activating enzyme 1 OS=Mus musculus OX=10090 GN=Uba1 PE=1 SV=1	4	5.74E-06	4.6481
4	Q80X82	Symplekin OS=Mus musculus OX=10090 GN=Sympk PE=1 SV=1	4	2.69E-05	5.8658
11	P68040	Receptor of activated protein C kinase 1 OS=Mus musculus OX=10090 GN=Rack1 PE=1 SV=3	4	4.72E-06	5.0671
6	O89053	Coronin-1A OS=Mus musculus OX=10090 GN=Coro1a PE=1 SV=5	4	1.88E-05	5.9341
7	P46061	Ran GTPase-activating protein 1 OS=Mus musculus OX=10090 GN=Rangap1 PE=1 SV=2	4	4.18E-05	6.4269
3	P70700	DNA-directed RNA polymerase I subunit RPA2 OS=Mus musculus OX=10090 GN=Polr1b PE=1 SV=2	4	3.68E-05	5.5475
4	Q8VDM4	26S proteasome non-ATPase regulatory subunit 2 OS=Mus musculus OX=10090 GN=Psmd2 PE=1 SV=1	4	3.14E-06	3.8679
10	P80315	T-complex protein 1 subunit delta OS=Mus musculus OX=10090 GN=Cct4 PE=1 SV=3	4	7.78E-07	2.9784
5	Q8VCY6	U3 small nucleolar RNA-associated protein 6 homolog OS=Mus musculus OX=10090 GN=Utp6 PE=2 SV=1	4	2.36E-05	2.6673
2	Q61687	Transcriptional regulator ATRX OS=Mus musculus OX=10090 GN=Atrx PE=1 SV=3	4	0.000137	4.3312
4	O70318	Band 4.1-like protein 2 OS=Mus musculus OX=10090 GN=Epb41l2 PE=1 SV=2	4	0.000359	2.5916
3	Q8K4Z5	Splicing factor 3A subunit 1 OS=Mus musculus OX=10090 GN=Sf3a1 PE=1 SV=1	4	1.98E-05	3.3488
18	Q921F2	TAR DNA-binding protein 43 OS=Mus musculus OX=10090 GN=Tardbp PE=1 SV=1	4	4.44E-07	8.0159
7	Q9DBL1	Short/branched chain specific acyl-CoA dehydrogenase, mitochondrial OS=Mus musculus OX=10090 GN=Acadsb PE=1 SV=1	4	1.39E-05	2.8236
9	P32020	Non-specific lipid-transfer protein OS=Mus musculus OX=10090 GN=Scp2 PE=1 SV=3	4	7.94E-07	8.9822
5	Q8BHD7	Polypyrimidine tract-binding protein 3 OS=Mus musculus OX=10090 GN=Ptbp3 PE=1 SV=1	4	0.000129	7.043
16	Q8BL97	Serine/arginine-rich splicing factor 7 OS=Mus musculus OX=10090 GN=Srsf7 PE=1 SV=1	4	3.07E-06	5.0575
10	Q9CZR8	Elongation factor Ts, mitochondrial OS=Mus musculus OX=10090 GN=Tsfm PE=1 SV=1	4	0.000134	4.4637
6	P51660	Peroxisomal multifunctional enzyme type 2 OS=Mus musculus OX=10090 GN=Hsd17b4 PE=1 SV=3	4	9.97E-07	5.3644
25	Q9CQR4	Acyl-coenzyme A thioesterase 13 OS=Mus musculus OX=10090 GN=Acot13 PE=1 SV=1	4	1.35E-06	4.5910
2	O88379	Bromodomain adjacent to zinc finger domain protein 1A OS=Mus musculus OX=10090 GN=Baz1a PE=1 SV=3	4	3.64E-06	7.0294
6	Q9CZ91	Serum response factor-binding protein 1 OS=Mus musculus OX=10090 GN=Srfbp1 PE=1 SV=1	4	2.77E-05	10.730
3	Q8BX70	Vacuolar protein sorting-associated protein 13C OS=Mus musculus OX=10090 GN=Vps13c PE=1 SV=2	4	0.018393	3.0064
